# Supplementary material for: The N-terminal region of DNMT3A engages the nucleosome surface to aid chromatin recruitment
Source: EMBO Rep. 2024 Nov 11;25(12):5743–79. doi: 10.1038/s44319-024-00306-3 (PMC11624362; doi:10.1038/s44319-024-00306-3)
Supplement: Supplementary file 1 — Appendix [file 44319_2024_306_MOESM1_ESM.pdf]

Appendix Figures and Tables

The N-terminal region of DNMT3A engages the nucleosome surface to guide recruitment.

Hannah Wapenaar[1], Gillian Clifford[1], Willow Rolls[1], Moira Pasquier[2], Hayden Burdett[1], Yujie Zhang[1], Gauri Deák[1], Juan Zou[1], Christos Spanos[1], Mark R. D. Taylor[1], Jacquie Mills[1,3], James A. Watson[1], Dhananjay Kumar[1], Richard Clark[4], Alakta Das[1], Devisree Valsakumar[1,5], Janice Bramham[6], Philipp Voigt[1,5], Duncan Sproul[2,7], Marcus D. Wilson[1,6]

1 Wellcome Centre for Cell Biology, University of Edinburgh, Michael Swann Building, Kings Buildings, Mayfield Road, Edinburgh, EH9 3JR, UK

2 MRC Human Genetics Unit, Institute of Genetics and Cancer, University of Edinburgh, UK.

3 present address: Cancer Research UK Scotland Institute, University of Glasgow, Bearsden, Glasgow, G61 1BD, UK

4 Edinburgh Clinical Research Facility, University of Edinburgh, Edinburgh, UK.

5 Epigenetics Programme, Babraham Institute, Cambridge CB22 3AT, UK

6 Institute of Quantitative Biology, Biochemistry and Biotechnology, University of Edinburgh, Michael Swann Building, Edinburgh EH9 3JR, UK

7 CRUK Edinburgh Centre, Institute of Genetics and Cancer, University of Edinburgh, UK.

*Correspondence should be addressed to marcus.wilson@ed.ac.uk

Table of contents

Appendix Figure S1. Cryo-EM data processing scheme for DNMT3A1-DNMT3L:nucleosome H2AK119ub....................3

Appendix Figure S2. Cryo-EM structure determination and validation for DNMT3A1-DNMT3L:nucleosome H2AK119ub complex. 5

Appendix Figure S3. Characterising DNMT3A1 recruitment to Polycomb regions via H2AK119ub binding. 7

Appendix Figure S4. Validation of DNMT3A1 UDR nucleosome interaction 9

Appendix Figure S5. Investigating DNMT3A1 ubiquitin site specificity....................11

Appendix Figure S6. DNMT3A can concurrently mediated interactions with acidic patch, H3K36me2 and H2AK119ub....................13

Appendix Table S1. Cryo-EM data collection, refinement and validation statistics 14

Appendix Table S2: Apparent binding affinities for DNMT3A constructs used in this study..15

Appendix Table S3. Expression constructs used in this study....................16

Appendix Table S4. DNA sequences used for wrapping nucleosomes....................18

Appendix Table S5. Read alignment statistics from Nanopore sequencing of in vitro methyltransferase assays. 19

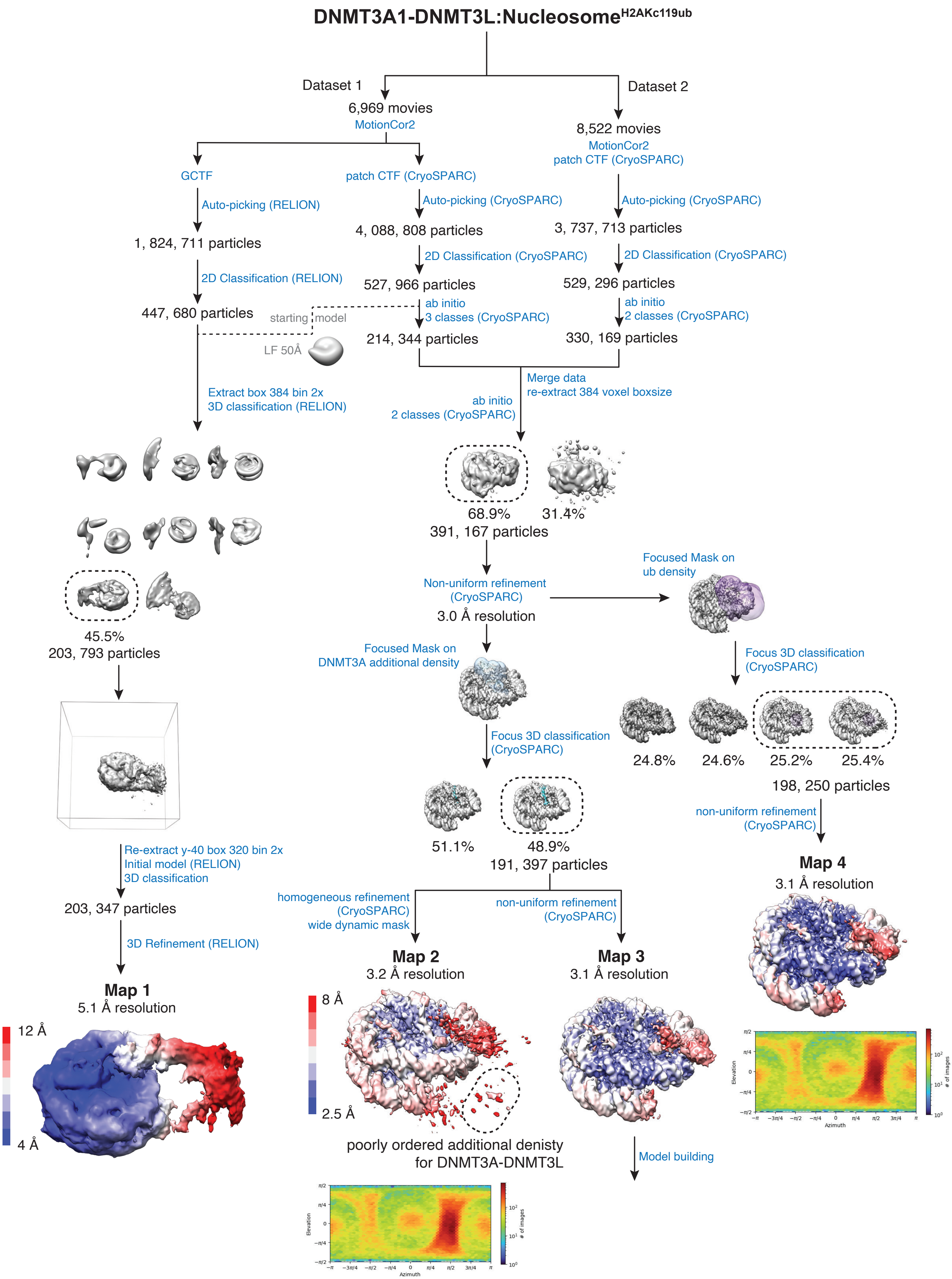
DNMT3A1-DNMT3L:Nucleosome^{H2AKc119ub}
Dataset 1
6,969 movies
MotionCor2
Dataset 2
8,522 movies
MotionCor2
patch CTF (CryoSPARC)
GCTF
patch CTF (CryoSPARC)
Auto-picking (RELION)
Auto-picking (CryoSPARC)
Auto-picking (CryoSPARC)
1, 824, 711 particles
4, 088, 808 particles
3, 737, 713 particles
2D Classification (RELION)
2D Classification (CryoSPARC)
2D Classification (CryoSPARC)
447, 680 particles
527, 966 particles
529, 296 particles
starting model
LF 50Å
ab initio
3 classes (CryoSPARC)
ab initio
2 classes (CryoSPARC)
214, 344 particles
330, 169 particles
Merge data
re-extract 384 voxel boxsize
ab initio
2 classes (CryoSPARC)
Extract box 384 bin 2x
3D classification (RELION)
68.9%
31.4%
391, 167 particles
45.5%
203, 793 particles
Non-uniform refinement
(CryoSPARC)
3.0 Å resolution
Focused Mask on
ub density
Focused Mask on
DNMT3A additional density
Focus 3D classification
(CryoSPARC)
24.8%
24.6%
25.2%
25.4%
198, 250 particles
Focus 3D classification
(CryoSPARC)
51.1%
48.9%
191, 397 particles
non-uniform refinement
(CryoSPARC)
Re-extract y-40 box 320 bin 2x
Initial model (RELION)
3D classification
203, 347 particles
3D Refinement (RELION)
homogeneous refinement
(CryoSPARC)
wide dynamic mask
non-uniform refinement
(CryoSPARC)
Map 1
5.1 Å resolution
12 Å
4 Å
Map 2
3.2 Å resolution
8 Å
2.5 Å
Map 3
3.1 Å resolution
Map 4
3.1 Å resolution
poorly ordered additional denisty
for DNMT3A-DNMT3L
Model building
Elevation
Azimuth
# of images

Appendix Figure S1. Cryo-EM data processing scheme for DNMT3A1-DNMT3L:nucleosome H2AK119ub.

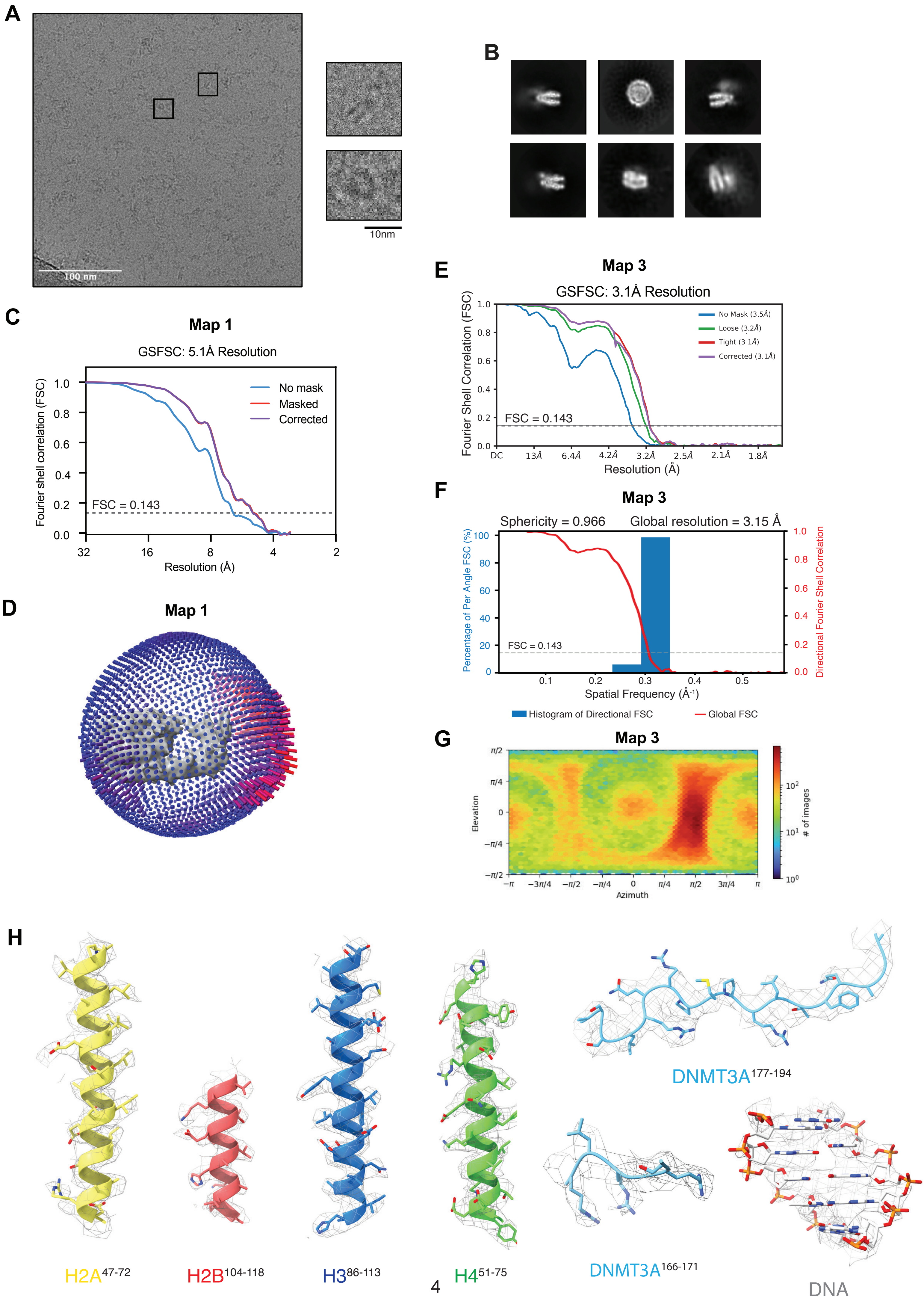

A
100 nm
10nm
B
C
Map 1
GSFSC: 5.1Å Resolution
Fourier shell correlation (FSC)
No mask
Masked
Corrected
FSC = 0.143
Resolution (Å)
D
Map 1
E
Map 3
GSFSC: 3.1Å Resolution
Fourier Shell Correlation (FSC)
No Mask (3.5Å)
Loose (3.2Å)
Tight (3.1Å)
Corrected (3.1Å)
FSC = 0.143
Resolution (Å)
F
Map 3
Sphericity = 0.966
Global resolution = 3.15 Å
Percentage of Per Angle FSC (%)
Directional Fourier Shell Correlation
FSC = 0.143
Spatial Frequency (Å-1)
Histogram of Directional FSC
Global FSC
G
Map 3
Elevation
Azimuth
# of images
H
H2A47-72
H2B104-118
H386-113
H451-75
DNMT3A177-194
DNMT3A166-171
DNA

Appendix Figure S2. Cryo-EM structure determination and validation for DNMT3A1-DNMT3L:nucleosome H2AK119ub complex.

A. Representative micrograph of cryo-EM of DNMT3A1-DNMT3L-StrepII on H2AKc119ub nucleosomes.

B. Representative 2D class images from first round of 2D classification in RELION of DNMT3A1-DNMT3L-StrepII on H2AKc119ub nucleosomes. Top views and side views shown, with additional, delocalised non-nucleosomal density seen in the projections.

C. Gold standard Fourier shell correlation (GS-FSC) of the reconstruction of DNMT3A1-DNMT3A1 (S2 map1) determined in RELION. Resolution determined to be 5.1 Å using the 0.143 threshold

D. Euler Angular distribution plot of DNMT3A1-DNMT3L-StrepII:H2AKc119ub nucleosomes (S2 map1). Rod heights and colours are proportional to the number of particles in each direction.

E. GS-FSC curve for final masked map (S2 map3) DNMT3A1^{UDR}:nucleosome^{H2AK19ub}, including unmasked and masked curves. Focused on nucleosome and adjacent density to reach higher detail than above. The dotted line corresponds to 0.143 threshold.

F. Three dimensional FSC for final DNMT3A1^{UDR}:nucleosome^{H2AK19ub} map (S2 map3) showing global FSC curve (red) and overlap of histogram of directional FSC with the major peak correlating with the global resolution estimate.

G. Euler angle distribution plot of all particles used in the final map (S2 map3). Despite some preferred orientations (red on heat map) no anisotropy in model was observed.

H. Representative regions of the DNMT3A1^{UDR}:nucleosome^{H2AK19ub} cryo-EM density map (S2 map3) for the different components of the complex. The densities for histones, DNA, and DNMT3A1 UDR region are depicted at a contour level of 0.25 (0.3 DNA) using a final map sharpened map with a b-factor of -50, and the corresponding structural models coloured as in Fig 1C and 2A & B.

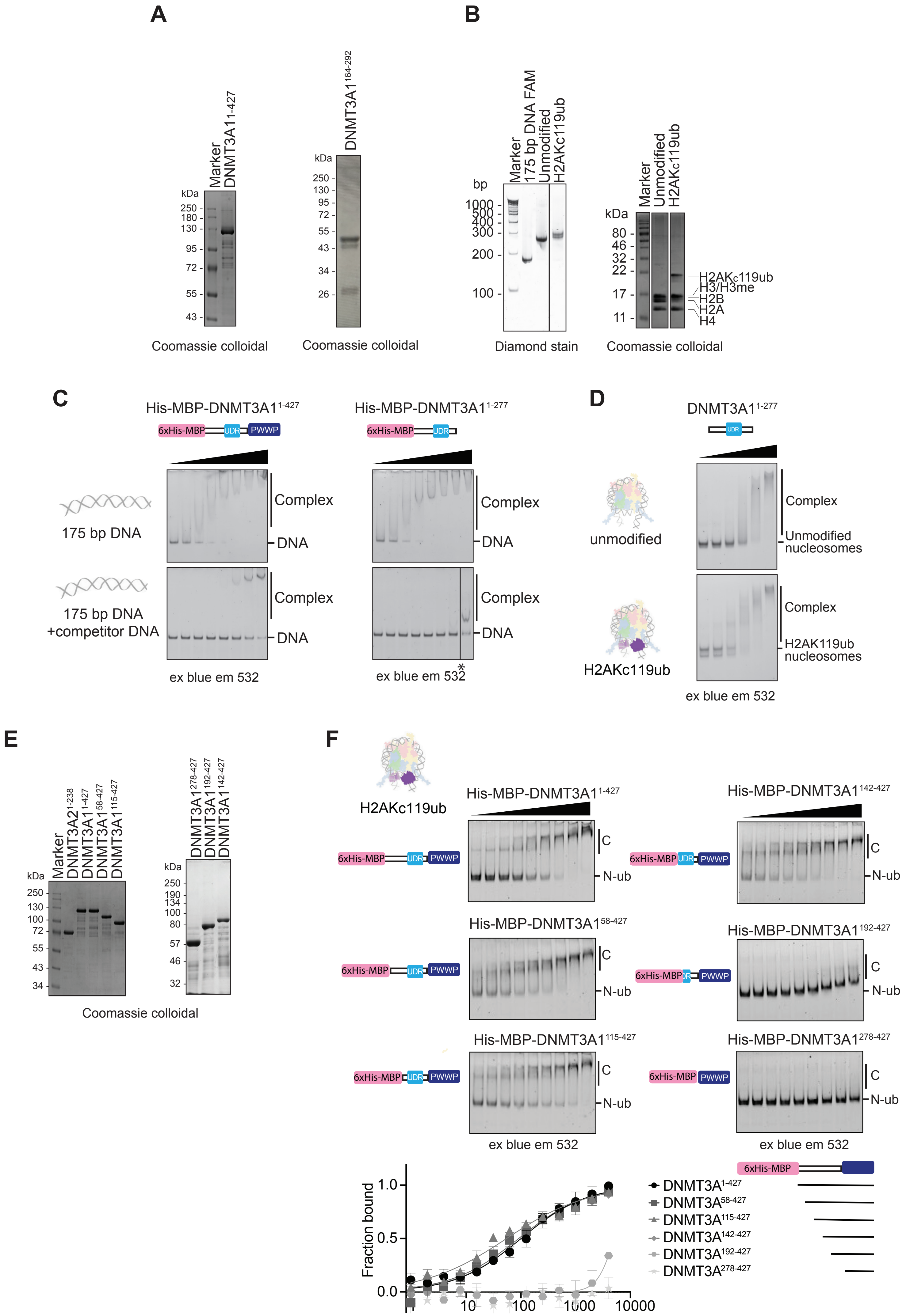
A
B
Marker
DNMT3A1 1-427
kDa
250
180
130
95
72
55
43
Coomassie colloidal
DNMT3A1 164-292
kDa
250
130
95
72
55
43
34
26
Coomassie colloidal
Marker
175 bp DNA FAM
Unmodified
H2AKc119ub
bp
1000
500
400
300
200
100
Diamond stain
Marker
Unmodified
H2AKc119ub
kDa
80
46
32
22
17
11
H2AKc119ub
H3/H3me
H2B
H2A
H4
Coomassie colloidal
C
His-MBP-DNMT3A1 1-427
6xHis-MBP
UDR
PWWP
His-MBP-DNMT3A1 1-277
175 bp DNA
175 bp DNA
+competitor DNA
Complex
DNA
ex blue em 532
D
DNMT3A1 1-277
unmodified
H2AKc119ub
Complex
Unmodified nucleosomes
H2AK119ub nucleosomes
ex blue em 532
E
Marker
DNMT3A2 1-238
DNMT3A1 1-427
DNMT3A1 58-427
DNMT3A1 115-427
DNMT3A1 278-427
DNMT3A1 192-427
DNMT3A1 142-427
kDa
250
130
100
72
55
43
34
kDa
250
190
134
100
80
57
46
32
Coomassie colloidal
F
H2AKc119ub
His-MBP-DNMT3A1 1-427
His-MBP-DNMT3A1 142-427
His-MBP-DNMT3A1 58-427
His-MBP-DNMT3A1 192-427
His-MBP-DNMT3A1 115-427
His-MBP-DNMT3A1 278-427
C
N-ub
ex blue em 532
Fraction bound
1.0
0.5
0.0
10
100
1000
10000
[proteins] (nM)
DNMT3A 1-427
DNMT3A 58-427
DNMT3A 115-427
DNMT3A 142-427
DNMT3A 192-427
DNMT3A 278-427

Appendix Figure S3. Characterising DNMT3A1 recruitment to Polycomb regions via H2AK119ub binding.

A. SDS-PAGE gel of purified DNMT3A proteins used in Fig 1D & E.

B. Native gels and SDS-PAGE gels of nucleosomes used in Fig 1D & E. The unmodified, and DNA lanes on SDS-PAGE and Native gels are duplicated from Fig EV3A.

C. EMSA showing binding of DNMT3A1$^{1-427}$ and DNMT3A1$^{1-277}$ to free 5' FAM labelled 175bp DNA with and without competitor DNA (0.5 mg/ml salmon sperm DNA). 5' FAM labelled tracer was incubated with 0-8000 nM (2x dilution series) of His-MBP-DNMT3A1$^{1-427}$. Representative gels show concentrations 8-1000 nM. Experiment was repeated twice. *Indicates skipped well while loading

D. EMSAs showing binding of DNMT3A1 1-277 after cleaving His-MBP tag to nucleosomes wrapped with FAM labelled 175bp DNA. DNMT3A1 1-277 (0 - 10μM, 2x dilution series) were mixed with unmodified and H2AKc119ub nucleosomes. Complexes were resolved by native-PAGE and imaged using blue light excitation and 532nm emission filters. Gels show concentrations 126 - 4000 nM.

E. SDS-PAGE gels showing purified protein constructs used in S3F.

F. EMSA comparing binding of DNMT3A1 constructs with different lengths of the N-terminal region to H2AKc119ub nucleosomes wrapped with 5' FAM labelled 175bp Widom601 DNA. Limiting amounts (2.3 nM) of H3K36me2 nucleosomes were incubated with increasing concentrations (0-8000 nM) of His-MBP-DNMT3A1 constructs. Complexes were resolved by native-PAGE and imaged using blue light excitation and 532nm emission filters. Gels show concentrations 25 nM-8000 nM for clarity, quantification was done with full concentration series in triplicate.

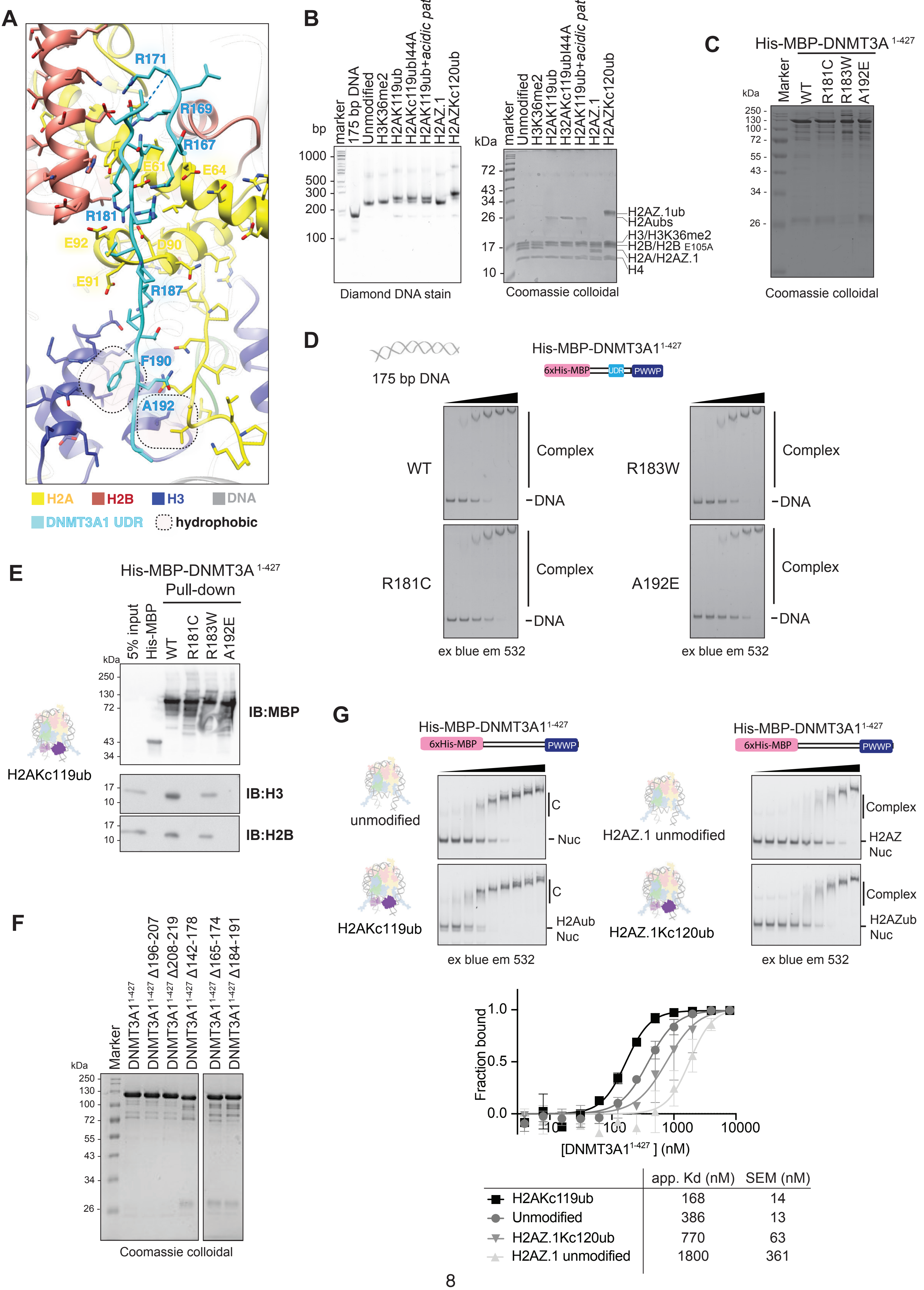
A
R171
R169
R167
E61
E64
R181
E92
D90
E91
R187
F190
A192
H2A
H2B
H3
DNA
DNMT3A1 UDR
hydrophobic
B
bp
marker
175 bp DNA
Unmodified
H3K36me2
H2AK119ub
H2AKc119ubI44A
H2AK119ub+acidic patch
H2AZ.1
H2AZKc120ub
1000
500
300
200
100
Diamond DNA stain
kDa
marker
Unmodified
H3K36me2
H2AK119ub
H32AKc119ubI44A
H2AK119ub+acidic patch
H2AZ.1
H2AZKc120ub
72
43
34
26
17
10
H2AZ.1ub
H2Aubs
H3/H3K36me2
H2B/H2B E105A
H2A/H2AZ.1
H4
Coomassie colloidal
C
His-MBP-DNMT3A 1-427
kDa
Marker
WT
R181C
R183W
A192E
250
130
100
72
55
43
34
26
Coomassie colloidal
D
175 bp DNA
His-MBP-DNMT3A1 1-427
6xHis-MBP
UDR
PWWP
WT
Complex
DNA
R183W
Complex
DNA
R181C
Complex
DNA
A192E
Complex
DNA
ex blue em 532
ex blue em 532
E
His-MBP-DNMT3A 1-427
Pull-down
5% input
His-MBP
WT
R181C
R183W
A192E
kDa
250
130
72
43
34
IB:MBP
17
10
IB:H3
17
10
IB:H2B
H2AKc119ub
F
kDa
Marker
DNMT3A1 1-427
DNMT3A1 1-427 Δ196-207
DNMT3A1 1-427 Δ208-219
DNMT3A1 1-427 Δ142-178
DNMT3A1 1-427 Δ165-174
DNMT3A1 1-427 Δ184-191
250
130
100
72
55
43
34
26
Coomassie colloidal
G
His-MBP-DNMT3A1 1-427
6xHis-MBP
PWWP
unmodified
C
Nuc
H2AKc119ub
C
H2Aub
Nuc
ex blue em 532
His-MBP-DNMT3A1 1-427
6xHis-MBP
PWWP
H2AZ.1 unmodified
Complex
H2AZ
Nuc
H2AZ.1Kc120ub
Complex
H2AZub
Nuc
ex blue em 532
Fraction bound
1.0
0.5
0.0
10
100
1000
10000
[DNMT3A1 1-427] (nM)
app. Kd (nM)
SEM (nM)
H2AKc119ub 168 14
Unmodified 386 13
H2AZ.1Kc120ub 770 63
H2AZ.1 unmodified 1800 361

Appendix Figure S4. Validation of DNMT3A1 UDR nucleosome interaction

A. Magnified view of DNMT3A1^{UDR}:nucleosome^{H2AK19ub} model focusing on the acidic patch interaction and H3-H3'-H2A interaction. Arg-181 interacts directly with carboxylate groups of Glu61, Asp90, and Glu92 in H2A. Arg-167, Arg-169 and Arg-171 form stabilising additional interactions along the acidic patch. Phe-190 and Ala-192 interact in hydrophobic regions, boxed.

B. Native gels and SDS-PAGE gels of modified and unmodified nucleosomes wrapped with 5' FAM labelled 175bp Widom601 DNA used in Fig 2 and Fig S4. H2A.Z runs lower than canonical H2A when unmodified but higher when ubiquitylated in denatured gel.

C. SDS-PAGE gel of purified deletion mutant proteins used in Fig 2E, S4D, E and 4F.

D. DNA binding assay of 175bp FAM labelled DNA to DNMT3A1$^{1\text{-}427}$ and cancer associated variants.

E. Pull down assay to investigate the effect of DNMT3A1 N-terminal region missense mutations in found in clinical patients on binding to H2AKc119ub nucleosomes. Equal amounts of wild type and mutant His-MBP-DNMT3A1$^{1\text{-}427}$ was immobilised on amylose beads and incubated with nucleosomes prior to washing and detection by western blot.

F. SDS-PAGE gel of deletion mutant purified proteins used in Fig 2F.

G. EMSA comparing binding of DNMT3A1$^{1\text{-}427}$ to unmodified, H2AKc119ub, H2AZ.1 and H2AZ.1Kc120ub nucleosomes wrapped with 5' FAM labelled 175bp Widom601 DNA. Limiting amounts (2.3 nM) of nucleosomes were incubated with increasing concentrations (0-8000 nM, 2x dilution series) of His-MBP- DNMT3A1$^{1\text{-}427}$. Complexes were resolved by native-PAGE and imaged using blue light excitation and 532nm emission filters. Gels show concentrations 25 nM-8000 nM, quantification was done with full concentration series of two replicates.

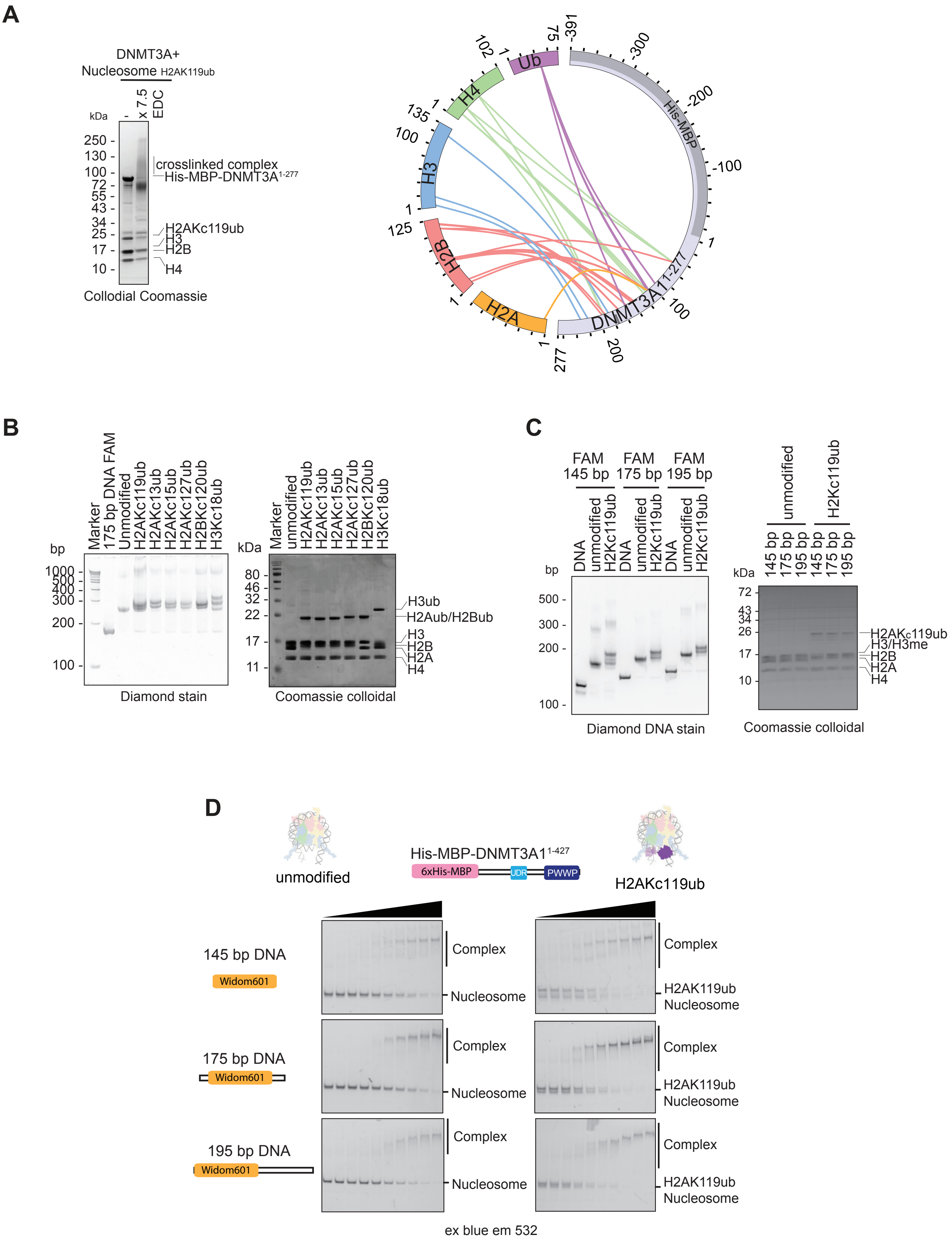
A
DNMT3A+
Nucleosome H2AK119ub
kDa
- x 7.5 EDC
250
130
100
72
55
43
34
25
17
10
crosslinked complex
His-MBP-DNMT3A1-277
H2AKc119ub
H3
H2B
H4
Collodial Coomassie
Ub
H4
H3
H2B
H2A
His-MBP
DNMT3A1-277
B
Marker
175 bp DNA FAM
Unmodified
H2AKc119ub
H2AKc13ub
H2AKc15ub
H2AKc127ub
H2BKc120ub
H3Kc18ub
bp
1000
500
400
300
200
100
Diamond stain
Marker
unmodified
H2AKc119ub
H2AKc13ub
H2AKc15ub
H2AKc127ub
H2BKc120ub
H3Kc18ub
kDa
80
46
32
22
17
11
H3ub
H2Aub/H2Bub
H3
H2B
H2A
H4
Coomassie colloidal
C
FAM 145 bp
FAM 175 bp
FAM 195 bp
DNA
unmodified
H2Kc119ub
bp
500
300
200
100
Diamond DNA stain
unmodified
H2Kc119ub
145 bp
175 bp
195 bp
kDa
72
43
34
26
17
10
H2AKc119ub
H3/H3me
H2B
H2A
H4
Coomassie colloidal
D
unmodified
His-MBP-DNMT3A11-427
6xHis-MBP
UDR
PWWP
H2AKc119ub
145 bp DNA
Widom601
175 bp DNA
Widom601
195 bp DNA
Widom601
Complex
Nucleosome
H2AK119ub
Nucleosome
ex blue em 532

Appendix Figure S5. Investigating DNMT3A1 ubiquitin site specificity

A. Crosslinking mass spectrometry $DNMT3A1^{1-277}$ with H2AKc119ub nucleosomes. (Left) SDS-PAGE gel of crosslinked complex for crosslinking mass spectrometry. Everything above non-crosslinked DNMT3A1 was excised from the gel and trypsin digested prior to identification in mass spectrometry. (right) Circular representation of cross-links detected between $DNMT3A1^{1-277}$ and H2AKc119ub nucleosomes.

B. Native gels and SDS-PAGE showing various ubiquitylated nucleosomes used in DNMT3A1 interaction assays (Fig 3B).

C. Native gels and SDS-PAGE gels of nucleosomes wrapped with different 5' FAM labelled Widom601 DNA used in S6D.

D. EMSA comparing $DNMT3A1^{1-427}$ to unmodified and H2AKc119ub nucleosomes wrapped with 5' FAM labelled 145, 175 and 195 bp Widom601 DNA. Limiting amounts (2.3 nM) of nucleosomes were incubated with increasing concentrations (0-8000 nM, 2x dilution series) of His-MBP- $DNMT3A1^{1-427}$. Complexes were resolved by native-PAGE and imaged using blue light excitation and 532nm emission filters. Gels show concentrations 7.8nM-8000 nM.

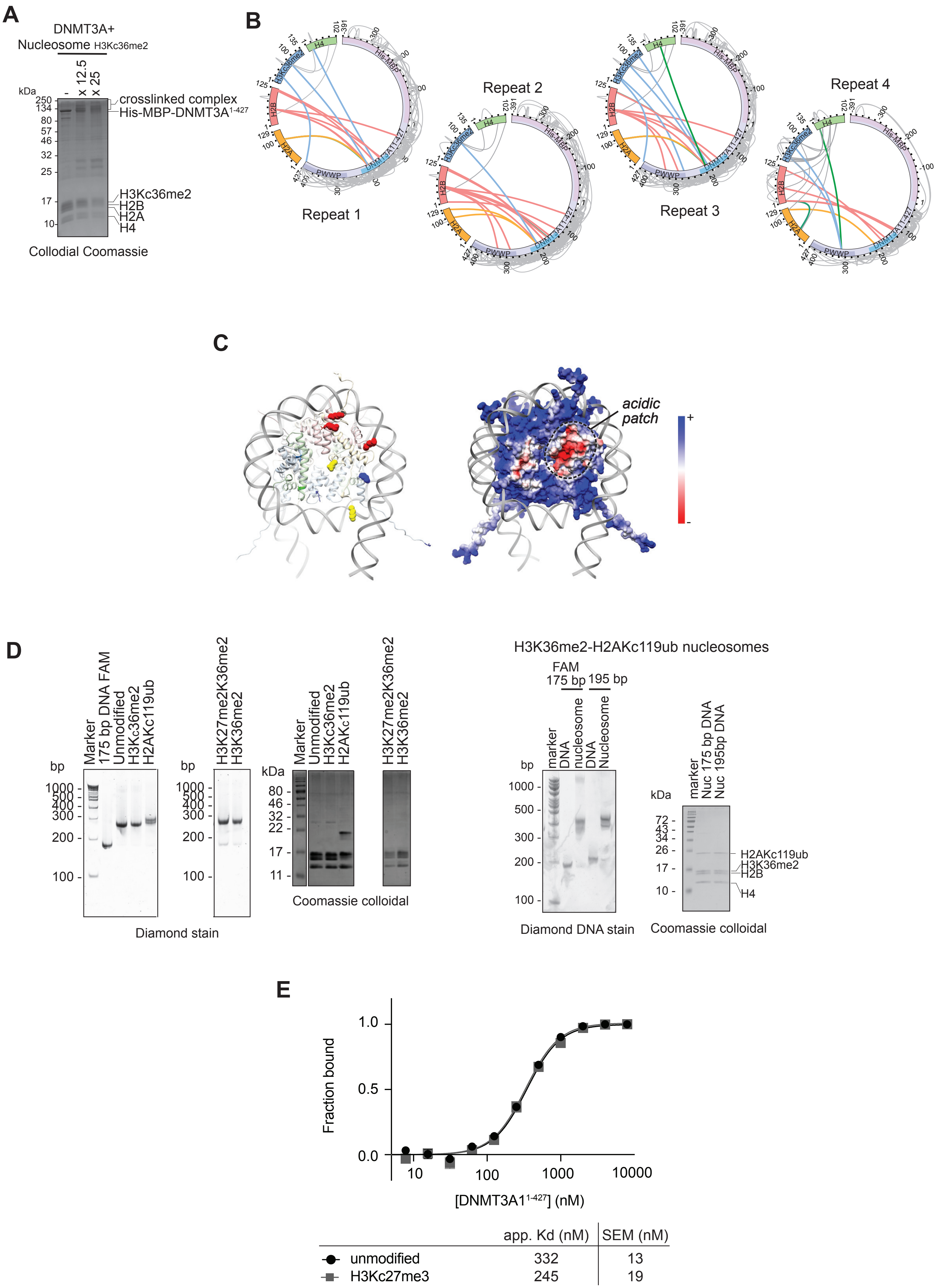
A
DNMT3A+
Nucleosome H3Kc36me2
x 12.5
x 25
kDa
crosslinked complex
His-MBP-DNMT3A1-427
H3Kc36me2
H2B
H2A
H4
Collodial Coomassie
B
Repeat 1
Repeat 2
Repeat 3
Repeat 4
H4
H3Kc36me2
H2B
H2A
PWWP
His-MBP
DNMT3A1-427
C
acidic patch
D
Marker
175 bp DNA FAM
Unmodified
H3Kc36me2
H2AKc119ub
H3K27me2K36me2
H3K36me2
bp
Diamond stain
kDa
Coomassie colloidal
H3K36me2-H2AKc119ub nucleosomes
FAM
175 bp
195 bp
marker
DNA
nucleosome
DNA
Nucleosome
Diamond DNA stain
marker
Nuc 175 bp DNA
Nuc 195bp DNA
H2AKc119ub
H3K36me2
H2B
H4
Coomassie colloidal
E
Fraction bound
[DNMT3A11-427] (nM)
app. Kd (nM)
SEM (nM)
unmodified 332 13
H3Kc27me3 245 19

Appendix Figure S6. DNMT3A can concurrently mediated interactions with acidic patch, H3K36me2 and H2AK119ub

A. SDS-PAGE gel of crosslinked complex for crosslinking mass spectrometry in Fig 4C. Everything above non-crosslinked DNMT3A1 was excised from the gel and trypsin digested prior to identification in mass spectrometry.

B. Crosslinking mass spectrometry replicates from Fig 4C. Circular representation of two biological replicates one measured in triplicate (repeat 1, 2, 3). Intra-protein crosslinks are shown in gray

C. Left: Crosslinks from Fig 4C mapped on a nucleosome model (PDB 1AOI (Luger et al., 1997)). More abundant crosslinked residues shown as spheres, less as stick representation. Model coloured, Yellow, H2A; Red, H2B; Blue, H3; Green, H4. Right: nucleosome model in same orientation as left coloured based on surface charge, to highlight the acidic patch region (dashed circle). Blue indicates positive charge; red indicates negative charge.

D. Native gels and SDS-PAGE Native gels and SDS-PAGE gels of modified and unmodified nucleosomes used in Fig 5A-C. The unmodified, H2AK119ub and DNA lanes on SDS-PAGE and Native gels are duplicated from Figure S3A.

E. Quantification of binding assay Fig 5B, performed with full concentration series in duplicate DNMT3A$^{1-427}$ to nucleosomes with or without tri-methylation at H3 Lys27 (H3K27me3).

Appendix Table S1. Cryo-EM data collection, refinement and validation statistics

Parameter	DNMT3A1-DNMT3L:Nucleosome^{H2AKc119ub}			
Data Collection				
Microscope	FEI Titan Krios			
Detector	Gatan K3			
Mode	Super-resolution			
Acceleration voltage (kV)	300			
Number of micrographs	15,491			
Frames per micrographs	56			
Exposure (s)	2.3			
Dose per frame (e-/pixel)	1.09			
Accumulated dose (e-/Å^2)	60.9			
defocus range (µm)	1.25-2.75 (0.25 step)			
Frames				
Alignment software	MotionCor2			
Frames used in final reconstruction	1-56			
Dose weighting	Yes			
CTF	**Map 1**		**Map 2-4**	
Fitting software	RELION		CryoSPARC	
Correction	full		full	
Particles				
Picking software	RELION		CryoSPARC	
Picked	1, 824, 711		7, 826, 521	
Alignment				
Alignment software	RELION		CryoSPARC	
Initial reference map	Map 2/3		Ab initio	
low pass filter limit (Å)	50		40	
Reconstruction	**Map 1**	**Map 2**	**Map 3**	**Map 4**
Reconstruction software	RELION-2	CryoSPARC	CryoSPARC	CryoSPARC
Box Size	160	384	384	384
Final Particle number	203, 347	191, 397	191, 397	198, 250
Voxel size (Å)	1.658	0.829	0.829	0.829
Symmetry	C1	C1	C1	C1
Resolution estimate (Å)	5.1	3.2	3.1	3.1
Masking	Yes (loose)	Yes (loose)	Yes (tight)	Yes (tight)
Sharpening (Å^2)	-50	-50	-109	-111
SCF*			0.94	
EMDB ID	EMD-18793		EMD-18778	
Model building				
Number of protein residues			783	
Number of DNA residues			290	
Bond length outliers			0	
Bond angle outliers			0	
Bonds (R.M.S.D)			0.004	
Angles (R.M.S.D)			0.600	
CaBLAM ouliers (%)			0.94	
Ramachandaran favoured/allowed/outlier			98.69/1.31/0.00	
Rotamer outlier (%)			0.31	
Clashscore			5.89	
Model vs Data CC (mask)			0.88	
EMringer			2.48	
Fsc model vs map 0.5			3.2	
Molprobity score			1.32	
PDB ID			8QZM	

Appendix Table S2: Apparent binding affinities for DNMT3A constructs used in this study

Nucleosome variants		DNMT3A1$^{1\text{-}427}$ App. K_d	SEM	N
unmodified	WT	440 nM	±20 nM	13
H3K36me2	WT	190 nM	±12 nM	7
H2AKc119ub	WT	141 nM	±15 nM	18
H2AKc119ub I44A	WT	208 nM	±23 nM	2
acidic patch	WT	ND	ND	2
H3Kc27me3	WT	345 nM	±19 nM	2
H3K36me2/H2AKc119ub	WT	109 nM	±2.5 nM	4
H2A.Z.1	WT	1805 nM	±361 nM	2
H2A.Z.1 Kc120ub	WT	770 nM	±63 nM	2
H2AKc13ub	WT	313 nM	±30 nM	3
H2AKc15ub	WT	442 nM	±51 nM	3
H2AKc127ub	WT	443 nM	±88 nM	3
H2BKc120ub	WT	495 nM	±51 nM	3
H3Kc18ub	WT	294 nM	±28 nM	3
unmodified	D333N	329 nM	±29 nM	2
H3K36me2	D333N	448 nM	±52 nM	2
unmodified	W330R	482 nM	±42 nM	2
H3K36me2	W330R	467 nM	±62 nM	2
H2AKc119ub	R181C	1235 nM	±182 nM	2
H2AKc119ub	R183W	126 nM	±9 nM	2
H2AKc119ub	A192E	714 nM	±95 nM	2
unmodified	Δ142-178	992 nM	±68 nM	2
H2AKc119ub	Δ142-178	422 nM	±19 nM	2
unmodified	Δ165-174	726 nM	±89 nM	2
H2AKc119ub	Δ165-174	317 nM	±21 nM	2
unmodified	Δ184-191	1103 nM	±109 nM	2
H2AKc119ub	Δ184-191	680 nM	±34 nM	2

		DNMT3A1$^{1\text{-}277}$ App. K_d	SEM	N
unmodified	WT	745 nM		1
H3K36me2	WT	982 nM		1
H2AK119ub	WT	164 nM		1
acidic patch	WT	ND		1

EMSAs were quantified by disappearance of the FAM-labelled nucleosome band using BioRad Image Lab 6.1. Nonlinear regression – binding saturation was fitted in GraphPad Prism 10. Apparent Kd values were averaged over all EMSAs performed in this manuscript and standard error of mean (SEM) values were calculated, N = nr of individual experiments performed for the binding interaction. ND = not determined, binding did not reach completion at the concentrations tested.

Appendix Table S3. Expression constructs used in this study.

Construct	Protein (aa)	Domain/regions(s)	Mutations
His-MBP			-
**DNMT3A1**			
His-MBP-DNMT3A1-427	1-427	NT UDR PWWP	-
His-MBP-DNMT3A1-277	1-277	NT UDR	-
His-MBP-DNMT3A1-427 R181C	1-427	NT UDR PWWP	R181C
His-MBP-DNMT3A1-427 R183W	1-427	NT UDR PWWP	R183W
His-MBP-DNMT3A1-427 A192E	1-427	NT UDR PWWP	A192E
His-MBP-DNMT3A1-427 W330R	1-427	NT UDR PWWP	W330R
His-MBP-DNMT3A1-427 D333N	1-427	NT UDR PWWP	D333N
His-MBP-DNMT3A1-427 P195A	1-427	NT UDR PWWP	P195A
His-MBP-DNMT3A1-427 Y197A	1-427	NT UDR PWWP	Y197A
His-MBP-DNMT3A1-427 I198A	1-427	NT UDR PWWP	I198A
His-MBP-DNMT3A1-427 K200A	1-427	NT UDR PWWP	K200A
His-MBP-DNMT3A1-427 R201A	1-427	NT UDR PWWP	R201A
His-MBP-DNMT3A1-427 K202A	1-427	NT UDR PWWP	K202A
His-MBP-DNMT3A1-427 R203A	1-427	NT UDR PWWP	R203A
His-MBP-DNMT3A1-427 D204A	1-427	NT UDR PWWP	D204A
His-MBP-DNMT3A1-427 E205A	1-427	NT UDR PWWP	E205A
His-MBP-DNMT3A1-427 L207A	1-427	NT UDR PWWP	L207A
His-MBP-DNMT3A58-427	58-427	NT UDR PWWP	-
His-MBP-DNMT3A115-427	115-427	NT UDR PWWP	-
His-MBP-DNMT3A142-427	142-427	NT UDR PWWP	-
His-MBP-DNMT3A192-427	192-427	NT PWWP	-
His-MBP-DNMTA164-219	164-219	UDR	-
His-MBP-DNMT3A1-427 Δ142-178	1-427	NT PWWP	Δ142-178
His-MBP-DNMT3A1-427 Δ165-174	1-427	NT PWWP	Δ142-174
His-MBP-DNMT3A1-427 Δ184-191	1-427	NT PWWP	Δ142-191
His-MBP-DNMT3A1	1-912	NT PWWP ADD Mtase	-
**DNMT3A2**			
His-MBP-DNMT3A2	1-689	PWWP ADD Mtase	-
**DNMT3L**			
His-GFP-DNMT3L-StrepII	1-386	ADD Mtase-like<br>-	
**DNMT3B3**			
**His-GFP-DNMT3B3 534-770-StrepII**	**534-770**	Mtase-like	
**Histones**			

unmodified H3.1 (No cys)	1-136	C110A, C96S
H3.3 T45CΔ1-44	44-136	T45CΔ1-44, C110A
H3.1 K36C	1-136	C110A, C96S, K36C
H3.1 K27C	1-136	C110A, C96S, K27C
H2A	1-130	-
H2A acidic patch	1-130	E61A E91A E92A
H2B	1-126	-
H2B acidic patch	1-126	E105A
H4	1-103	-
H2A K13C	1-130	K13C
H2A K15C	1-130	K15C
H2A K119C	1-130	K119C
H1A K127C	1-130	K127C
H2B K120C	1-126	K120C
H3 K18C	1-136	C110A, C96S, K18C
H2AK119C acidic patch	1-130	E61A E91A E92A K119C
H3tailless	25-135	C110A, C96S, A25C Δ1-24
H3K27CK36C	1-136	C110A, C96S, K27C/K36C
**Ubiquitin**		
His-TEV-Ubiquitin	1-76	G76C
His-TEV-Ubiquitin I44A	1-76	I44A/G76C

Appendix Table S4. DNA sequences used for wrapping nucleosomes.

Name	Length	Sequence
145bp DNA Widom 601	145	TGGAGAATCCCGGTGCCGAGGCCGCTCAATTGGTCGTAGACAGCTCTAGCACCGCTTAAACGCACGTACGCGCTGTCC CCCGCGTTTTAACCGCCAAGGGGATTACTCCCTAGTCTCCAGGCACGTGTCAGATATATACATCCTG
175bp DNA Widom 601	175	AATAGCCACCTGCCCTGGAGAATCCCGGTGCCGAGGCCGCTCAATTGGTCGTAGACAGCTCTAGCACCGCTTAAACGCA CGTACGCGCTGTCCCCCGCGTTTTAACCGCCAAGGGGATTACTCCCTAGTCTCCAGGCACGTGTCAGATATATACATCCTG TGCAATGTGTTCCAT
195 bp DNA Widom 601	195	TGGAGAATCCCGGTGCCGAGGCCGCTCAATTGGTCGTAGACAGCTCTAGCACCGCTTAAACGCACGTACGCGCTGTCCCC CGCGTTTTAACCGCCAAGGGGATTACTCCCTAGTCTCCAGGCACGTGTCAGATATATACATCCTGTCACCATACGCCCTAAT TAGAGGCGTAATCCCCCAGTTCGCGCGCCCACC
193 bp DNA Widom 603	193	GTGAGCCGTAAAATAATCGACACTCTCGGGTGCCCAGTTCGCGCGCCCACCTACCGTGTGAAGTCGTCACTCGGGCTTCTA AGTACGCTTAGCGCACGGTAGAGCGCAATCCAAGGCTAACCACCGTGCATCGATGTTGAAAGAGGCCCTCCGTCCTTATTAC TTCAAGTCCCTGGGGTACCCGTTTCGGTGC
207bp Widom 601	207	CGCTCTAGACCATGATGCCGGATCCCCTGGAGAATCCCGGTGCCGAGGCCGCTCAATTGGTCGTAGACAGCTCTAGCACCG CTTAAACGCACGTACGCGCTGTCCCCCGCGTTTTAACCGCCAAGGGGATTACTCCCTAGTCTCCAGGCACGTGTCACATATA TACATCCTGTCCCAGTGCCGGTGTCGCTTGGGTCACGAGGTGAAGC

Appendix Table S5. Read alignment statistics from Nanopore sequencing of in vitro methyltransferase assays.

Sample	Total reads	Aligned reads
DNA alone – rep 1	16717	16257
Unmodified – rep1	22521	22023
Unmodified – rep 2	23282	22748
Acidic patch – rep 1	22686	22120
H3K36me2 – rep 1	20923	20426
H3K36me2 – rep 2	22696	22115
H2AKc119ub – rep 1	20983	20399
H2AKc119ub – rep 2	19633	19212
H3K36me2/H2AKc119ub – rep 1	18713	18215
H3K36me2/H2AKc119ub – rep 2	21523	21080

LUGER, K., MADER, A. W., RICHMOND, R. K., SARGENT, D. F. & RICHMOND, T. J. 1997. Crystal structure of the nucleosome core particle at 2.8 A resolution. *Nature,* 389**,** 251-60.
