# Supplementary figures and images for: The N-terminal region of DNMT3A engages the nucleosome surface to aid chromatin recruitment
Source: EMBO Rep. 2024 Nov 11;25(12):5743–79. doi: 10.1038/s44319-024-00306-3 (PMC11624362; doi:10.1038/s44319-024-00306-3)

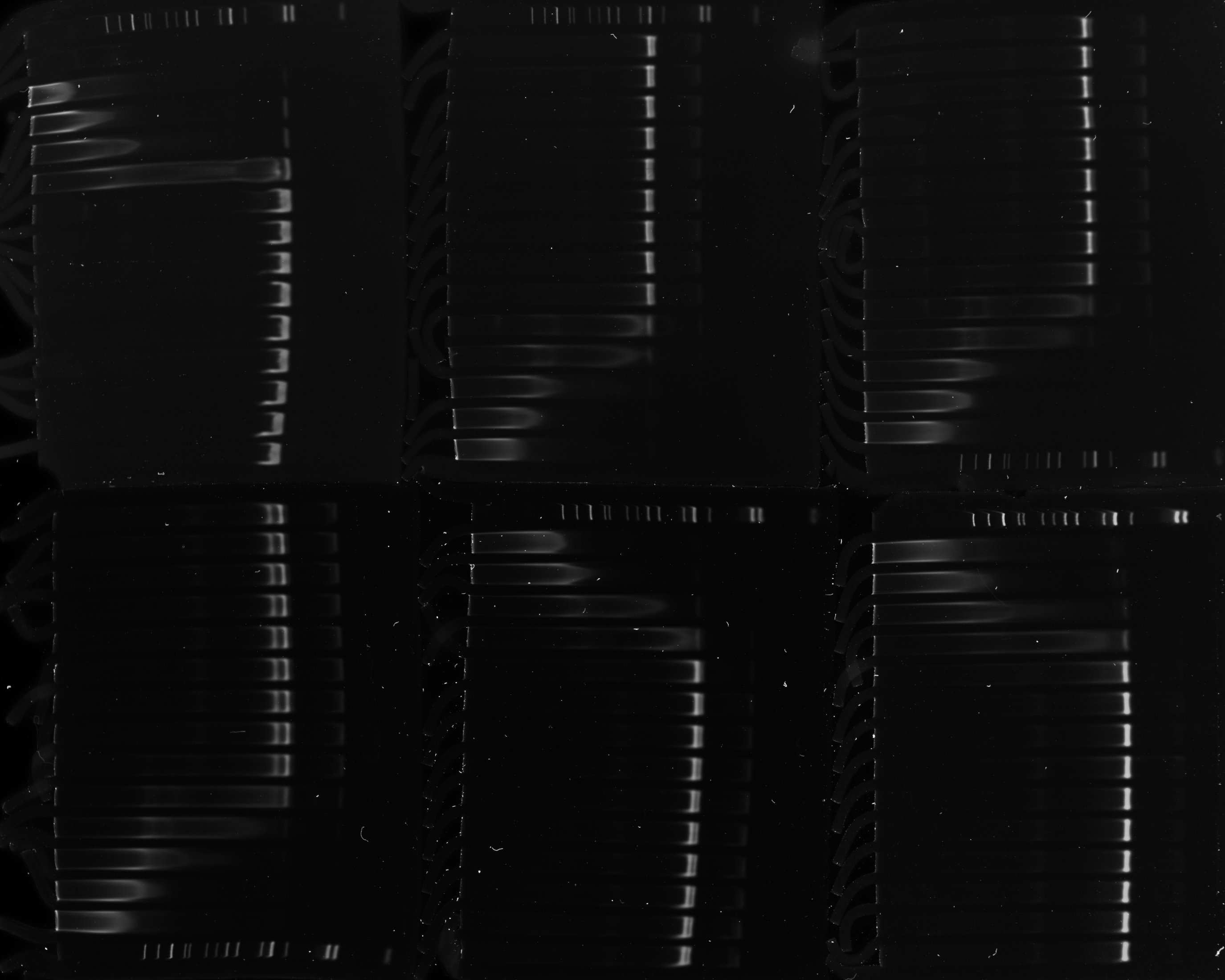

Supplement: Supplementary file 3 — Source data Fig. 1 [file 44319_2024_306_MOESM3_ESM.zip › EMBOR-2024-60481V2_SourceDataForFigure 1/Figure 1B/Figure1B repeat 2.tif]

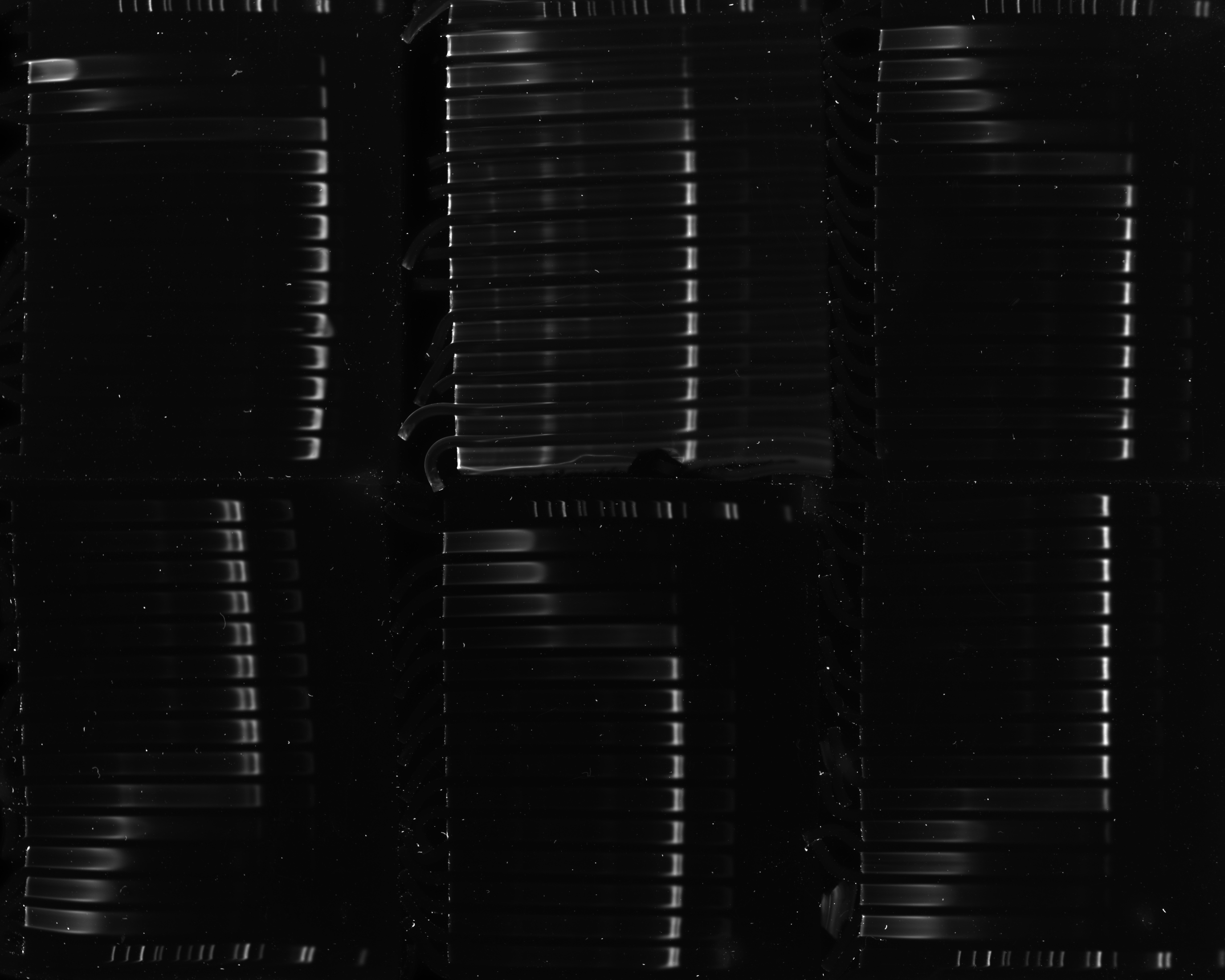

Supplement: Supplementary file 3 — Source data Fig. 1 [file 44319_2024_306_MOESM3_ESM.zip › EMBOR-2024-60481V2_SourceDataForFigure 1/Figure 1B/Figure1B repeat 1.tif]

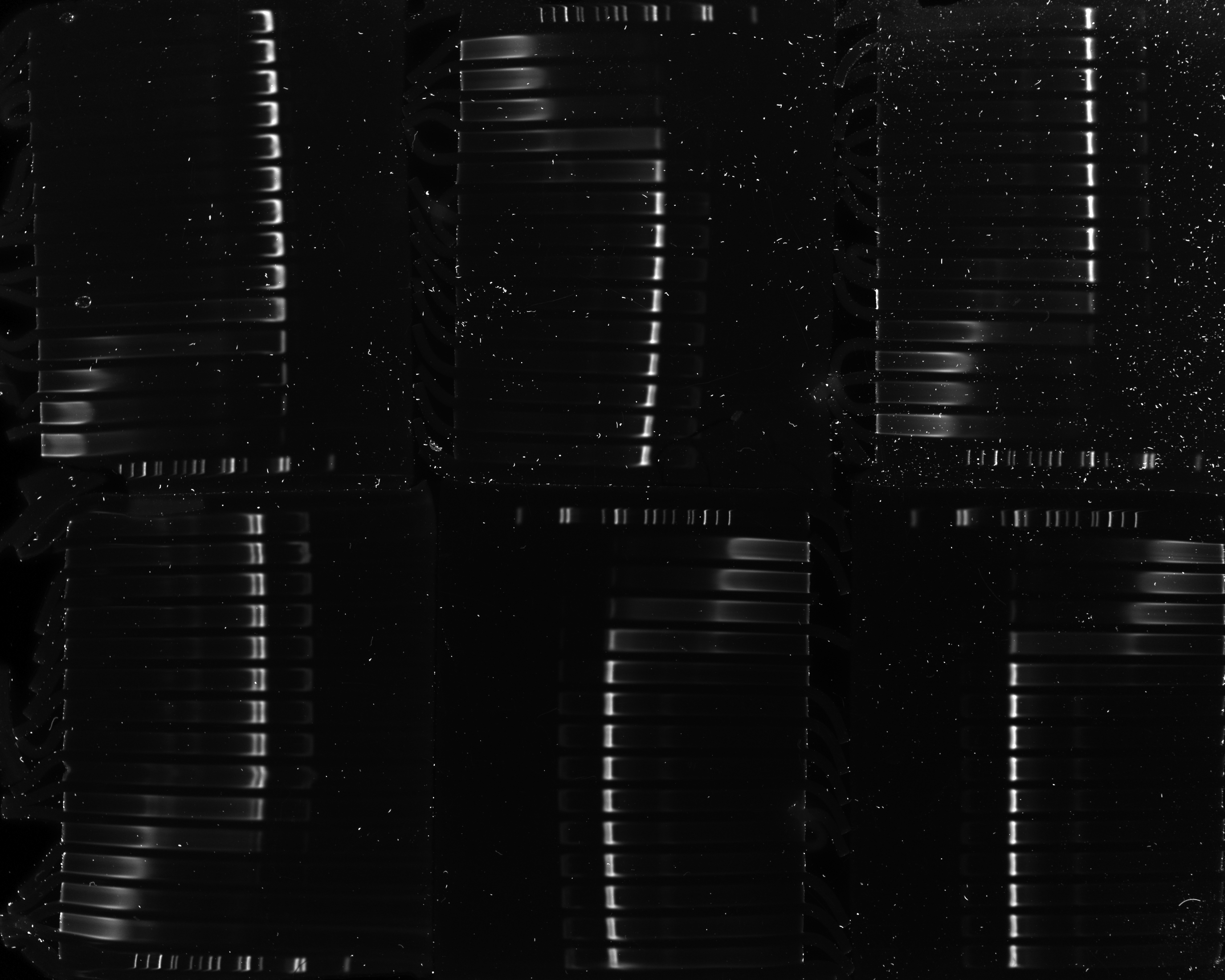

Supplement: Supplementary file 3 — Source data Fig. 1 [file 44319_2024_306_MOESM3_ESM.zip › EMBOR-2024-60481V2_SourceDataForFigure 1/Figure 1B/Figure1B.tif]

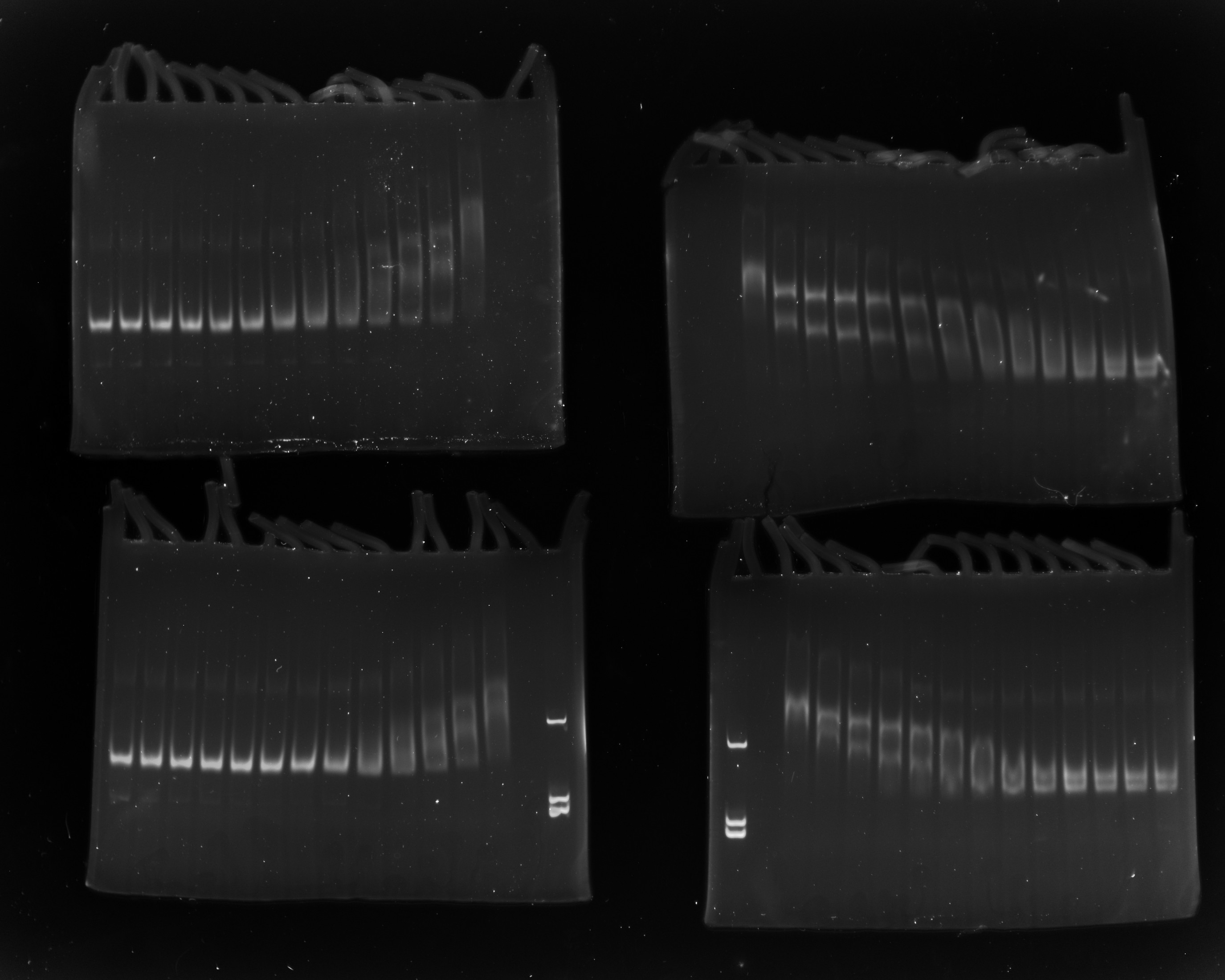

Supplement: Supplementary file 3 — Source data Fig. 1 [file 44319_2024_306_MOESM3_ESM.zip › EMBOR-2024-60481V2_SourceDataForFigure 1/Figure 1E/Figure 1E repeat 2.tif]

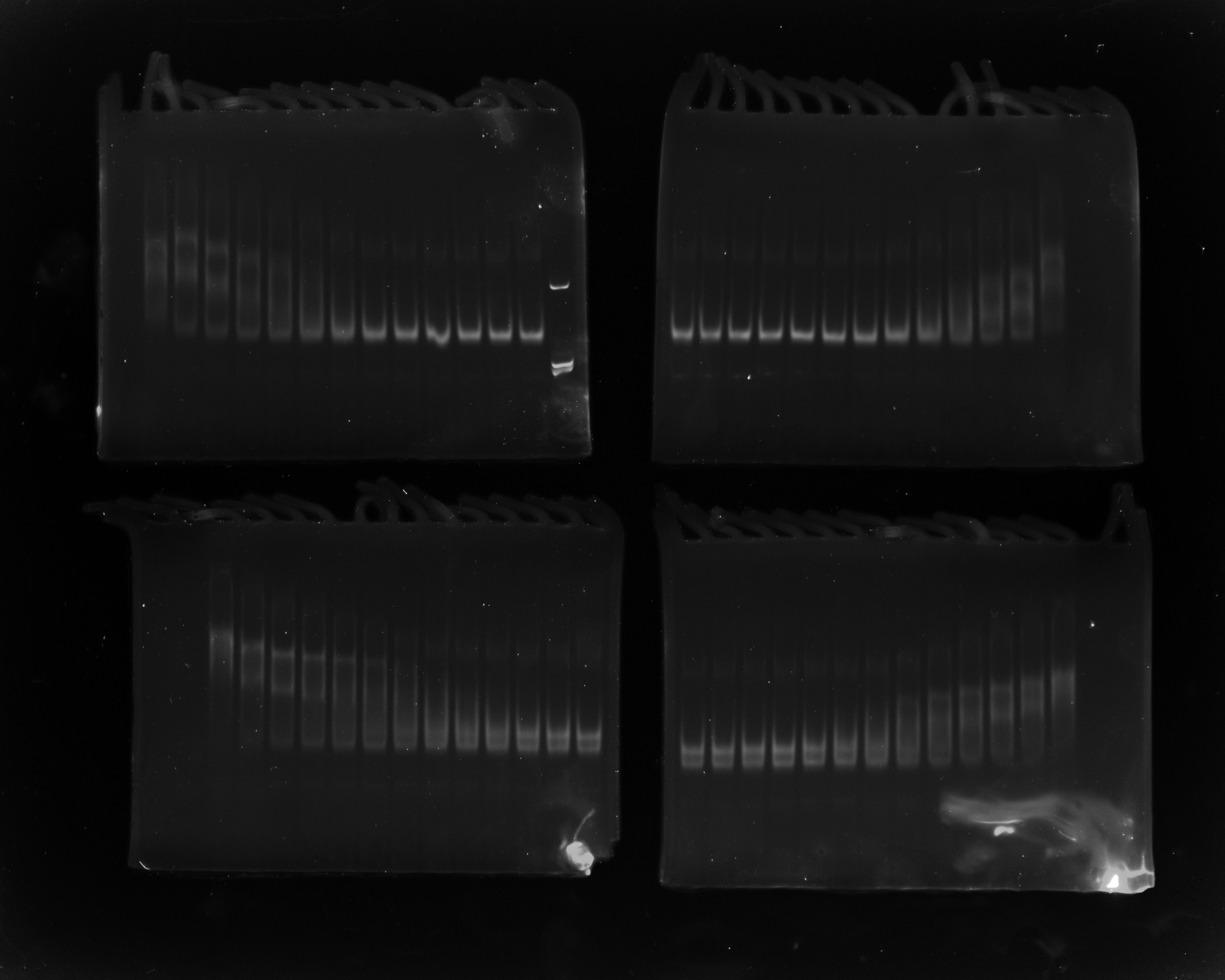

Supplement: Supplementary file 3 — Source data Fig. 1 [file 44319_2024_306_MOESM3_ESM.zip › EMBOR-2024-60481V2_SourceDataForFigure 1/Figure 1E/Figure 1E repeat 1.tif]

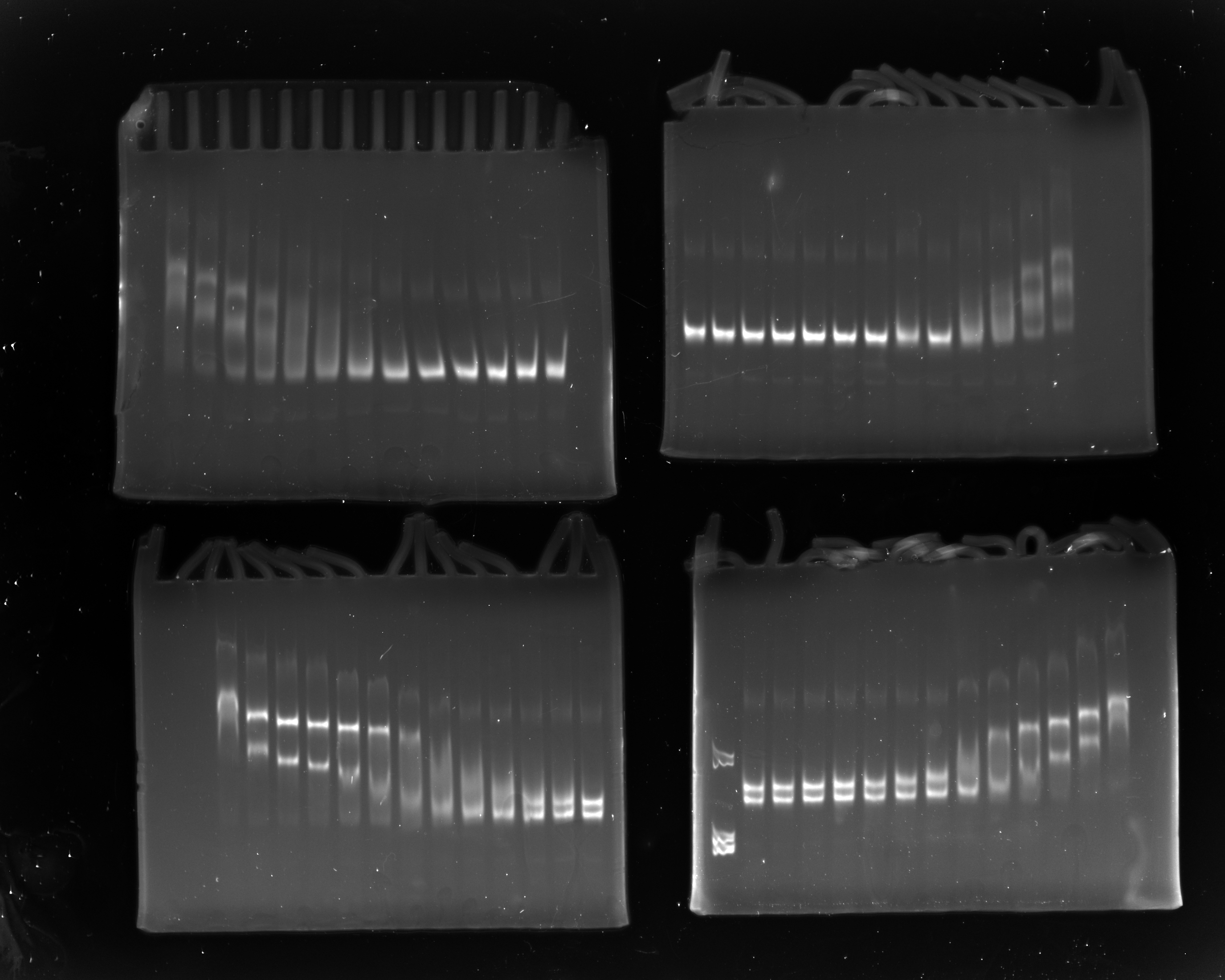

Supplement: Supplementary file 3 — Source data Fig. 1 [file 44319_2024_306_MOESM3_ESM.zip › EMBOR-2024-60481V2_SourceDataForFigure 1/Figure 1E/Figure1E.tif]

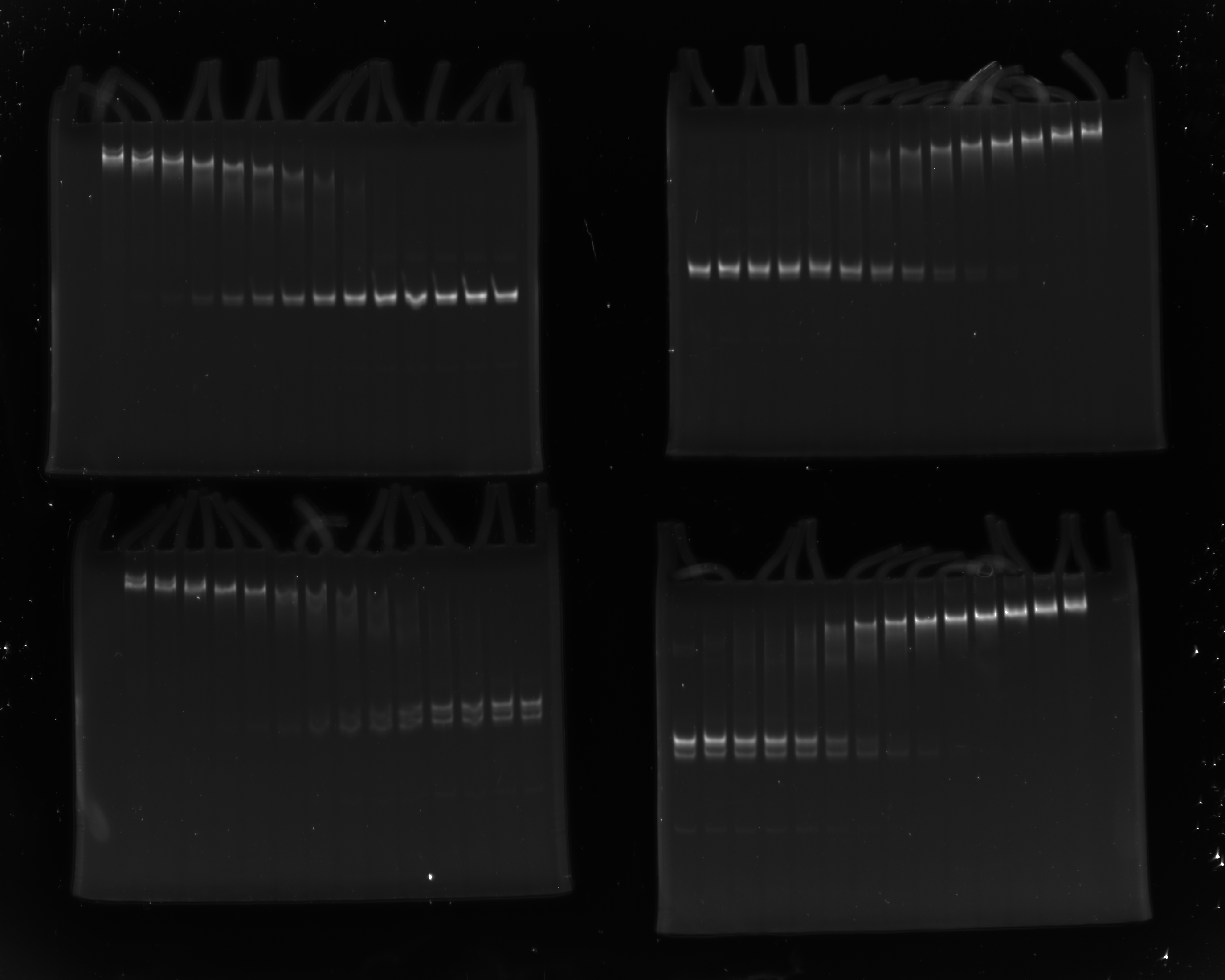

Supplement: Supplementary file 3 — Source data Fig. 1 [file 44319_2024_306_MOESM3_ESM.zip › EMBOR-2024-60481V2_SourceDataForFigure 1/Figure 1D/Figure 1D repeat 3.tif]

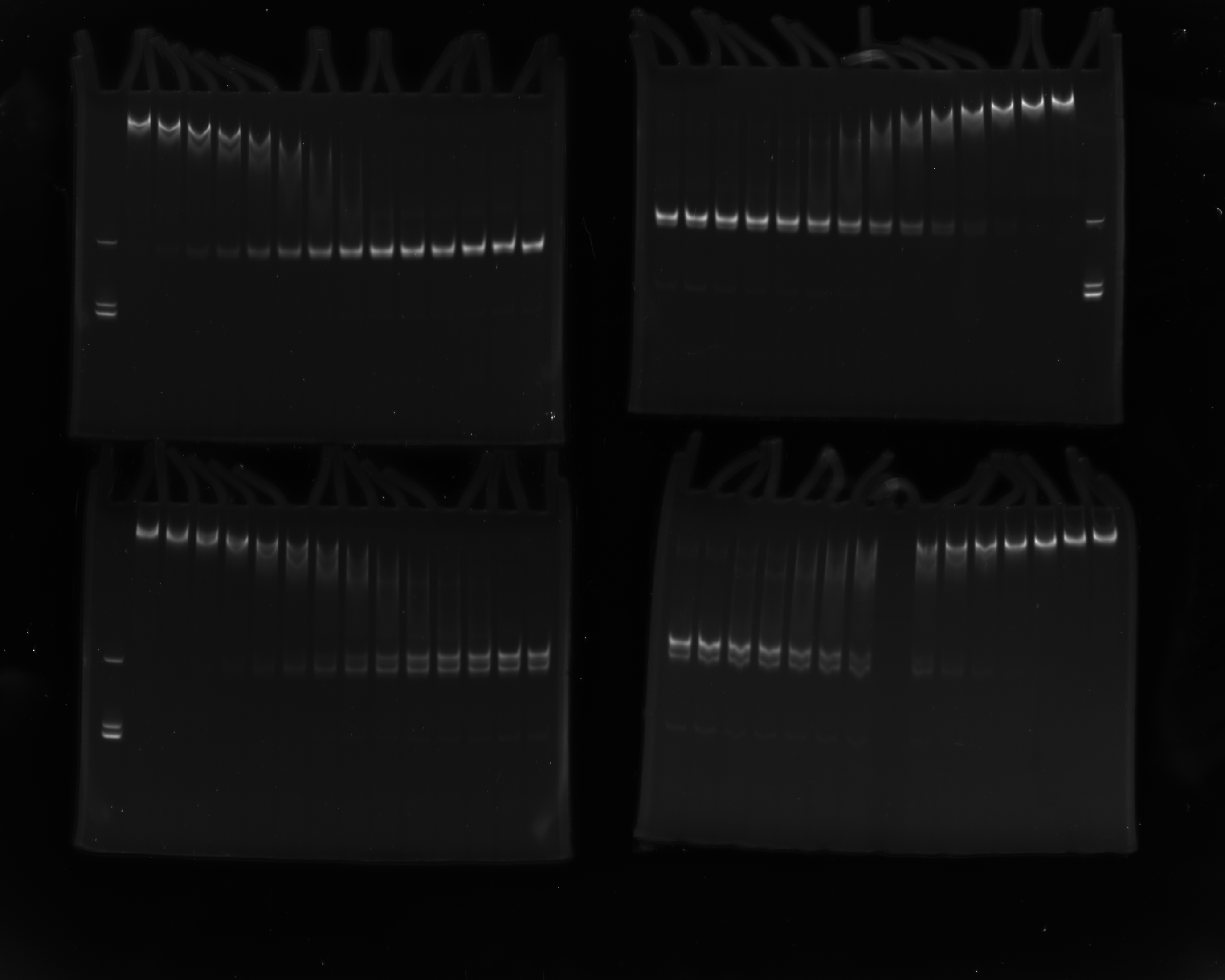

Supplement: Supplementary file 3 — Source data Fig. 1 [file 44319_2024_306_MOESM3_ESM.zip › EMBOR-2024-60481V2_SourceDataForFigure 1/Figure 1D/Figure 1D repeat 2.tif]

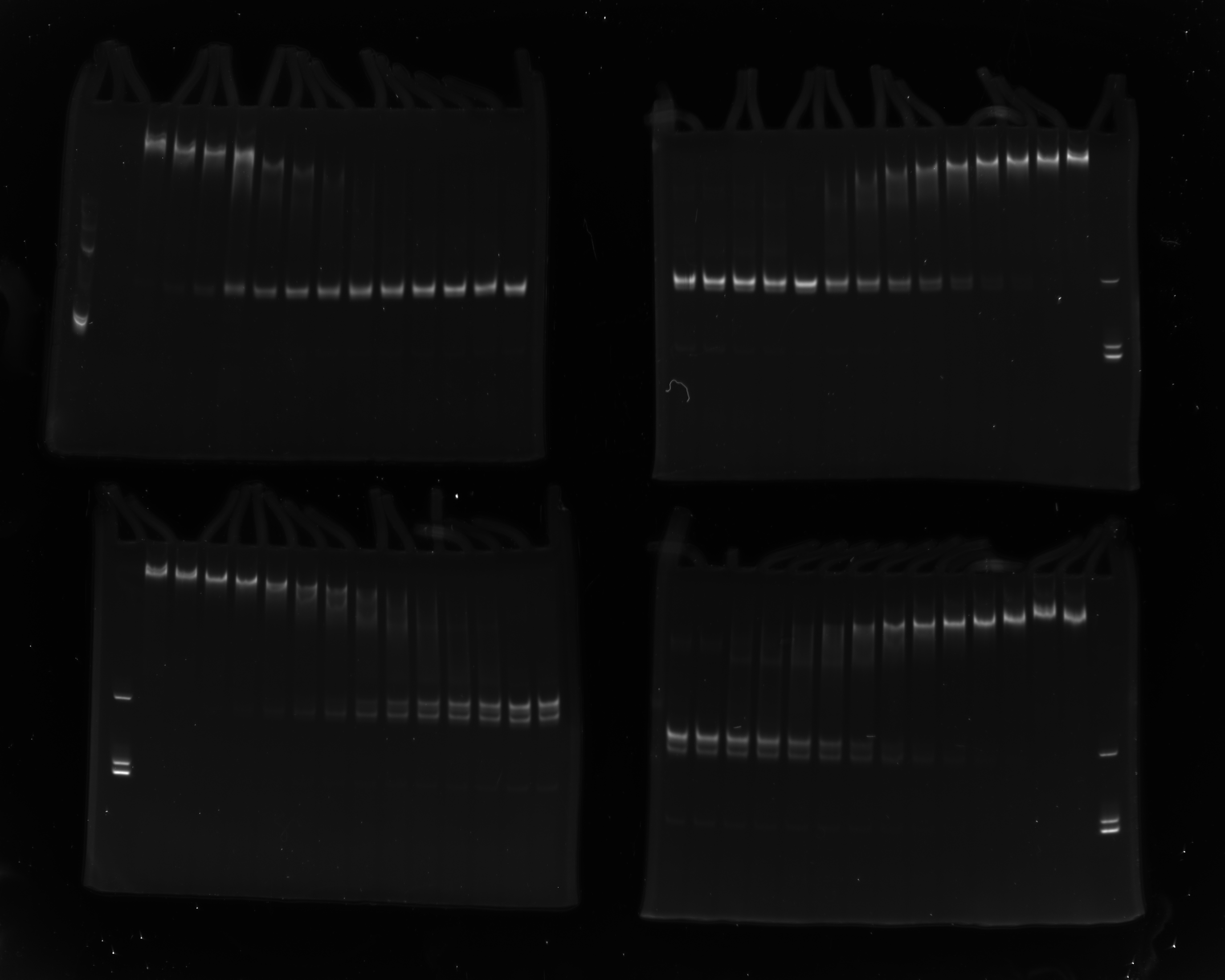

Supplement: Supplementary file 3 — Source data Fig. 1 [file 44319_2024_306_MOESM3_ESM.zip › EMBOR-2024-60481V2_SourceDataForFigure 1/Figure 1D/Figure 1D repeat1.tif]

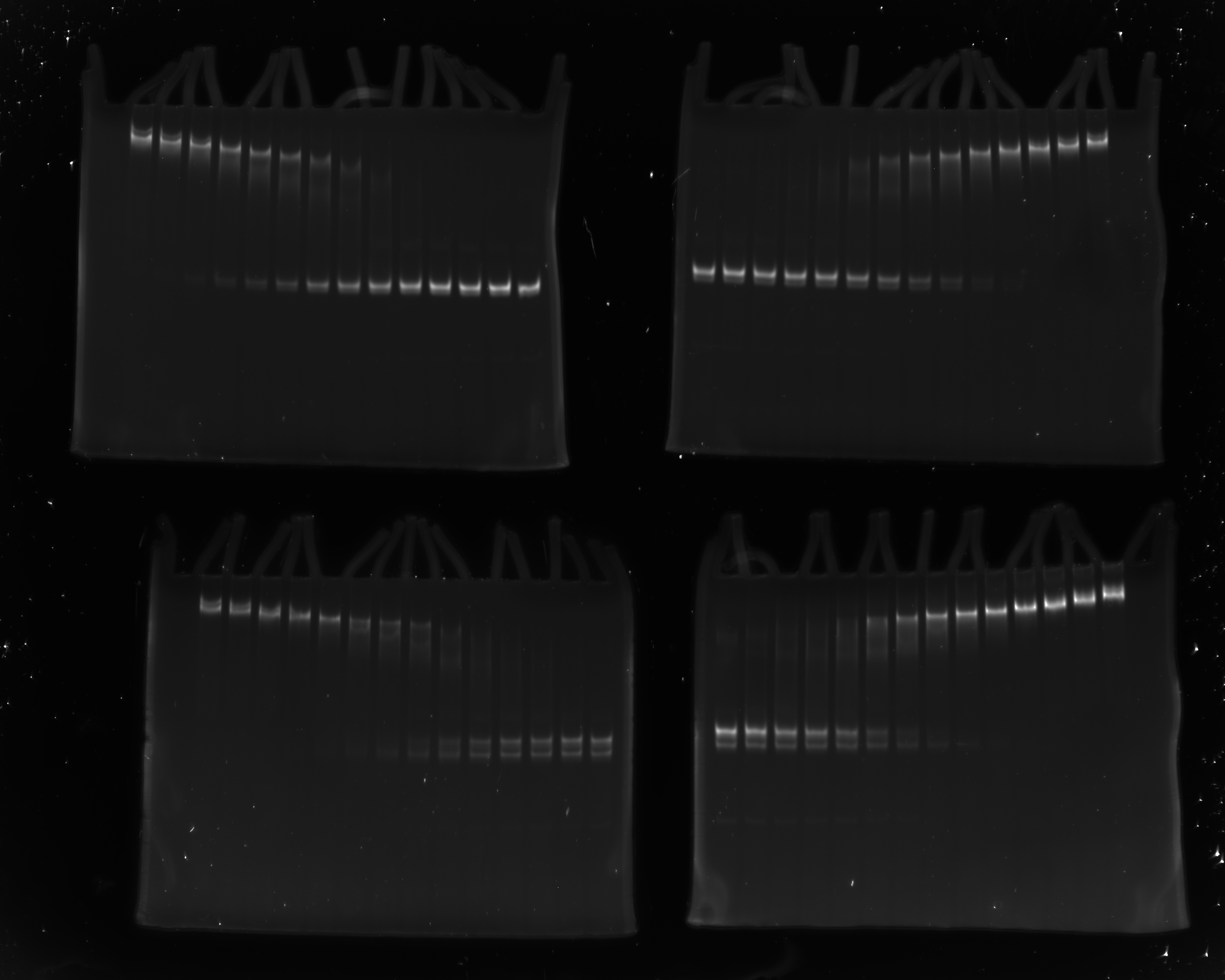

Supplement: Supplementary file 3 — Source data Fig. 1 [file 44319_2024_306_MOESM3_ESM.zip › EMBOR-2024-60481V2_SourceDataForFigure 1/Figure 1D/Figure 1D.tif]

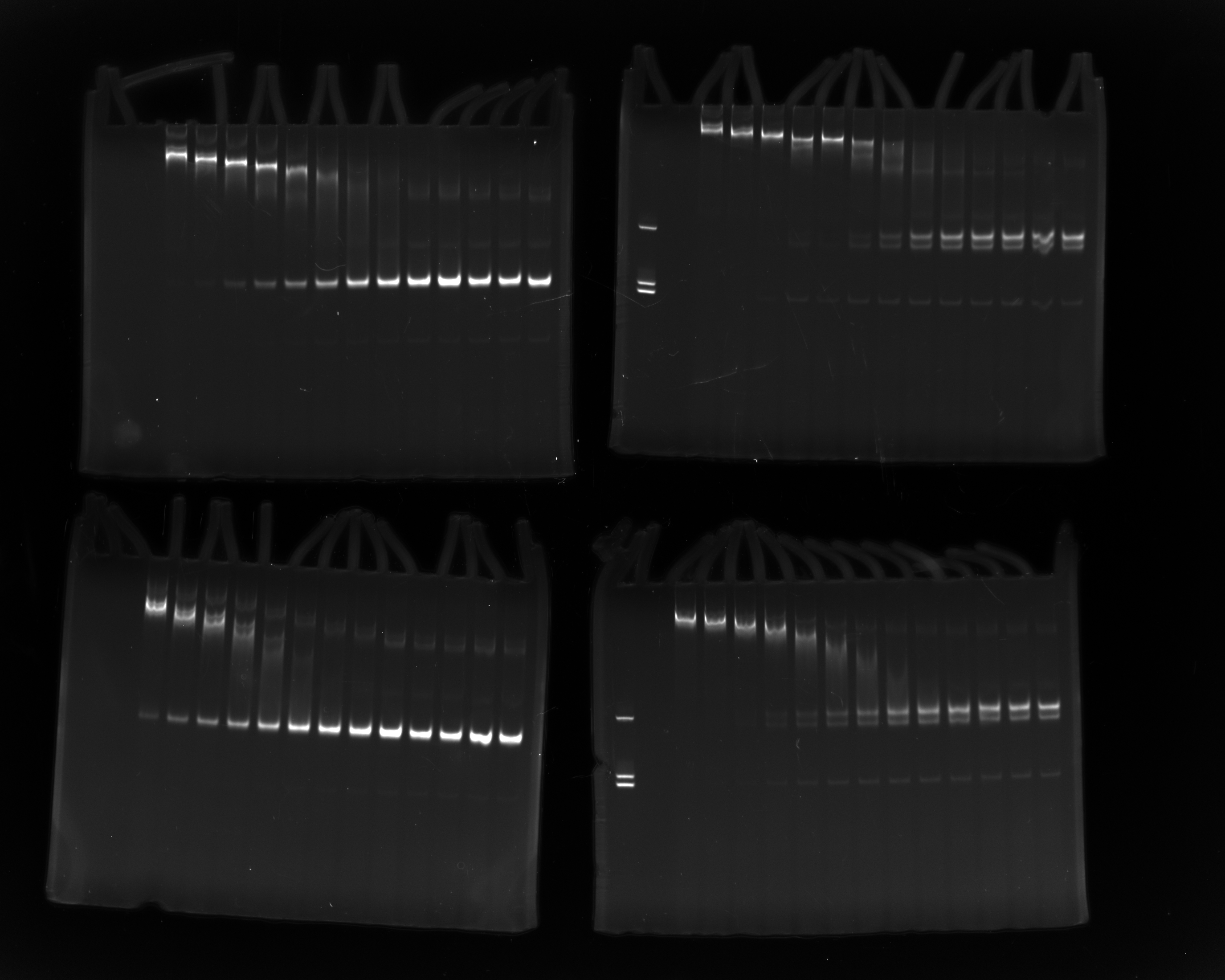

Supplement: Supplementary file 4 — Source data Fig. 2 [file 44319_2024_306_MOESM4_ESM.zip › EMBOR-2024-60481V2_SourceDataForFigure 2/Figure 2C/Figure 2F/Figure 2F 165.tif]

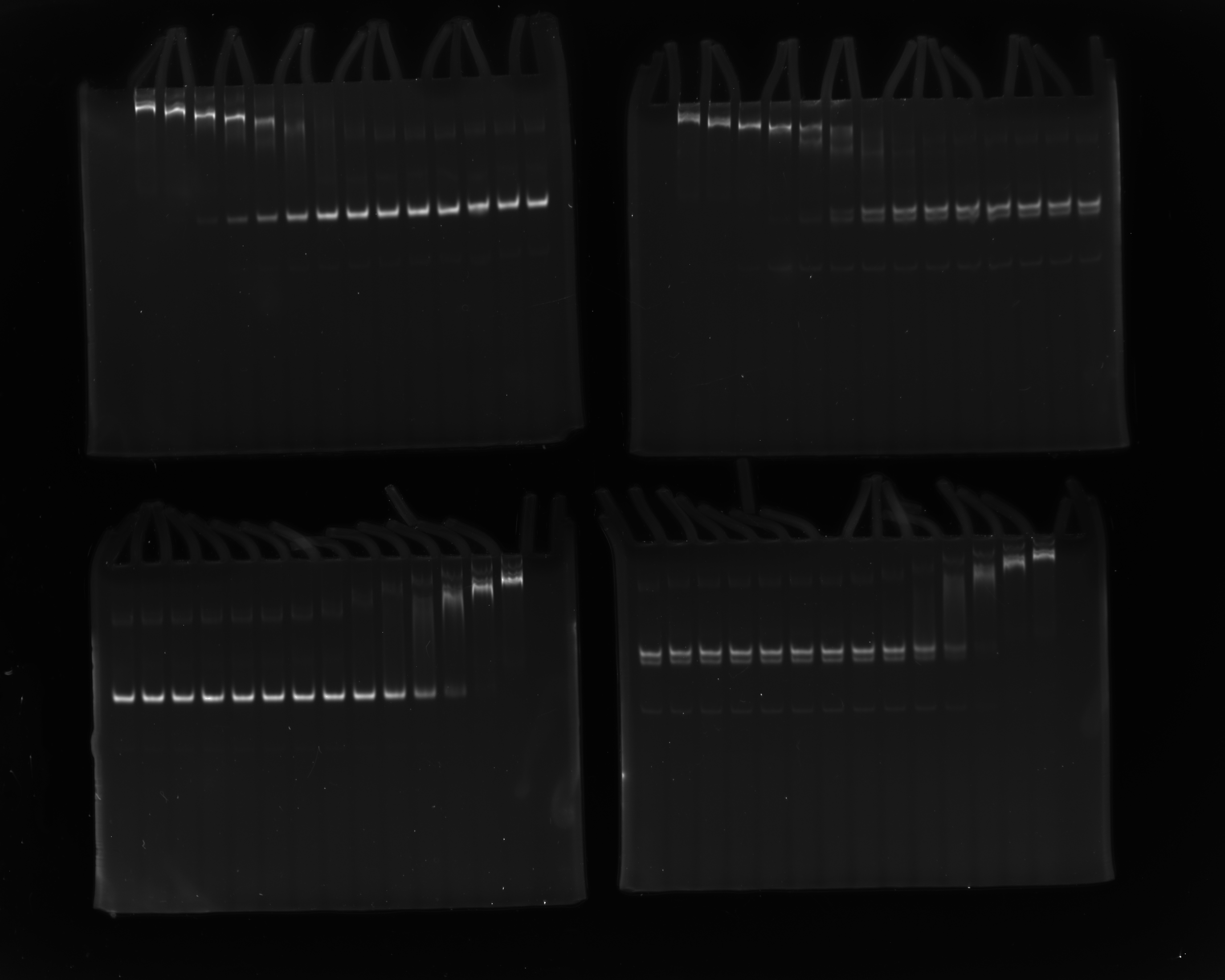

Supplement: Supplementary file 4 — Source data Fig. 2 [file 44319_2024_306_MOESM4_ESM.zip › EMBOR-2024-60481V2_SourceDataForFigure 2/Figure 2C/Figure 2F/Figure 2F repeat 1 WT, 184-191.tif]

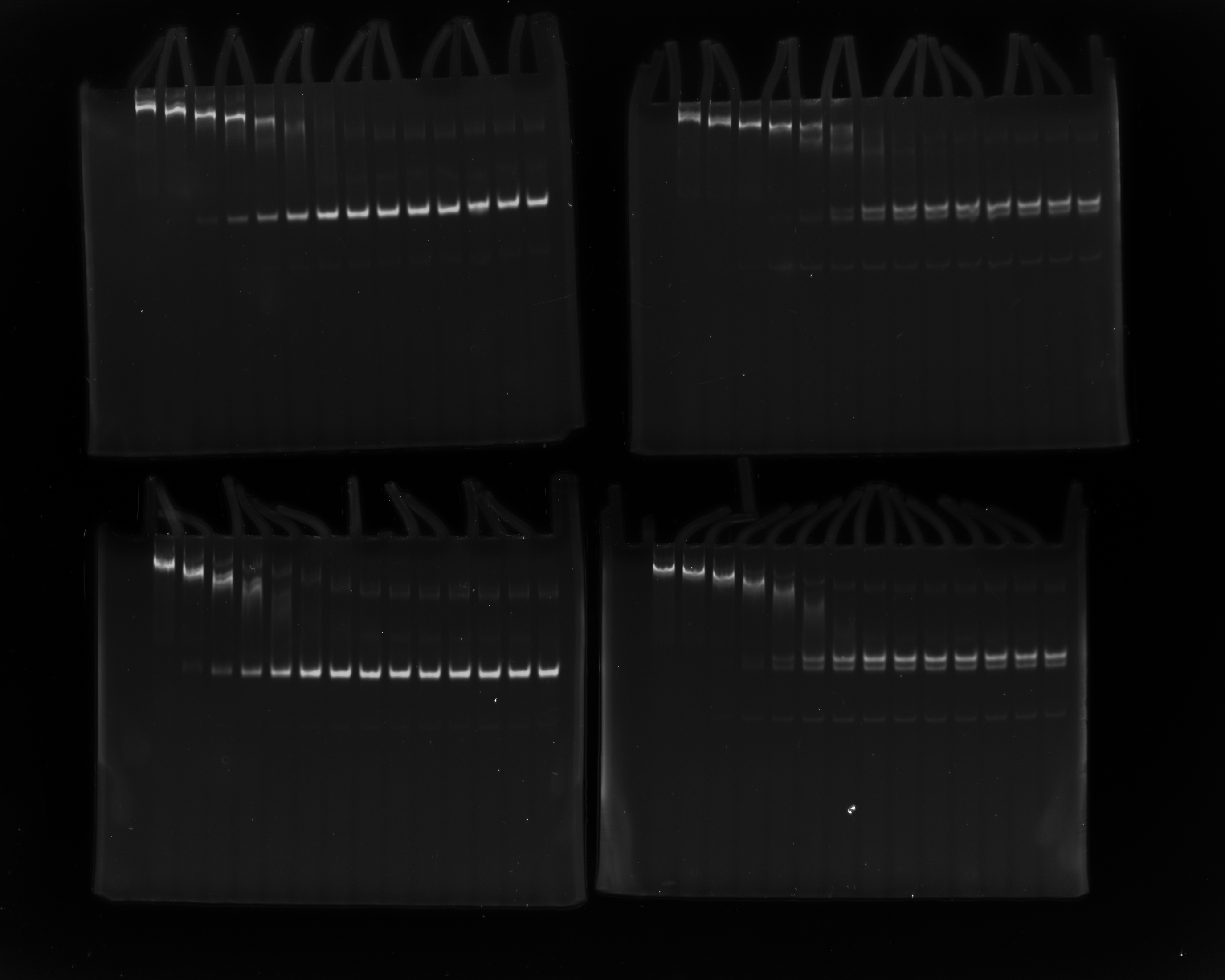

Supplement: Supplementary file 4 — Source data Fig. 2 [file 44319_2024_306_MOESM4_ESM.zip › EMBOR-2024-60481V2_SourceDataForFigure 2/Figure 2C/Figure 2F/Figure 2F repeat 1 WT, 165-174 shorter.tif]

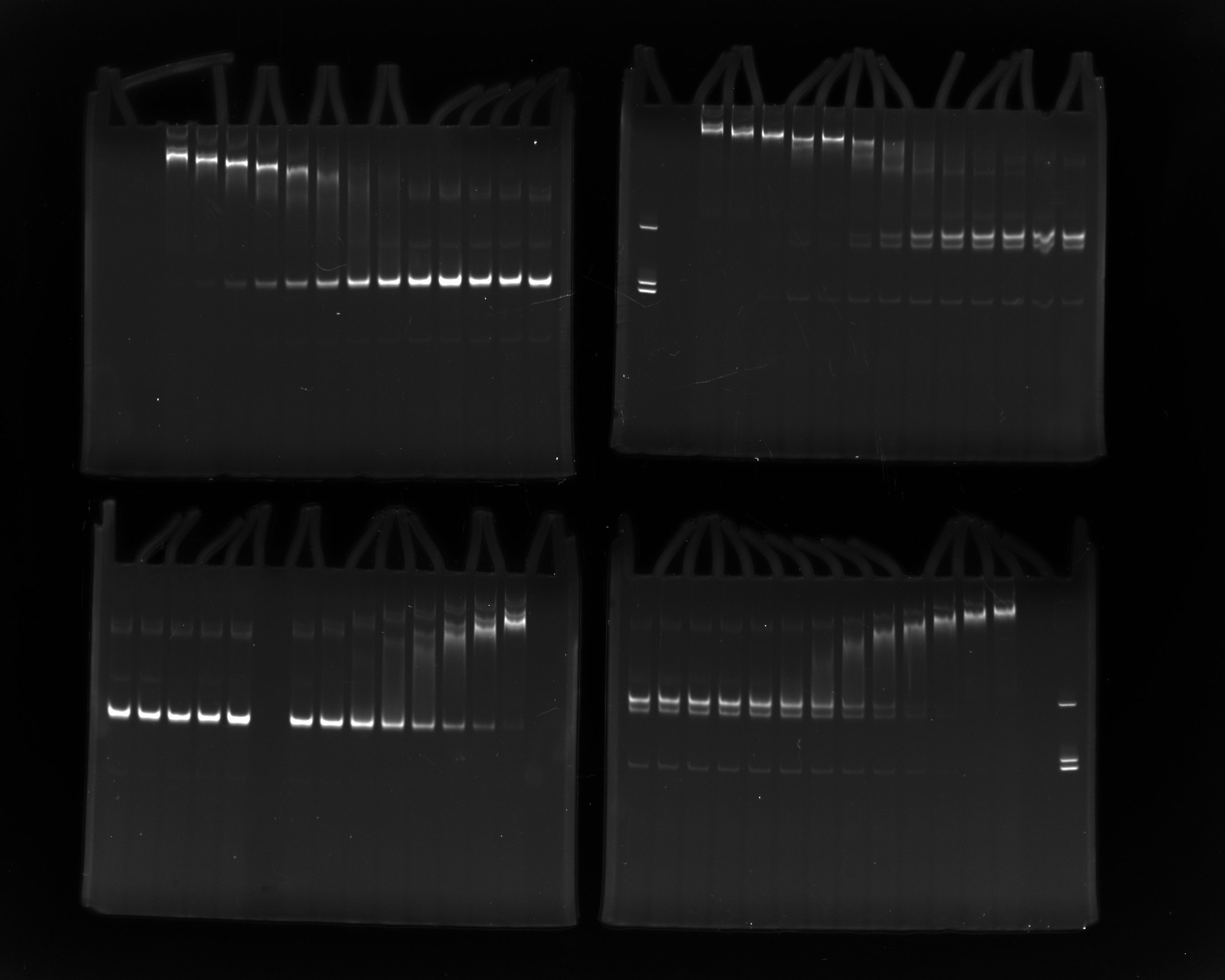

Supplement: Supplementary file 4 — Source data Fig. 2 [file 44319_2024_306_MOESM4_ESM.zip › EMBOR-2024-60481V2_SourceDataForFigure 2/Figure 2C/Figure 2F/Figure 2F WT 142.tif]

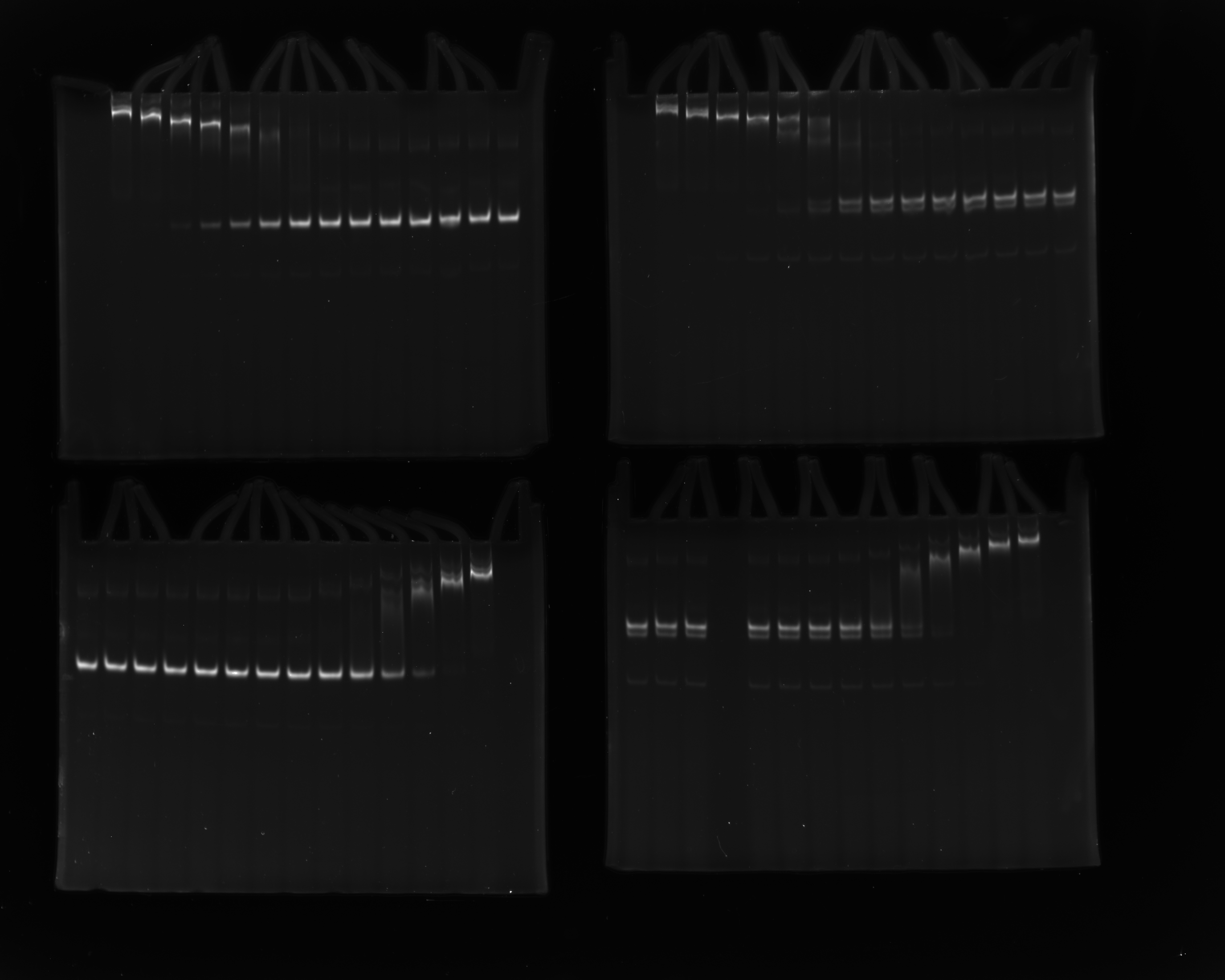

Supplement: Supplementary file 4 — Source data Fig. 2 [file 44319_2024_306_MOESM4_ESM.zip › EMBOR-2024-60481V2_SourceDataForFigure 2/Figure 2C/Figure 2F/Figure 2F repeat 1 WT, 142-178.tif]

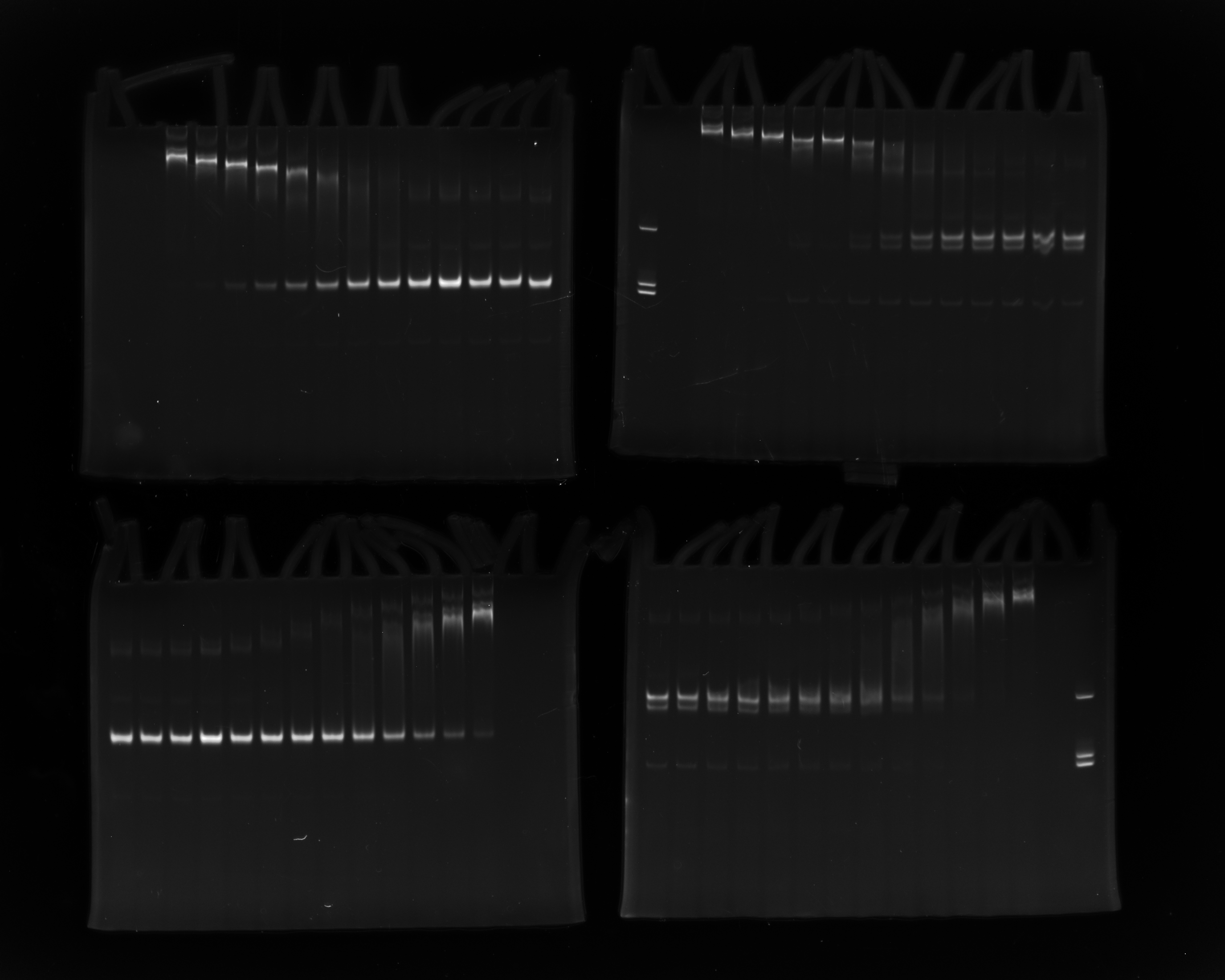

Supplement: Supplementary file 4 — Source data Fig. 2 [file 44319_2024_306_MOESM4_ESM.zip › EMBOR-2024-60481V2_SourceDataForFigure 2/Figure 2C/Figure 2F/Figure 2F 184.tif]

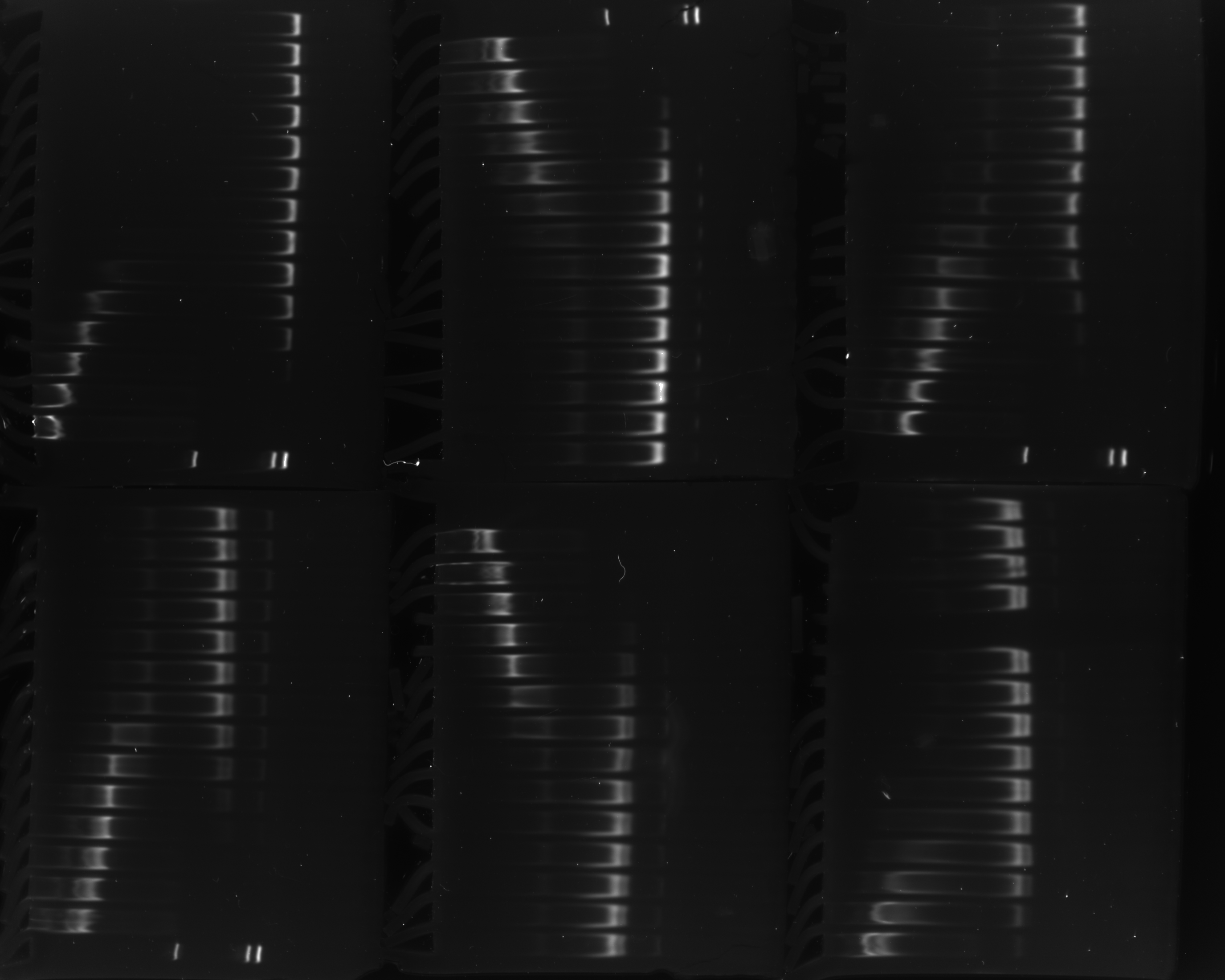

Supplement: Supplementary file 4 — Source data Fig. 2 [file 44319_2024_306_MOESM4_ESM.zip › EMBOR-2024-60481V2_SourceDataForFigure 2/Figure 2C/Figure 2C/Figure 2C.tif]

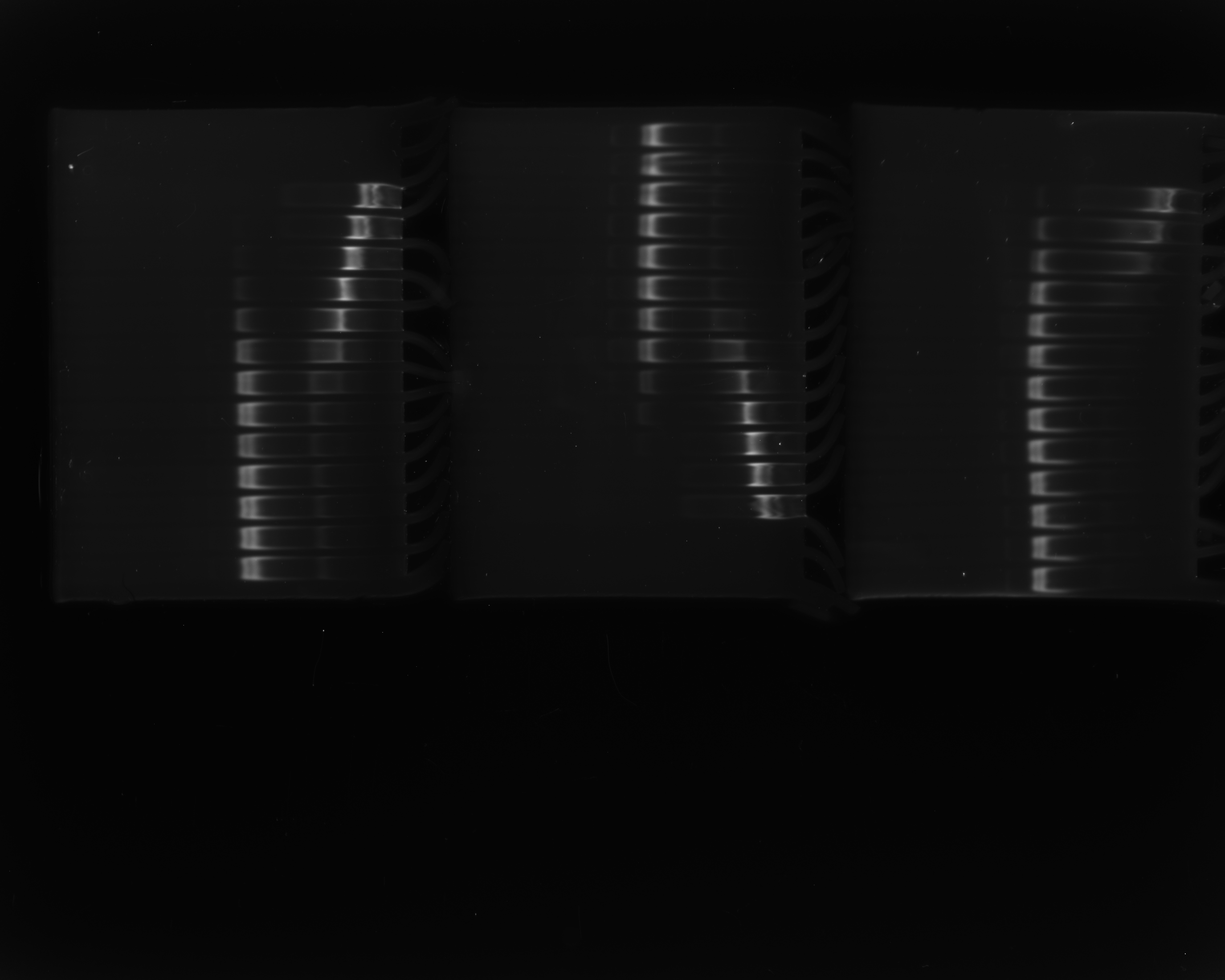

Supplement: Supplementary file 4 — Source data Fig. 2 [file 44319_2024_306_MOESM4_ESM.zip › EMBOR-2024-60481V2_SourceDataForFigure 2/Figure 2C/Figure 2C/Figure 2C repeat 1.2.tif]

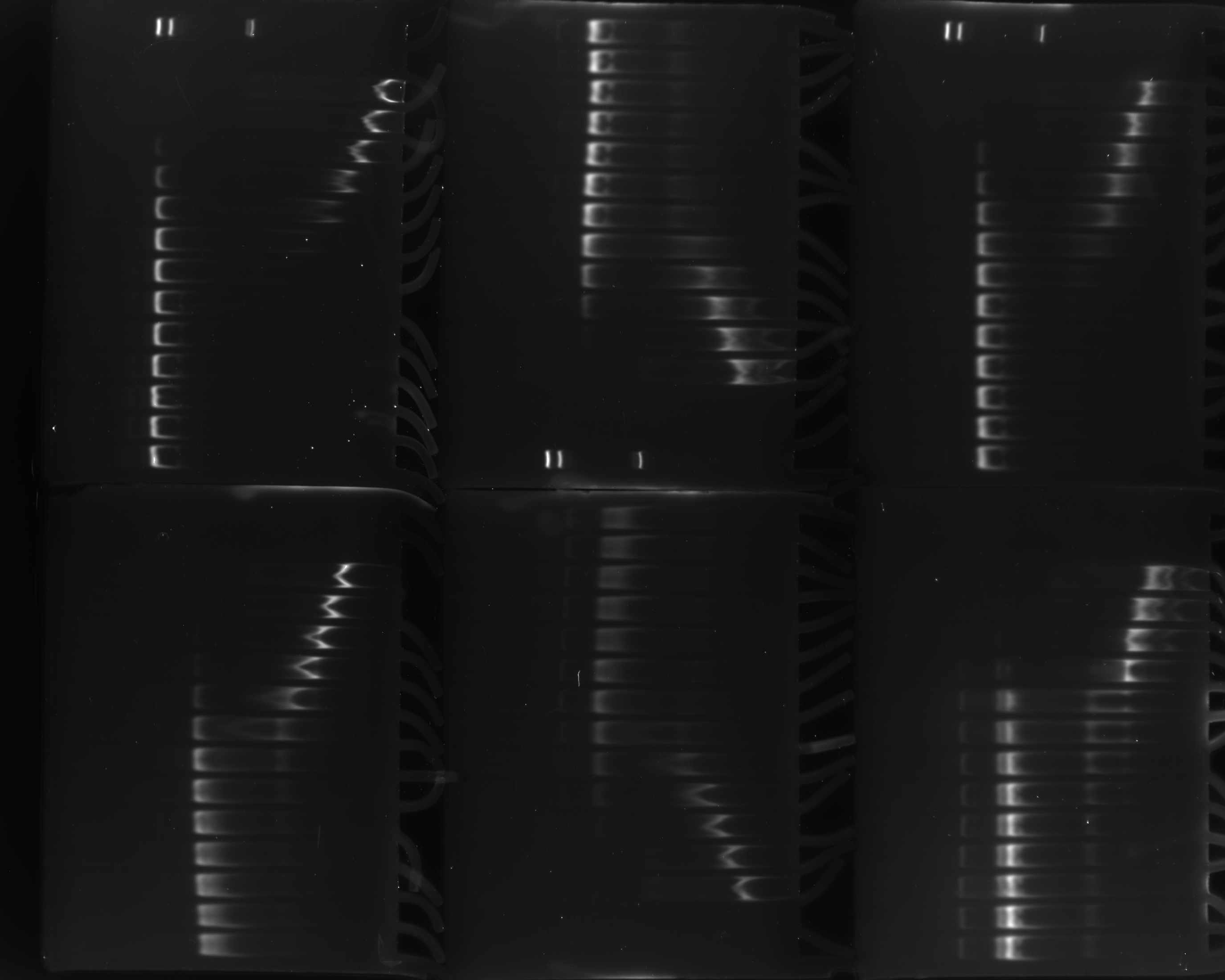

Supplement: Supplementary file 4 — Source data Fig. 2 [file 44319_2024_306_MOESM4_ESM.zip › EMBOR-2024-60481V2_SourceDataForFigure 2/Figure 2C/Figure 2C/Figure 2C repeat 1.1.tif]

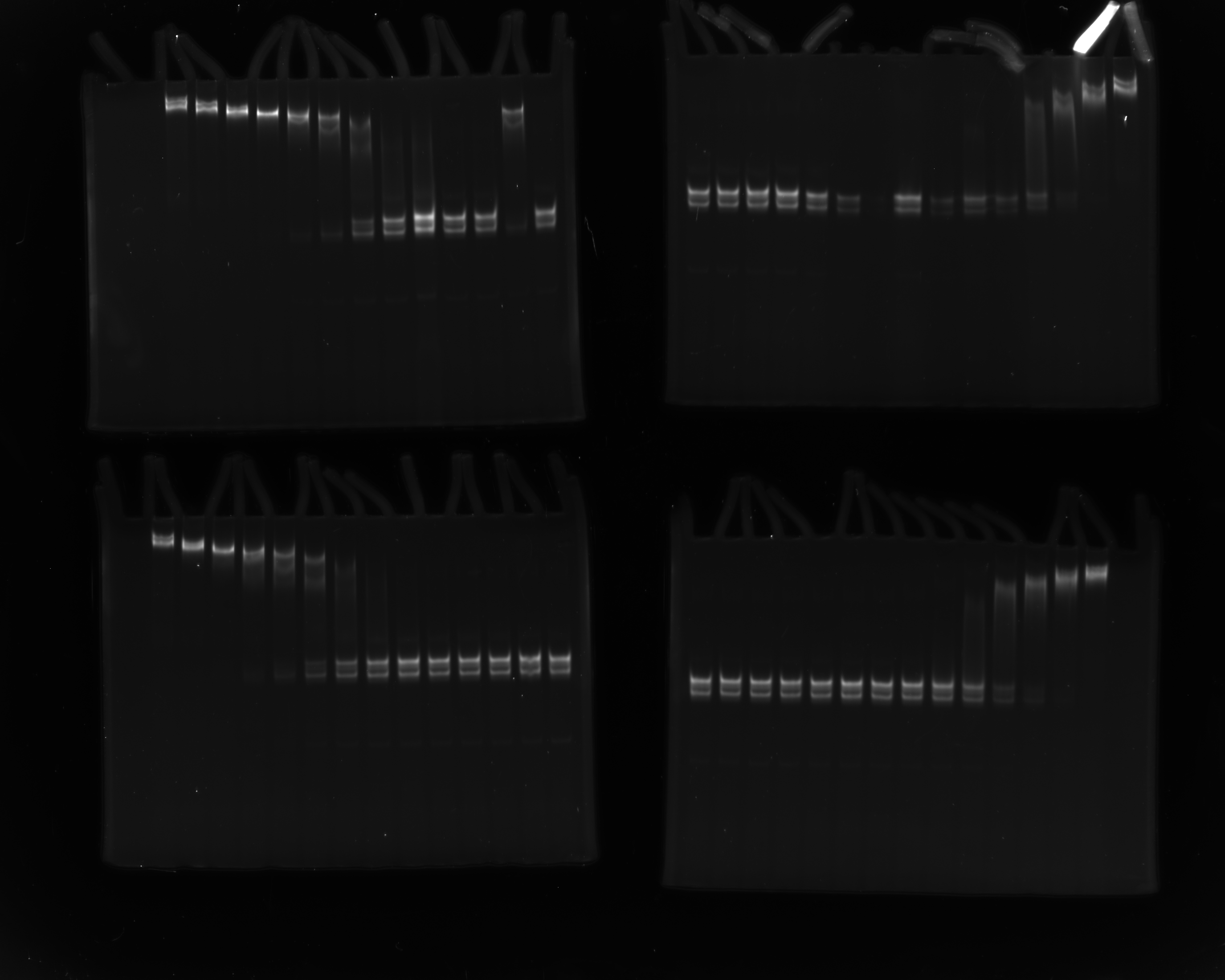

Supplement: Supplementary file 4 — Source data Fig. 2 [file 44319_2024_306_MOESM4_ESM.zip › EMBOR-2024-60481V2_SourceDataForFigure 2/Figure 2C/Figure 2E/Figure2E repeat 1.tif]

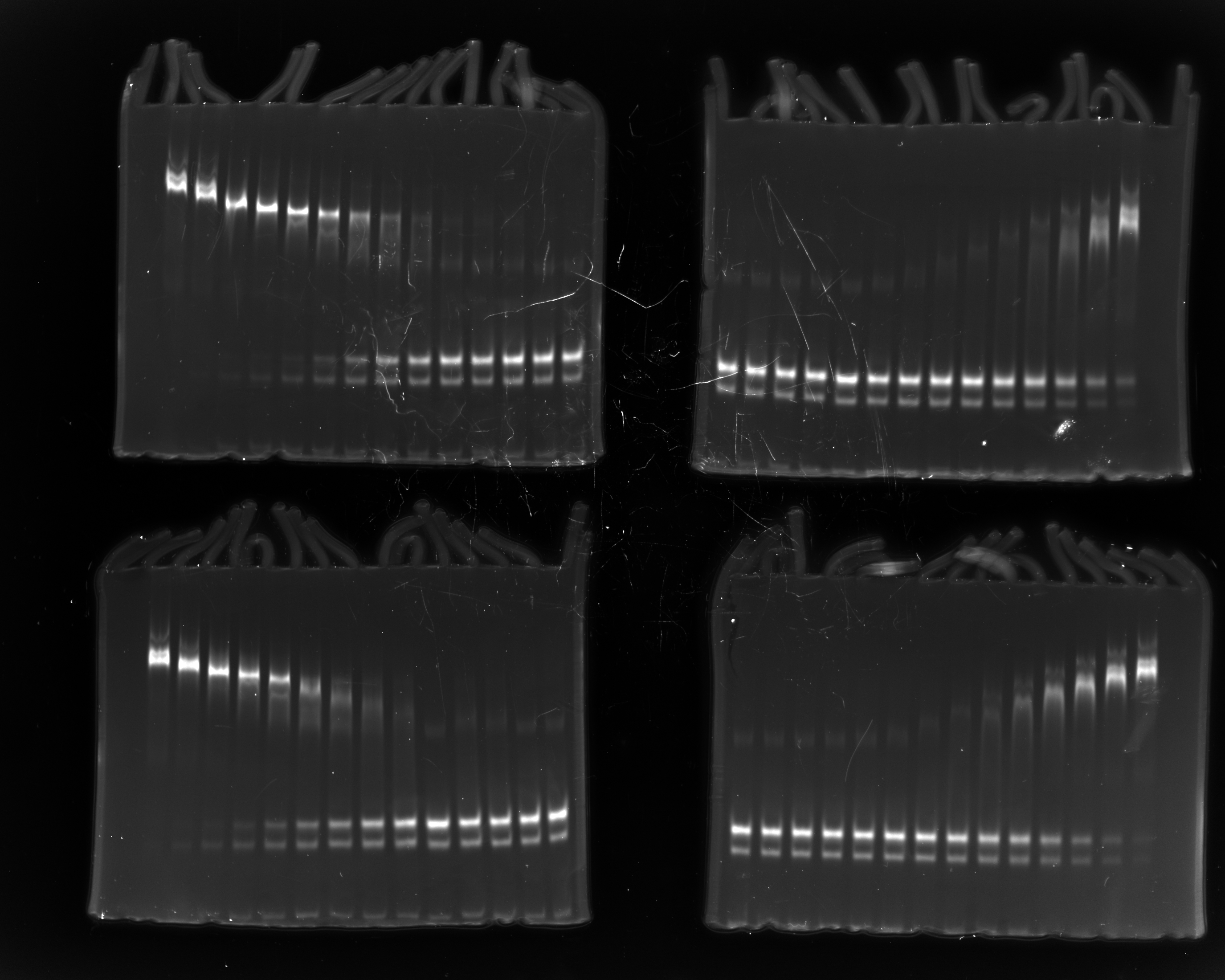

Supplement: Supplementary file 4 — Source data Fig. 2 [file 44319_2024_306_MOESM4_ESM.zip › EMBOR-2024-60481V2_SourceDataForFigure 2/Figure 2C/Figure 2E/Figure 2E.tif]

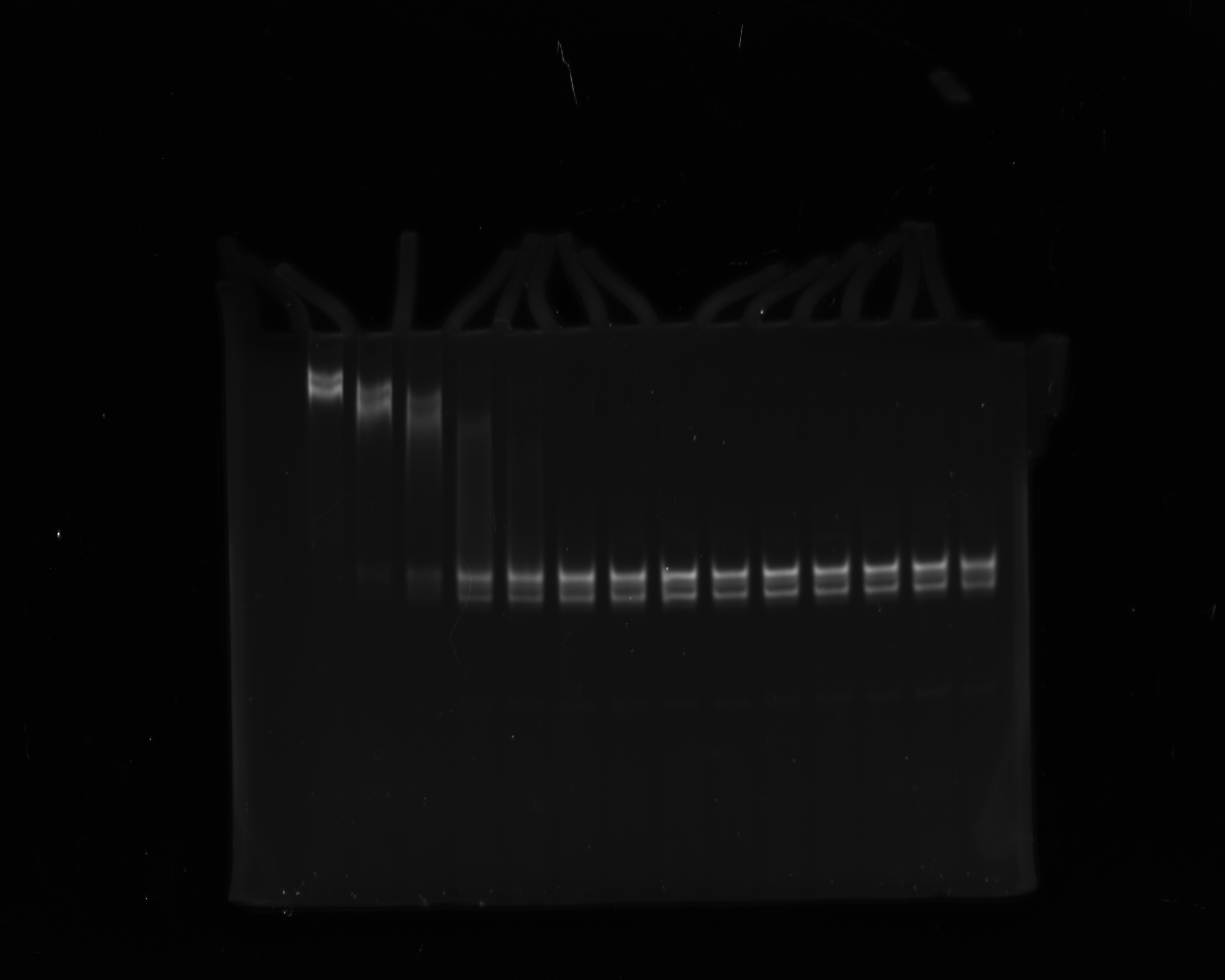

Supplement: Supplementary file 4 — Source data Fig. 2 [file 44319_2024_306_MOESM4_ESM.zip › EMBOR-2024-60481V2_SourceDataForFigure 2/Figure 2C/Figure 2E/Figure 2 repeat 1.2.tif]

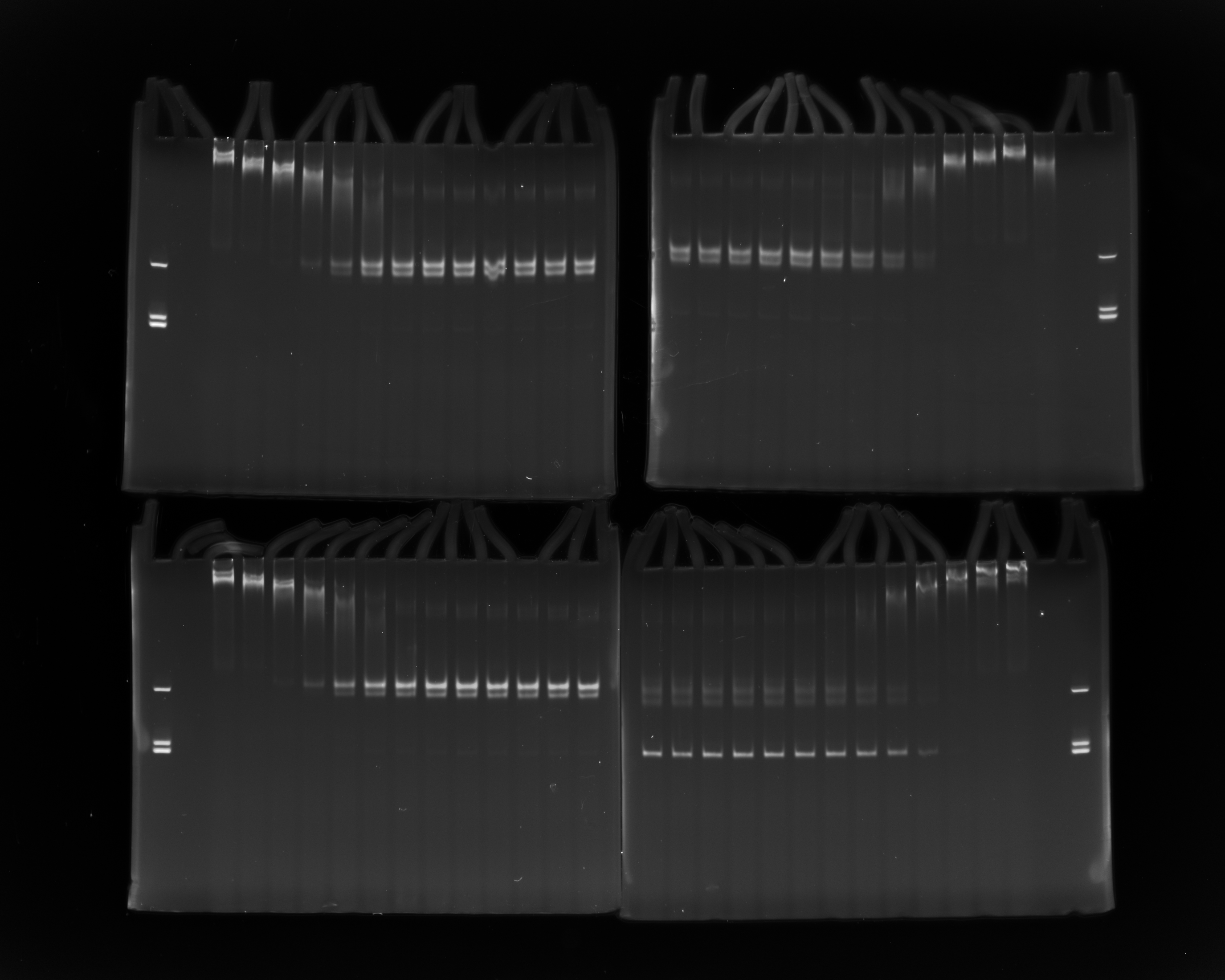

Supplement: Supplementary file 5 — Source data Fig. 3 [file 44319_2024_306_MOESM5_ESM.zip › EMBOR-2024-60481V2_SourceDataForFigure 3/Figure 3B/Figure 3B 2.tif]

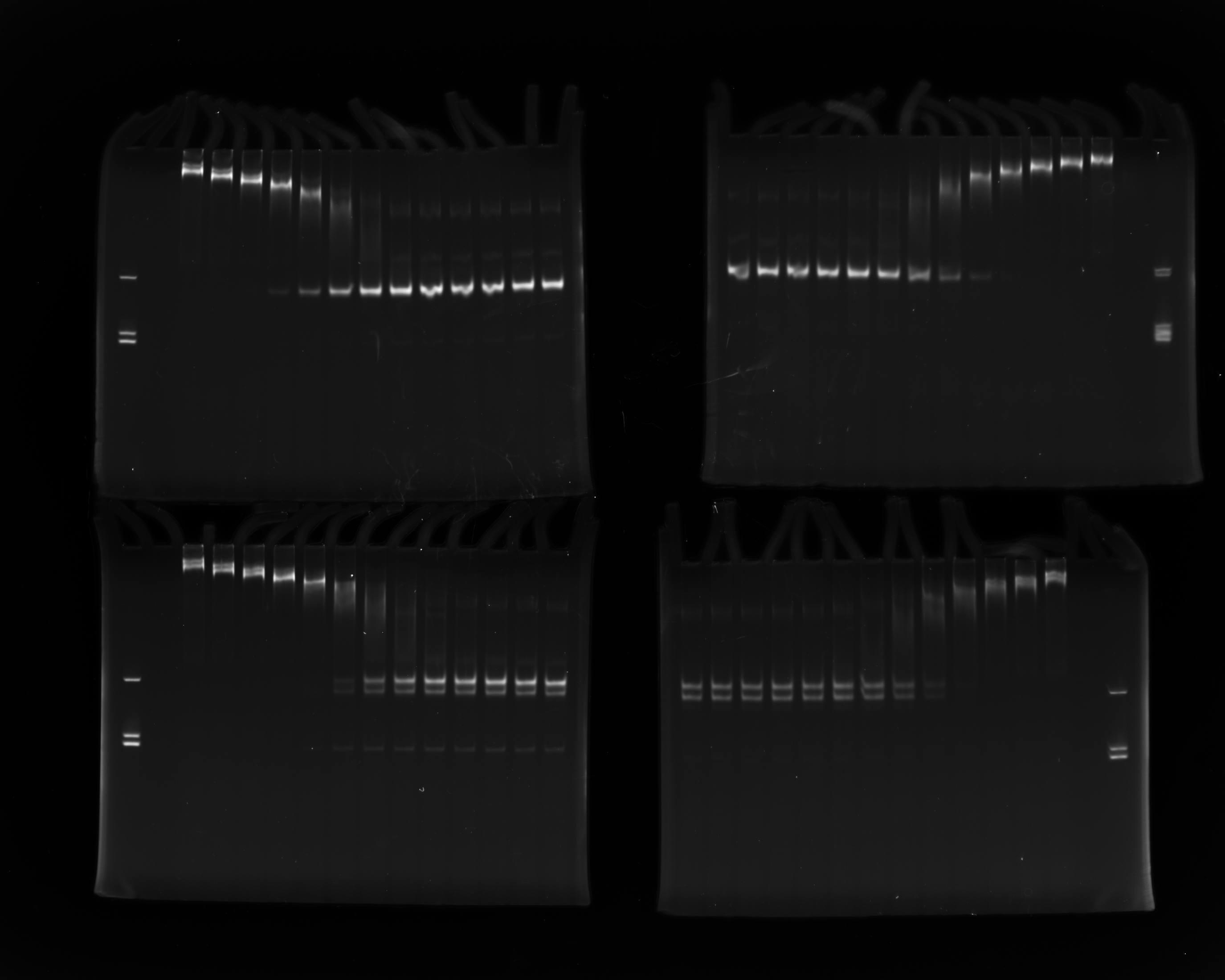

Supplement: Supplementary file 5 — Source data Fig. 3 [file 44319_2024_306_MOESM5_ESM.zip › EMBOR-2024-60481V2_SourceDataForFigure 3/Figure 3B/Figure 3B 1.tif]

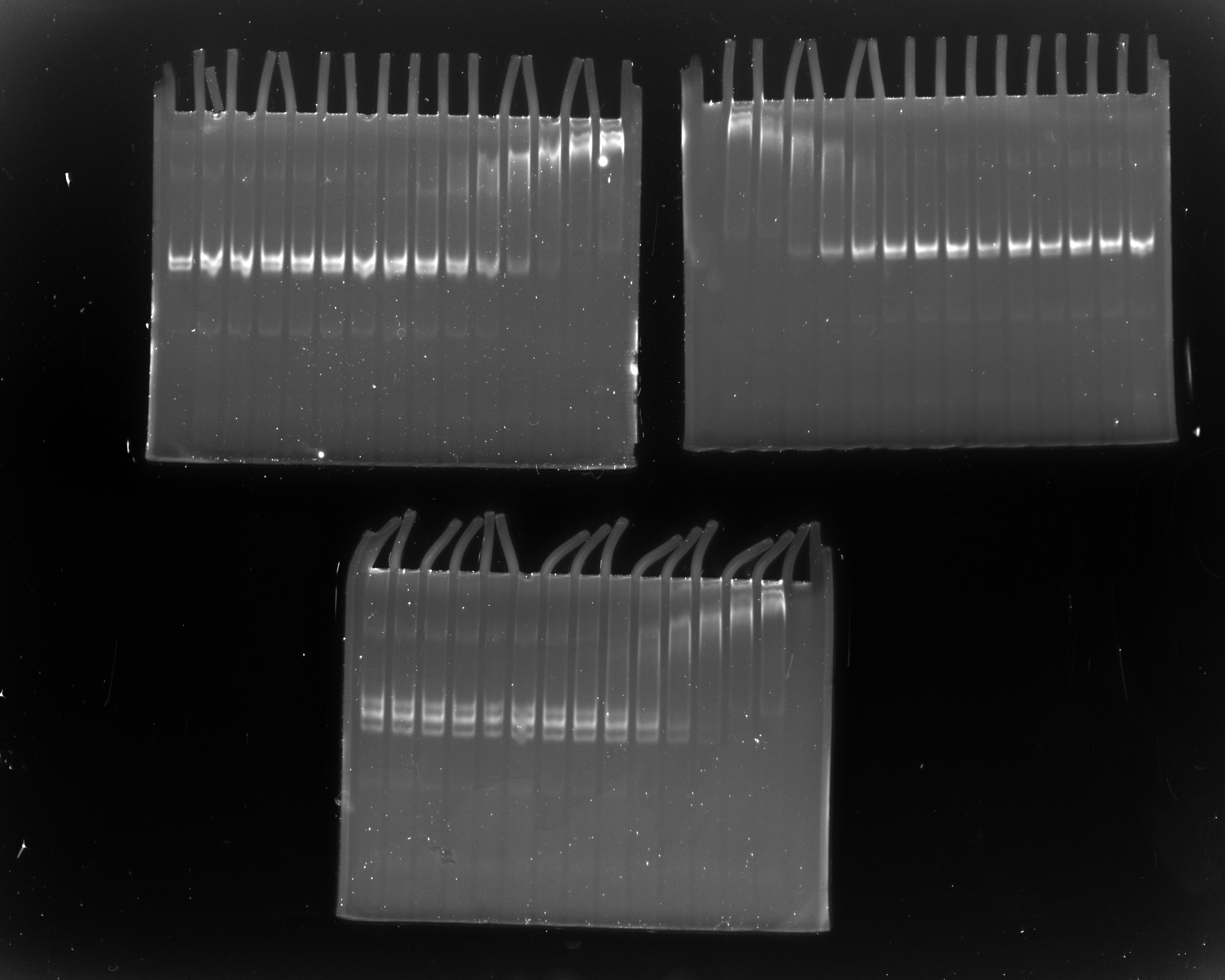

Supplement: Supplementary file 5 — Source data Fig. 3 [file 44319_2024_306_MOESM5_ESM.zip › EMBOR-2024-60481V2_SourceDataForFigure 3/Figure 3B/Figure 3B repeat 3.2.tif]

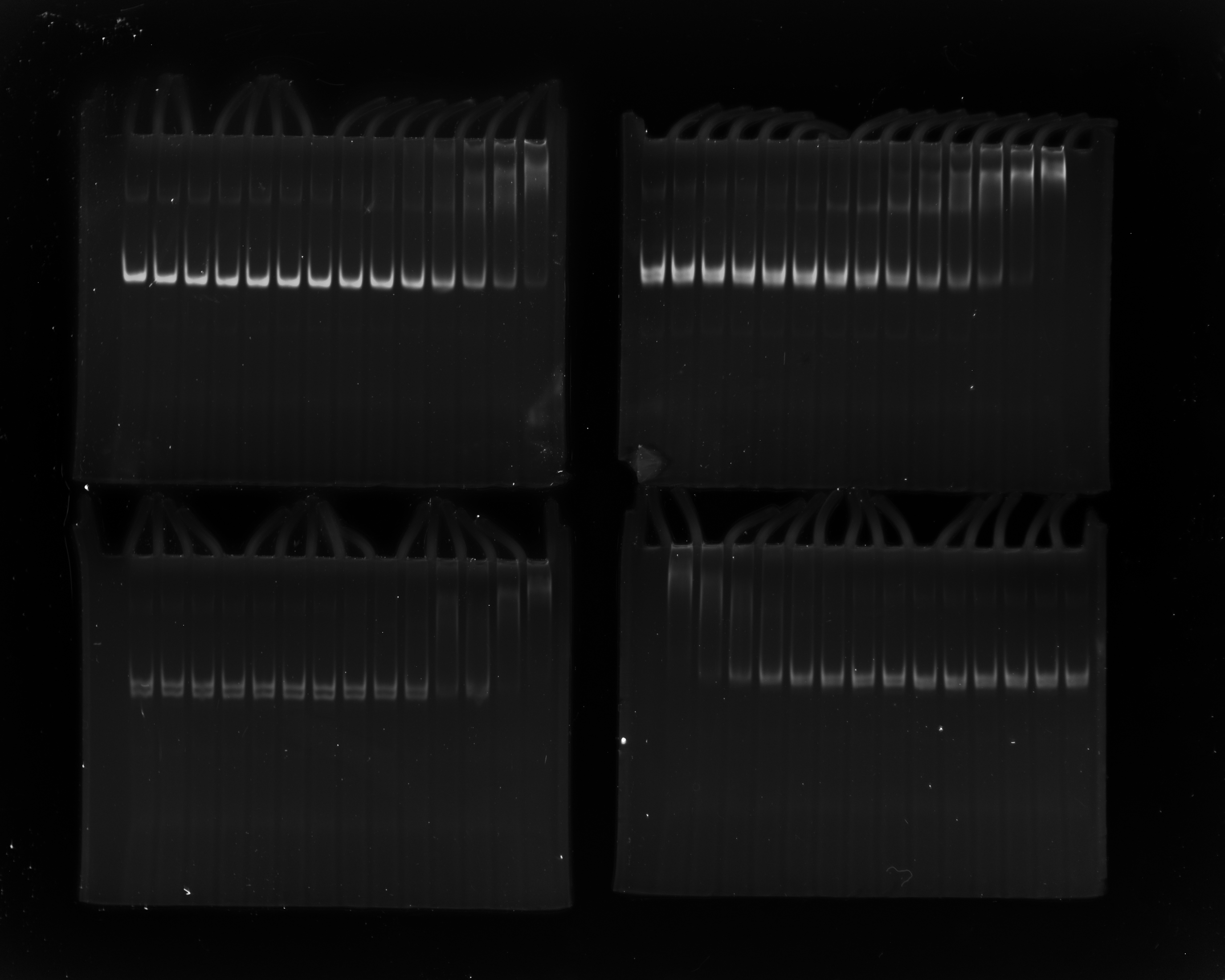

Supplement: Supplementary file 5 — Source data Fig. 3 [file 44319_2024_306_MOESM5_ESM.zip › EMBOR-2024-60481V2_SourceDataForFigure 3/Figure 3B/Figure 3B repeat 1.1.tif]

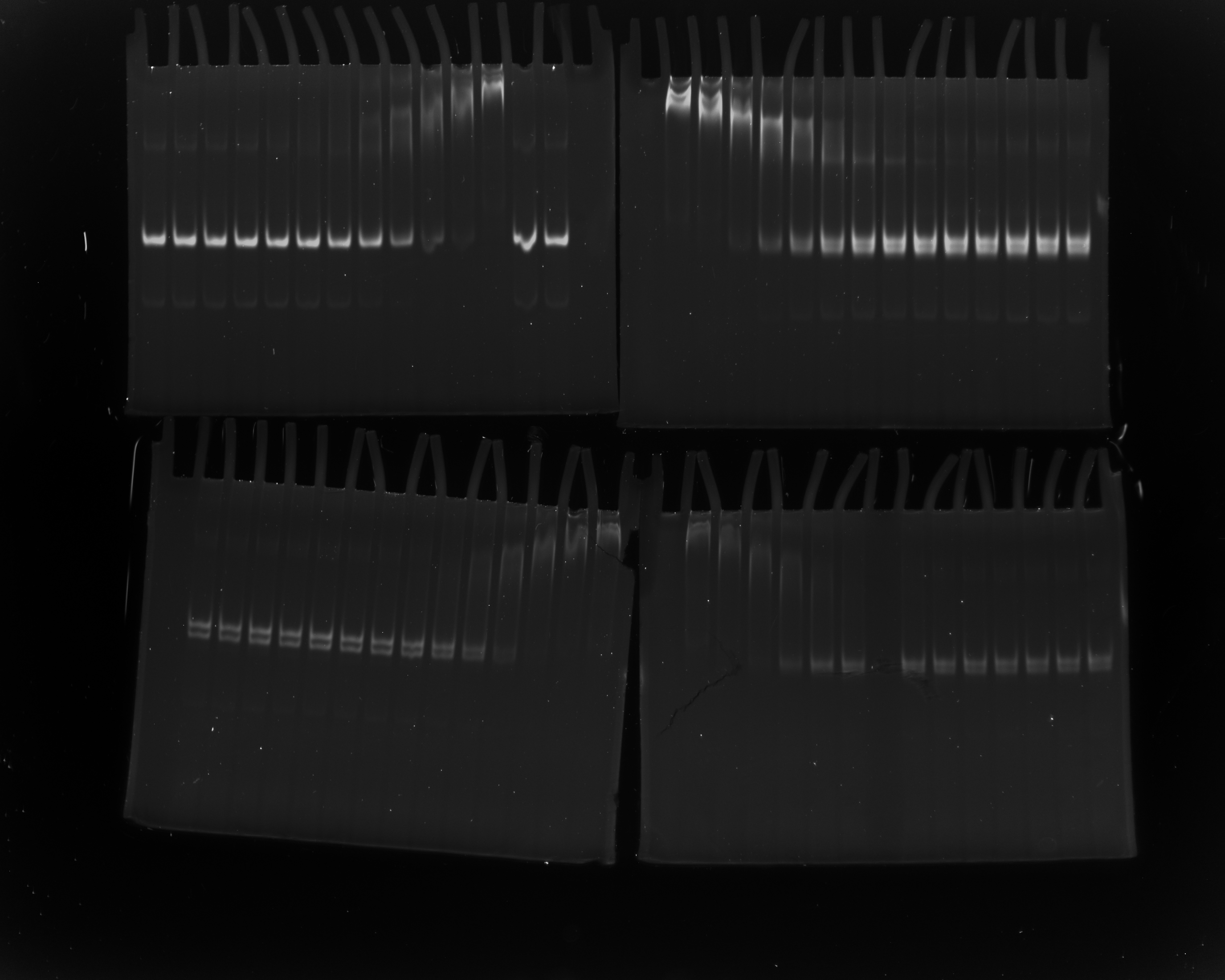

Supplement: Supplementary file 5 — Source data Fig. 3 [file 44319_2024_306_MOESM5_ESM.zip › EMBOR-2024-60481V2_SourceDataForFigure 3/Figure 3B/Figure 3B repeat 3.1.tif]

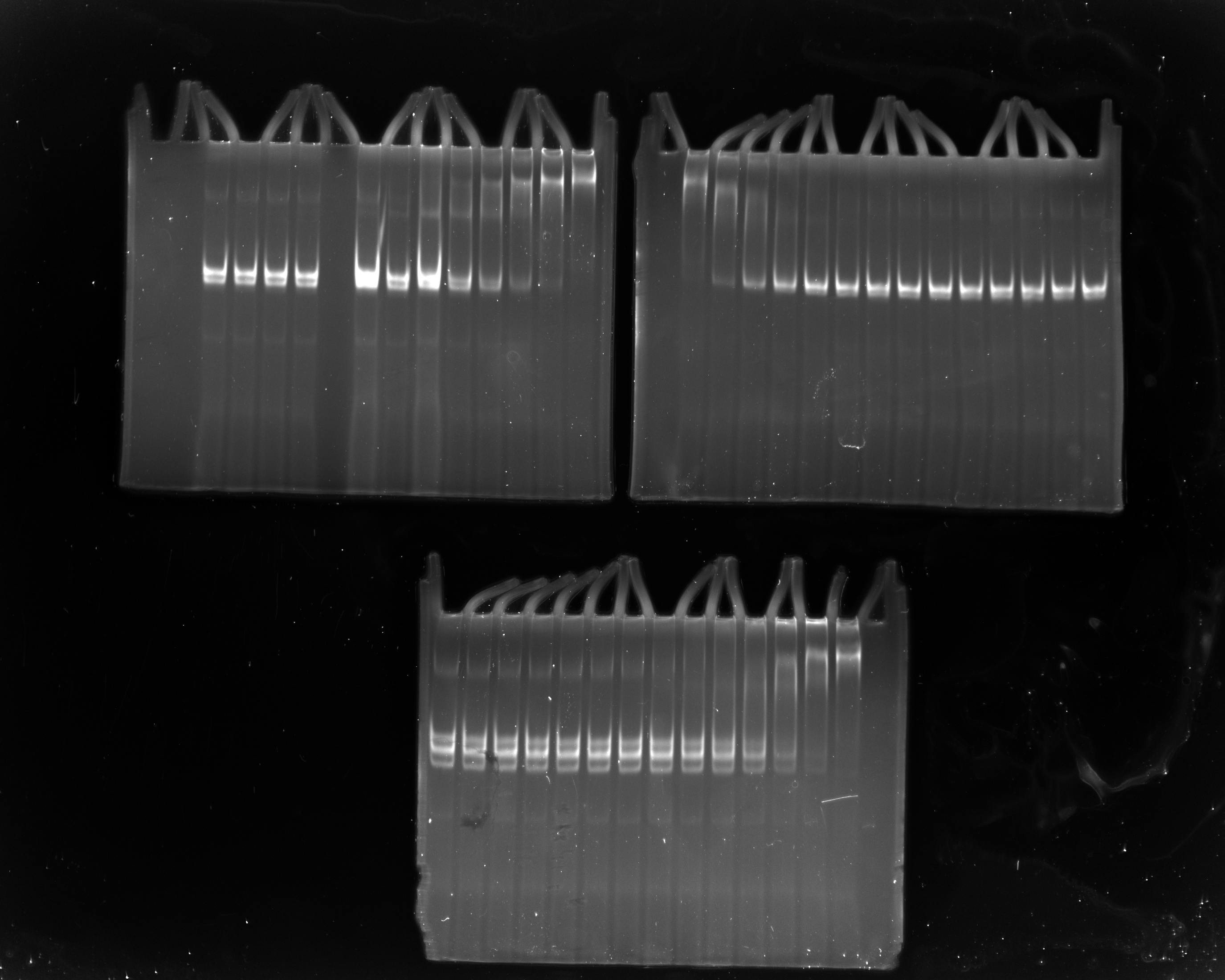

Supplement: Supplementary file 5 — Source data Fig. 3 [file 44319_2024_306_MOESM5_ESM.zip › EMBOR-2024-60481V2_SourceDataForFigure 3/Figure 3B/Figure 3B repeat 1.2.tif]

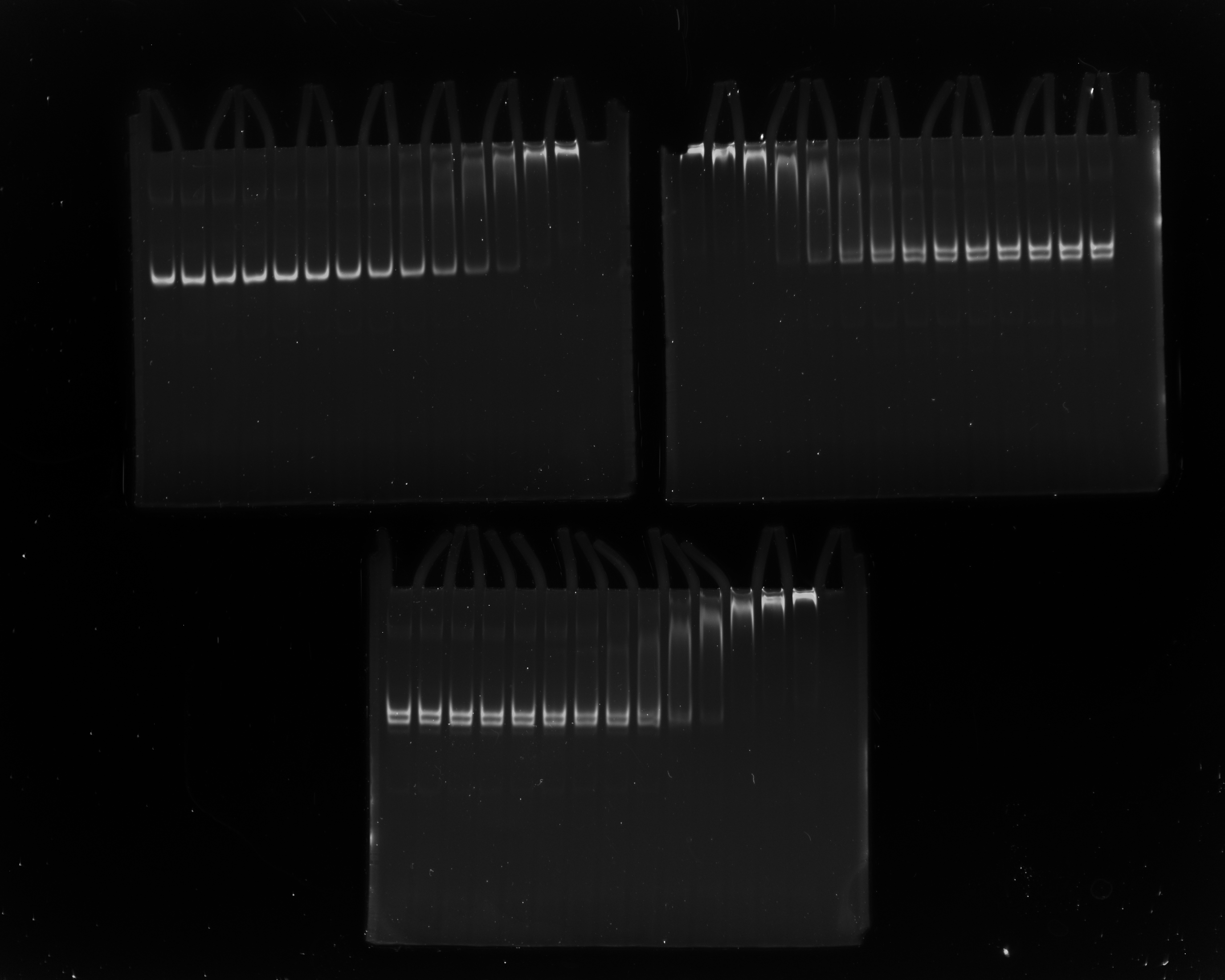

Supplement: Supplementary file 5 — Source data Fig. 3 [file 44319_2024_306_MOESM5_ESM.zip › EMBOR-2024-60481V2_SourceDataForFigure 3/Figure 3B/Figure 3B repeat 2.1.tif]

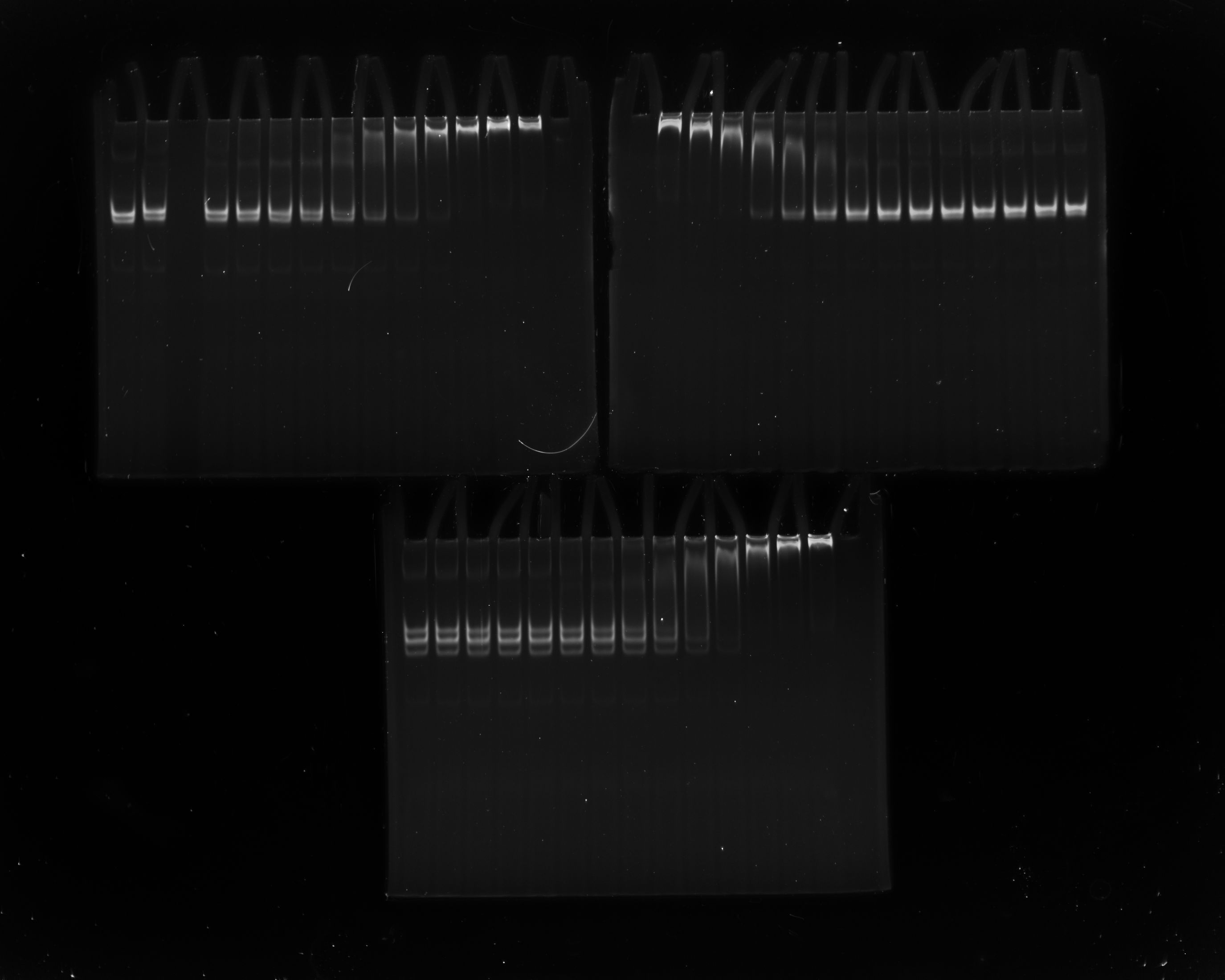

Supplement: Supplementary file 5 — Source data Fig. 3 [file 44319_2024_306_MOESM5_ESM.zip › EMBOR-2024-60481V2_SourceDataForFigure 3/Figure 3B/Figure 3B repeat 2.2.tif]

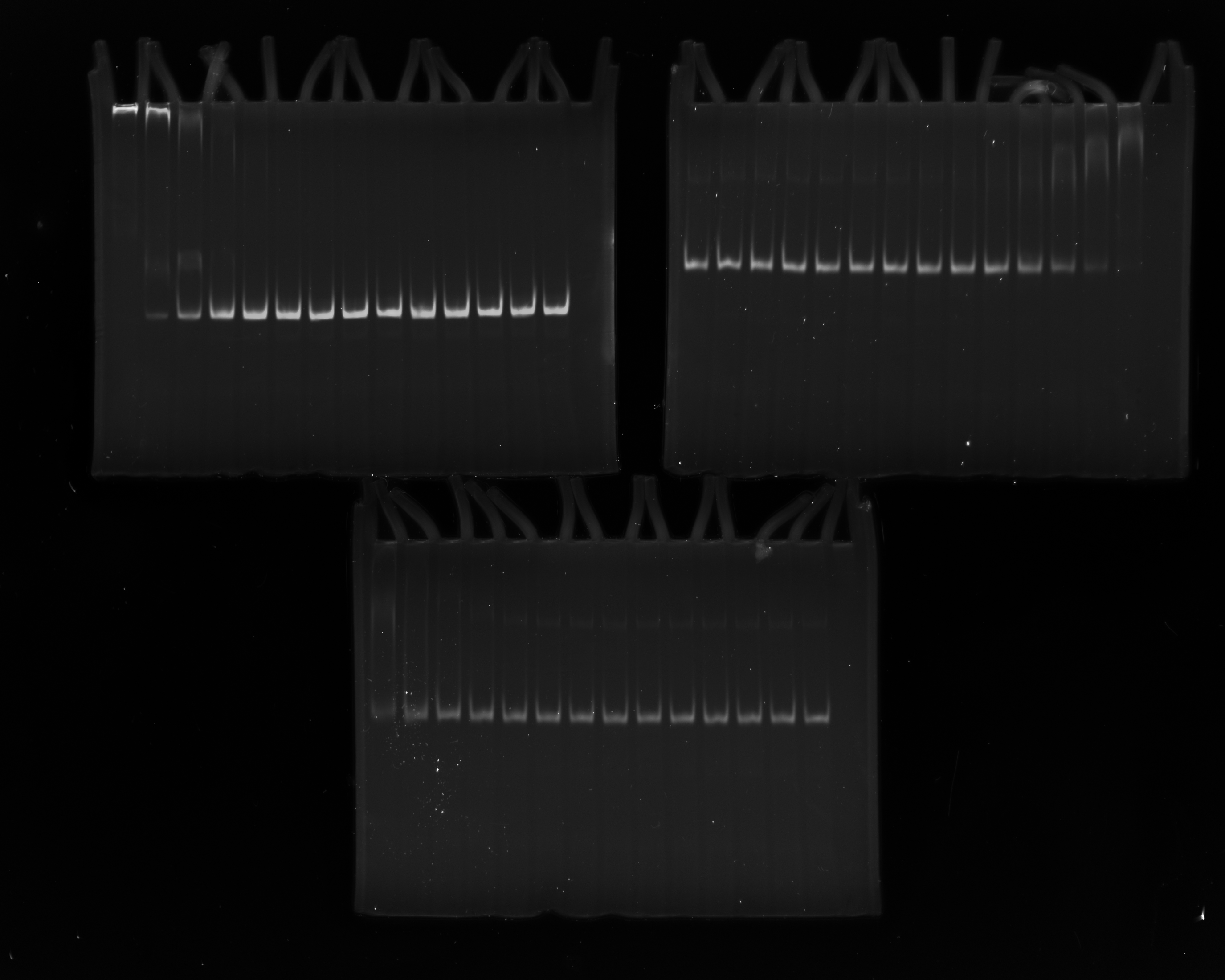

Supplement: Supplementary file 6 — Source data Fig. 4 [file 44319_2024_306_MOESM6_ESM.zip › EMBOR-2024-60481V2_SourceDataForFigure 4/Figure 4E/Figure 4E.tif]

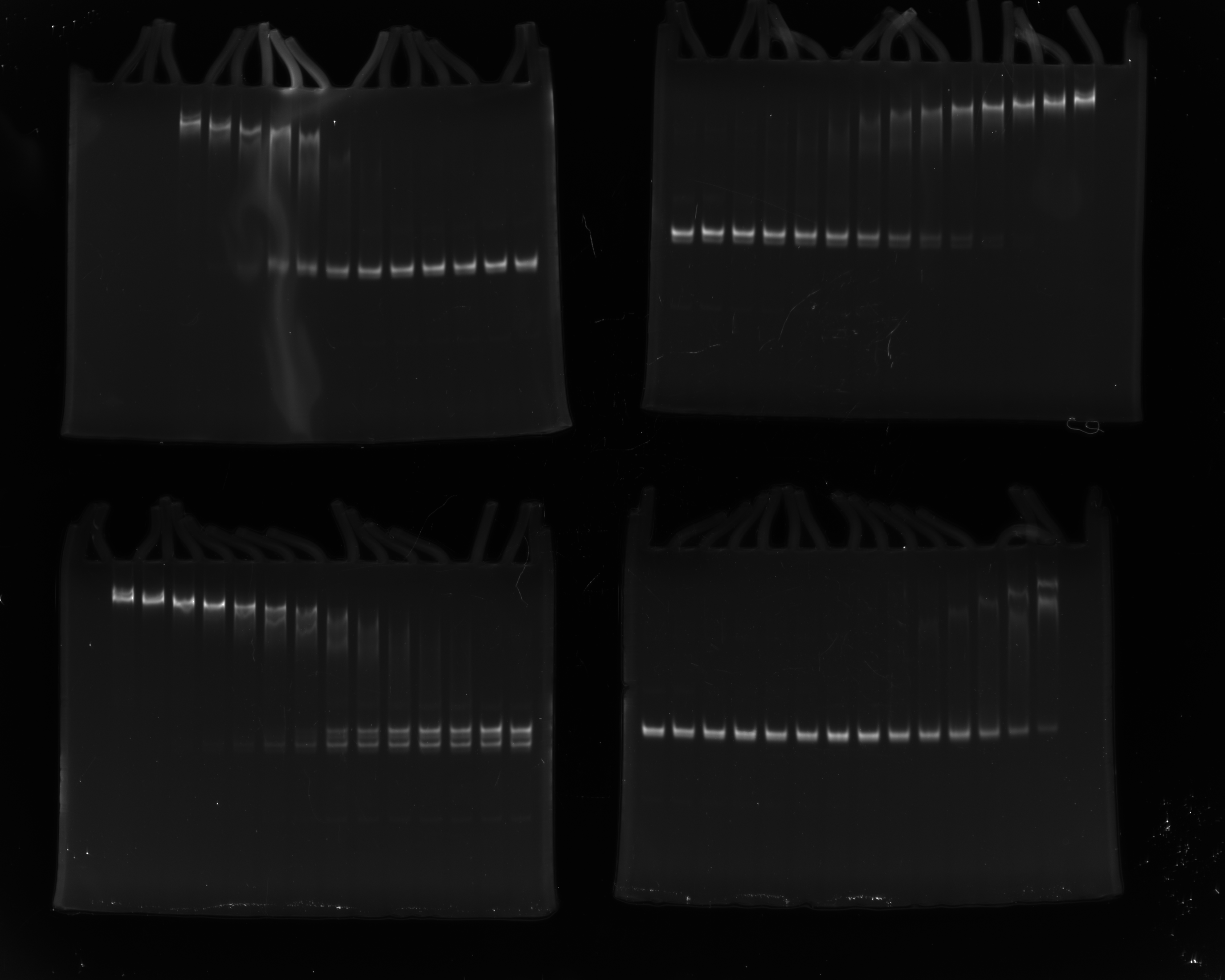

Supplement: Supplementary file 6 — Source data Fig. 4 [file 44319_2024_306_MOESM6_ESM.zip › EMBOR-2024-60481V2_SourceDataForFigure 4/Figure 4E/Figure 4E repeat.tif]

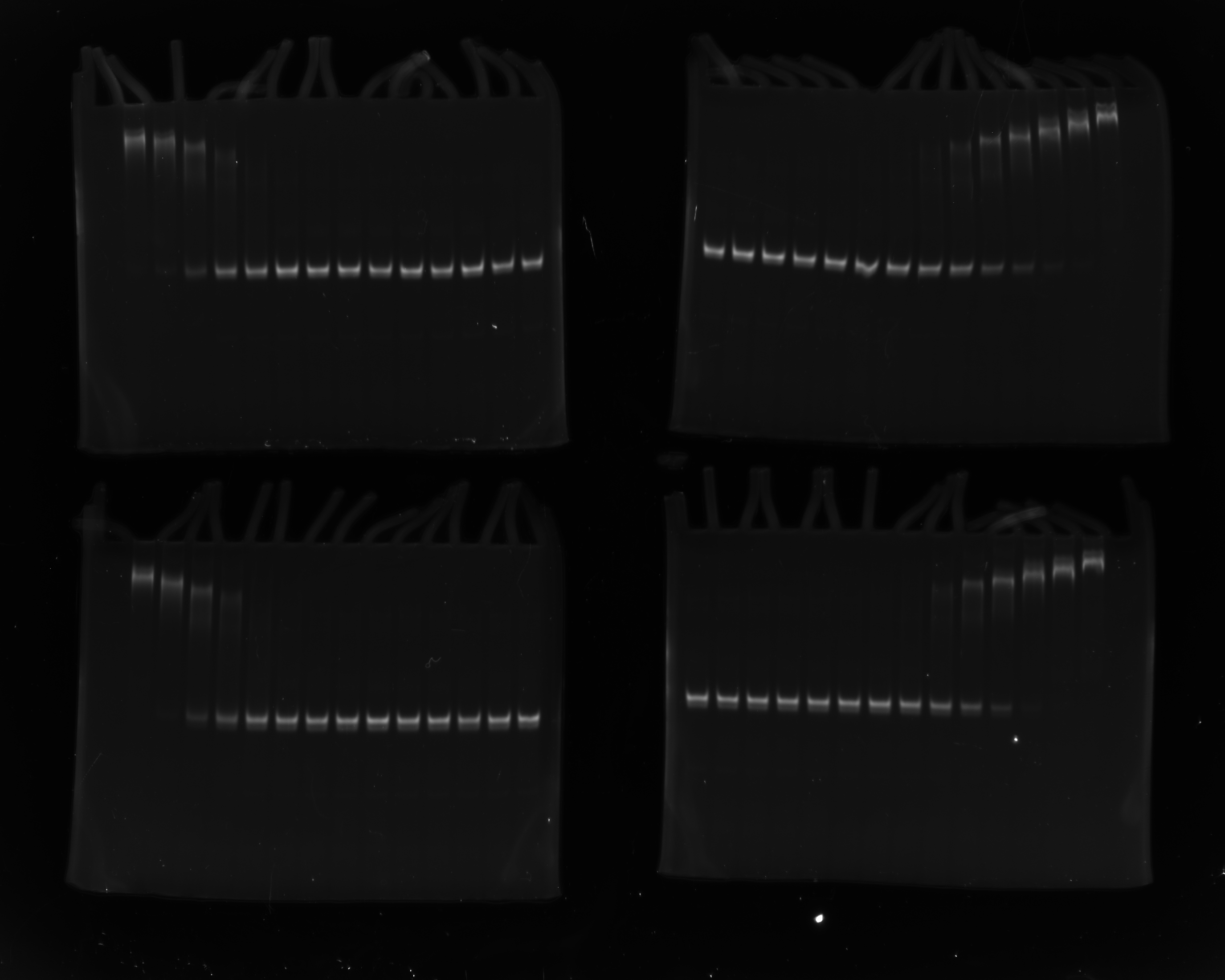

Supplement: Supplementary file 6 — Source data Fig. 4 [file 44319_2024_306_MOESM6_ESM.zip › EMBOR-2024-60481V2_SourceDataForFigure 4/Figure 4B/Figure 4B repeat.tif]

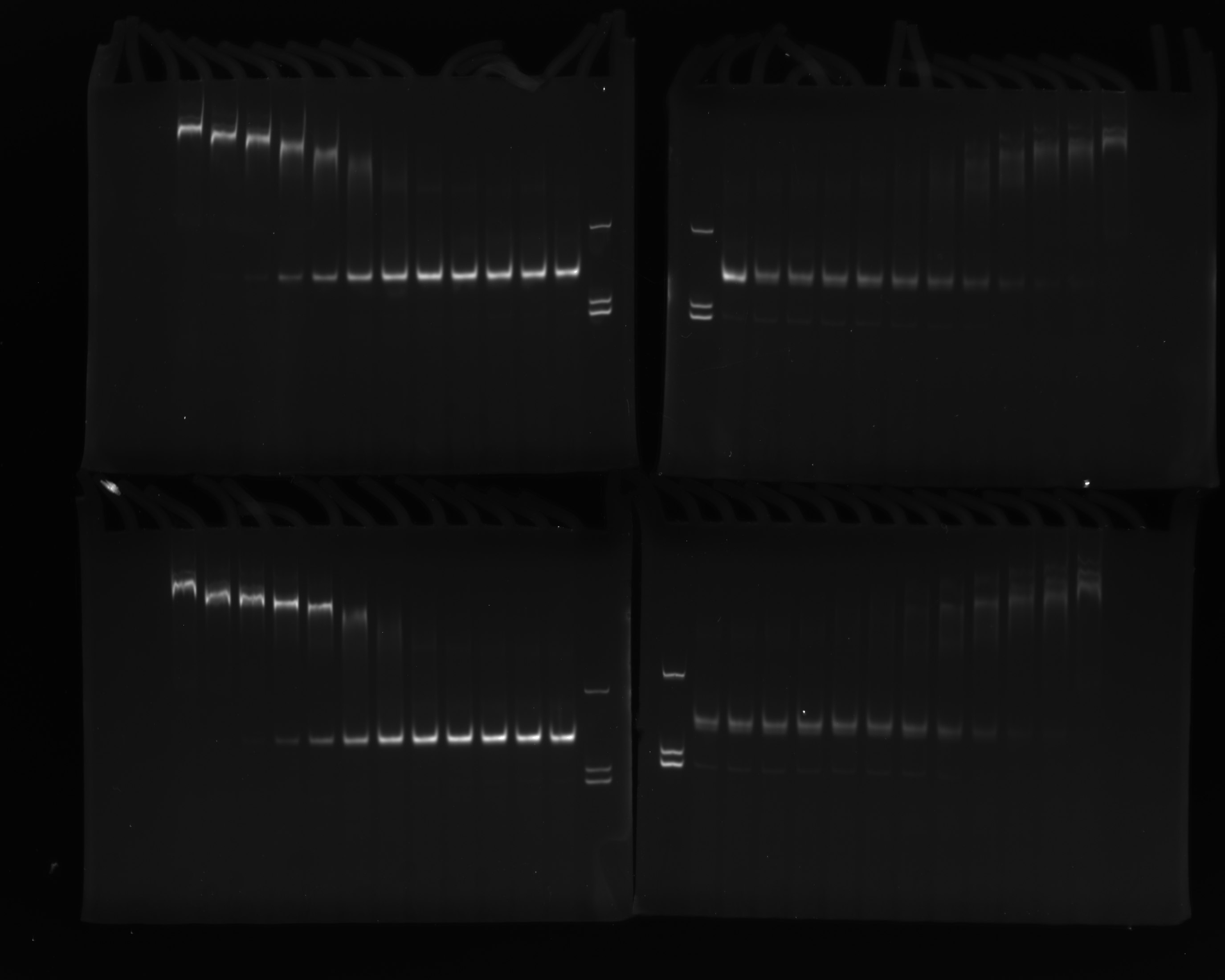

Supplement: Supplementary file 6 — Source data Fig. 4 [file 44319_2024_306_MOESM6_ESM.zip › EMBOR-2024-60481V2_SourceDataForFigure 4/Figure 4B/Figure 4B W330R D333N.tif]

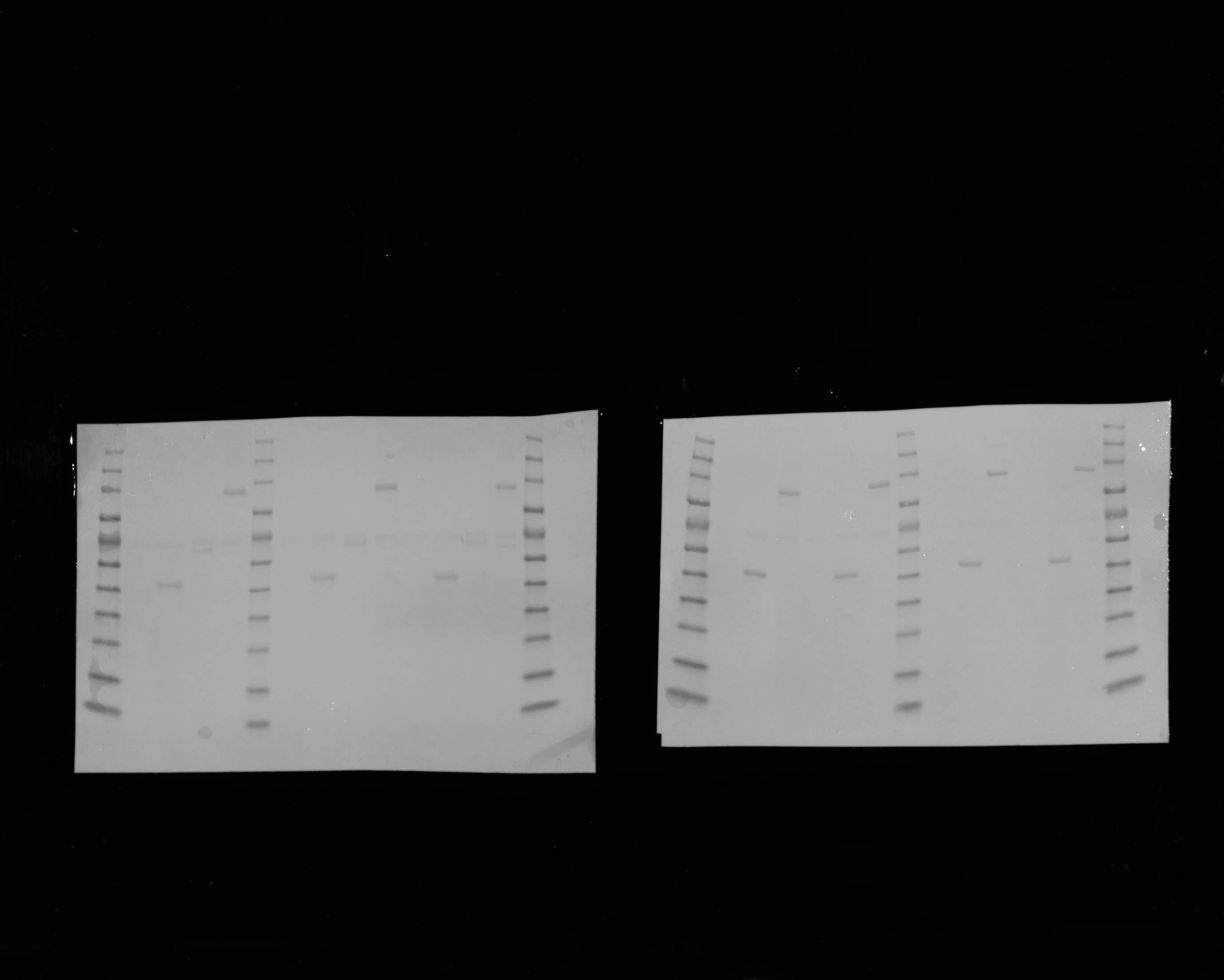

Supplement: Supplementary file 6 — Source data Fig. 4 [file 44319_2024_306_MOESM6_ESM.zip › EMBOR-2024-60481V2_SourceDataForFigure 4/Figure 4D/APponceau.tif]

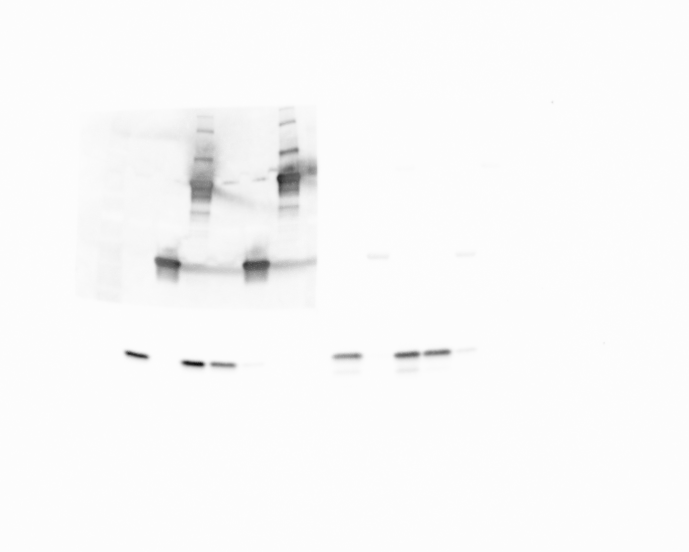

Supplement: Supplementary file 6 — Source data Fig. 4 [file 44319_2024_306_MOESM6_ESM.zip › EMBOR-2024-60481V2_SourceDataForFigure 4/Figure 4D/APH3H2B.tif]

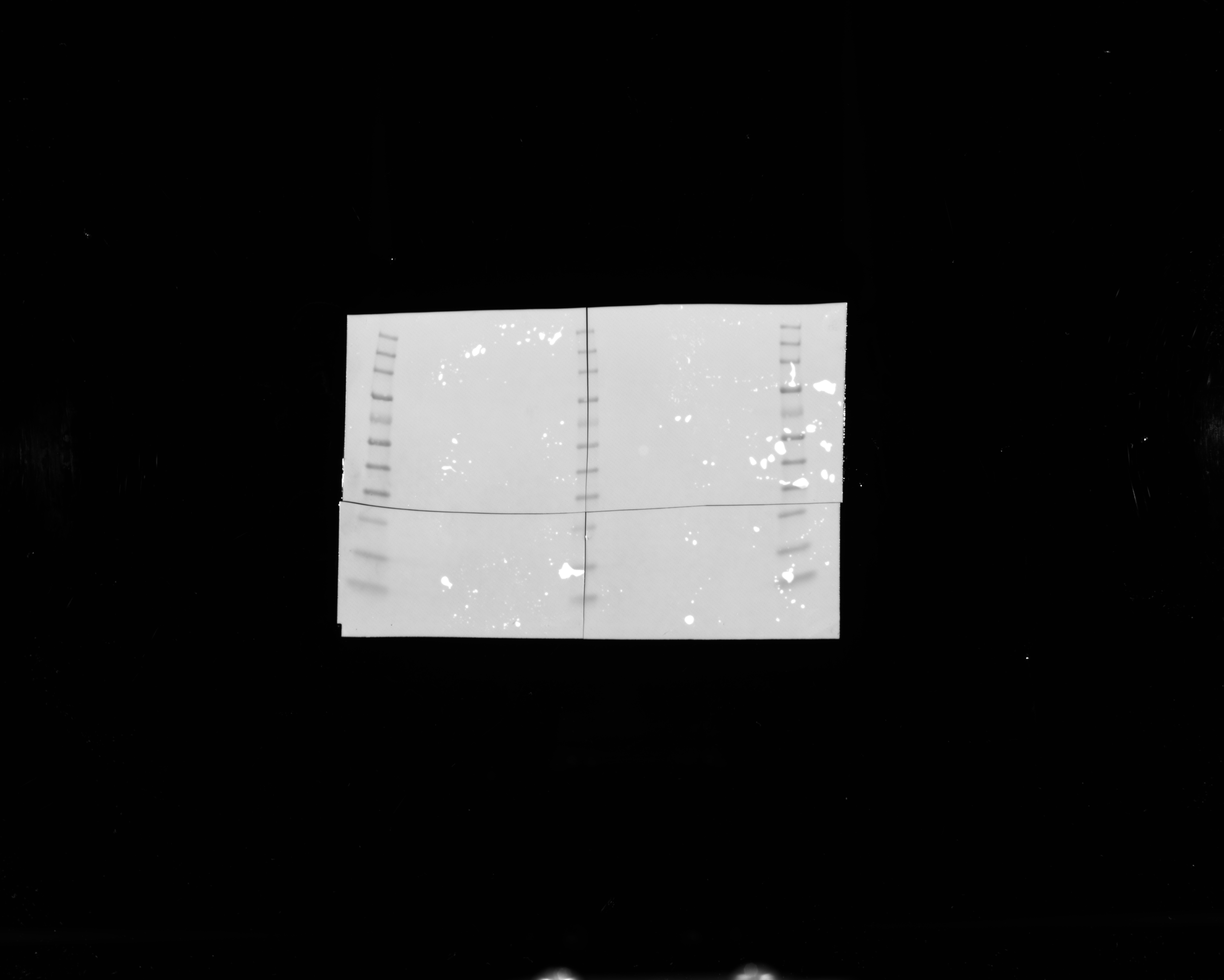

Supplement: Supplementary file 6 — Source data Fig. 4 [file 44319_2024_306_MOESM6_ESM.zip › EMBOR-2024-60481V2_SourceDataForFigure 4/Figure 4D/APmarker.tif]

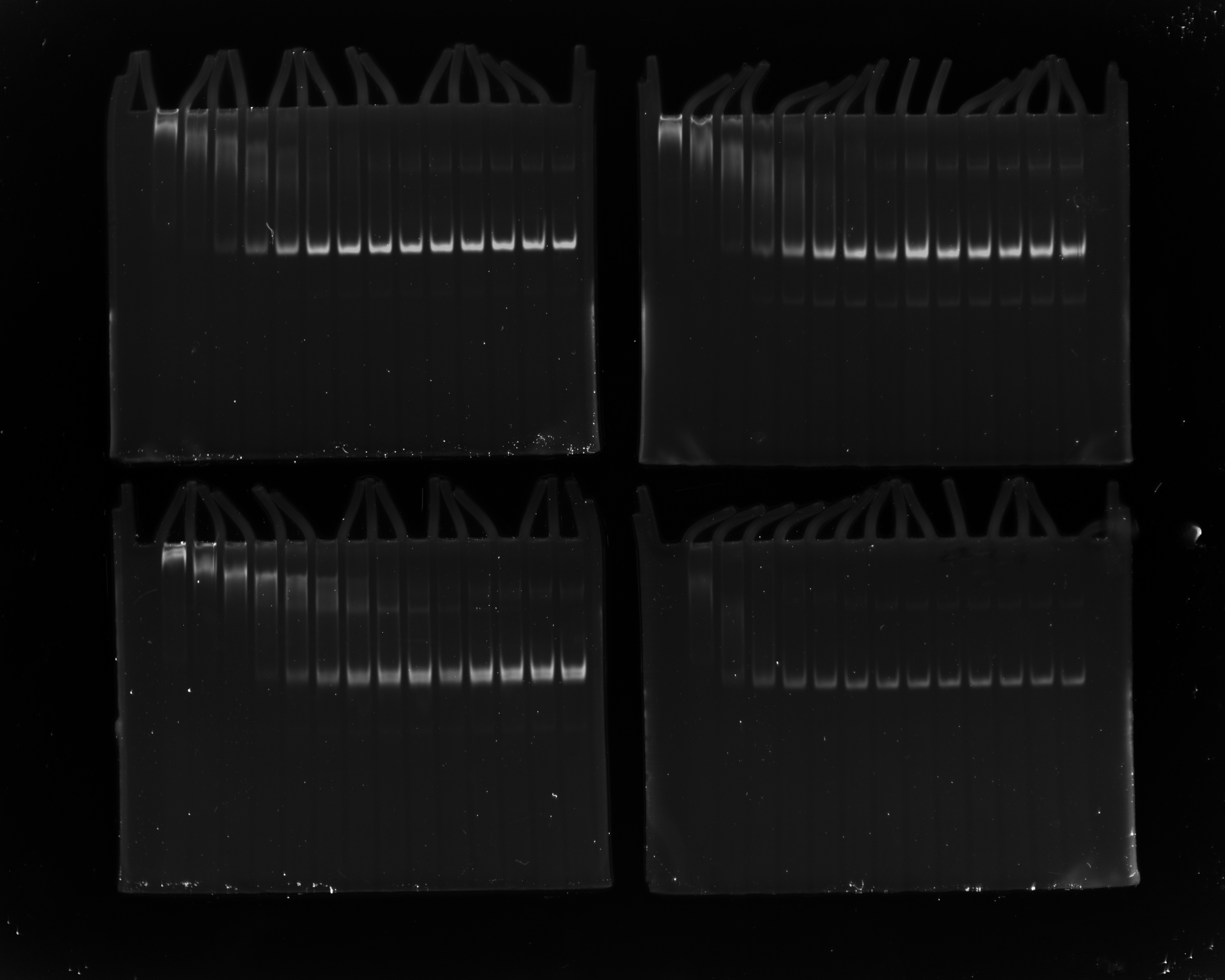

Supplement: Supplementary file 6 — Source data Fig. 4 [file 44319_2024_306_MOESM6_ESM.zip › EMBOR-2024-60481V2_SourceDataForFigure 4/Figure 4A/Figure 4A.tif]

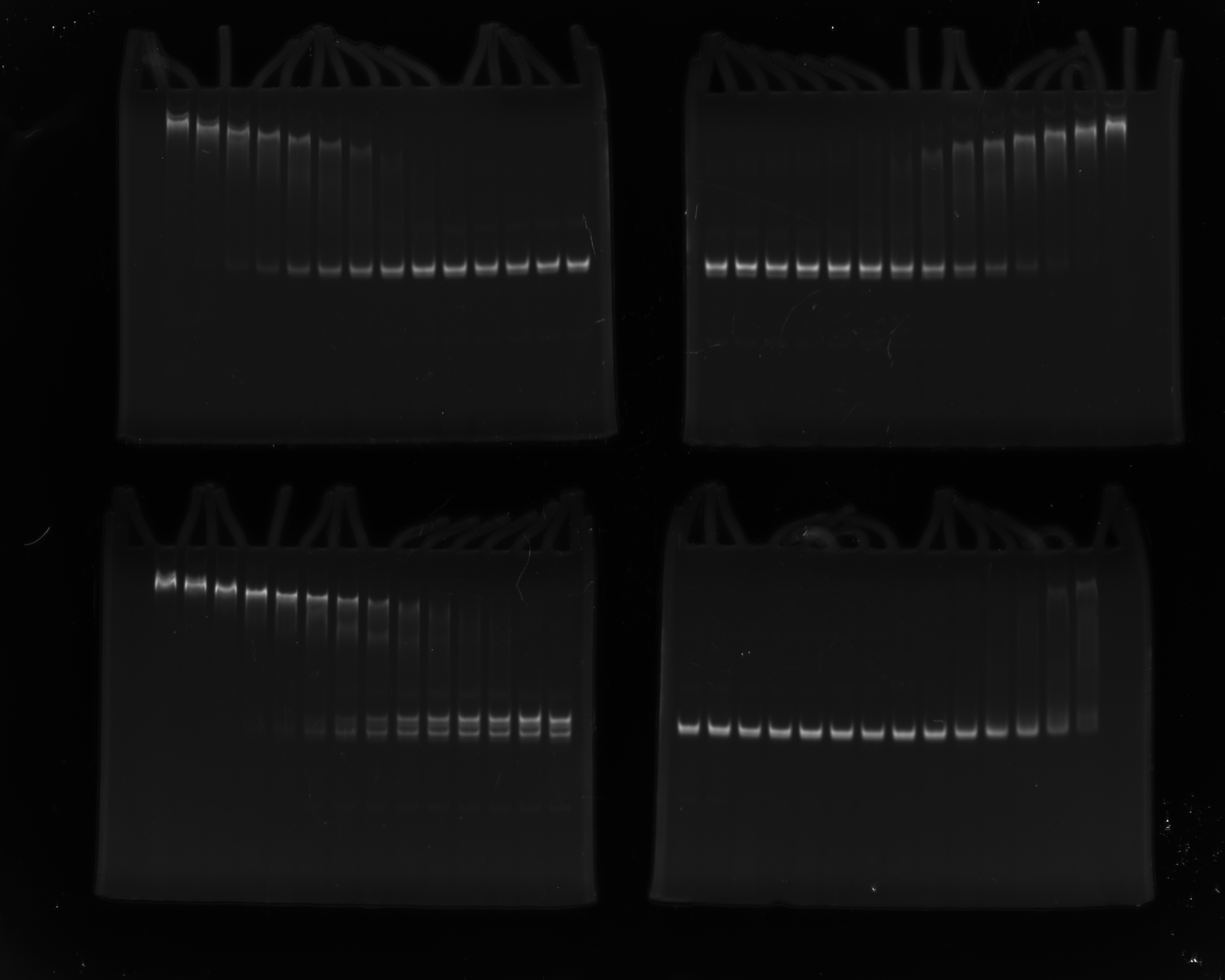

Supplement: Supplementary file 6 — Source data Fig. 4 [file 44319_2024_306_MOESM6_ESM.zip › EMBOR-2024-60481V2_SourceDataForFigure 4/Figure 4A/Figure 4A repeat 2.tif]

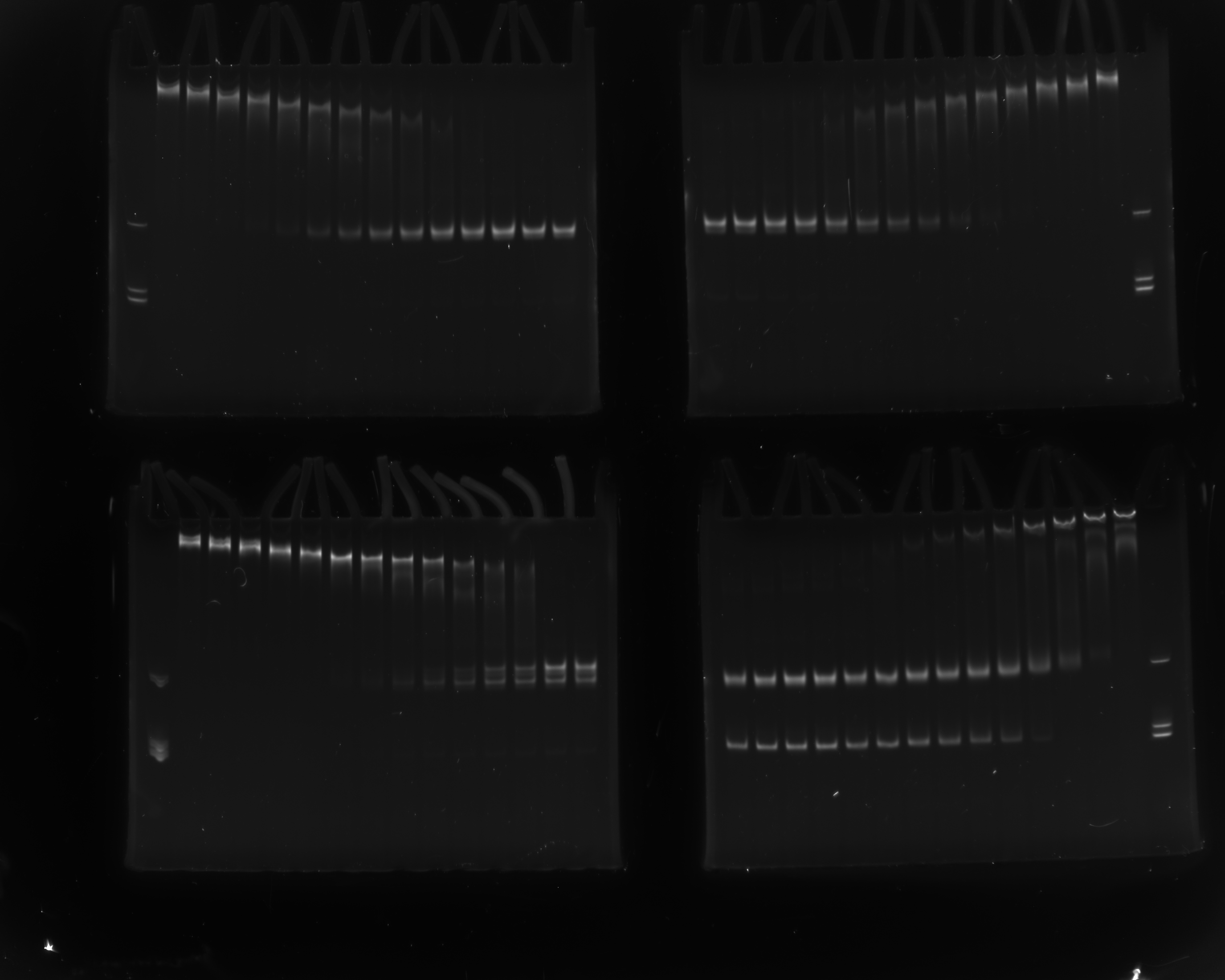

Supplement: Supplementary file 6 — Source data Fig. 4 [file 44319_2024_306_MOESM6_ESM.zip › EMBOR-2024-60481V2_SourceDataForFigure 4/Figure 4A/Figure 4A repeat 1.tif]

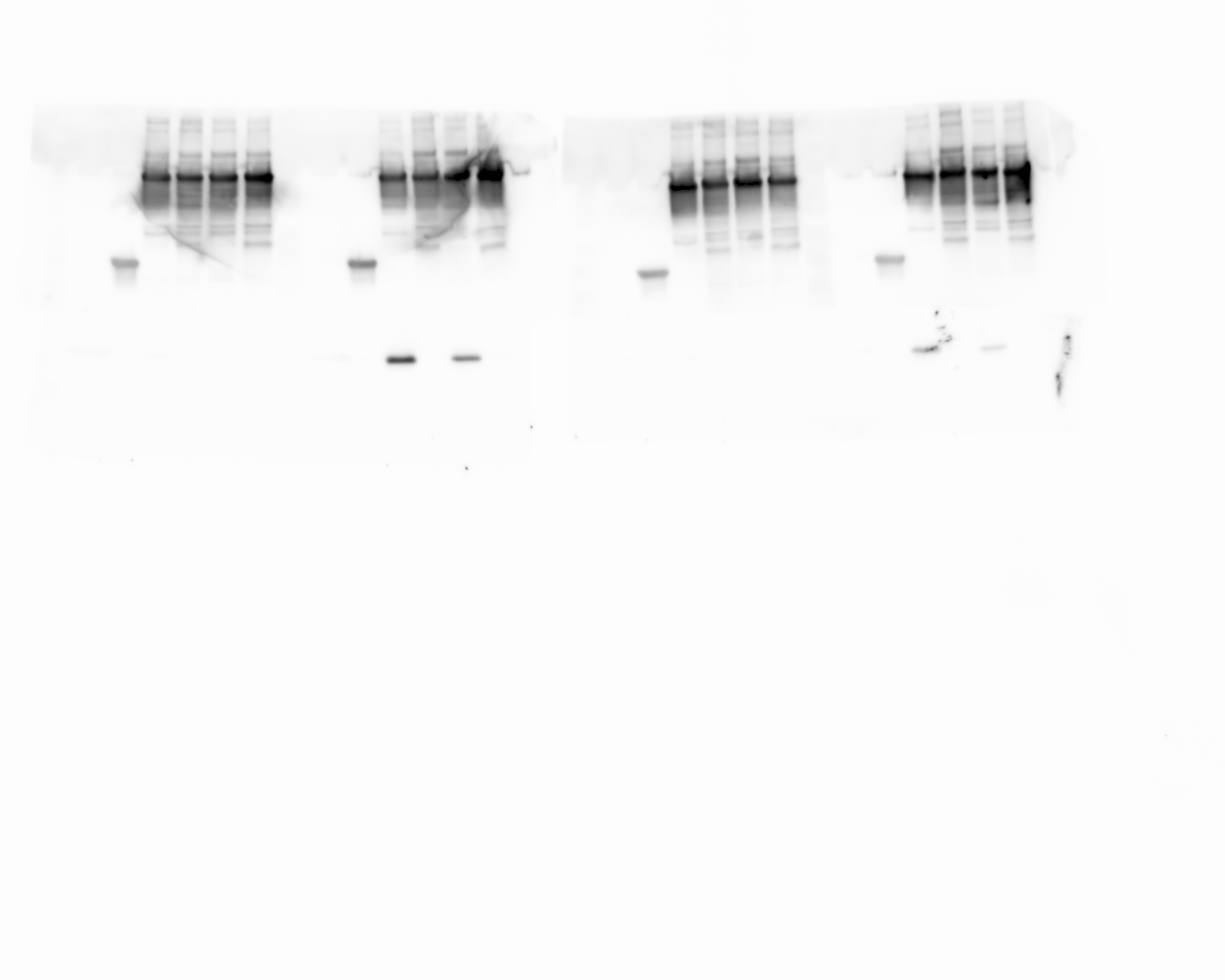

Supplement: Supplementary file 6 — Source data Fig. 4 [file 44319_2024_306_MOESM6_ESM.zip › EMBOR-2024-60481V2_SourceDataForFigure 4/Figure 4F/USER2 2023-10-18 15h55m06s WT me2 histones.tif]

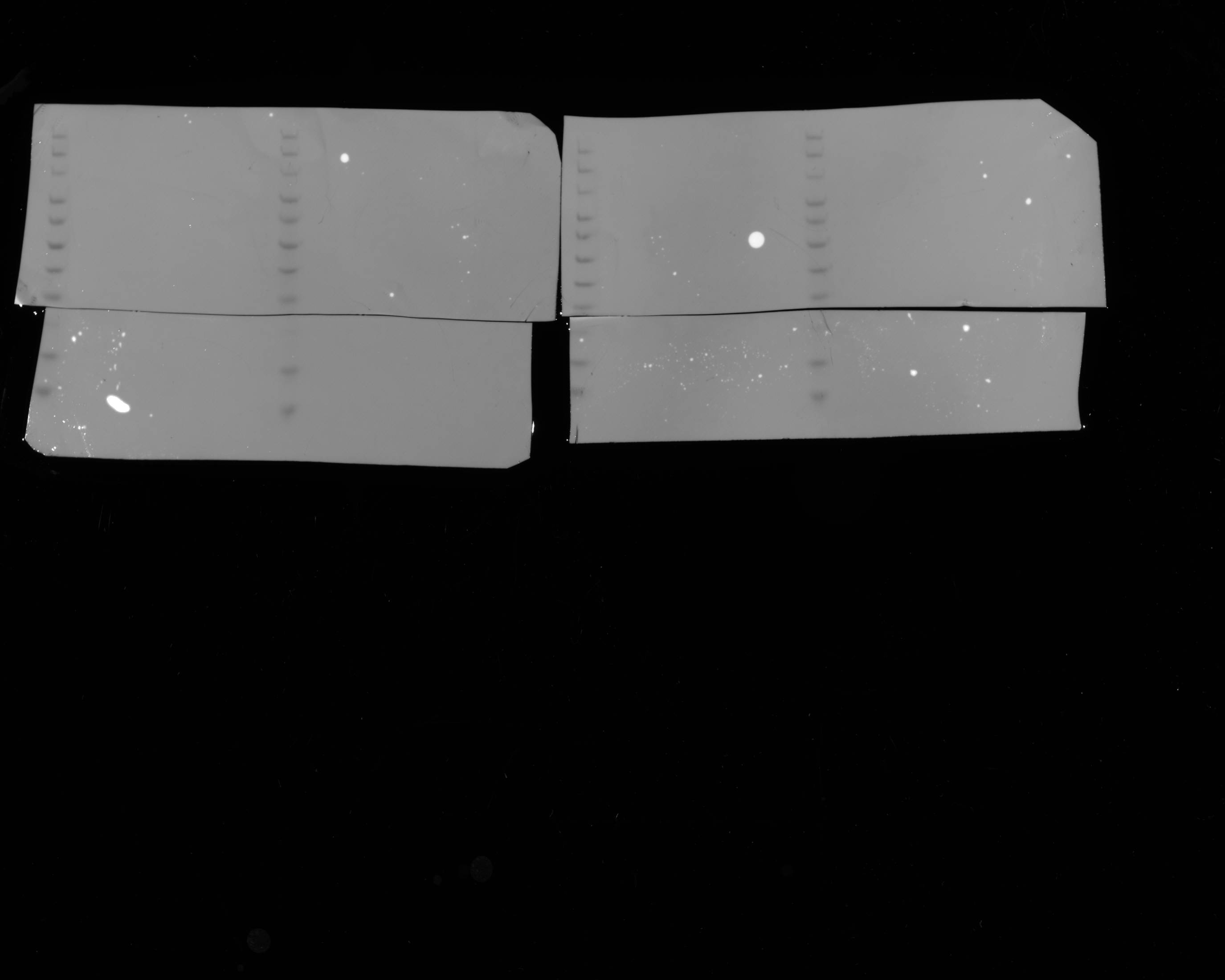

Supplement: Supplementary file 6 — Source data Fig. 4 [file 44319_2024_306_MOESM6_ESM.zip › EMBOR-2024-60481V2_SourceDataForFigure 4/Figure 4F/USER2 2023-10-18 15h50m48s WT me2 colorimetric.tif]

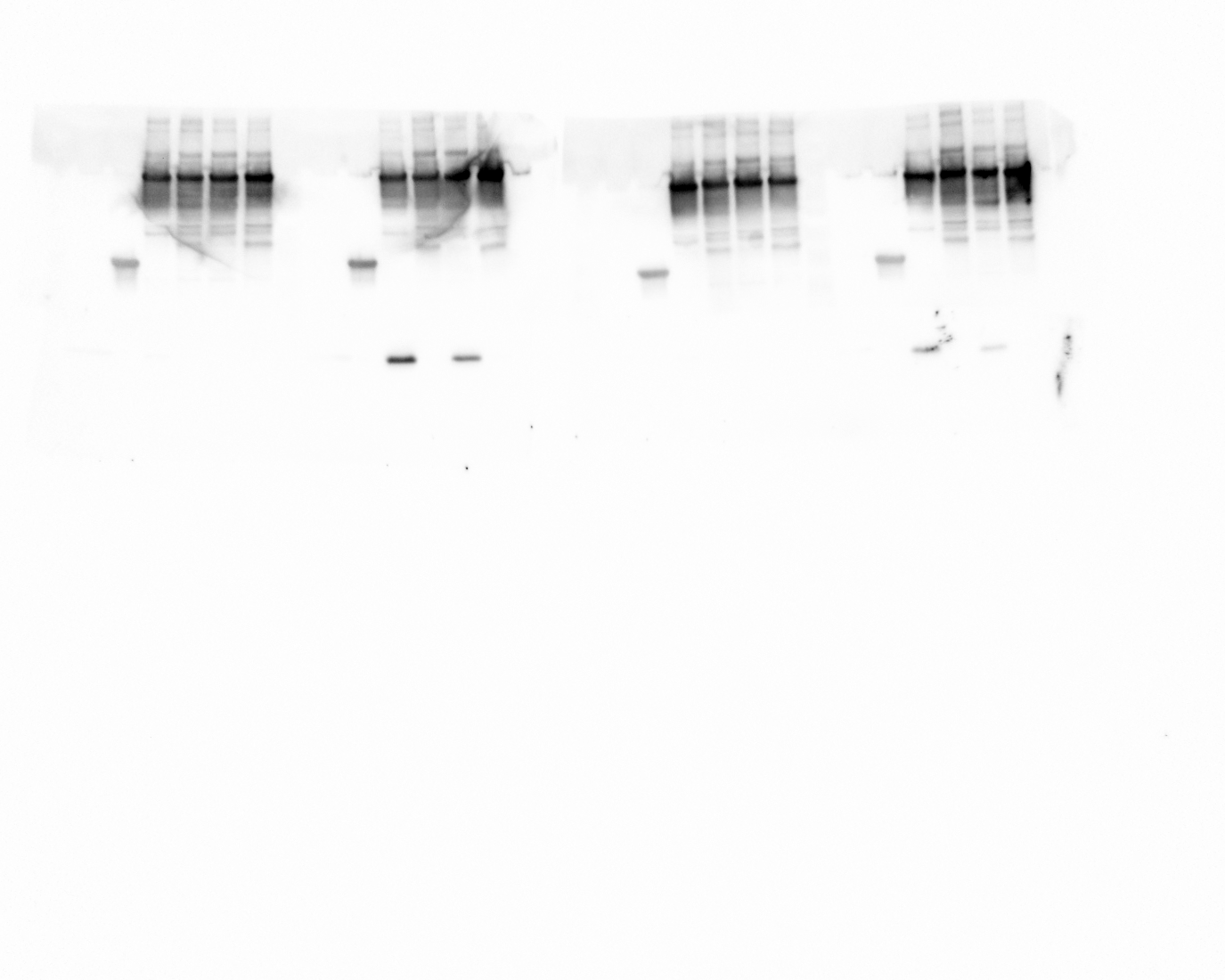

Supplement: Supplementary file 6 — Source data Fig. 4 [file 44319_2024_306_MOESM6_ESM.zip › EMBOR-2024-60481V2_SourceDataForFigure 4/Figure 4F/USER2 2023-10-18 15h51m21s WT me2 MBP.tif]

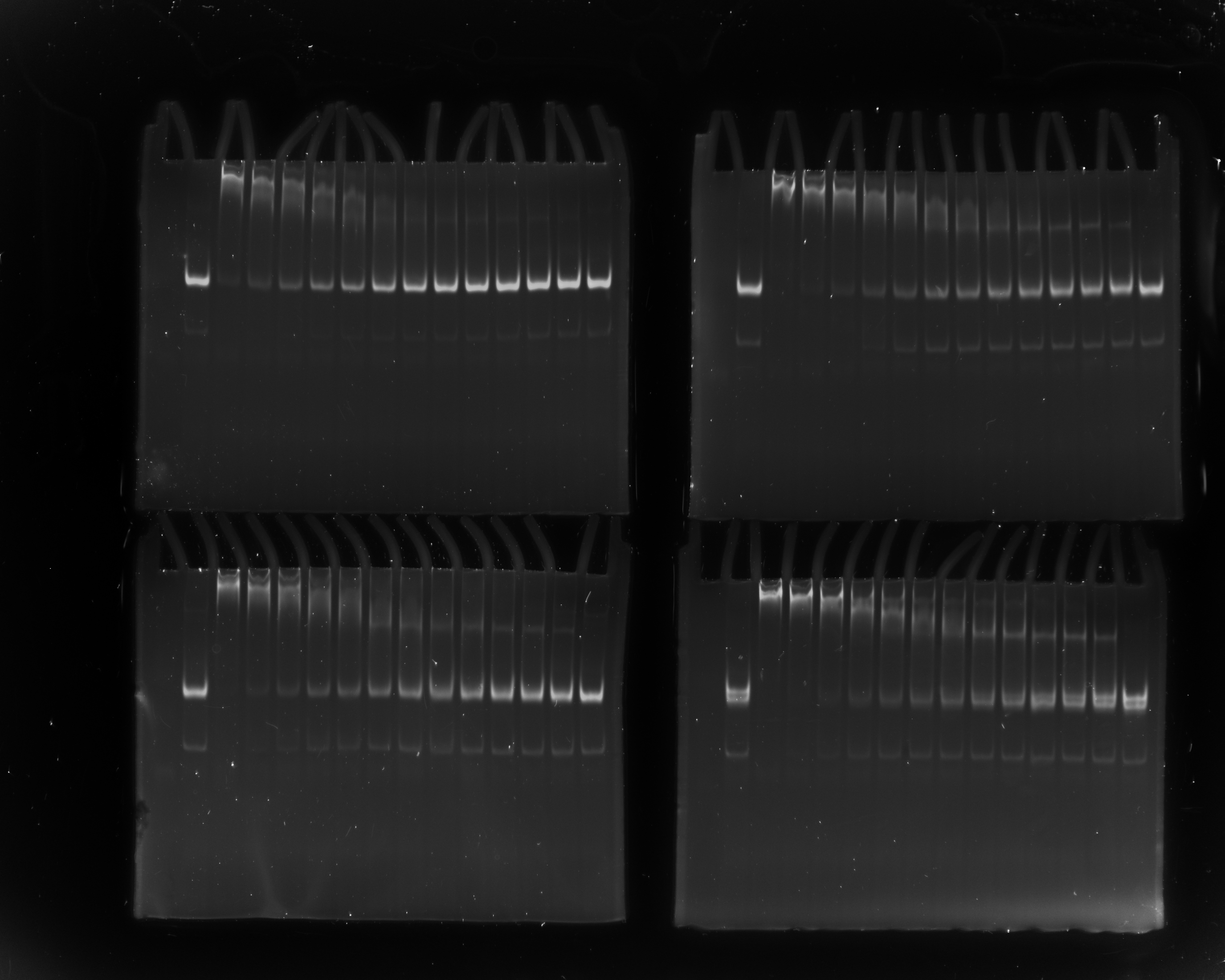

Supplement: Supplementary file 7 — Source data Fig. 5 [file 44319_2024_306_MOESM7_ESM.zip › EMBOR-2024-60481V2_SourceDataForFigure 5/Figure 5A/Figure 5A.tif]

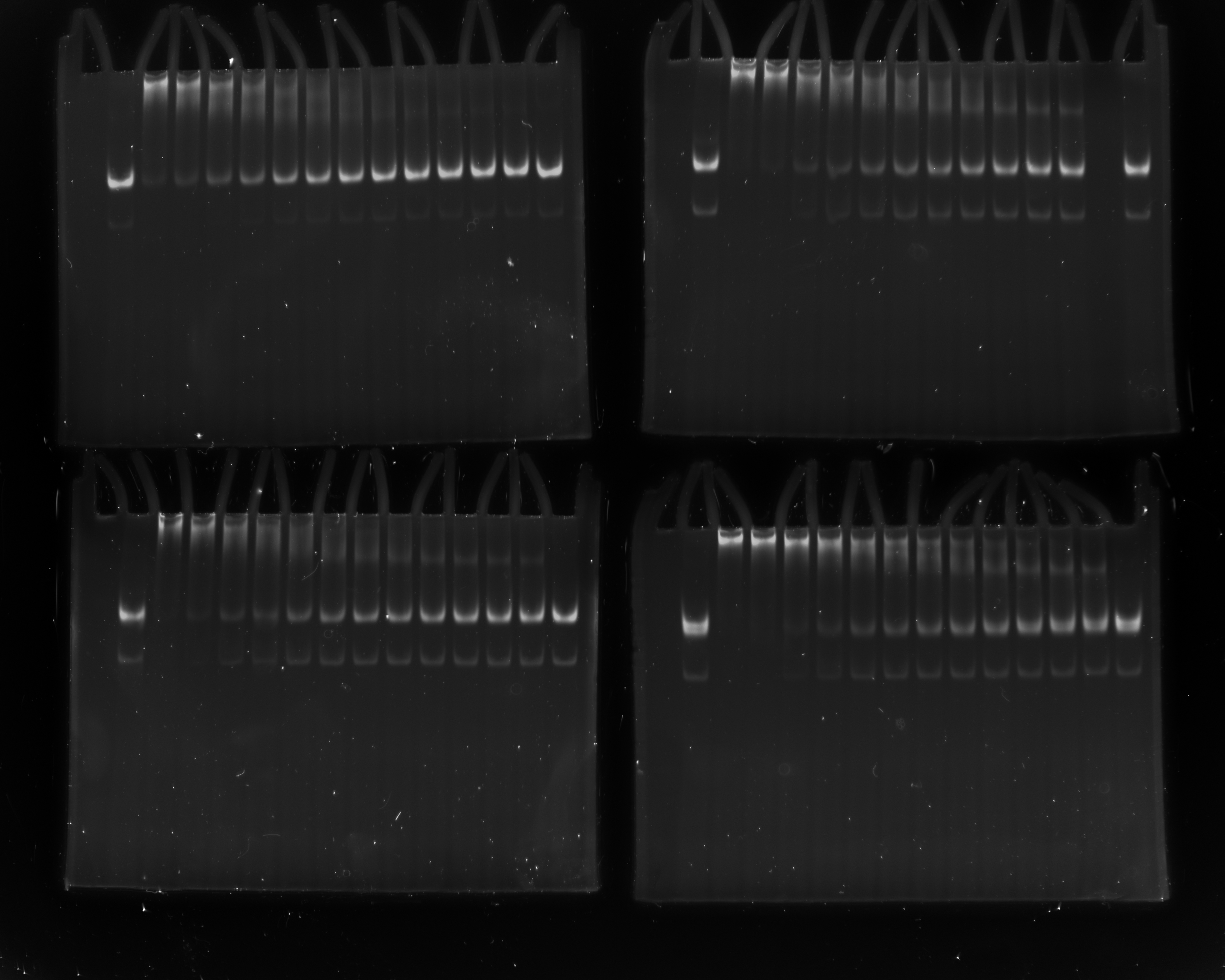

Supplement: Supplementary file 7 — Source data Fig. 5 [file 44319_2024_306_MOESM7_ESM.zip › EMBOR-2024-60481V2_SourceDataForFigure 5/Figure 5A/Figure 5A repeat.tif]

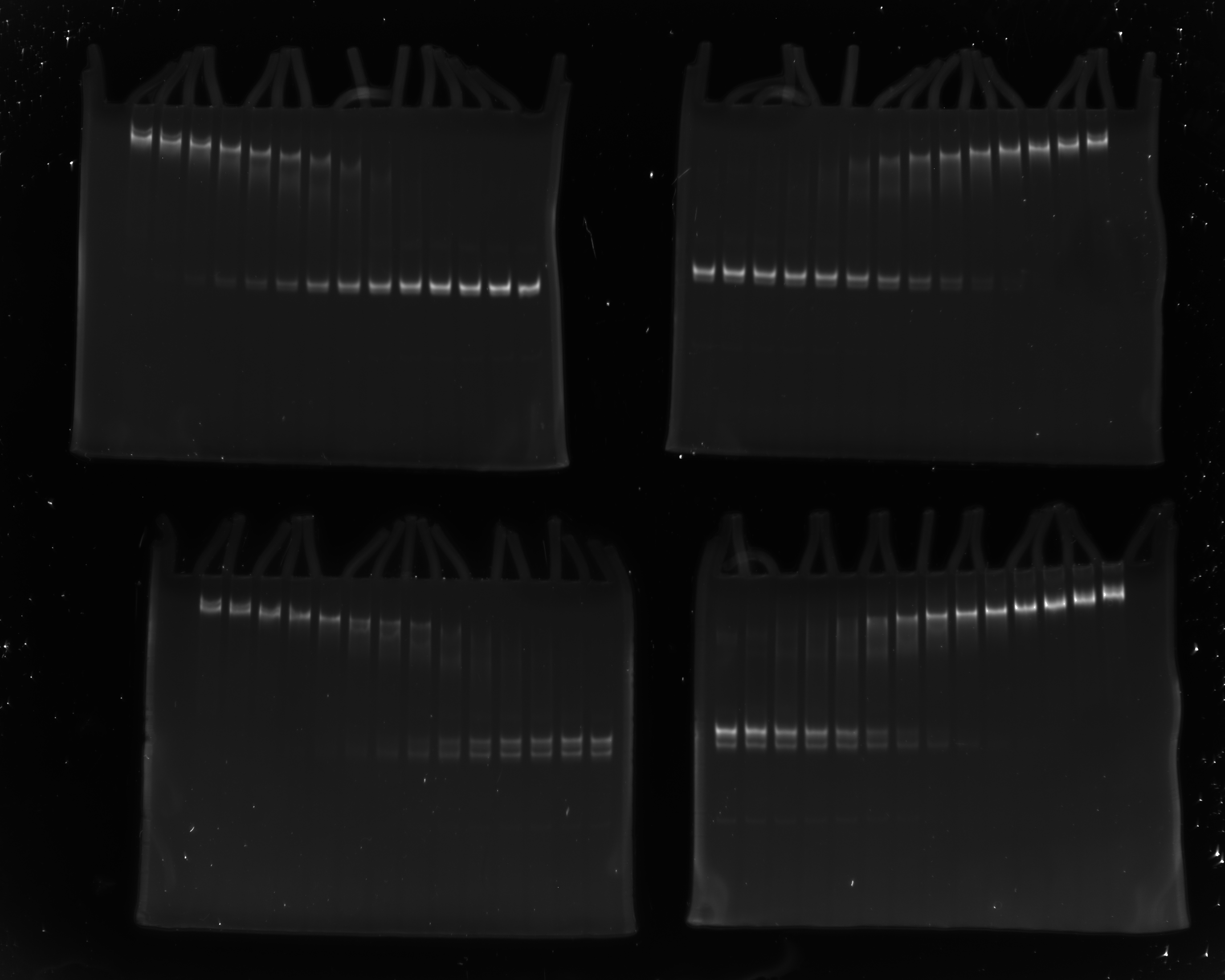

Supplement: Supplementary file 7 — Source data Fig. 5 [file 44319_2024_306_MOESM7_ESM.zip › EMBOR-2024-60481V2_SourceDataForFigure 5/Figure 5C/Figure 5C.tif]

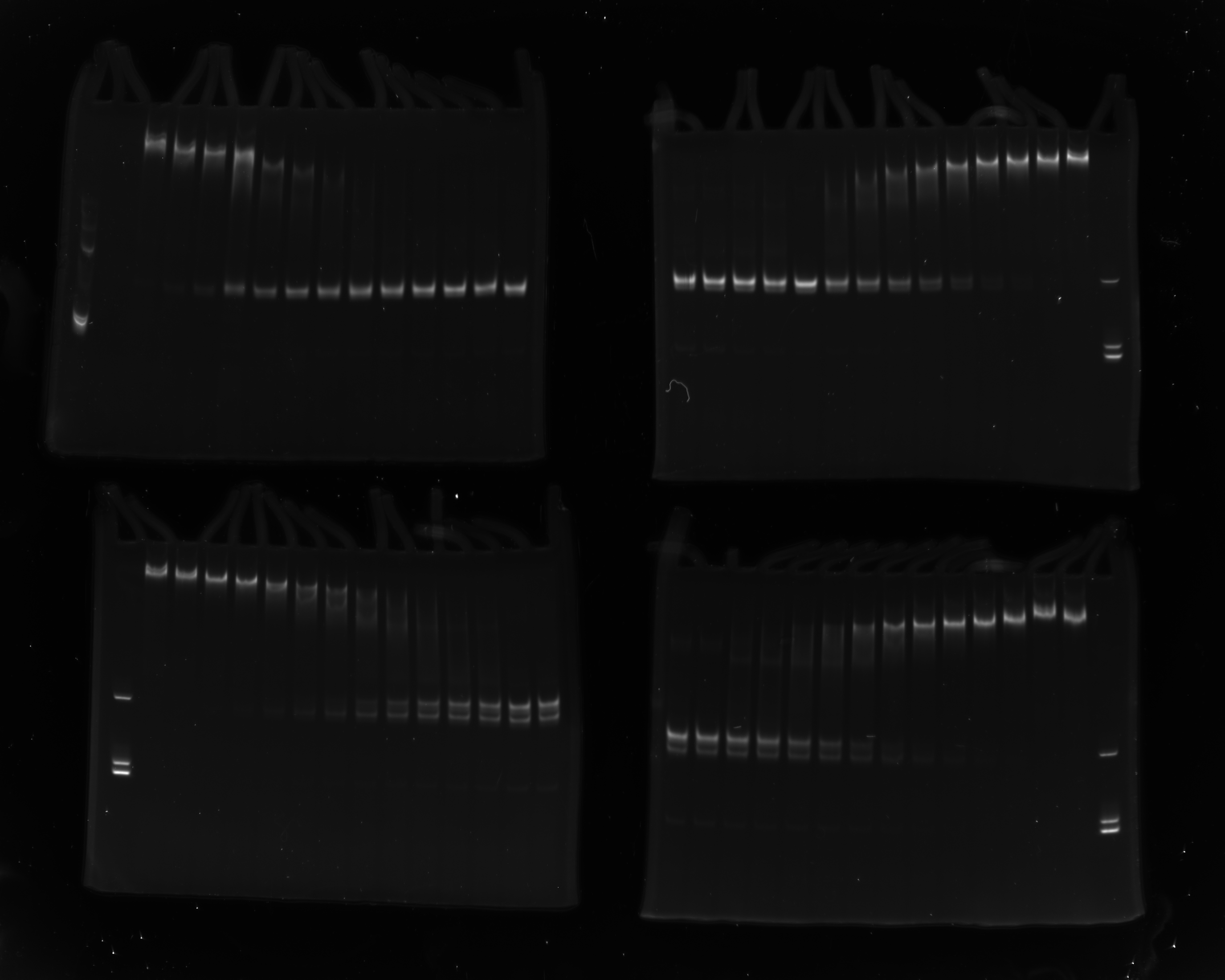

Supplement: Supplementary file 7 — Source data Fig. 5 [file 44319_2024_306_MOESM7_ESM.zip › EMBOR-2024-60481V2_SourceDataForFigure 5/Figure 5C/Figure 5C repeat 4.tif]

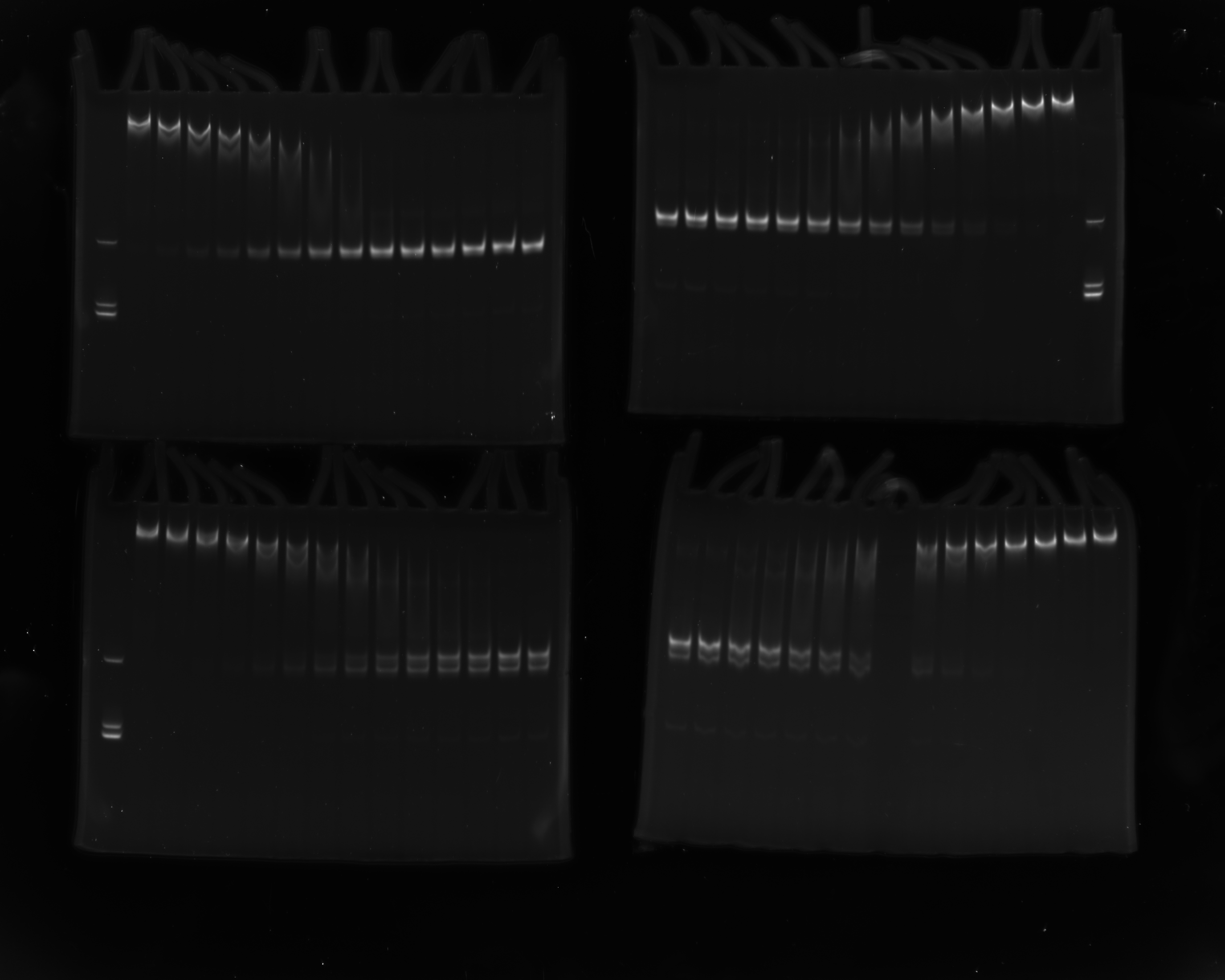

Supplement: Supplementary file 7 — Source data Fig. 5 [file 44319_2024_306_MOESM7_ESM.zip › EMBOR-2024-60481V2_SourceDataForFigure 5/Figure 5C/Figure 5C repeat 5.tif]

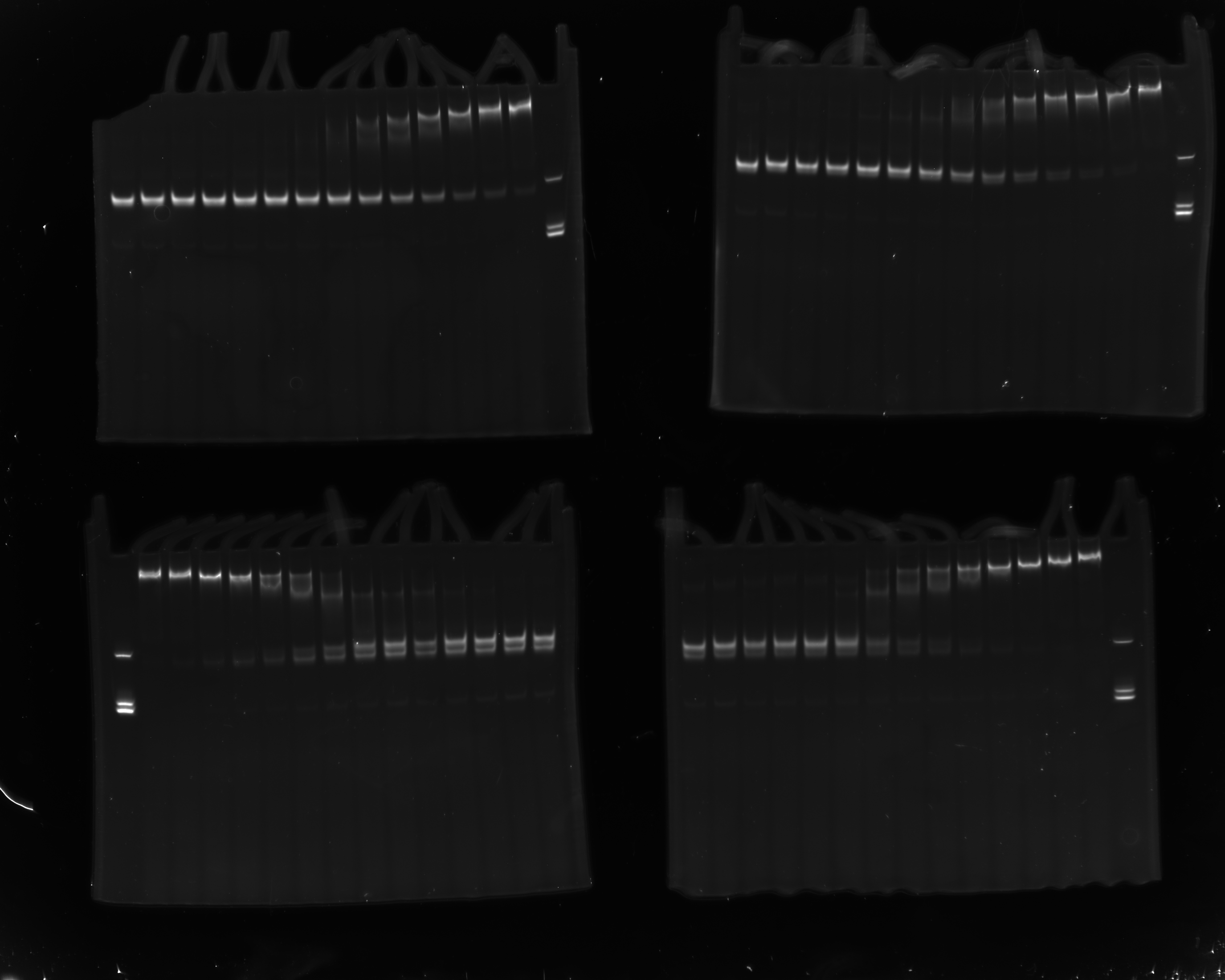

Supplement: Supplementary file 7 — Source data Fig. 5 [file 44319_2024_306_MOESM7_ESM.zip › EMBOR-2024-60481V2_SourceDataForFigure 5/Figure 5C/Figure 5C repeat 2.tif]

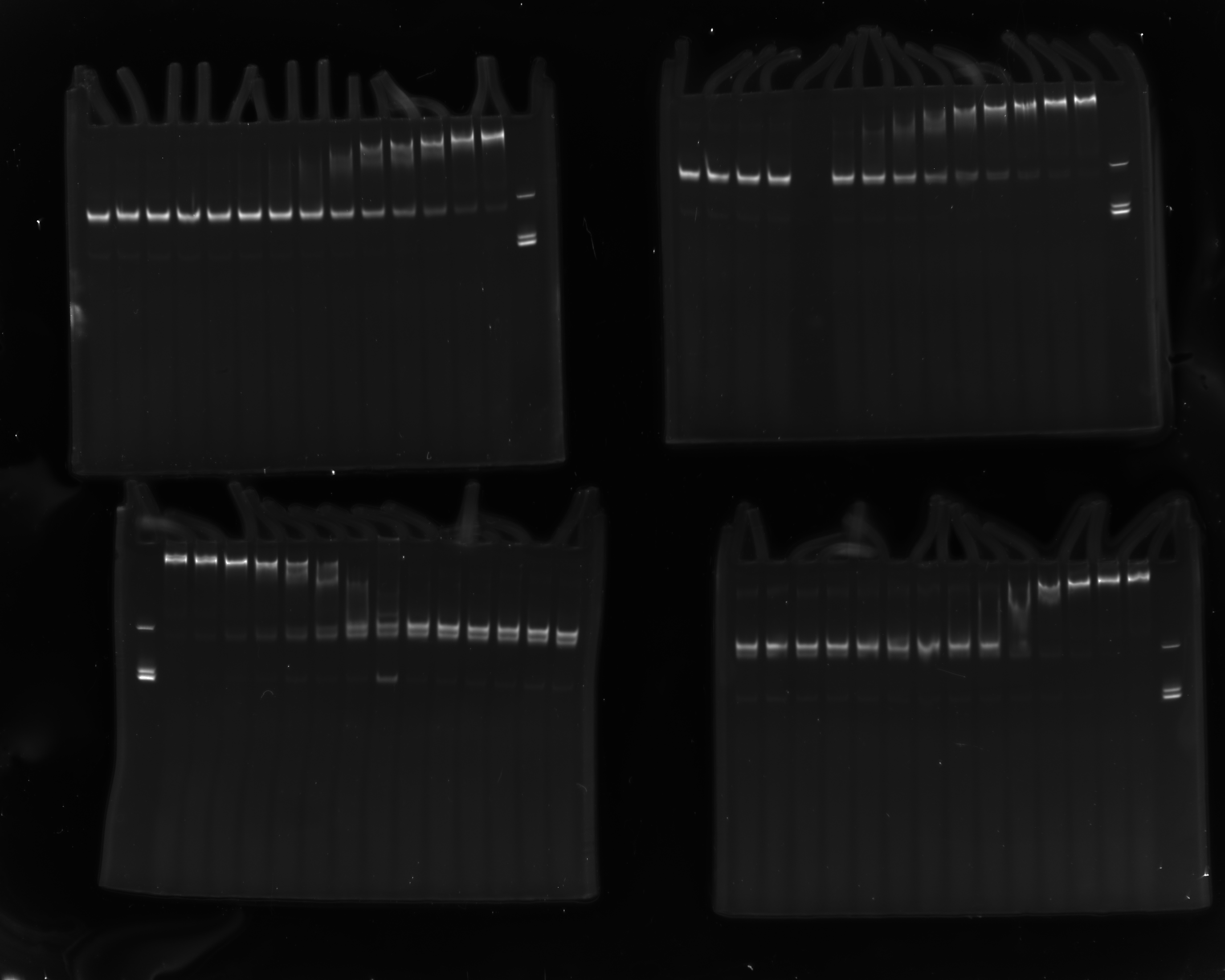

Supplement: Supplementary file 7 — Source data Fig. 5 [file 44319_2024_306_MOESM7_ESM.zip › EMBOR-2024-60481V2_SourceDataForFigure 5/Figure 5C/Figure 5C repeat 3.tif]

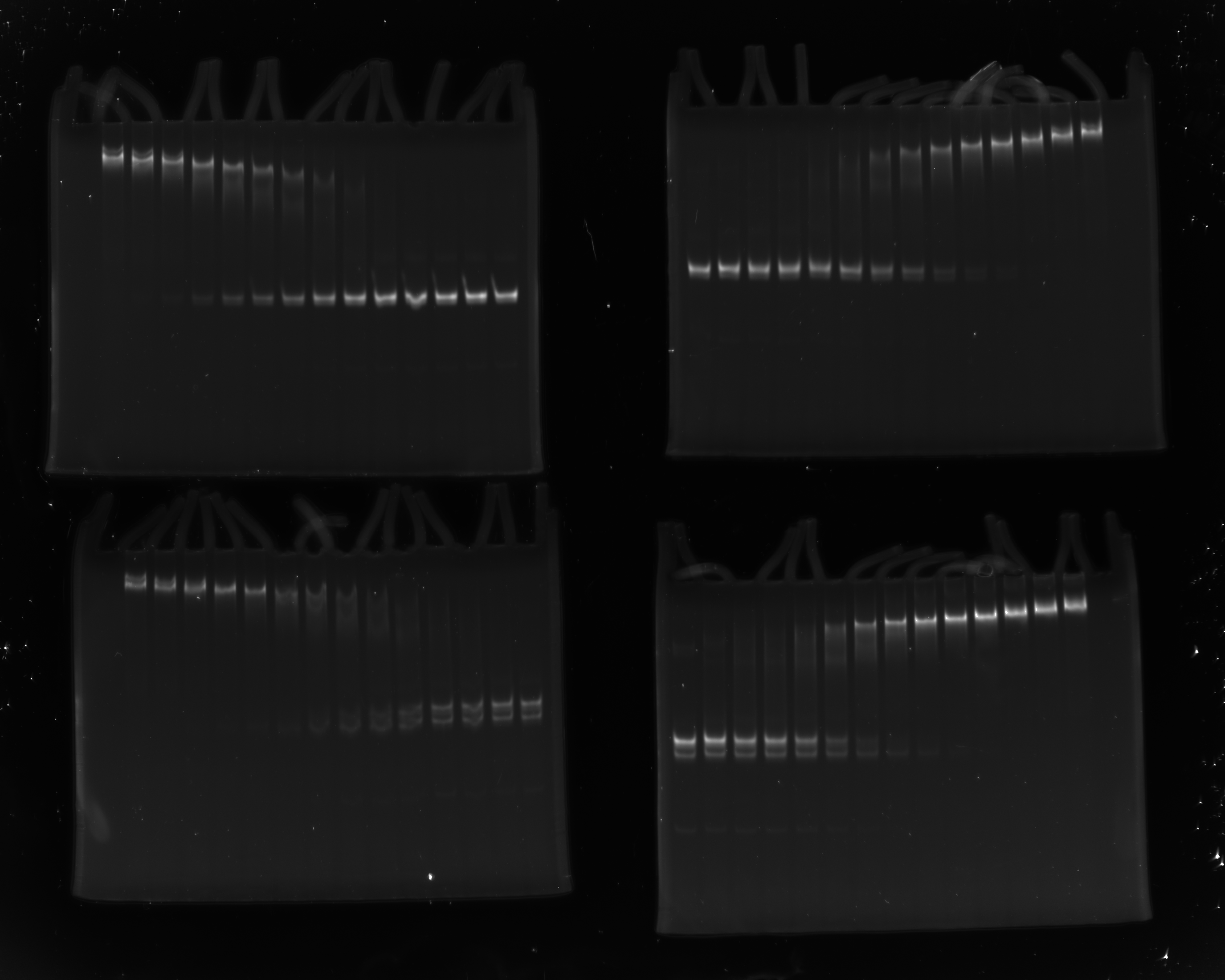

Supplement: Supplementary file 7 — Source data Fig. 5 [file 44319_2024_306_MOESM7_ESM.zip › EMBOR-2024-60481V2_SourceDataForFigure 5/Figure 5C/Figure 5C repeat 1.tif]

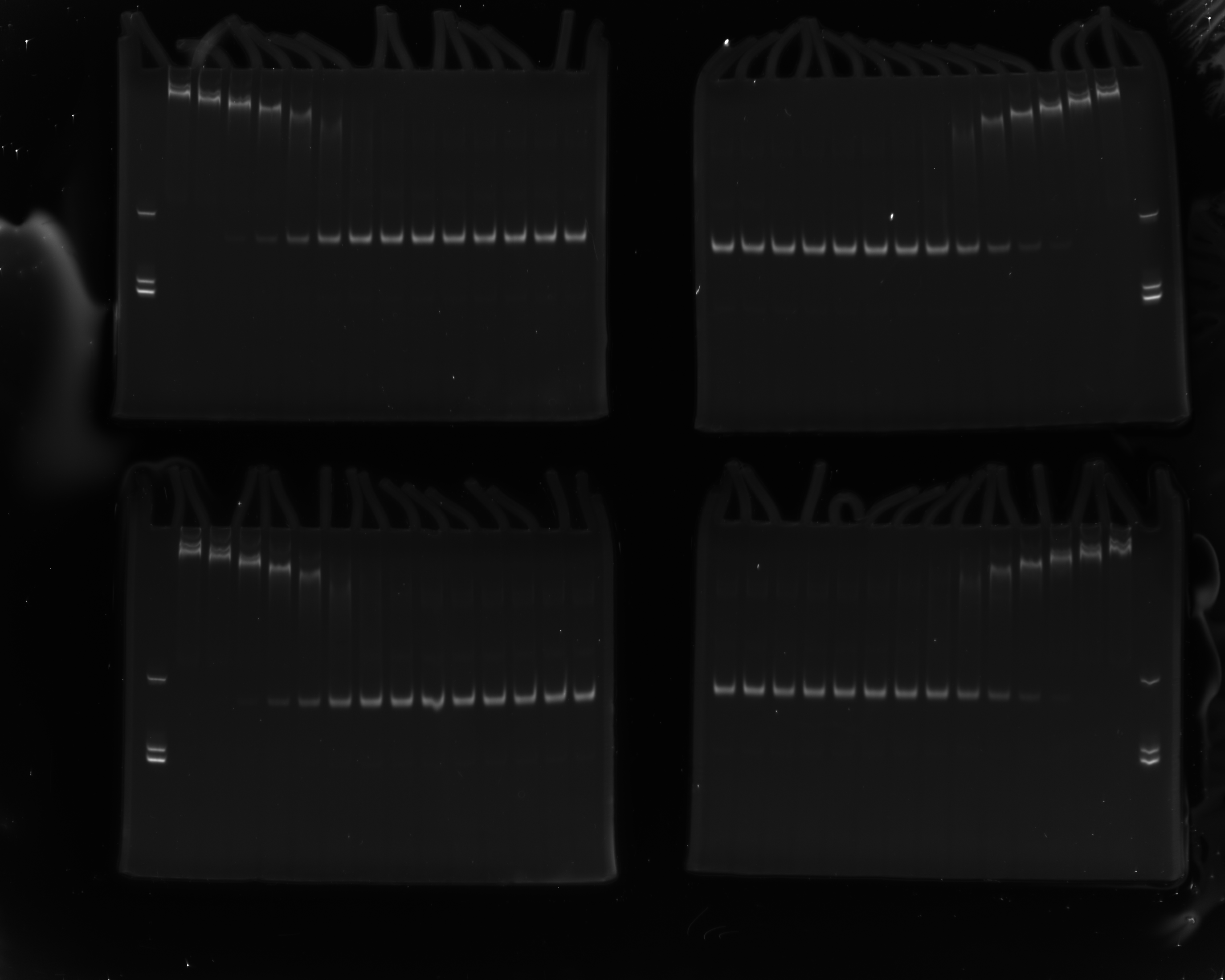

Supplement: Supplementary file 7 — Source data Fig. 5 [file 44319_2024_306_MOESM7_ESM.zip › EMBOR-2024-60481V2_SourceDataForFigure 5/Figure 5B/Figure 5B and repeat.tif]

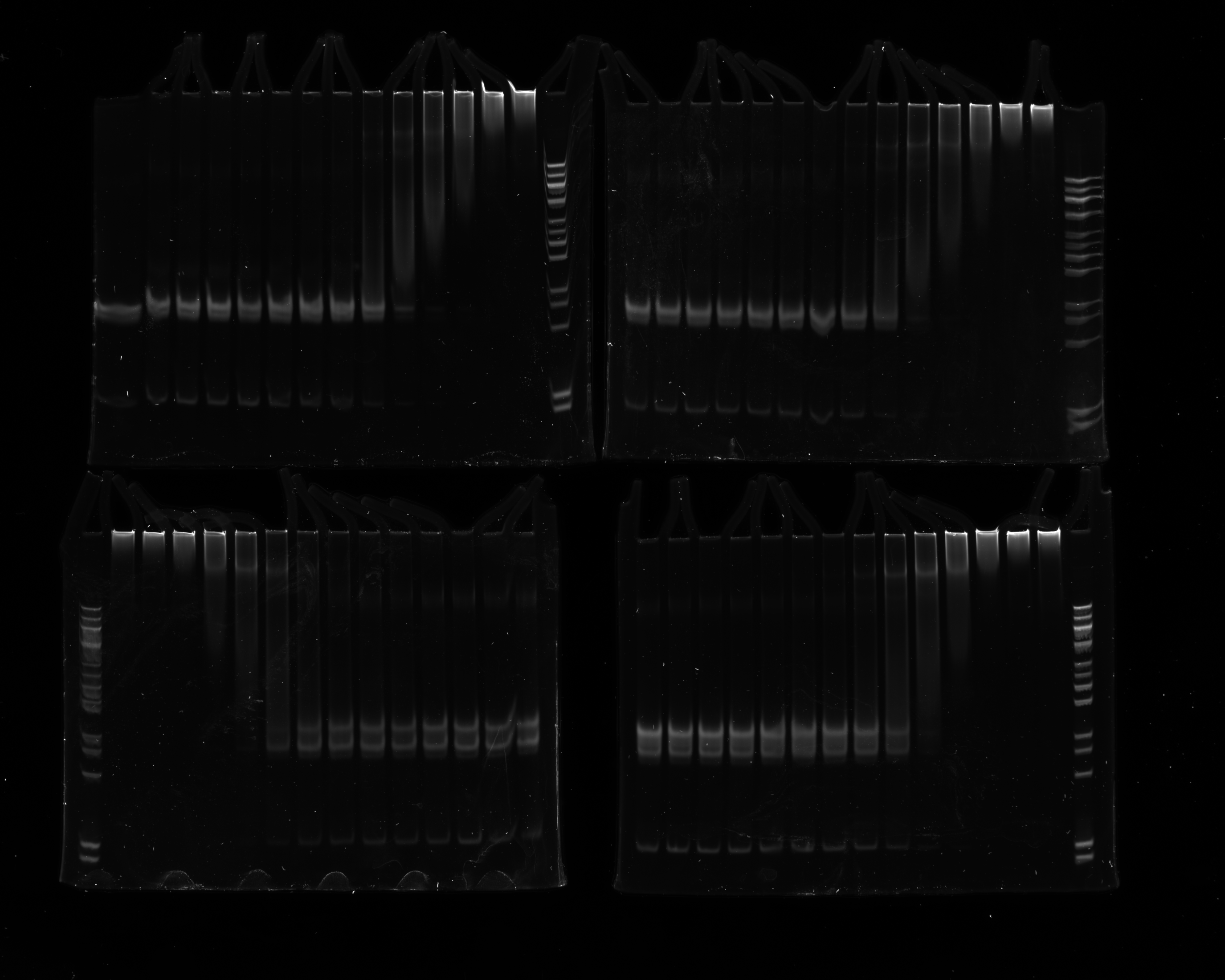

Supplement: Supplementary file 8 — Source data Fig. 6 [file 44319_2024_306_MOESM8_ESM.zip › EMBOR-2024-60481V2_SourceDataForFigure 6/Figure 6D/Figure 6D repeat.tif]

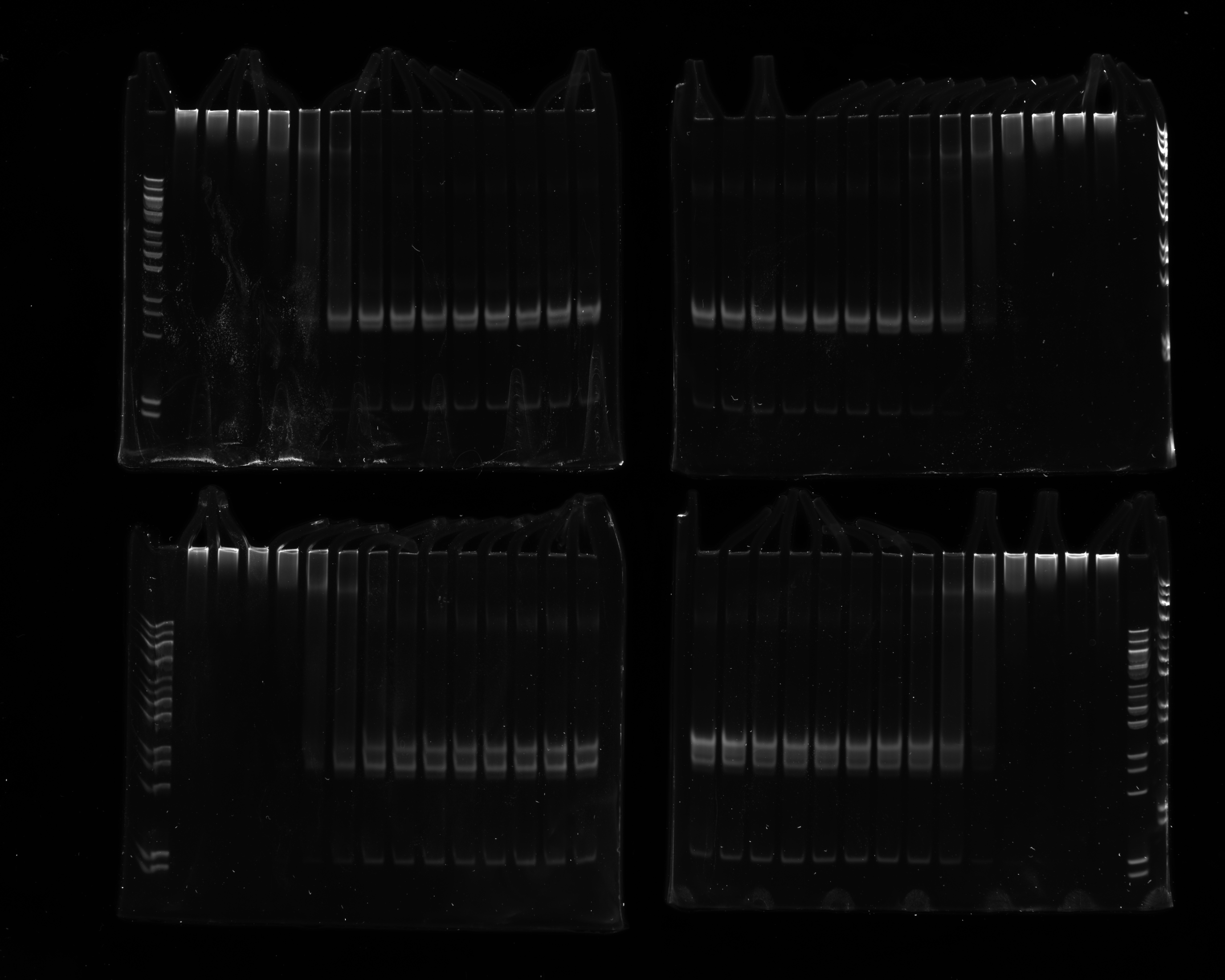

Supplement: Supplementary file 8 — Source data Fig. 6 [file 44319_2024_306_MOESM8_ESM.zip › EMBOR-2024-60481V2_SourceDataForFigure 6/Figure 6D/Figure 6D.tif]

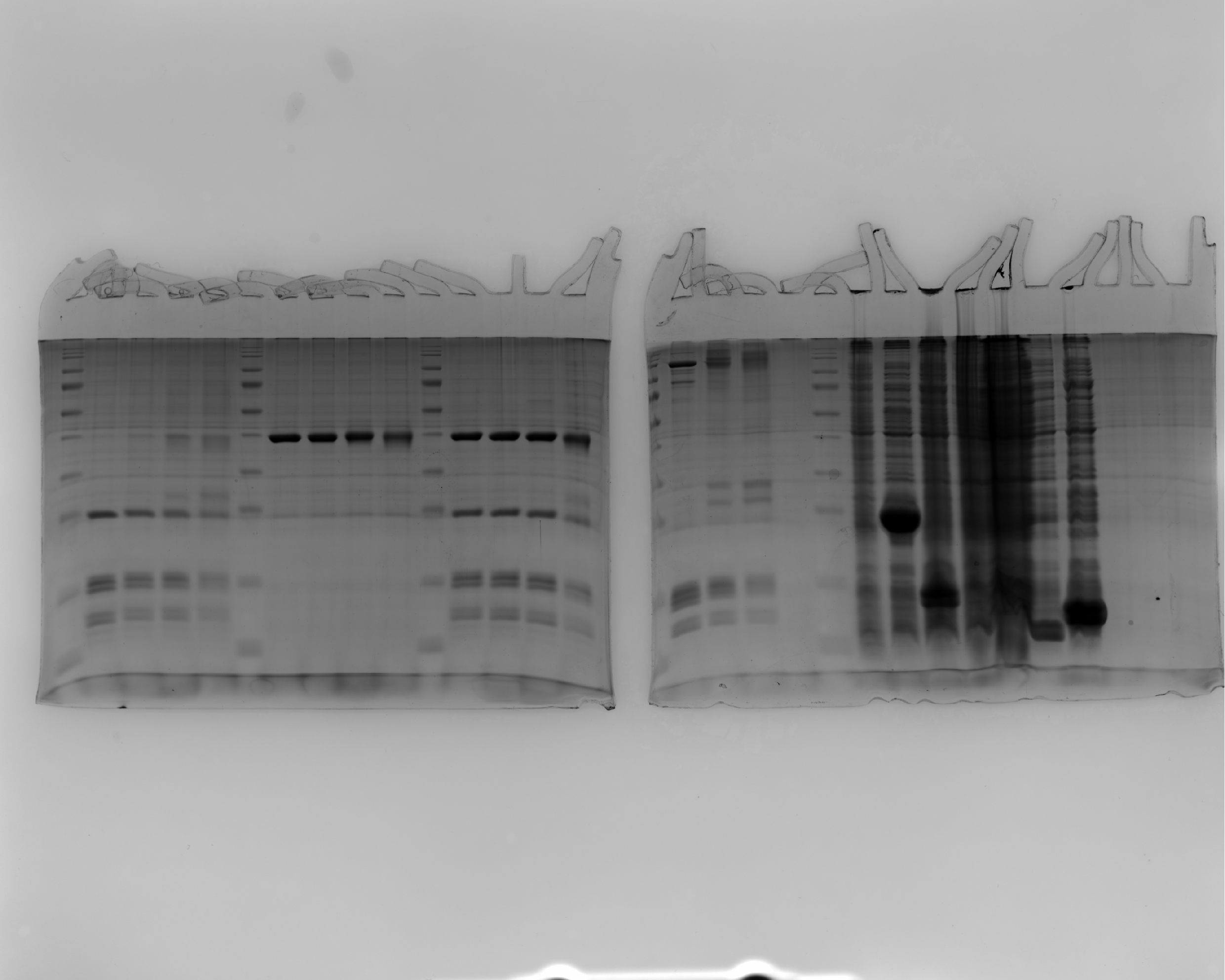

Supplement: Supplementary file 9 — Figure Source Data appendix [file 44319_2024_306_MOESM9_ESM.zip › EMBOR-2024-60481V2_SourceDataFor_appendix/EMBOR-2024-60481V2_SourceDataForAppendix fig S6/S6A/S6A.tif]

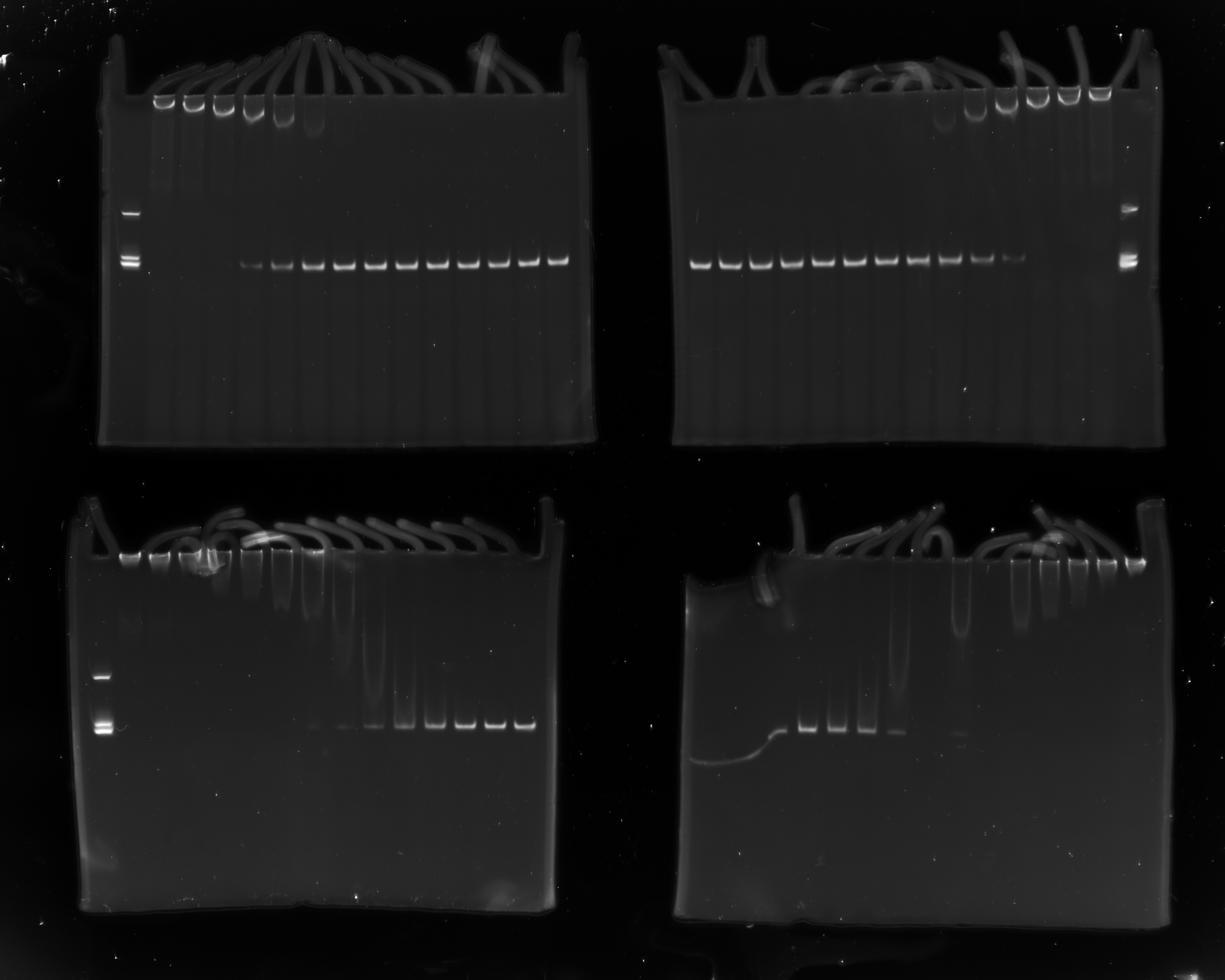

Supplement: Supplementary file 9 — Figure Source Data appendix [file 44319_2024_306_MOESM9_ESM.zip › EMBOR-2024-60481V2_SourceDataFor_appendix/EMBOR-2024-60481V2_SourceDataForAppendix fig S3/S3C/S3C repeat1.tif]

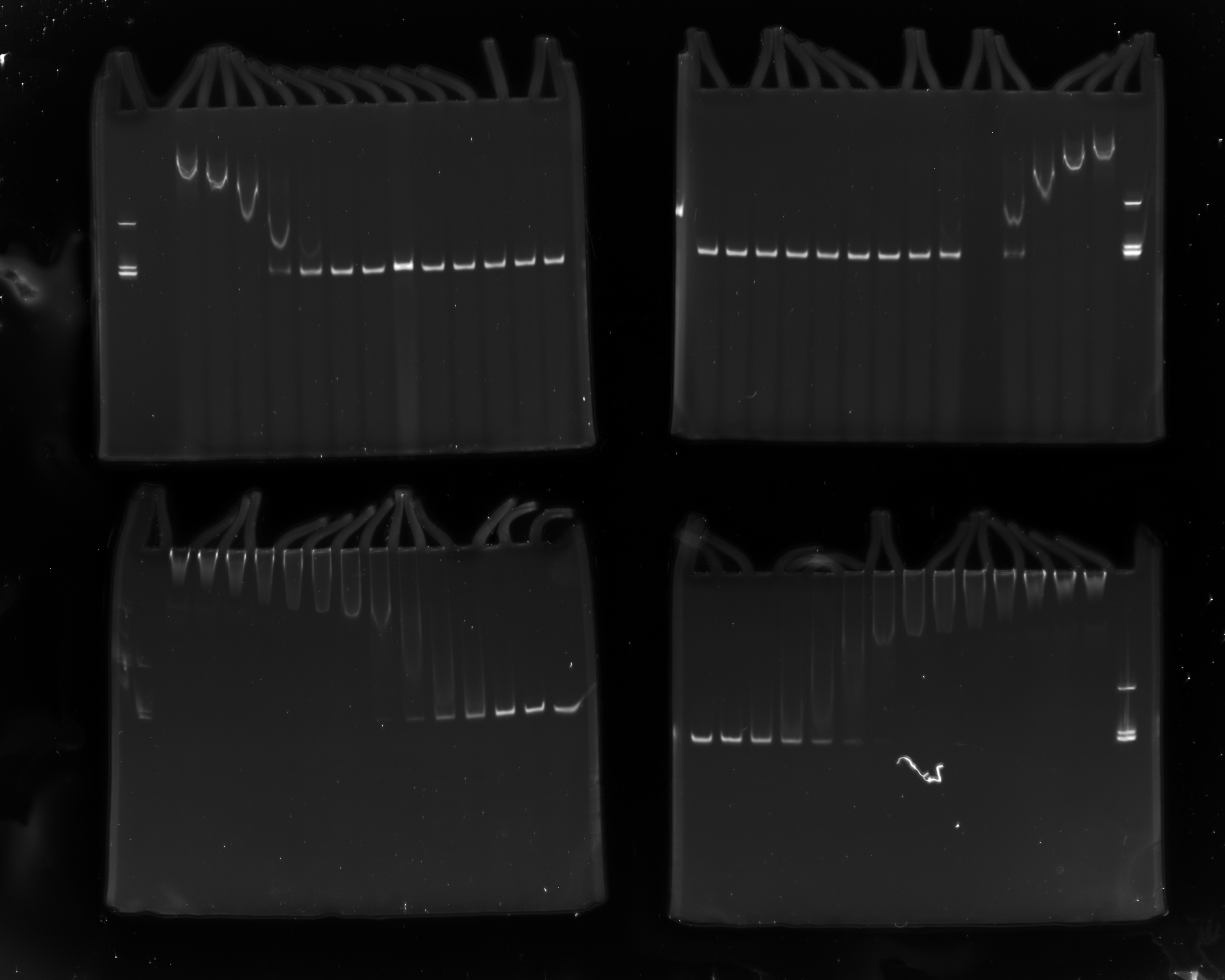

Supplement: Supplementary file 9 — Figure Source Data appendix [file 44319_2024_306_MOESM9_ESM.zip › EMBOR-2024-60481V2_SourceDataFor_appendix/EMBOR-2024-60481V2_SourceDataForAppendix fig S3/S3C/S3C.tif]

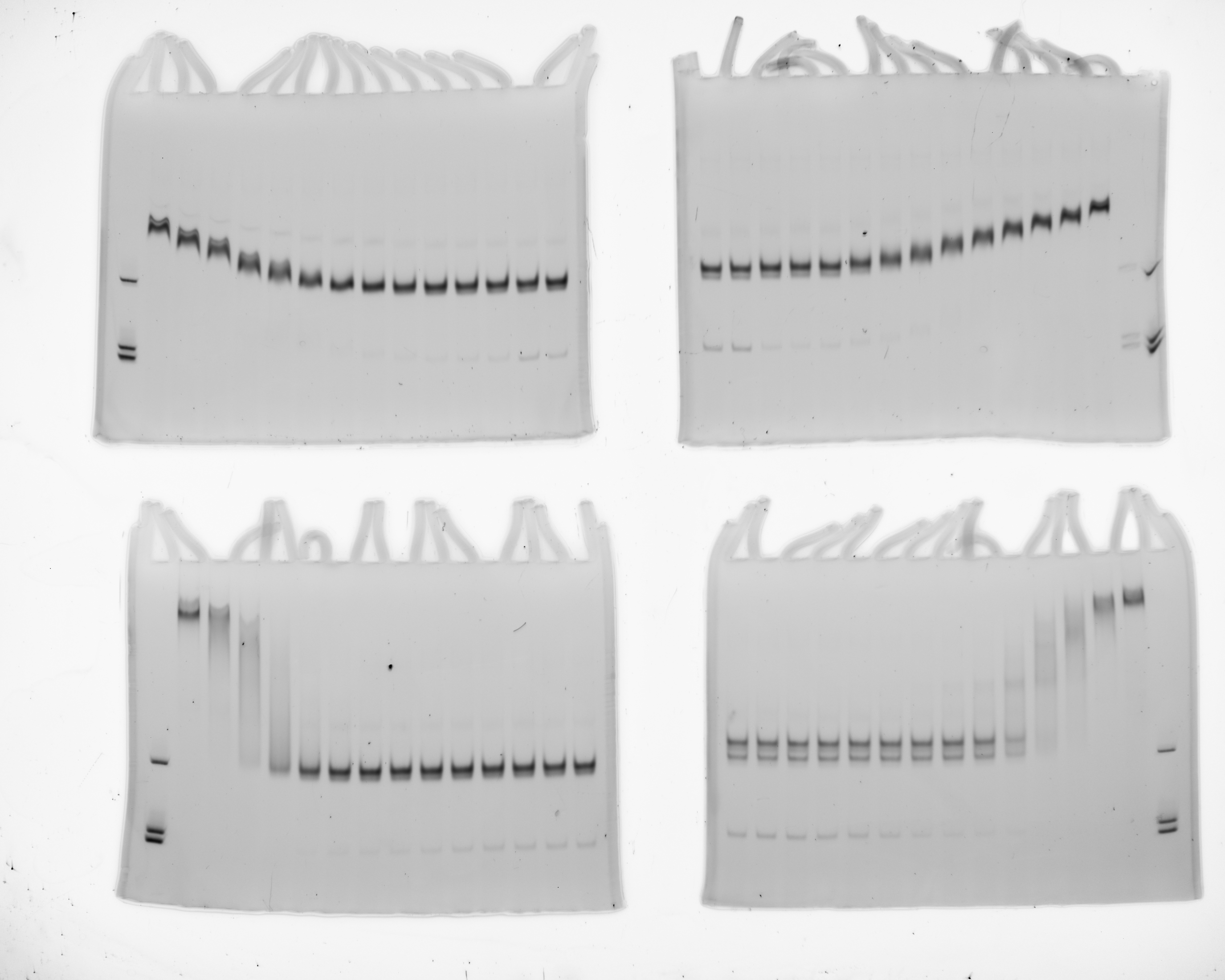

Supplement: Supplementary file 9 — Figure Source Data appendix [file 44319_2024_306_MOESM9_ESM.zip › EMBOR-2024-60481V2_SourceDataFor_appendix/EMBOR-2024-60481V2_SourceDataForAppendix fig S3/S3D/USER2 2024-05-09 16h09m16s analysis.tif]

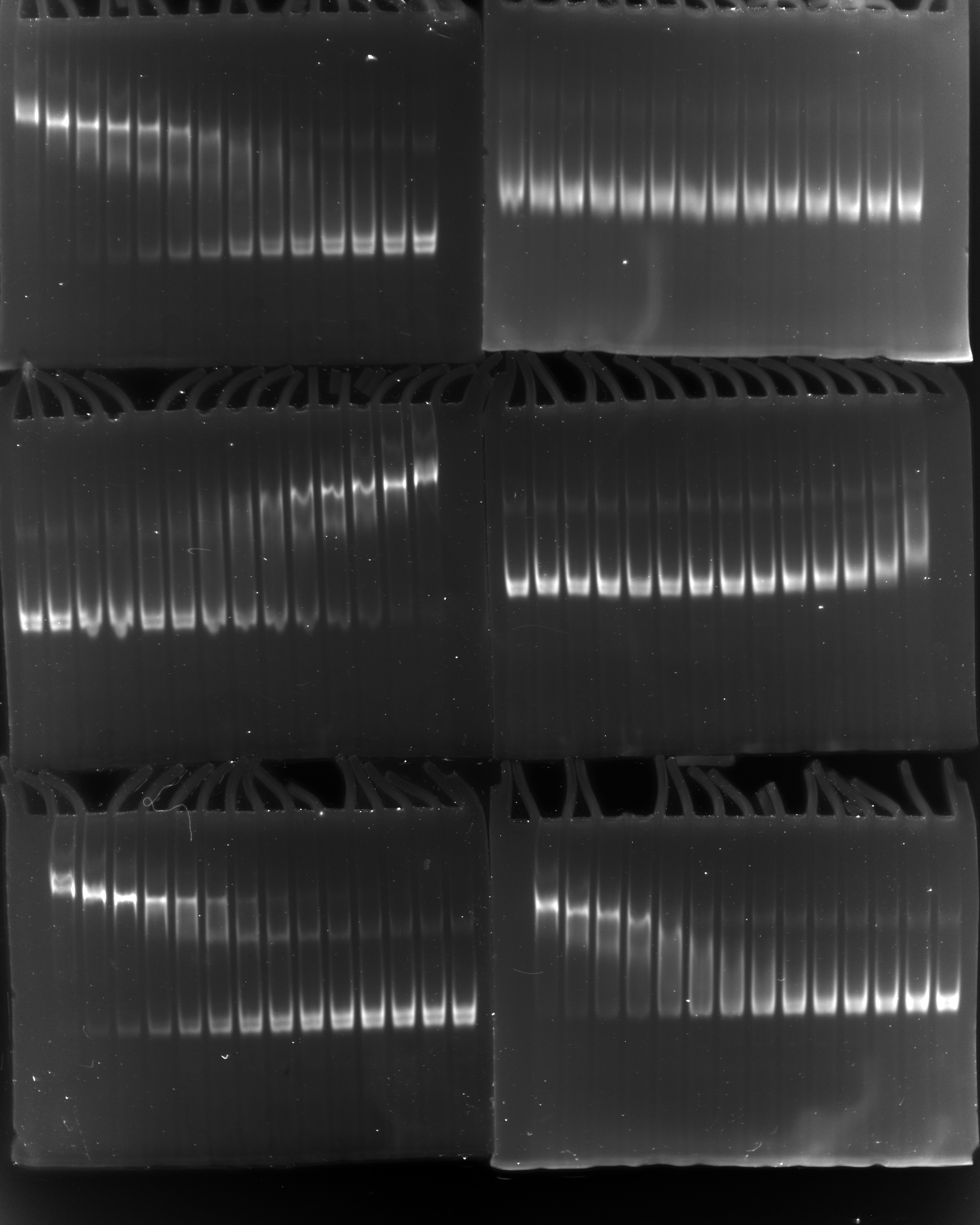

Supplement: Supplementary file 9 — Figure Source Data appendix [file 44319_2024_306_MOESM9_ESM.zip › EMBOR-2024-60481V2_SourceDataFor_appendix/EMBOR-2024-60481V2_SourceDataForAppendix fig S3/S3F/S3F repeat 2.tif]

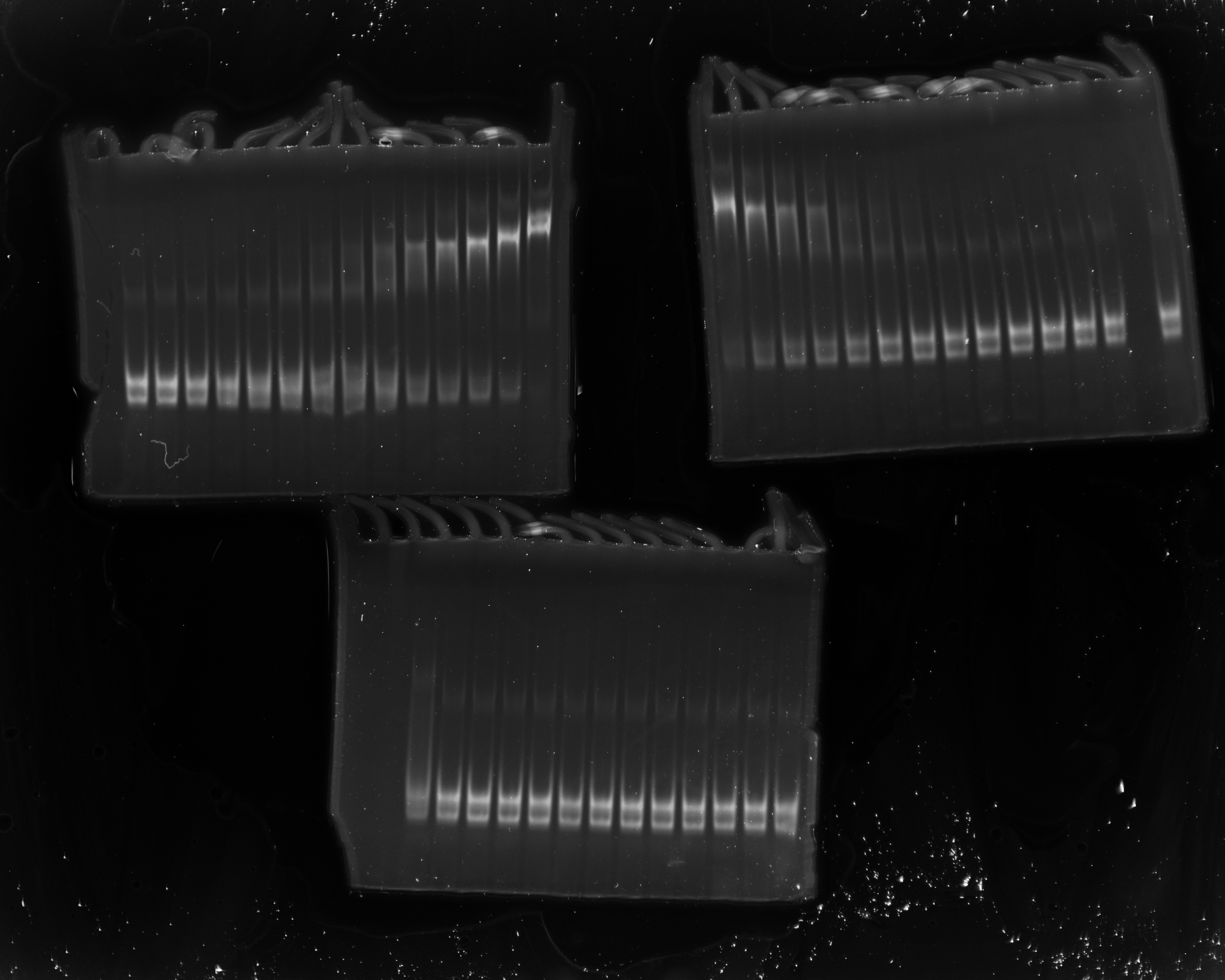

Supplement: Supplementary file 9 — Figure Source Data appendix [file 44319_2024_306_MOESM9_ESM.zip › EMBOR-2024-60481V2_SourceDataFor_appendix/EMBOR-2024-60481V2_SourceDataForAppendix fig S3/S3F/S3F repeat 1.1.tif]

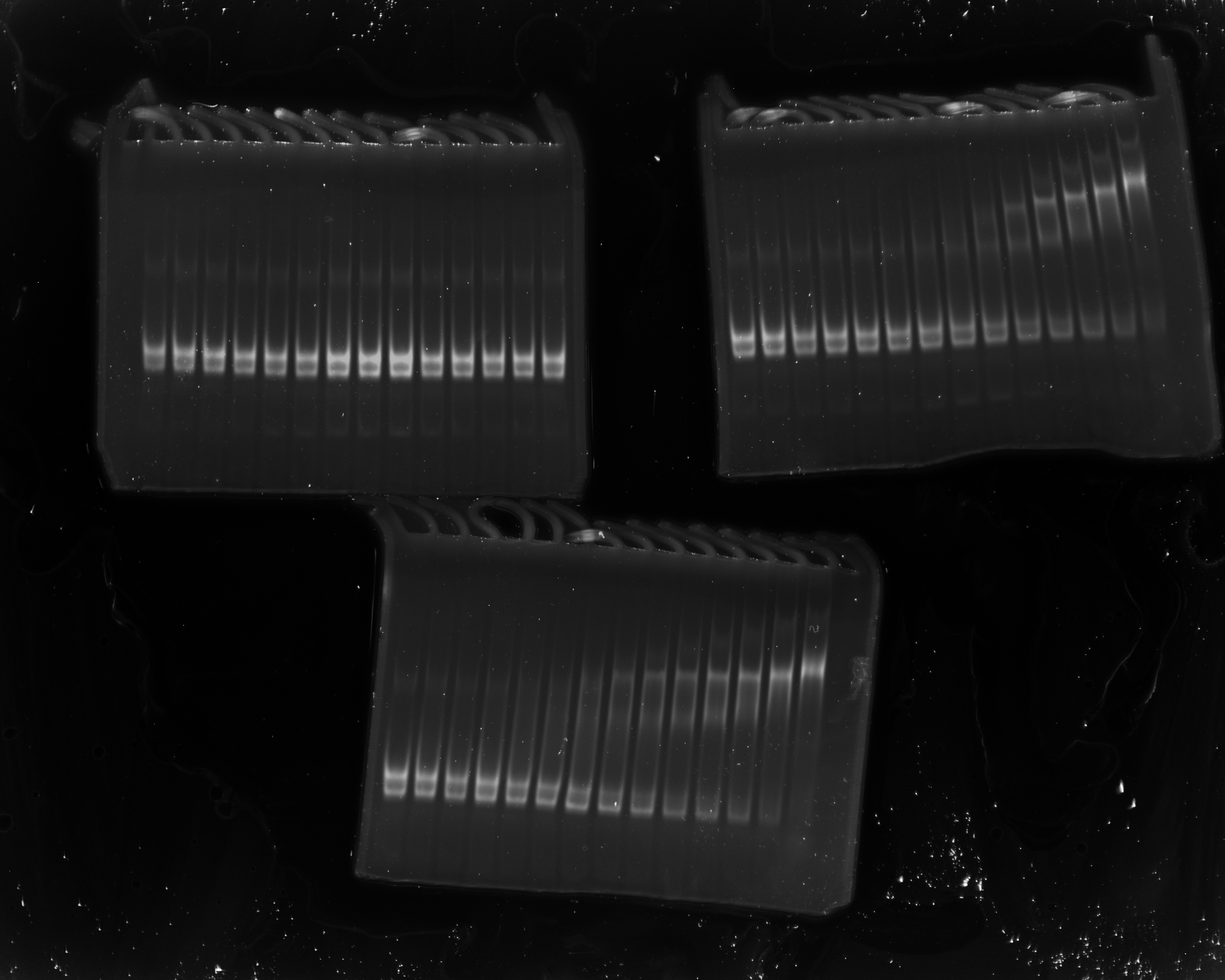

Supplement: Supplementary file 9 — Figure Source Data appendix [file 44319_2024_306_MOESM9_ESM.zip › EMBOR-2024-60481V2_SourceDataFor_appendix/EMBOR-2024-60481V2_SourceDataForAppendix fig S3/S3F/S3F repeat 1.2.tif]

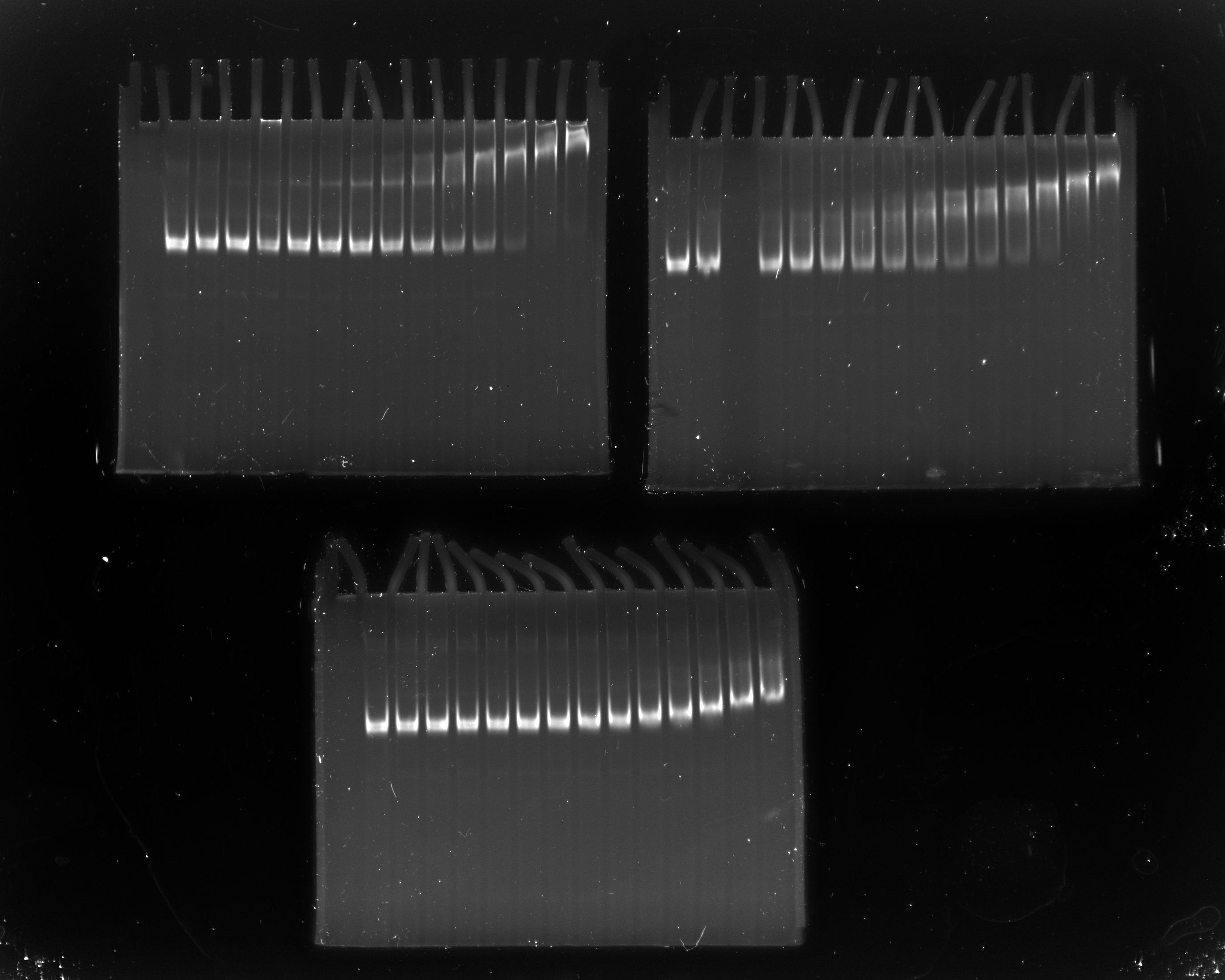

Supplement: Supplementary file 9 — Figure Source Data appendix [file 44319_2024_306_MOESM9_ESM.zip › EMBOR-2024-60481V2_SourceDataFor_appendix/EMBOR-2024-60481V2_SourceDataForAppendix fig S3/S3F/S3F 1.tif]

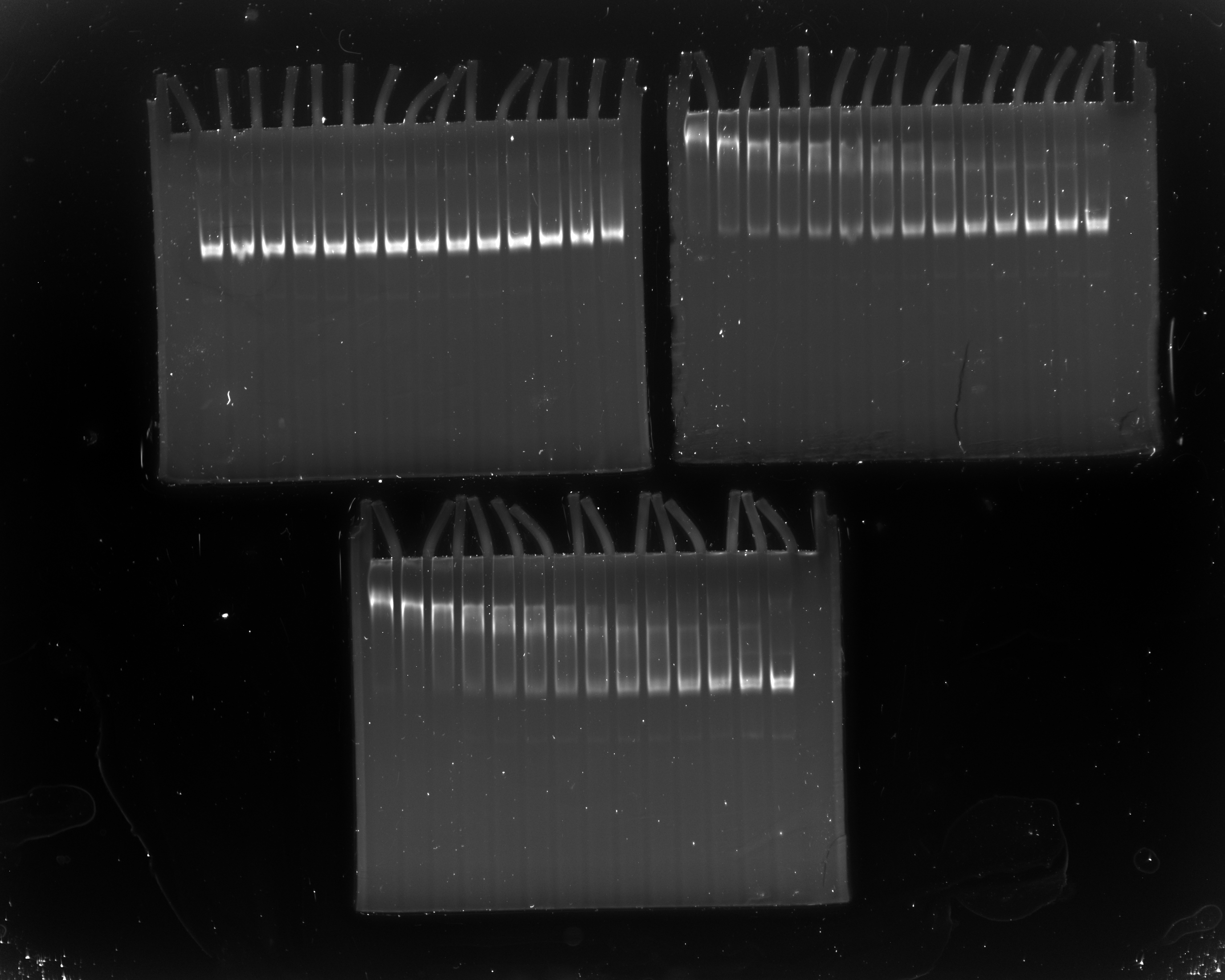

Supplement: Supplementary file 9 — Figure Source Data appendix [file 44319_2024_306_MOESM9_ESM.zip › EMBOR-2024-60481V2_SourceDataFor_appendix/EMBOR-2024-60481V2_SourceDataForAppendix fig S3/S3F/S3F 2.tif]

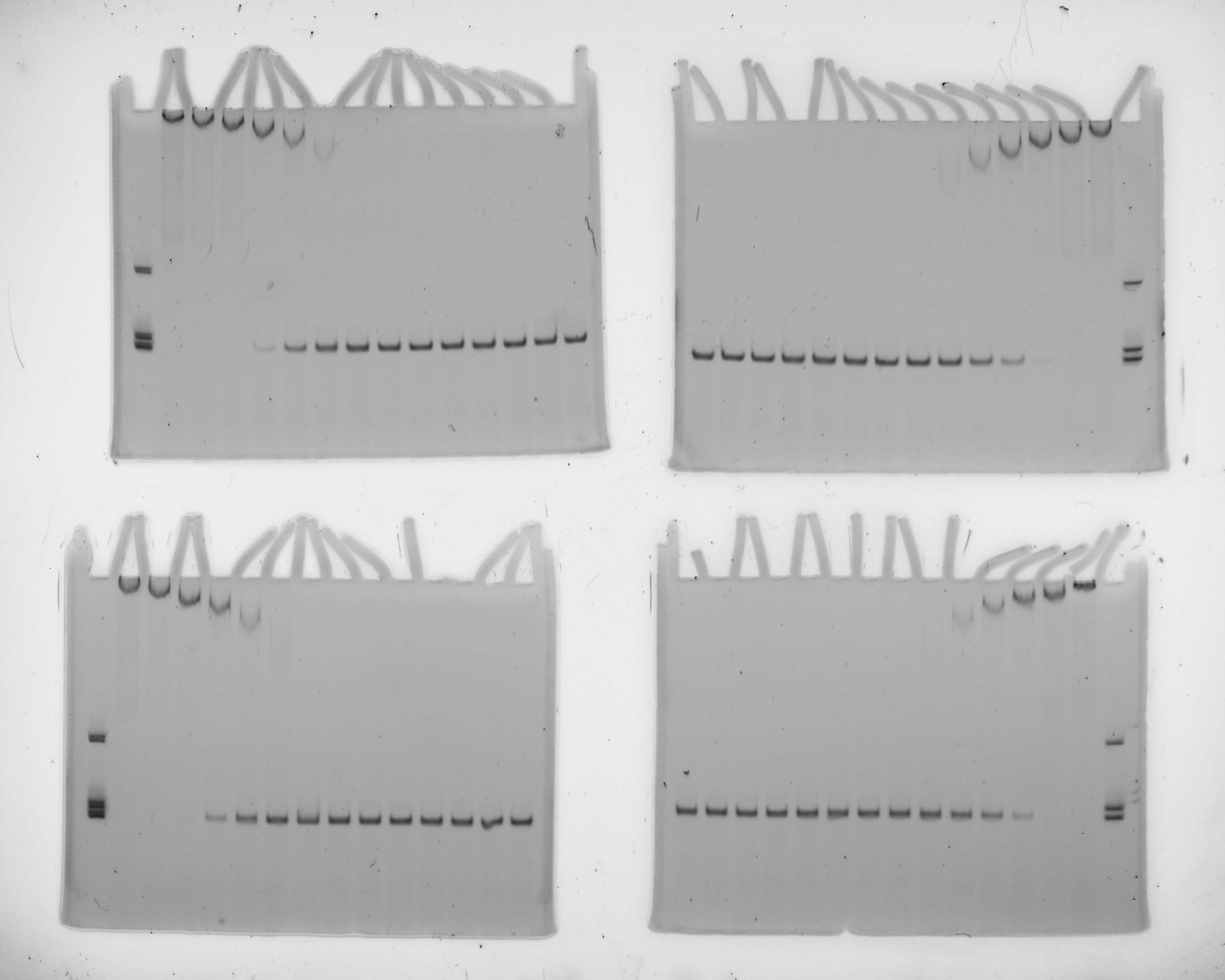

Supplement: Supplementary file 9 — Figure Source Data appendix [file 44319_2024_306_MOESM9_ESM.zip › EMBOR-2024-60481V2_SourceDataFor_appendix/EMBOR-2024-60481V2_SourceDataForAppendix fig S4/S4D/S4D.tif]

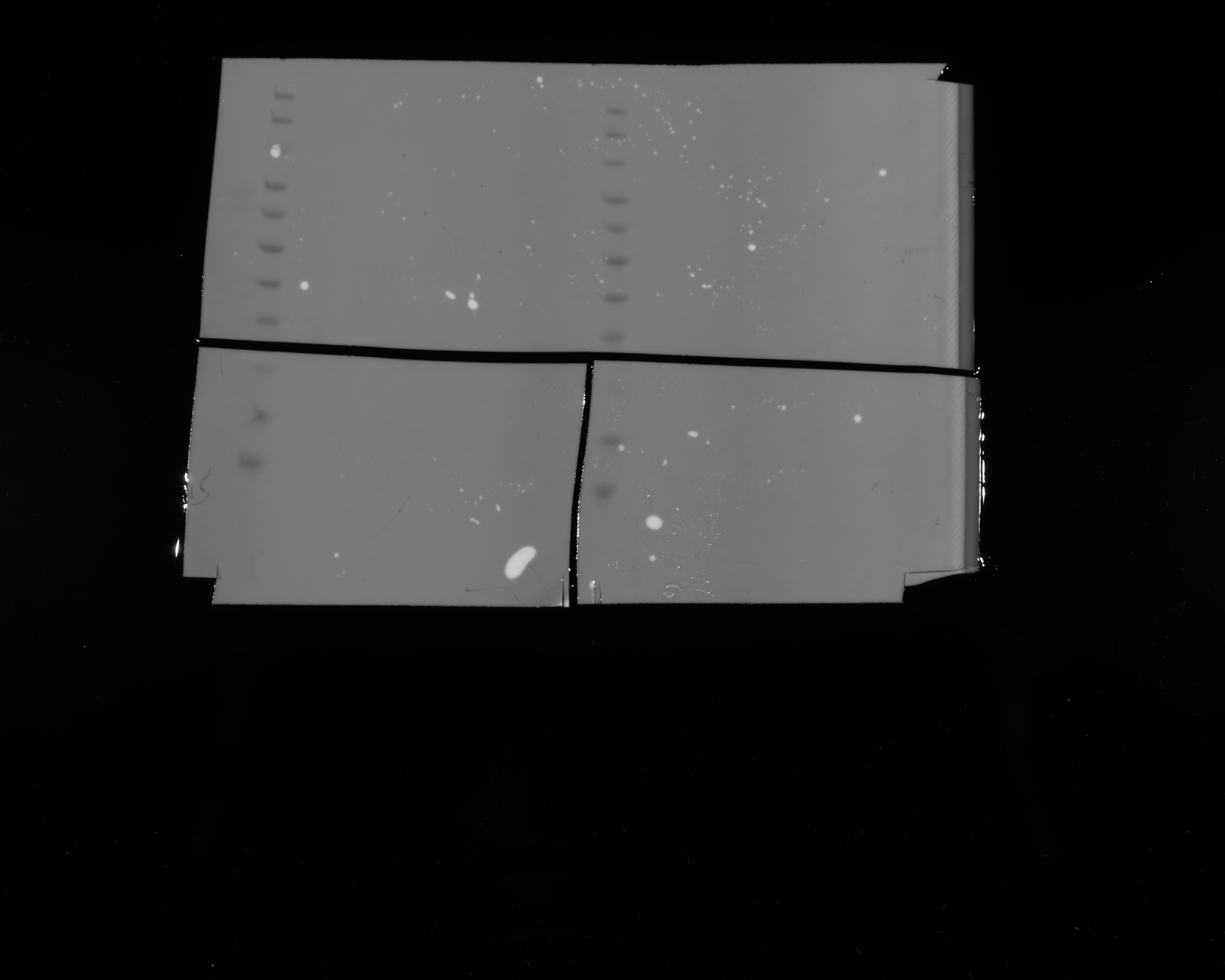

Supplement: Supplementary file 9 — Figure Source Data appendix [file 44319_2024_306_MOESM9_ESM.zip › EMBOR-2024-60481V2_SourceDataFor_appendix/EMBOR-2024-60481V2_SourceDataForAppendix fig S4/S4E/USER2 2023-10-18 16h01m45s colorimetric.tif]

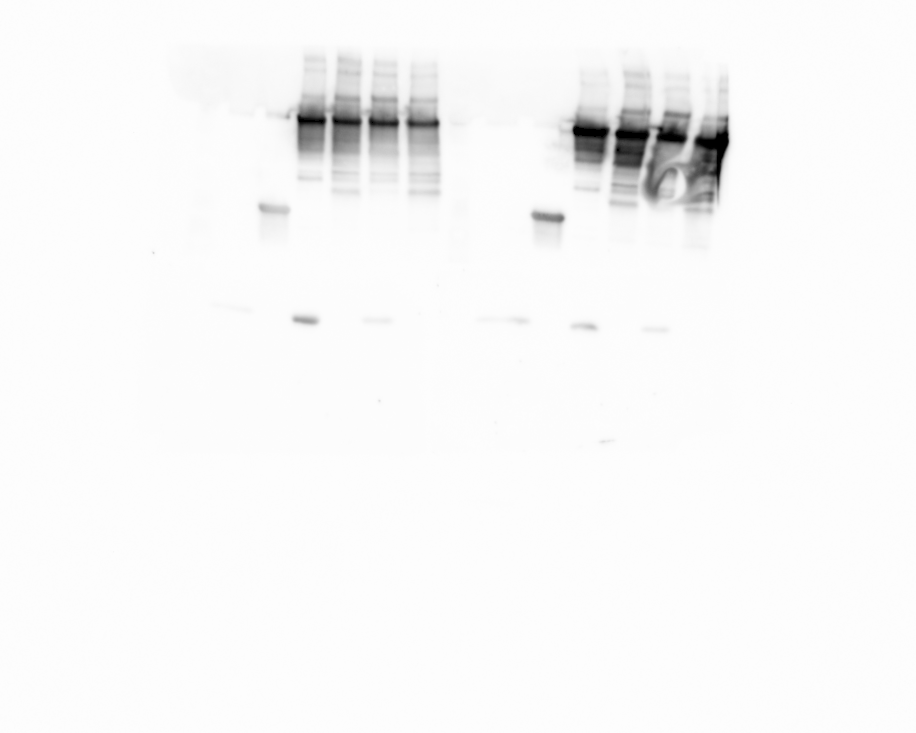

Supplement: Supplementary file 9 — Figure Source Data appendix [file 44319_2024_306_MOESM9_ESM.zip › EMBOR-2024-60481V2_SourceDataFor_appendix/EMBOR-2024-60481V2_SourceDataForAppendix fig S4/S4E/USER2 2023-10-18 16h02m57s 119 Histone.tif]

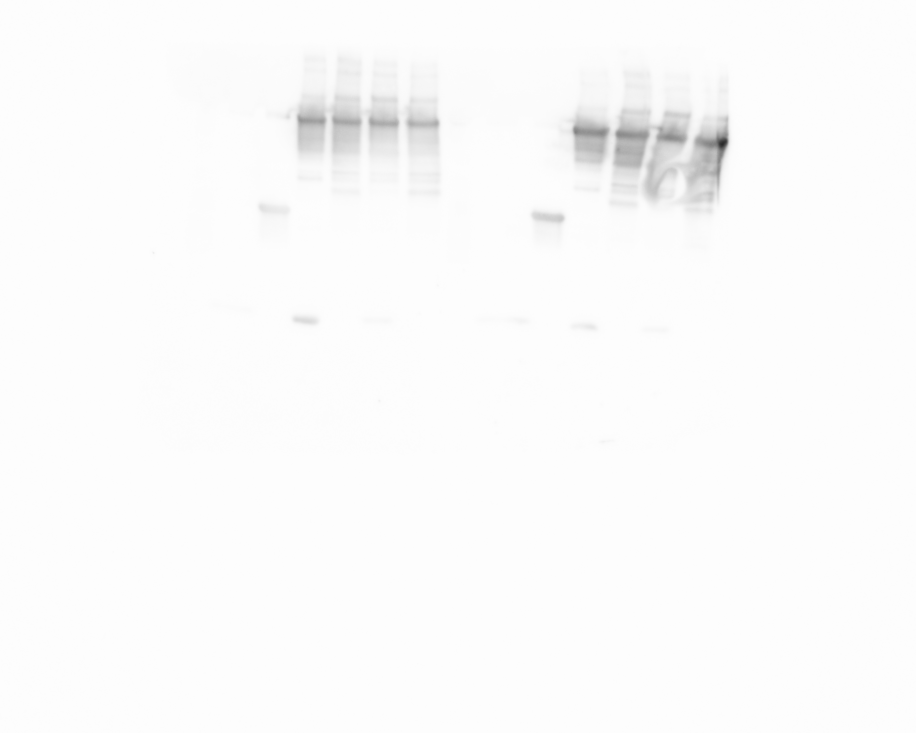

Supplement: Supplementary file 9 — Figure Source Data appendix [file 44319_2024_306_MOESM9_ESM.zip › EMBOR-2024-60481V2_SourceDataFor_appendix/EMBOR-2024-60481V2_SourceDataForAppendix fig S4/S4E/USER2 2023-10-18 16h02m42s 119MBP.tif]

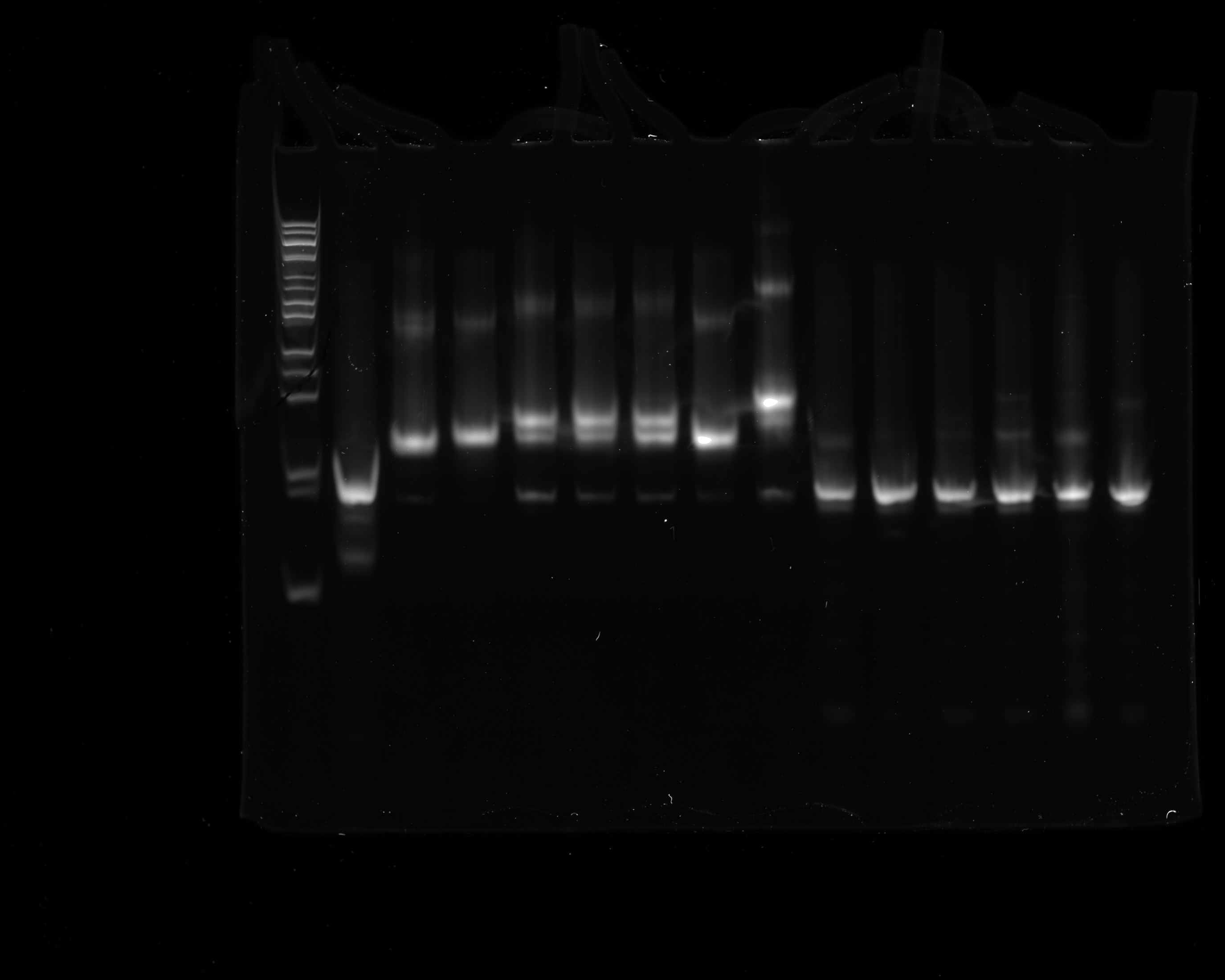

Supplement: Supplementary file 9 — Figure Source Data appendix [file 44319_2024_306_MOESM9_ESM.zip › EMBOR-2024-60481V2_SourceDataFor_appendix/EMBOR-2024-60481V2_SourceDataForAppendix fig S4/S4B/S4B native 2.tif]

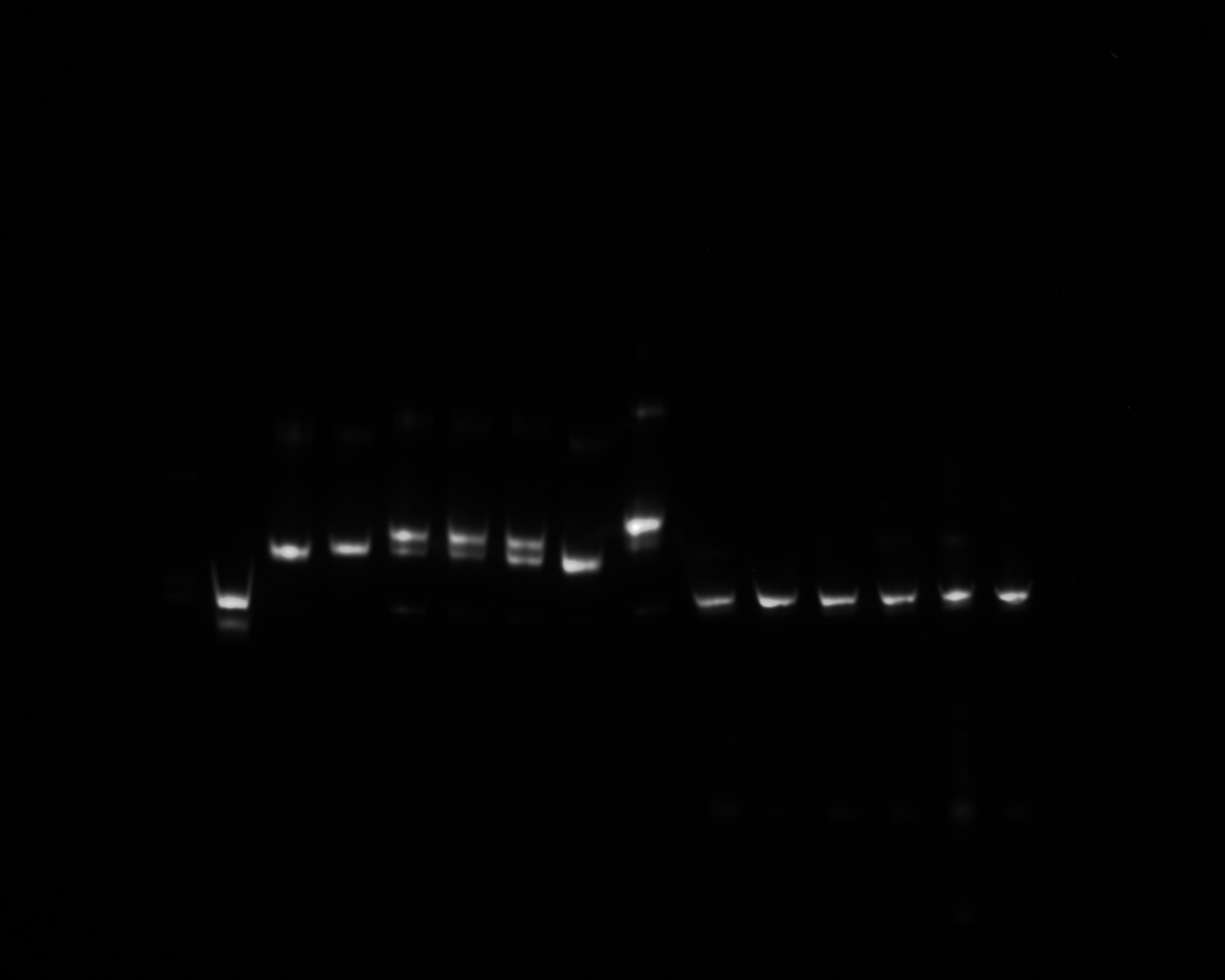

Supplement: Supplementary file 9 — Figure Source Data appendix [file 44319_2024_306_MOESM9_ESM.zip › EMBOR-2024-60481V2_SourceDataFor_appendix/EMBOR-2024-60481V2_SourceDataForAppendix fig S4/S4B/S4B native 1.tif]

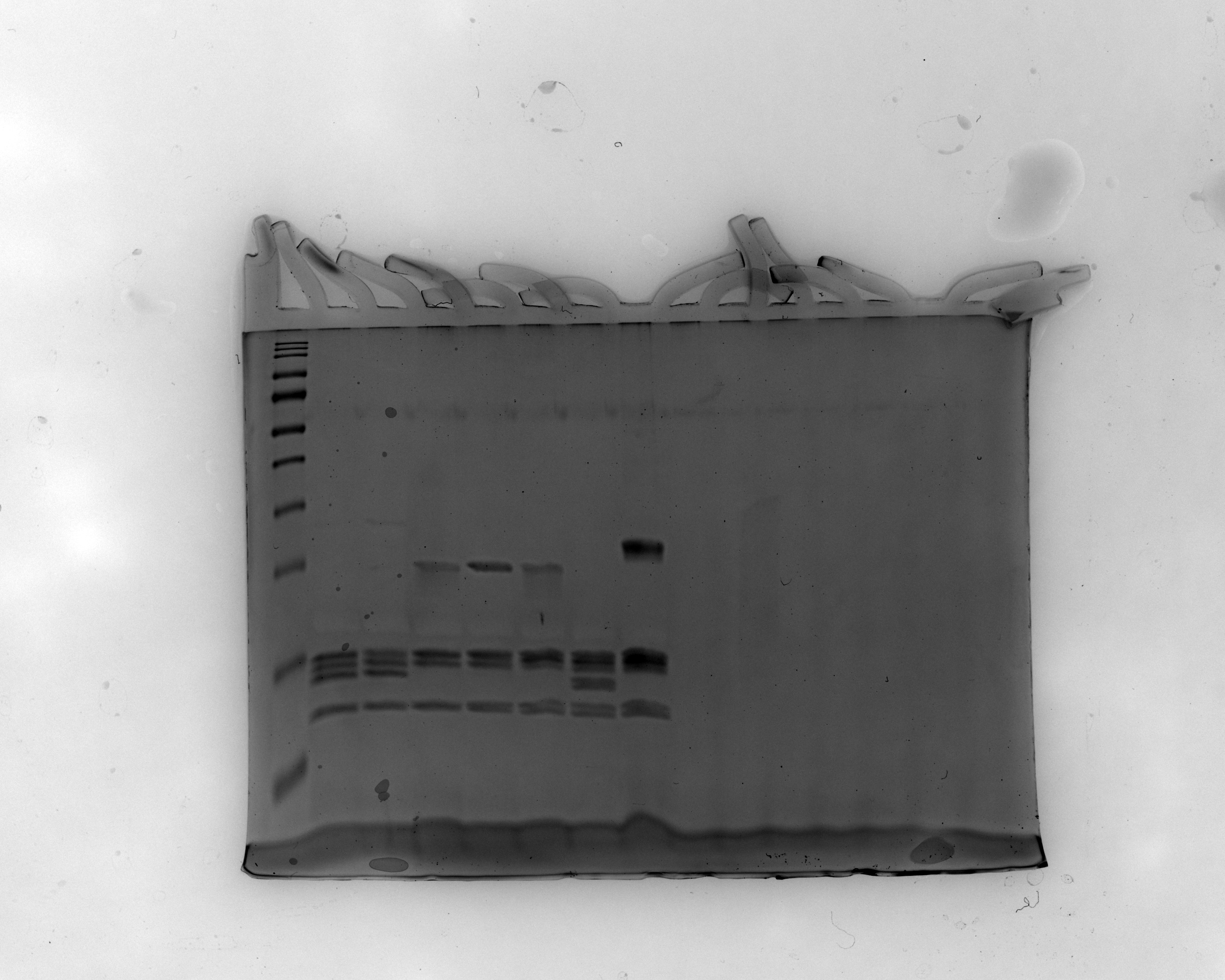

Supplement: Supplementary file 9 — Figure Source Data appendix [file 44319_2024_306_MOESM9_ESM.zip › EMBOR-2024-60481V2_SourceDataFor_appendix/EMBOR-2024-60481V2_SourceDataForAppendix fig S4/S4B/S4B SDSpage.tif]

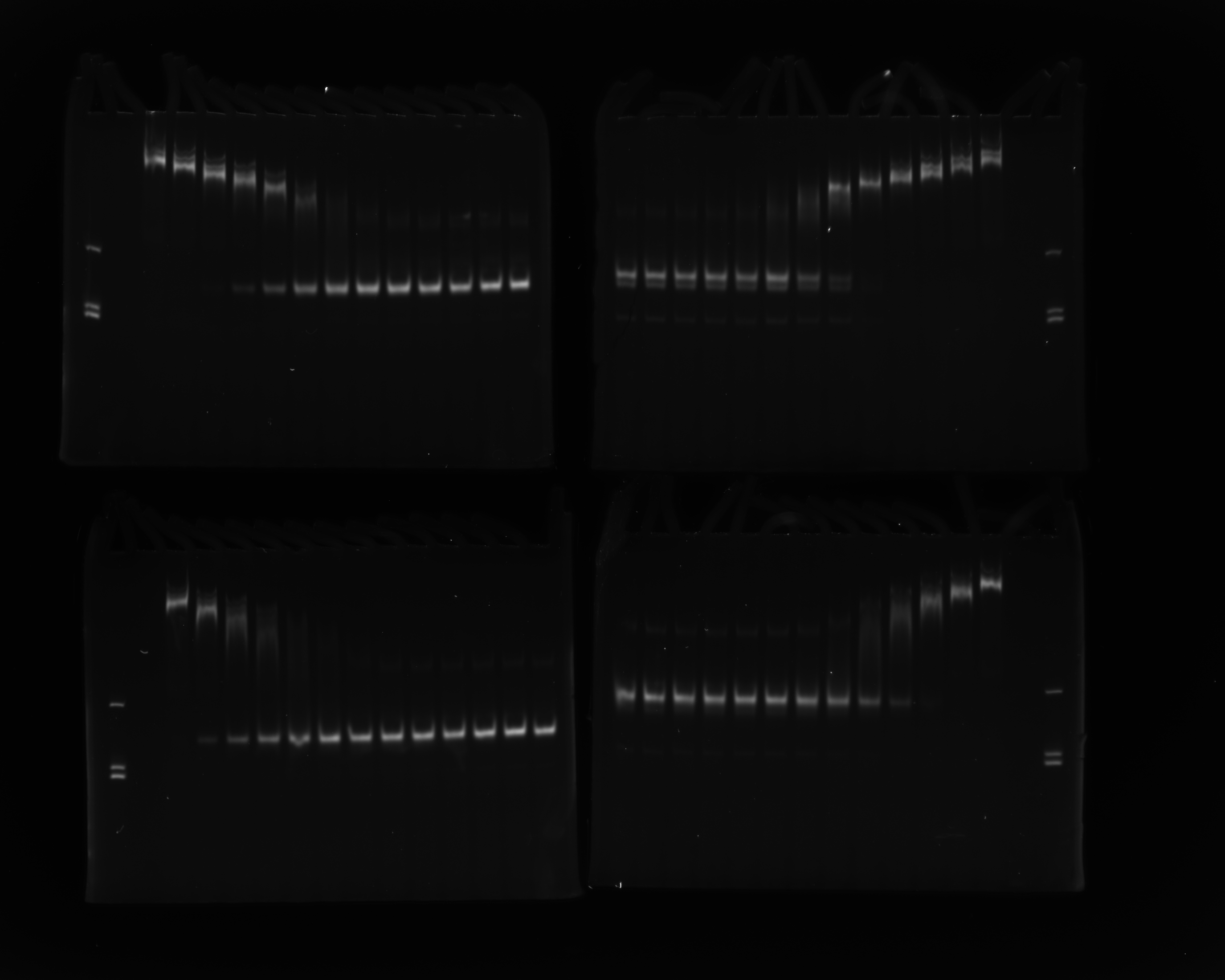

Supplement: Supplementary file 9 — Figure Source Data appendix [file 44319_2024_306_MOESM9_ESM.zip › EMBOR-2024-60481V2_SourceDataFor_appendix/EMBOR-2024-60481V2_SourceDataForAppendix fig S4/S4G/S4G.tif]

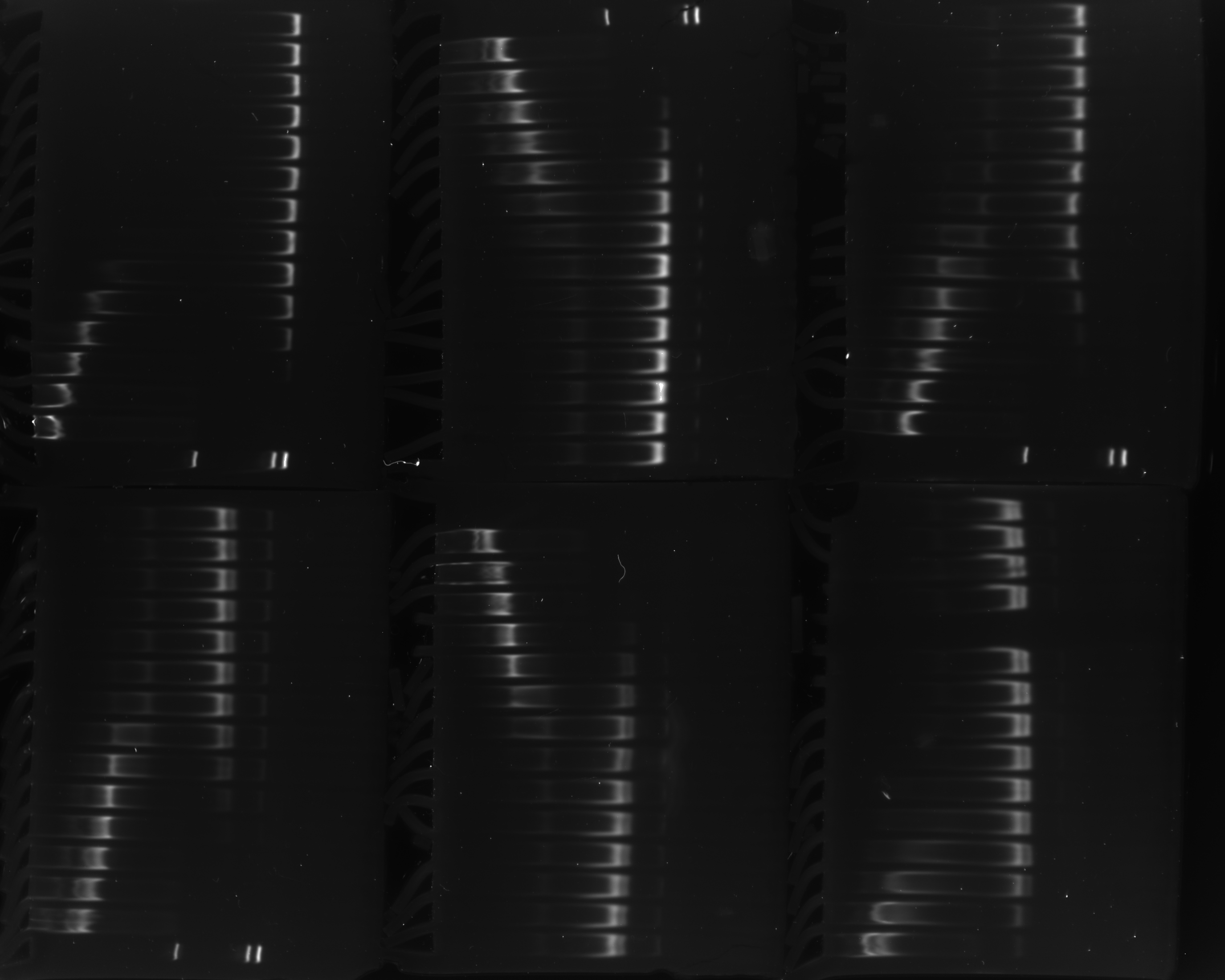

Supplement: Supplementary file 9 — Figure Source Data appendix [file 44319_2024_306_MOESM9_ESM.zip › EMBOR-2024-60481V2_SourceDataFor_appendix/EMBOR-2024-60481V2_SourceDataForAppendix fig S4/S4G/S4G repeat.tif]

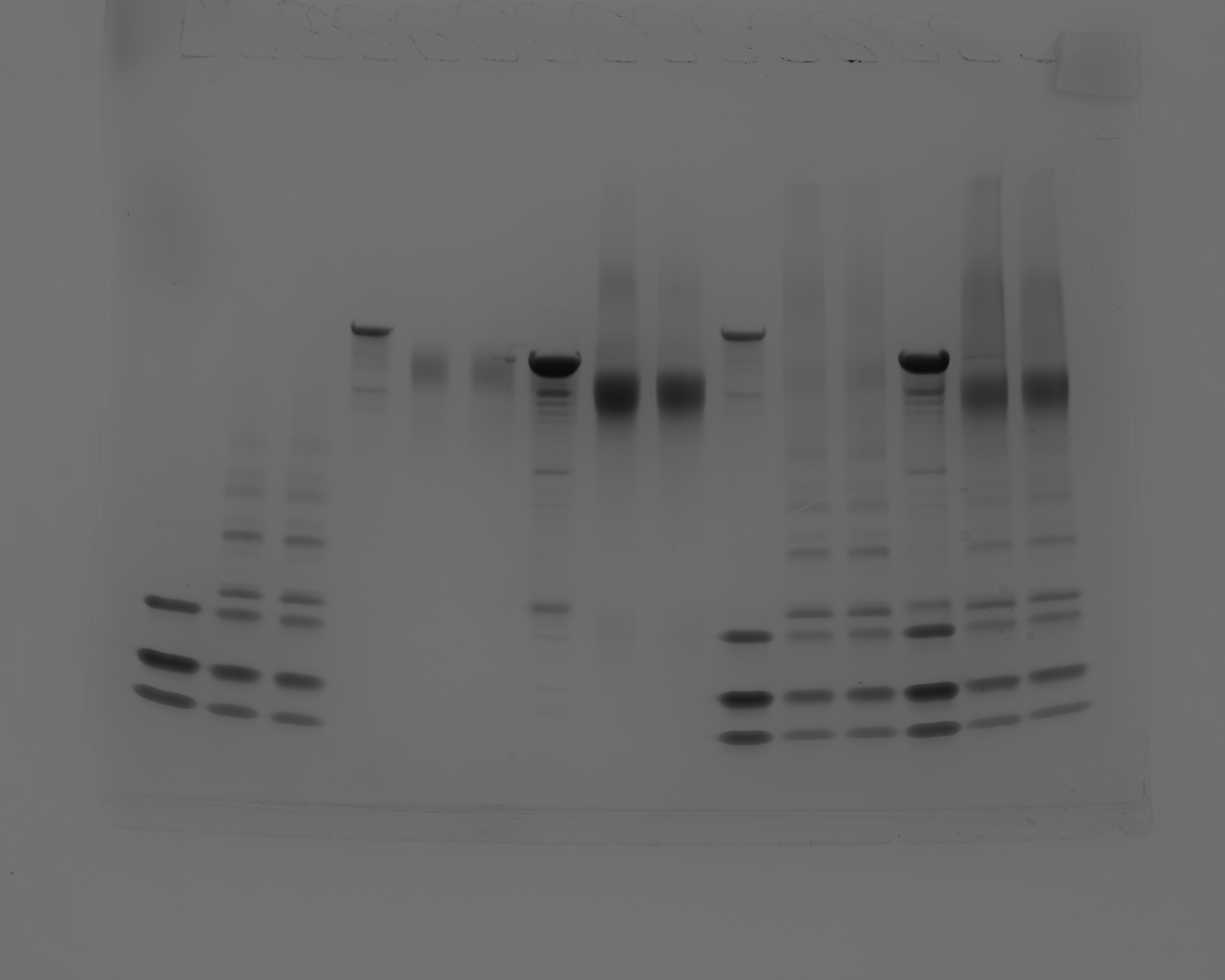

Supplement: Supplementary file 9 — Figure Source Data appendix [file 44319_2024_306_MOESM9_ESM.zip › EMBOR-2024-60481V2_SourceDataFor_appendix/EMBOR-2024-60481V2_SourceDataForAppendix fig S5/S5A/S6A.tif]

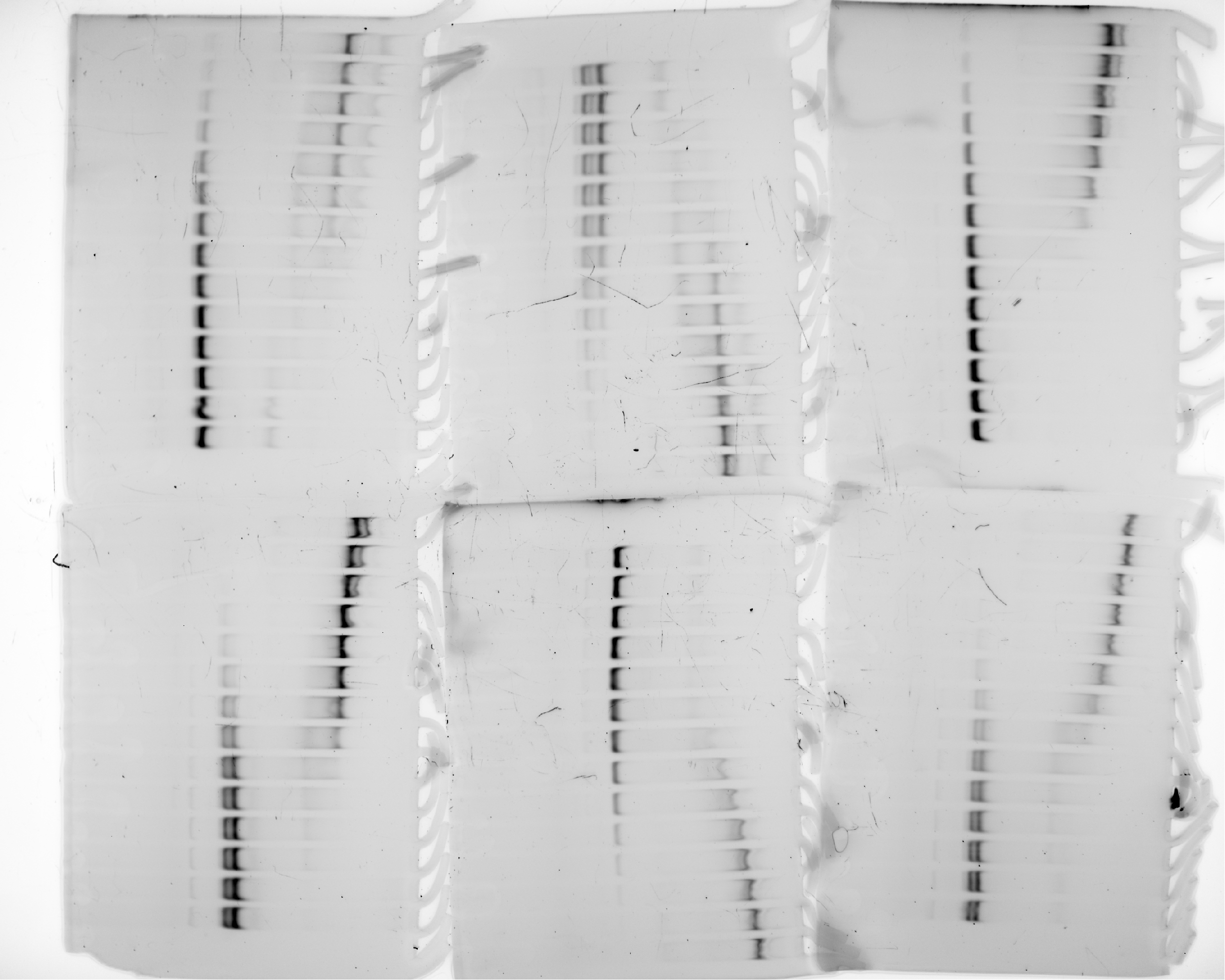

Supplement: Supplementary file 9 — Figure Source Data appendix [file 44319_2024_306_MOESM9_ESM.zip › EMBOR-2024-60481V2_SourceDataFor_appendix/EMBOR-2024-60481V2_SourceDataForAppendix fig S5/S5D/S5D repeat.tif]

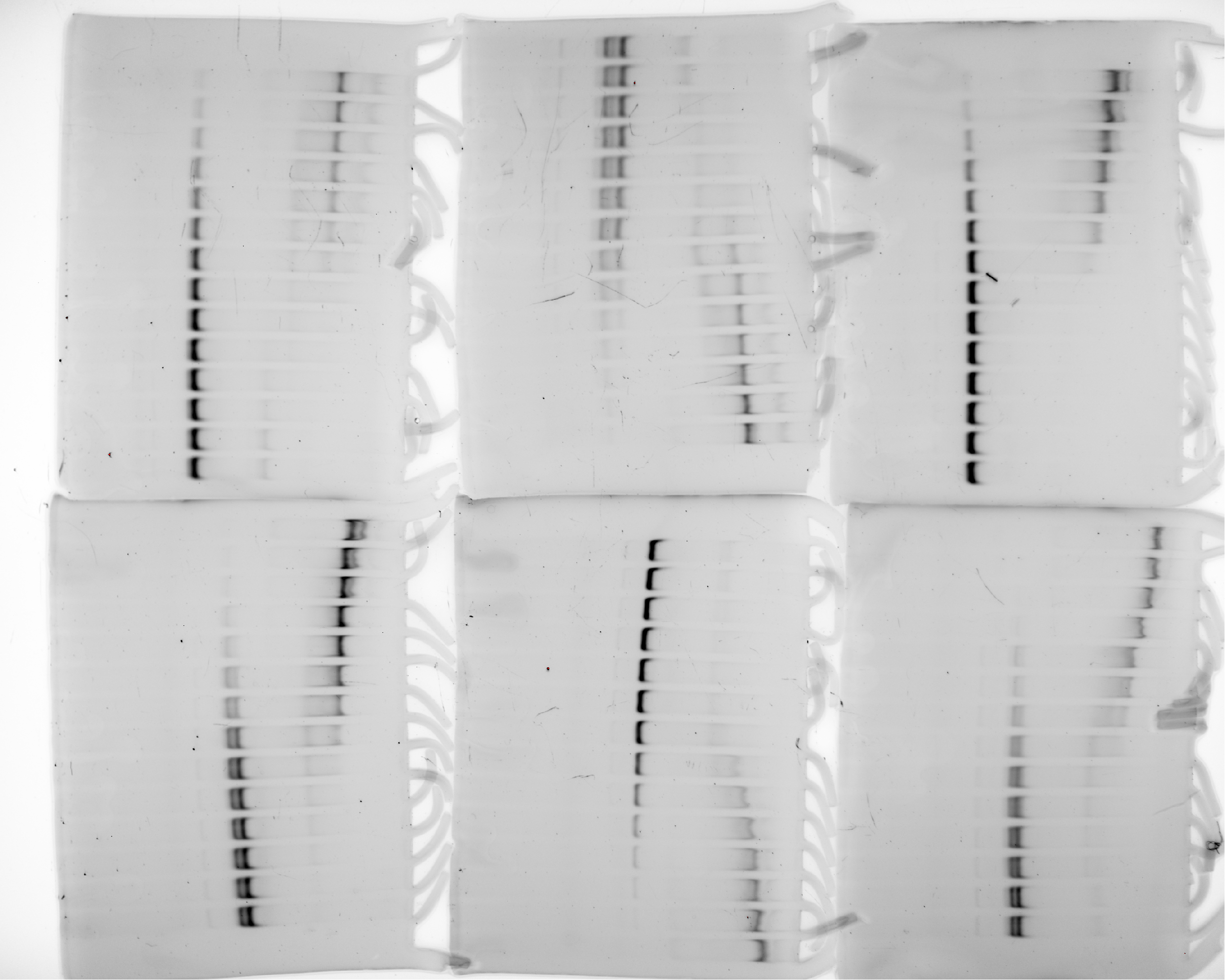

Supplement: Supplementary file 9 — Figure Source Data appendix [file 44319_2024_306_MOESM9_ESM.zip › EMBOR-2024-60481V2_SourceDataFor_appendix/EMBOR-2024-60481V2_SourceDataForAppendix fig S5/S5D/S5D.tif]

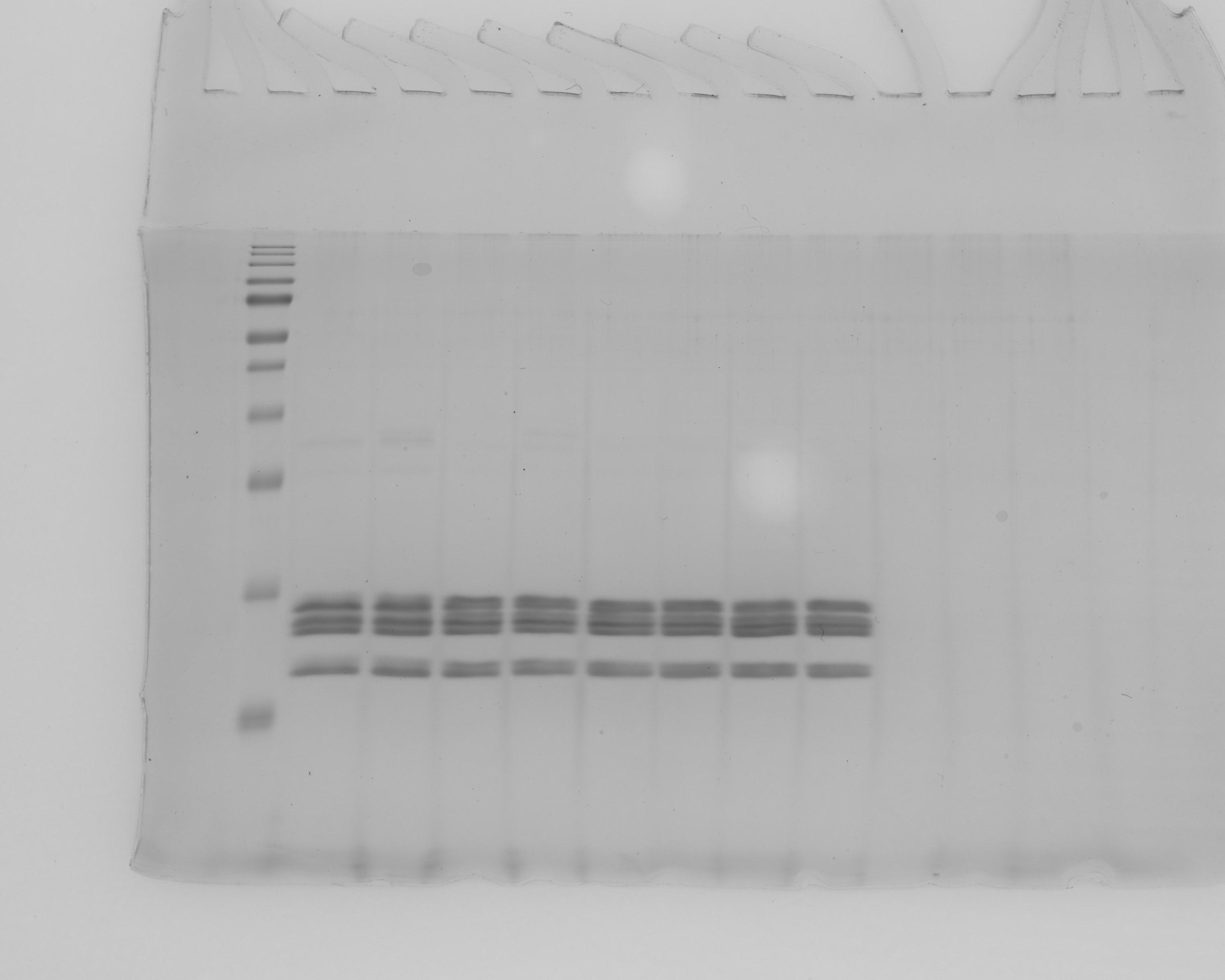

Supplement: Supplementary file 10 — Expanded View Figure Source Data [file 44319_2024_306_MOESM10_ESM.zip › EMBOR-2024-60481V2_SourceDataFor_Expandedview/EMBOR-2024-60481V2_SourceDataForFigEV5/EV5B/321018 nucs hw_3.tif]

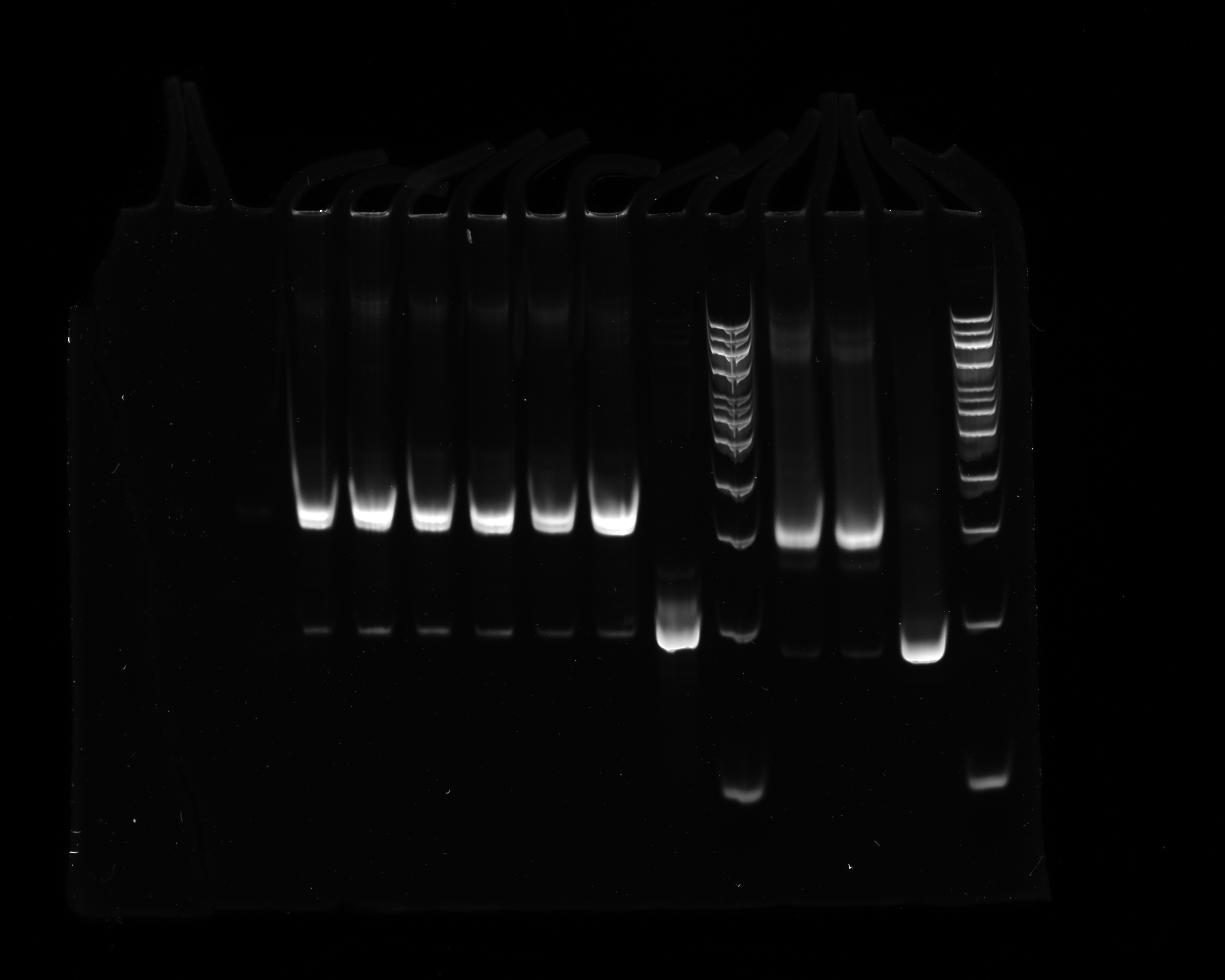

Supplement: Supplementary file 10 — Expanded View Figure Source Data [file 44319_2024_306_MOESM10_ESM.zip › EMBOR-2024-60481V2_SourceDataFor_Expandedview/EMBOR-2024-60481V2_SourceDataForFigEV5/EV5B/321018 nucs hw_1.tif]

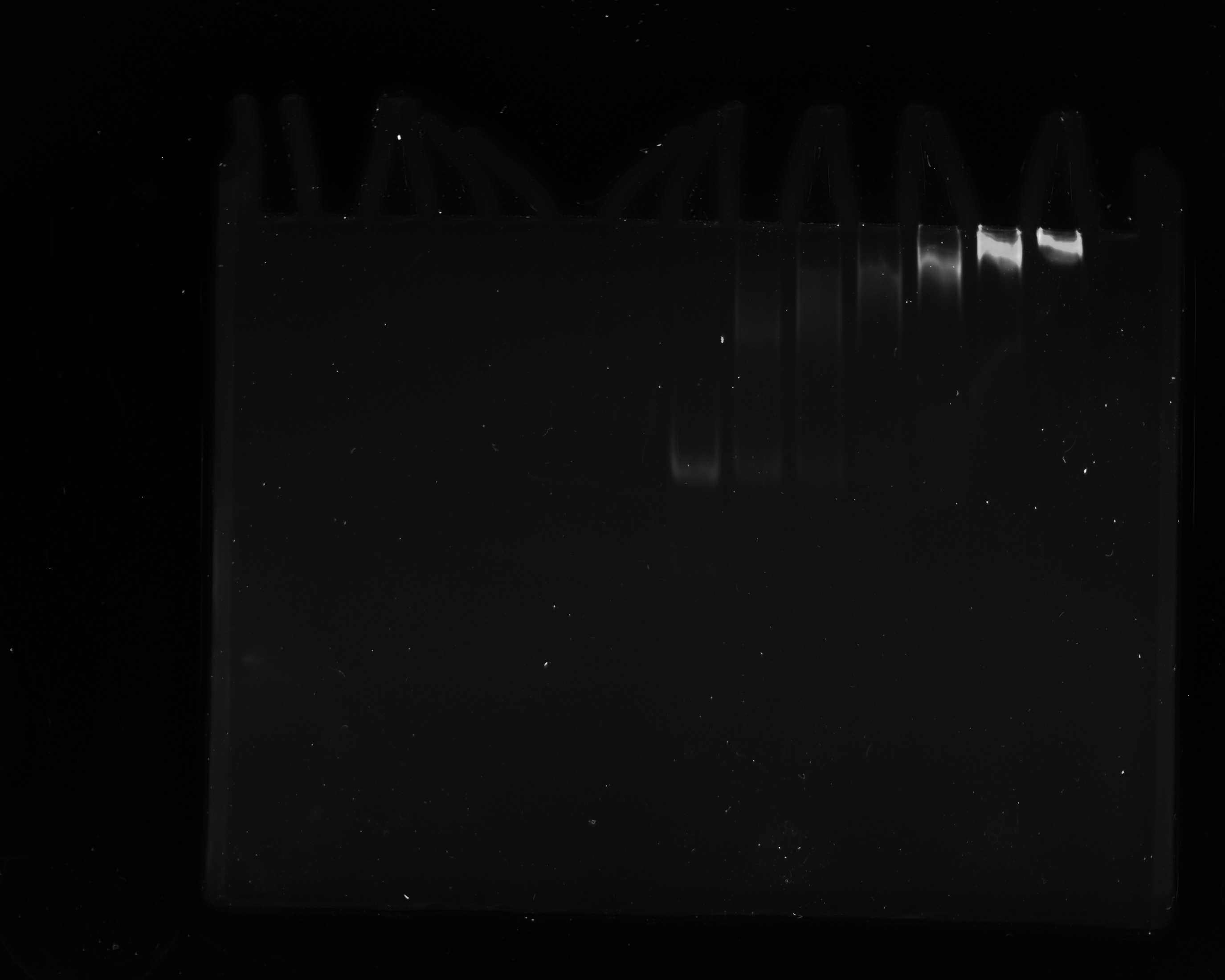

Supplement: Supplementary file 10 — Expanded View Figure Source Data [file 44319_2024_306_MOESM10_ESM.zip › EMBOR-2024-60481V2_SourceDataFor_Expandedview/EMBOR-2024-60481V2_SourceDataForFigEV5/EV5D/EV5D 1.2.tif]

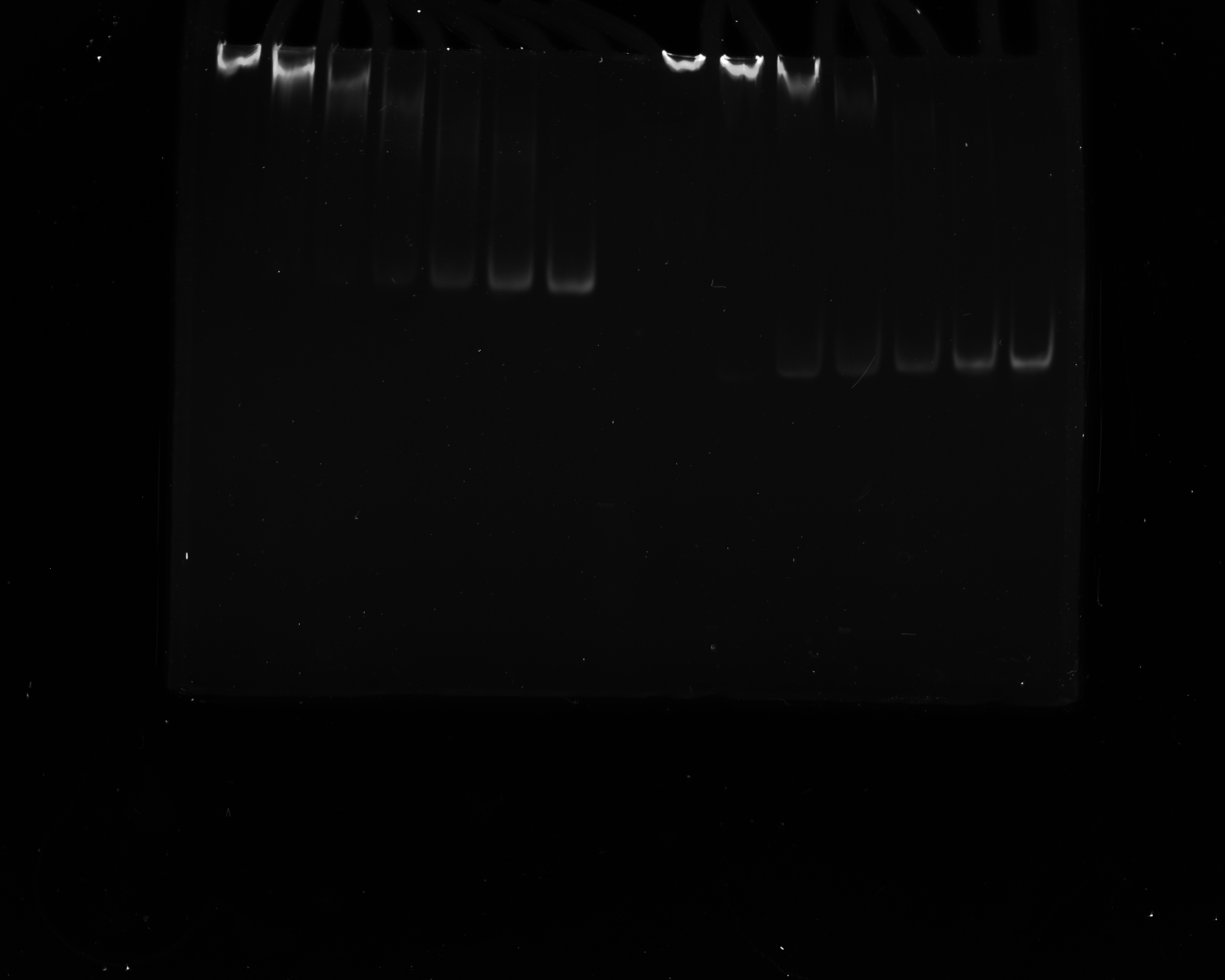

Supplement: Supplementary file 10 — Expanded View Figure Source Data [file 44319_2024_306_MOESM10_ESM.zip › EMBOR-2024-60481V2_SourceDataFor_Expandedview/EMBOR-2024-60481V2_SourceDataForFigEV5/EV5D/EV5D 1.1.tif]

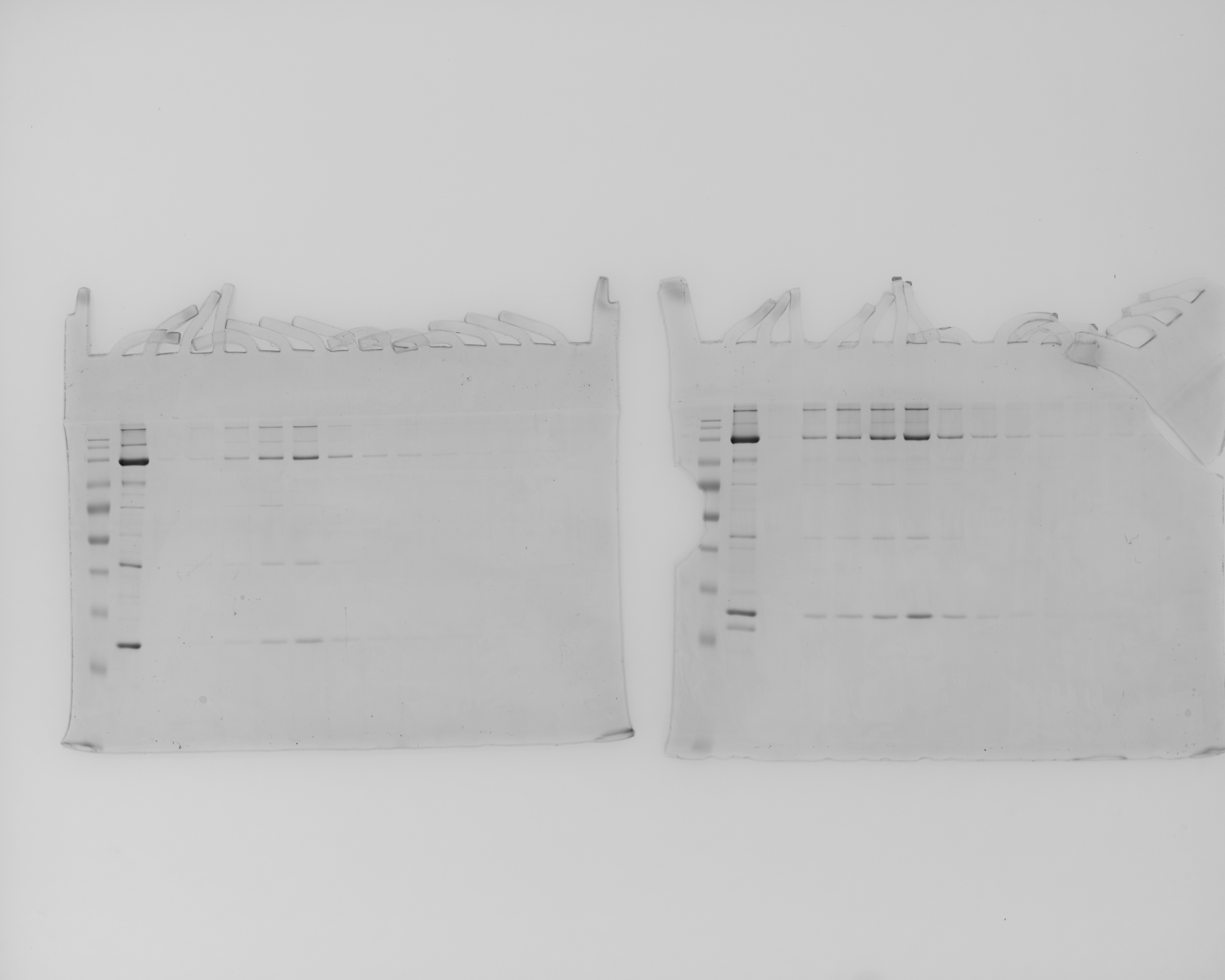

Supplement: Supplementary file 10 — Expanded View Figure Source Data [file 44319_2024_306_MOESM10_ESM.zip › EMBOR-2024-60481V2_SourceDataFor_Expandedview/EMBOR-2024-60481V2_SourceDataForFigEV5/EV5A/EV5 SDS page.tif]

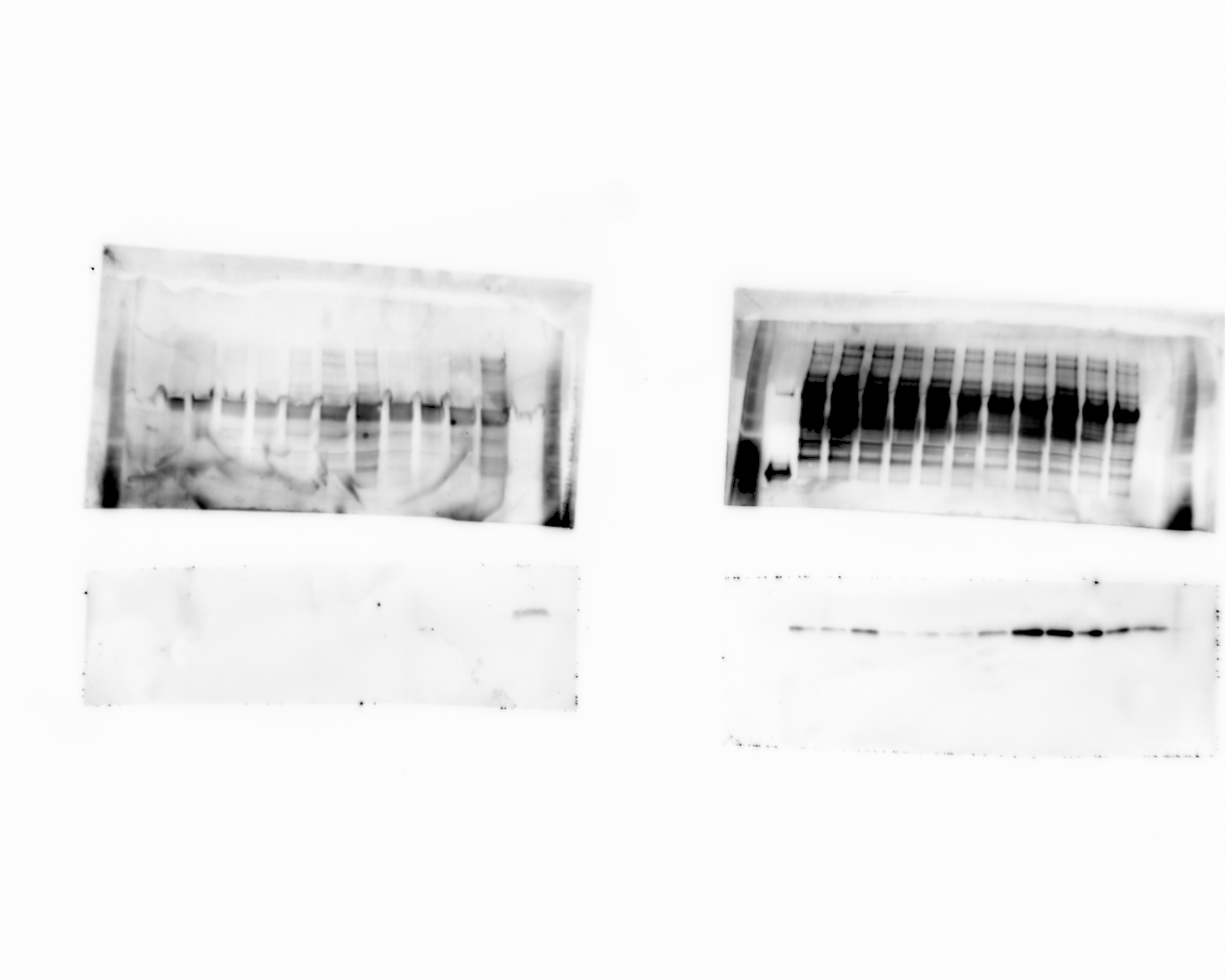

Supplement: Supplementary file 10 — Expanded View Figure Source Data [file 44319_2024_306_MOESM10_ESM.zip › EMBOR-2024-60481V2_SourceDataFor_Expandedview/EMBOR-2024-60481V2_SourceDataForFigEV2/EV2D/EV2D H2B.tif]

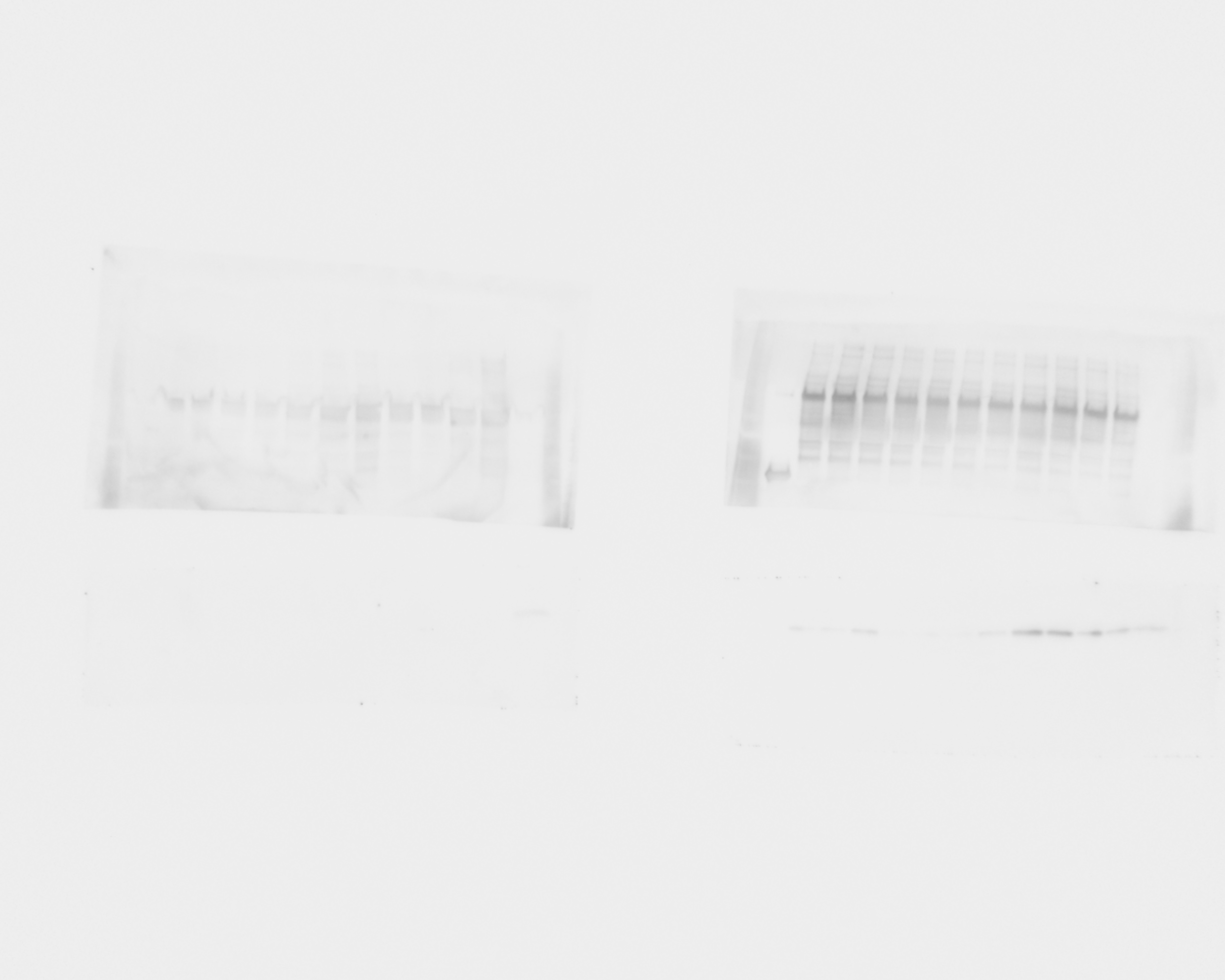

Supplement: Supplementary file 10 — Expanded View Figure Source Data [file 44319_2024_306_MOESM10_ESM.zip › EMBOR-2024-60481V2_SourceDataFor_Expandedview/EMBOR-2024-60481V2_SourceDataForFigEV2/EV2D/EV2D MBP.tif]

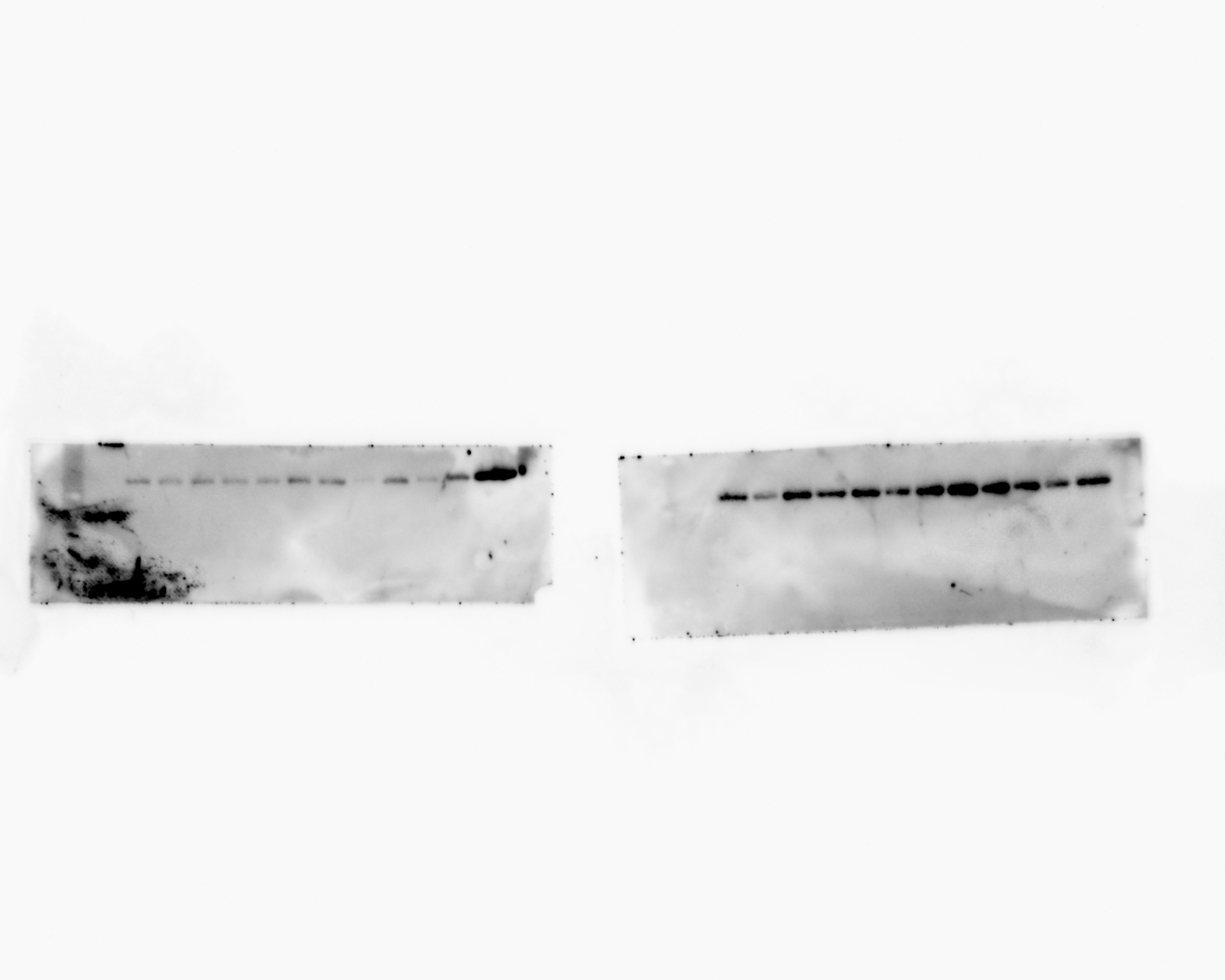

Supplement: Supplementary file 10 — Expanded View Figure Source Data [file 44319_2024_306_MOESM10_ESM.zip › EMBOR-2024-60481V2_SourceDataFor_Expandedview/EMBOR-2024-60481V2_SourceDataForFigEV2/EV2D/EV2D H3.tif]

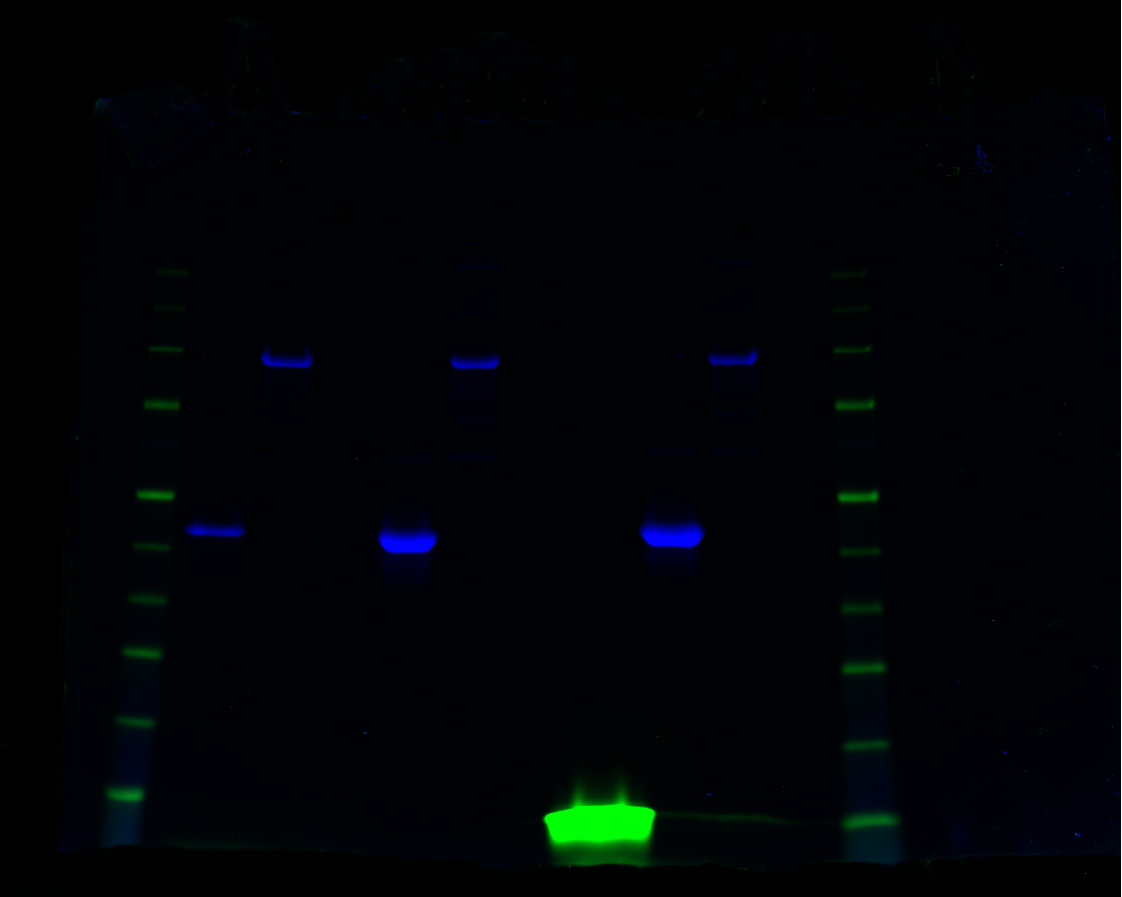

Supplement: Supplementary file 10 — Expanded View Figure Source Data [file 44319_2024_306_MOESM10_ESM.zip › EMBOR-2024-60481V2_SourceDataFor_Expandedview/EMBOR-2024-60481V2_SourceDataForFigEV2/EV2C/220901 stainfree with fluorescence.tif]

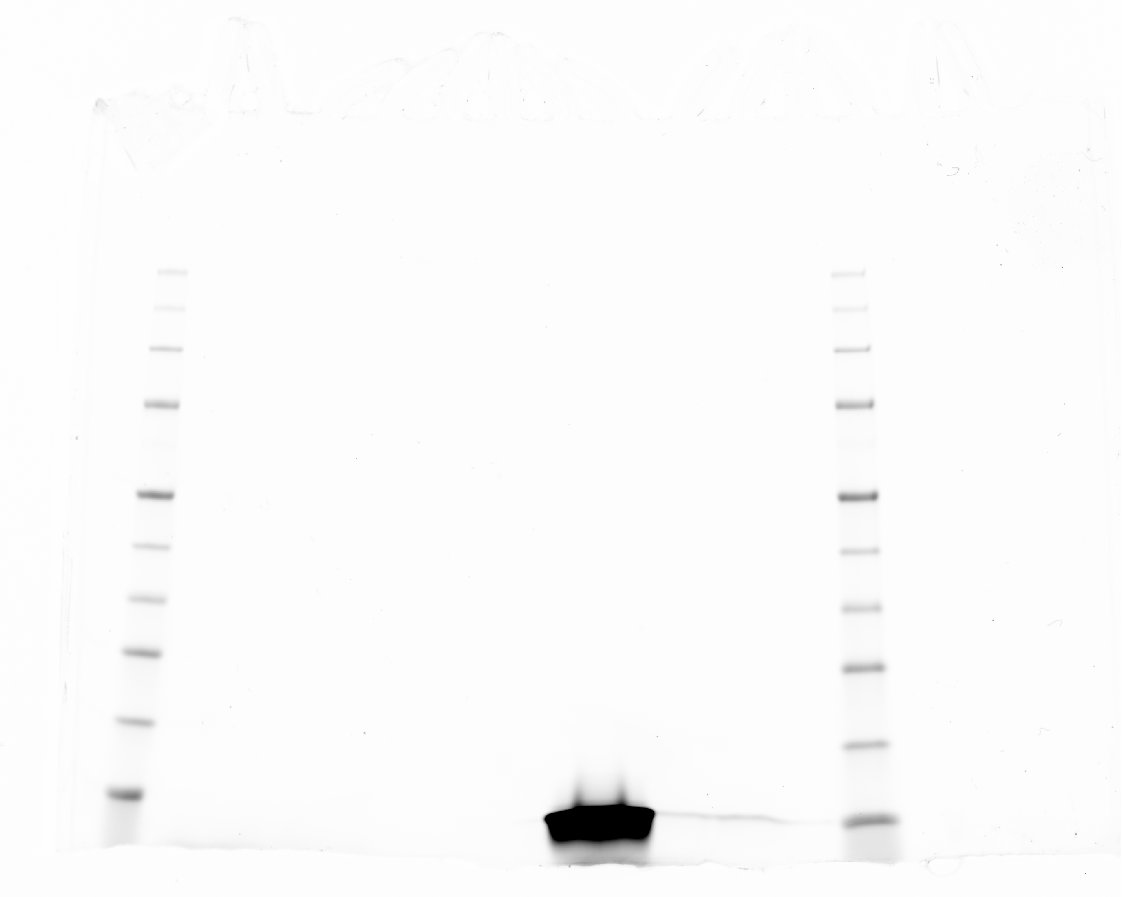

Supplement: Supplementary file 10 — Expanded View Figure Source Data [file 44319_2024_306_MOESM10_ESM.zip › EMBOR-2024-60481V2_SourceDataFor_Expandedview/EMBOR-2024-60481V2_SourceDataForFigEV2/EV2C/2022-09-02 16h22m27s Alexa 647 99.993s.tif]

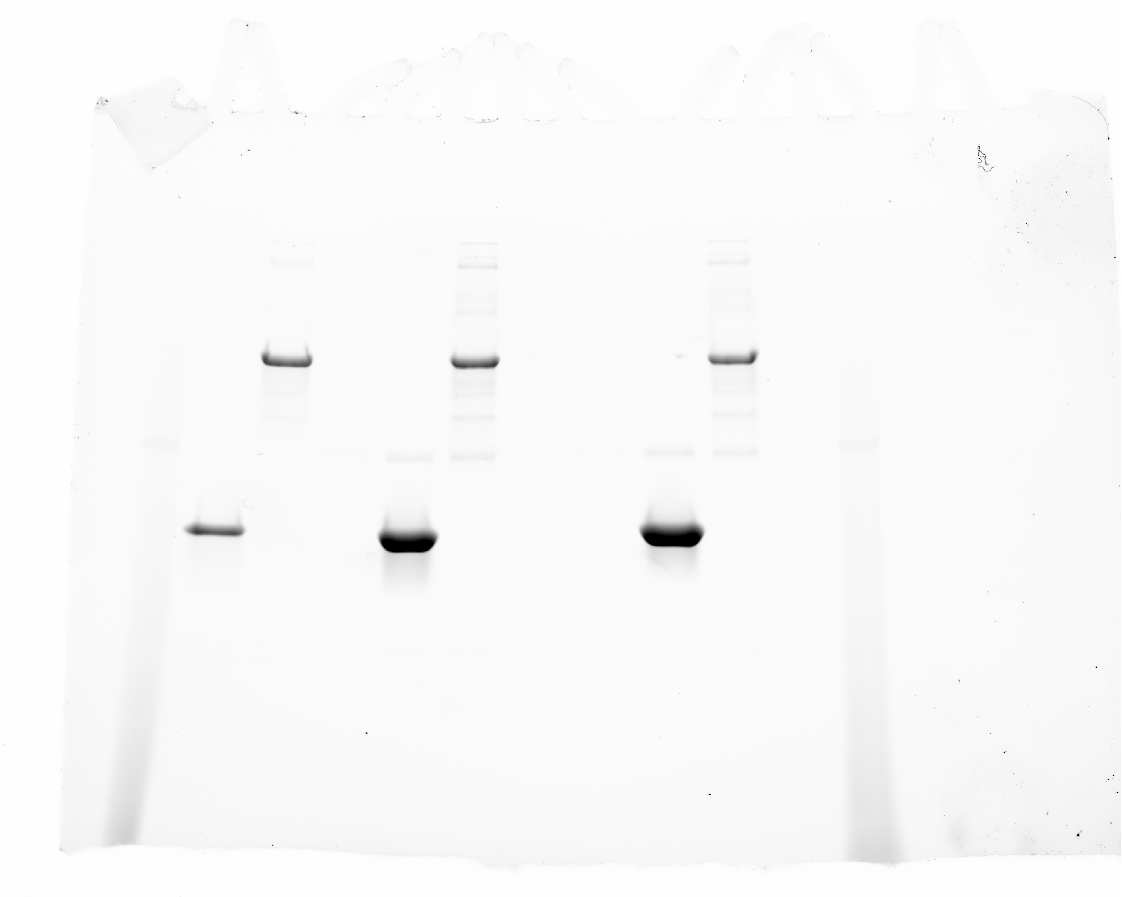

Supplement: Supplementary file 10 — Expanded View Figure Source Data [file 44319_2024_306_MOESM10_ESM.zip › EMBOR-2024-60481V2_SourceDataFor_Expandedview/EMBOR-2024-60481V2_SourceDataForFigEV2/EV2C/220901 stainfree.tif]

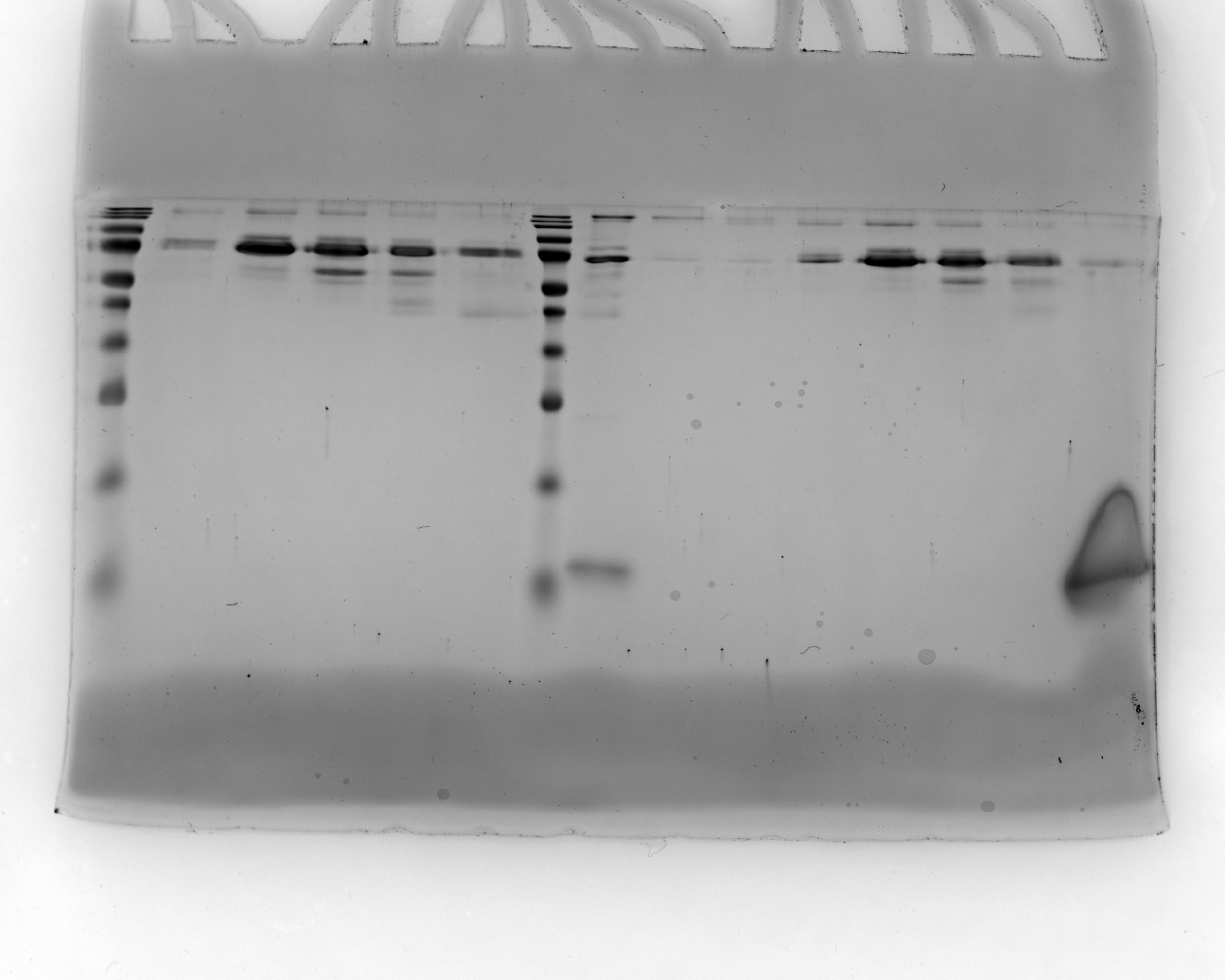

Supplement: Supplementary file 10 — Expanded View Figure Source Data [file 44319_2024_306_MOESM10_ESM.zip › EMBOR-2024-60481V2_SourceDataFor_Expandedview/EMBOR-2024-60481V2_SourceDataForFigEV2/EV2A/230329 sec ub 3a nt hw.tif]

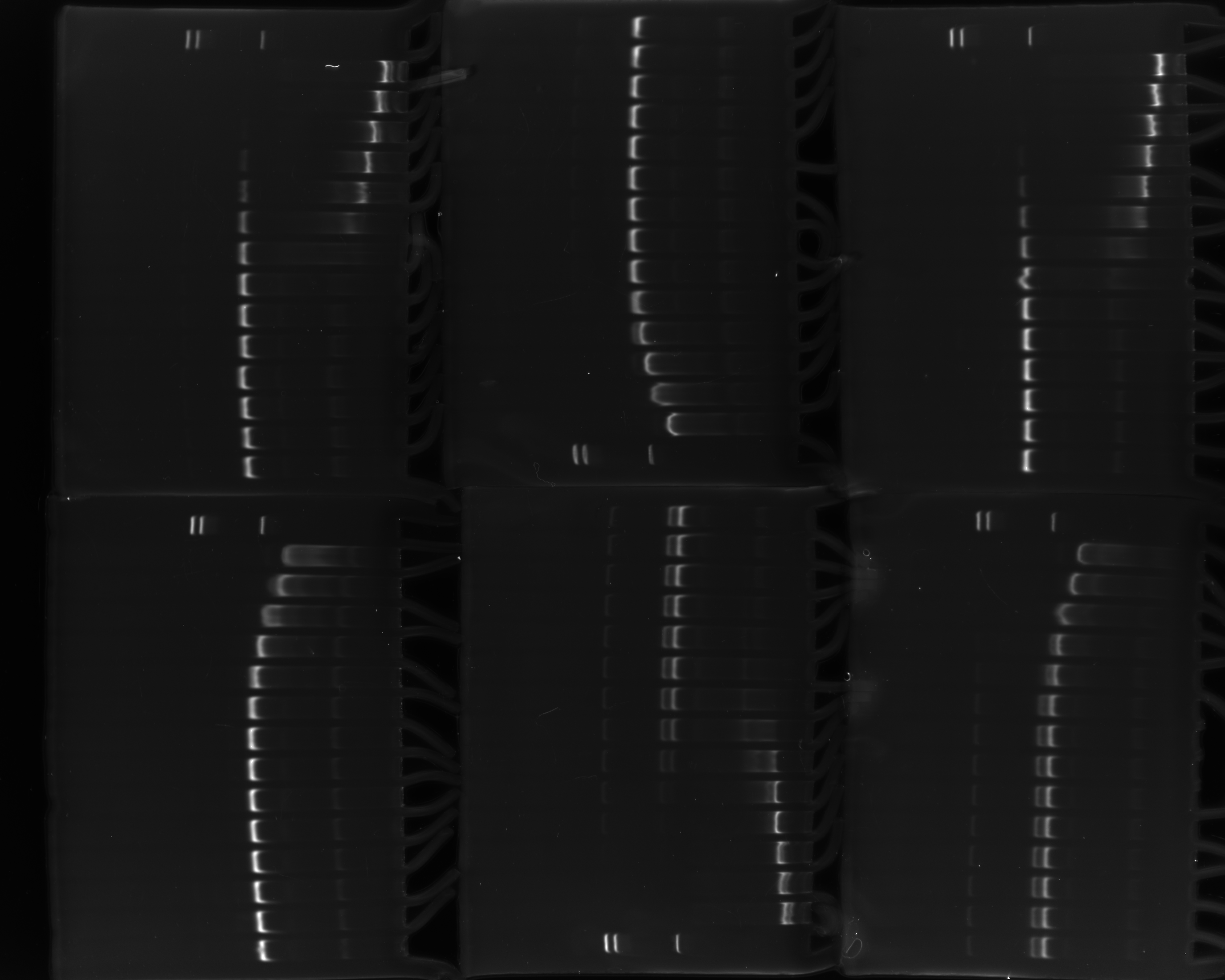

Supplement: Supplementary file 10 — Expanded View Figure Source Data [file 44319_2024_306_MOESM10_ESM.zip › EMBOR-2024-60481V2_SourceDataFor_Expandedview/EMBOR-2024-60481V2_SourceDataForFigEV3/EV3F/EV3F.tif]

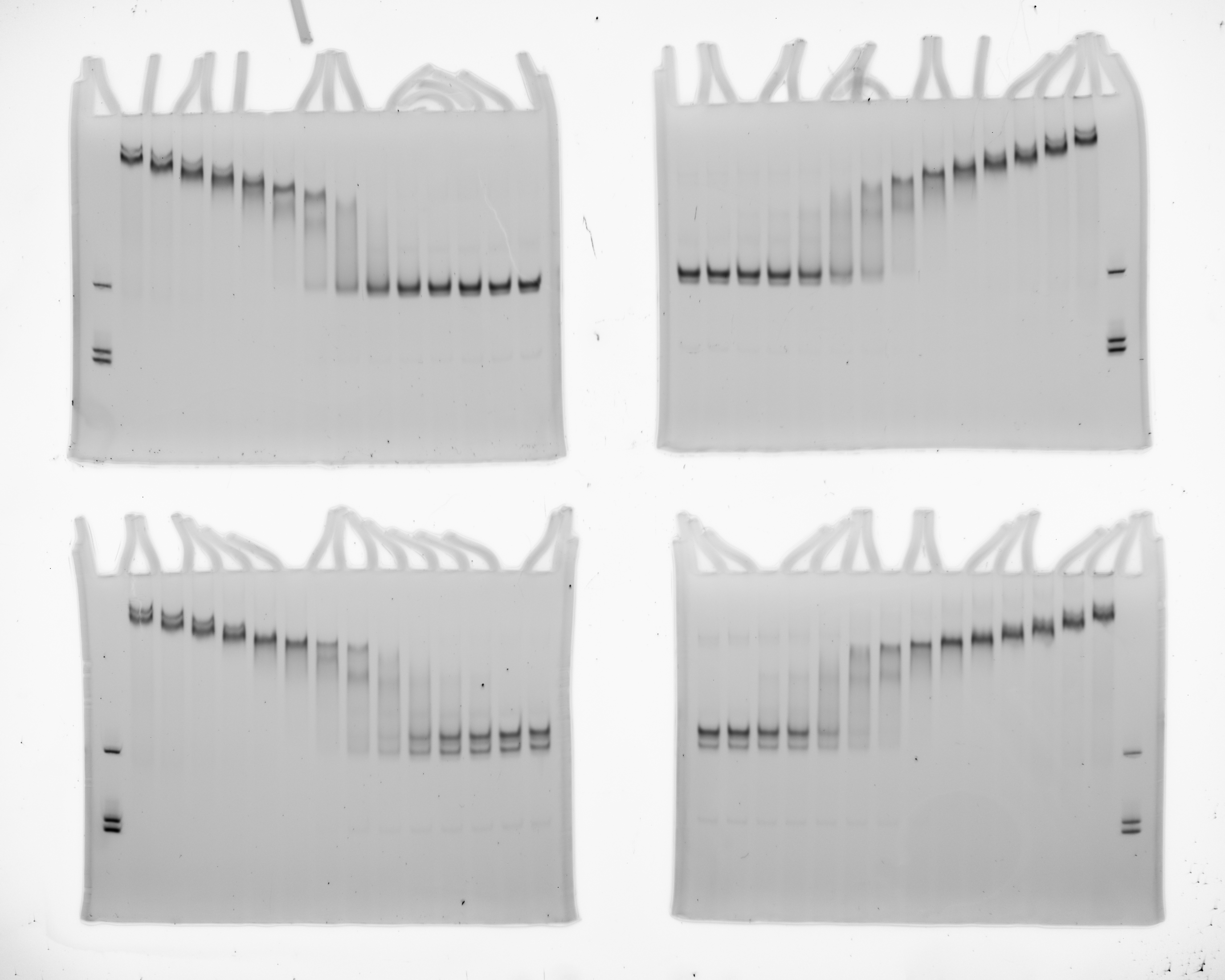

Supplement: Supplementary file 10 — Expanded View Figure Source Data [file 44319_2024_306_MOESM10_ESM.zip › EMBOR-2024-60481V2_SourceDataFor_Expandedview/EMBOR-2024-60481V2_SourceDataForFigEV3/EV3B/EV3B.tif]

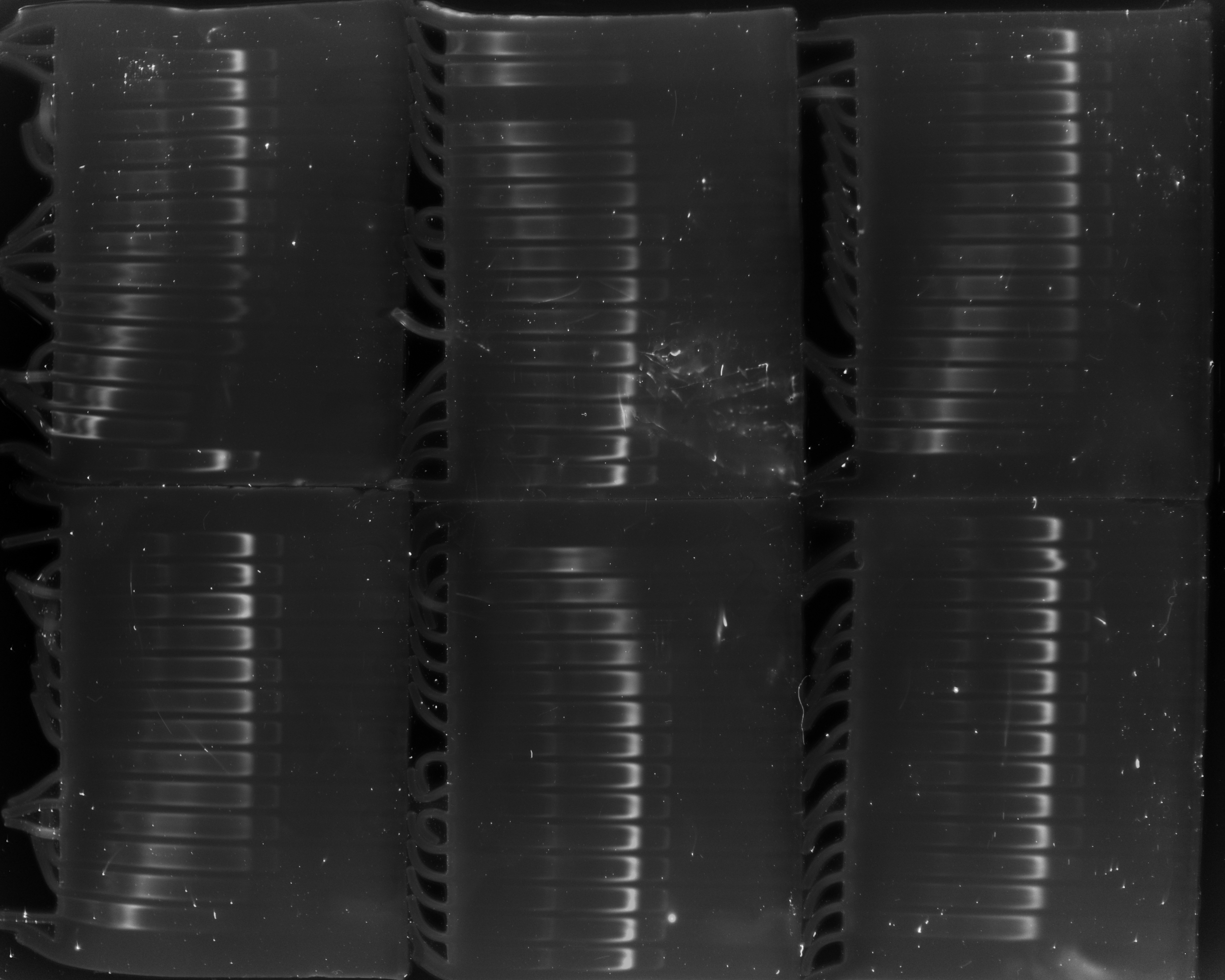

Supplement: Supplementary file 10 — Expanded View Figure Source Data [file 44319_2024_306_MOESM10_ESM.zip › EMBOR-2024-60481V2_SourceDataFor_Expandedview/EMBOR-2024-60481V2_SourceDataForFigEV3/EV3E/Figure EV3E repeat 1.tif]

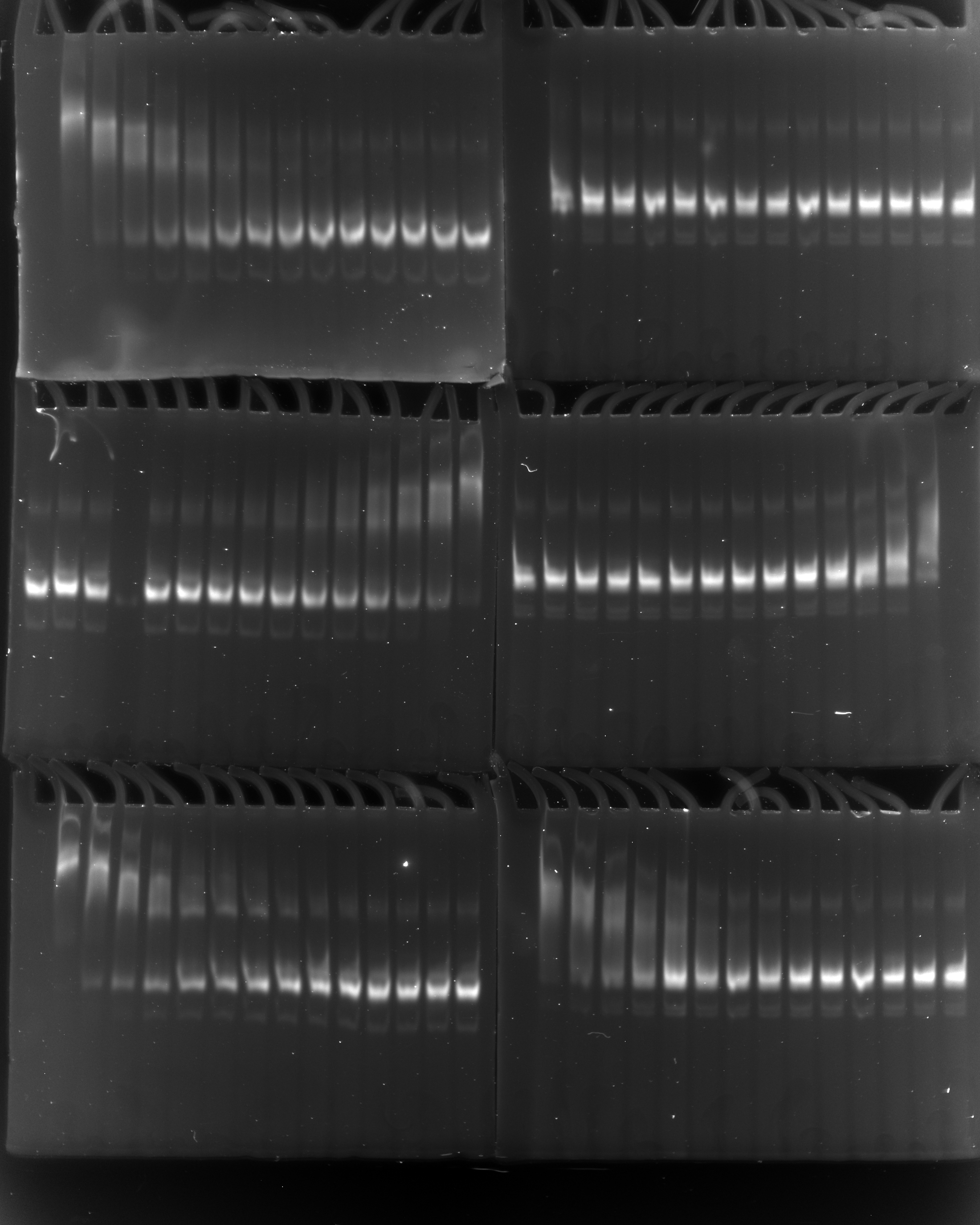

Supplement: Supplementary file 10 — Expanded View Figure Source Data [file 44319_2024_306_MOESM10_ESM.zip › EMBOR-2024-60481V2_SourceDataFor_Expandedview/EMBOR-2024-60481V2_SourceDataForFigEV3/EV3E/Figure EV3E repeat 2.tif]

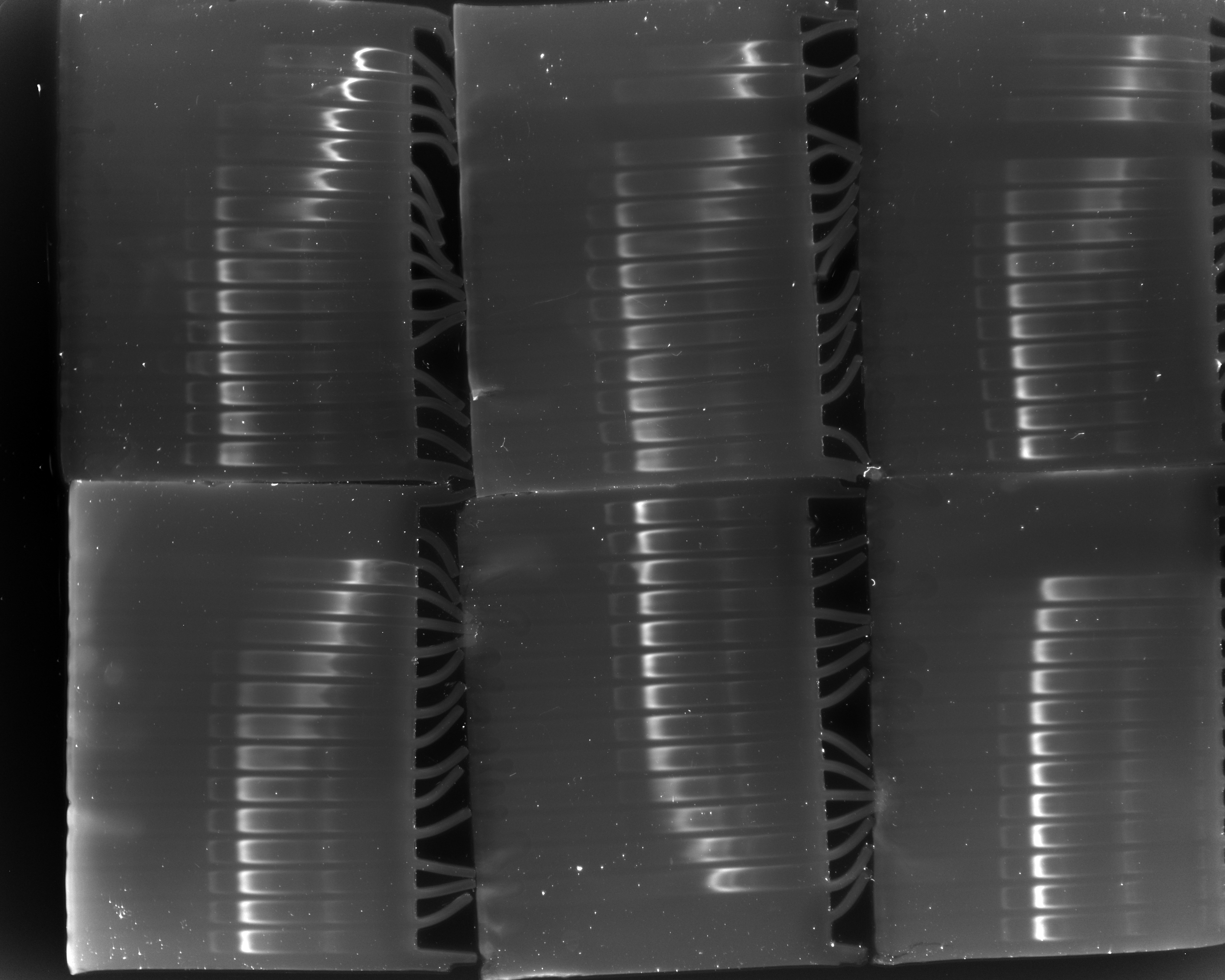

Supplement: Supplementary file 10 — Expanded View Figure Source Data [file 44319_2024_306_MOESM10_ESM.zip › EMBOR-2024-60481V2_SourceDataFor_Expandedview/EMBOR-2024-60481V2_SourceDataForFigEV3/EV3E/Figure EV3E.tif]

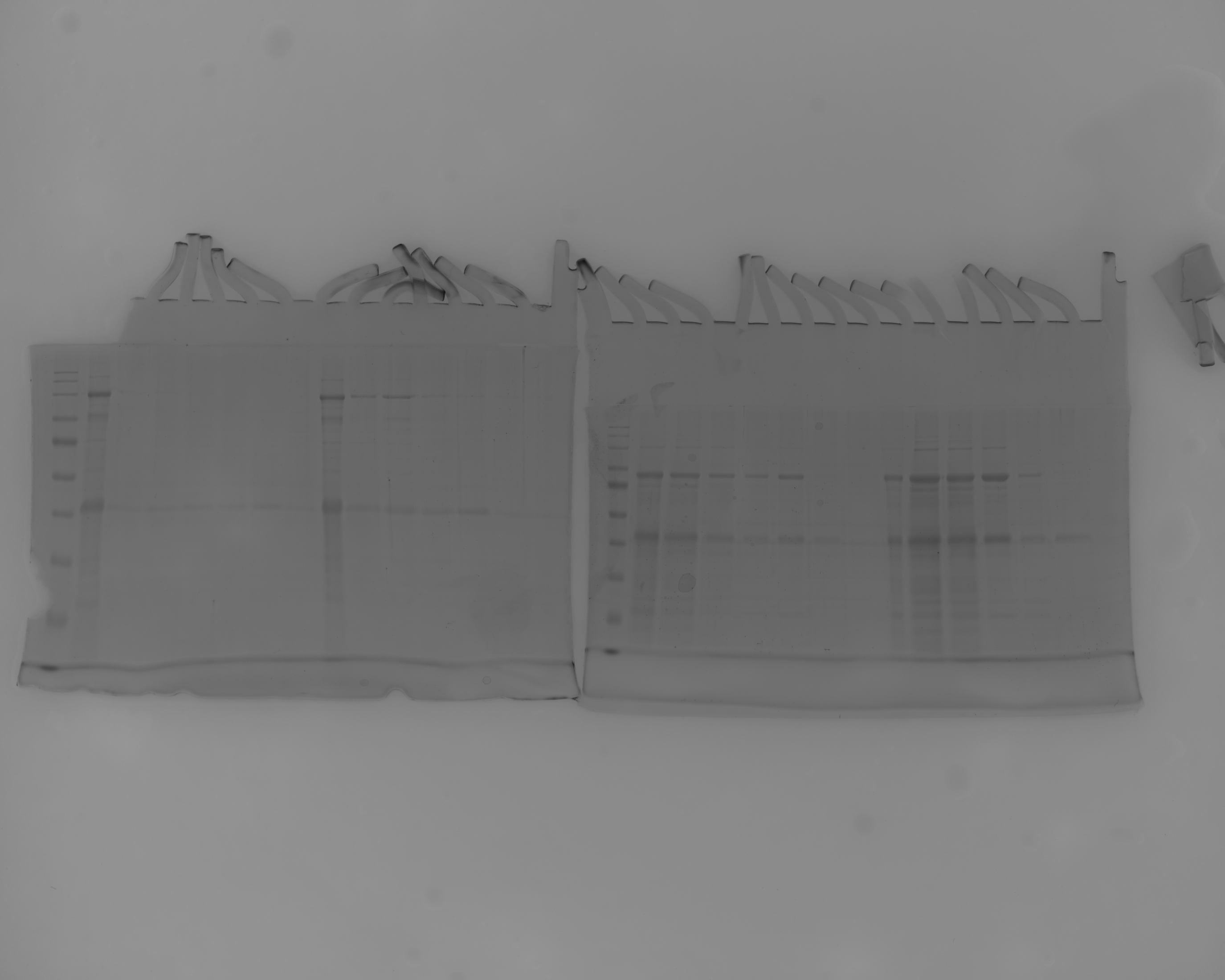

Supplement: Supplementary file 10 — Expanded View Figure Source Data [file 44319_2024_306_MOESM10_ESM.zip › EMBOR-2024-60481V2_SourceDataFor_Expandedview/EMBOR-2024-60481V2_SourceDataForFigEV4/EV4I/221202 hw akta micro runs.tif]

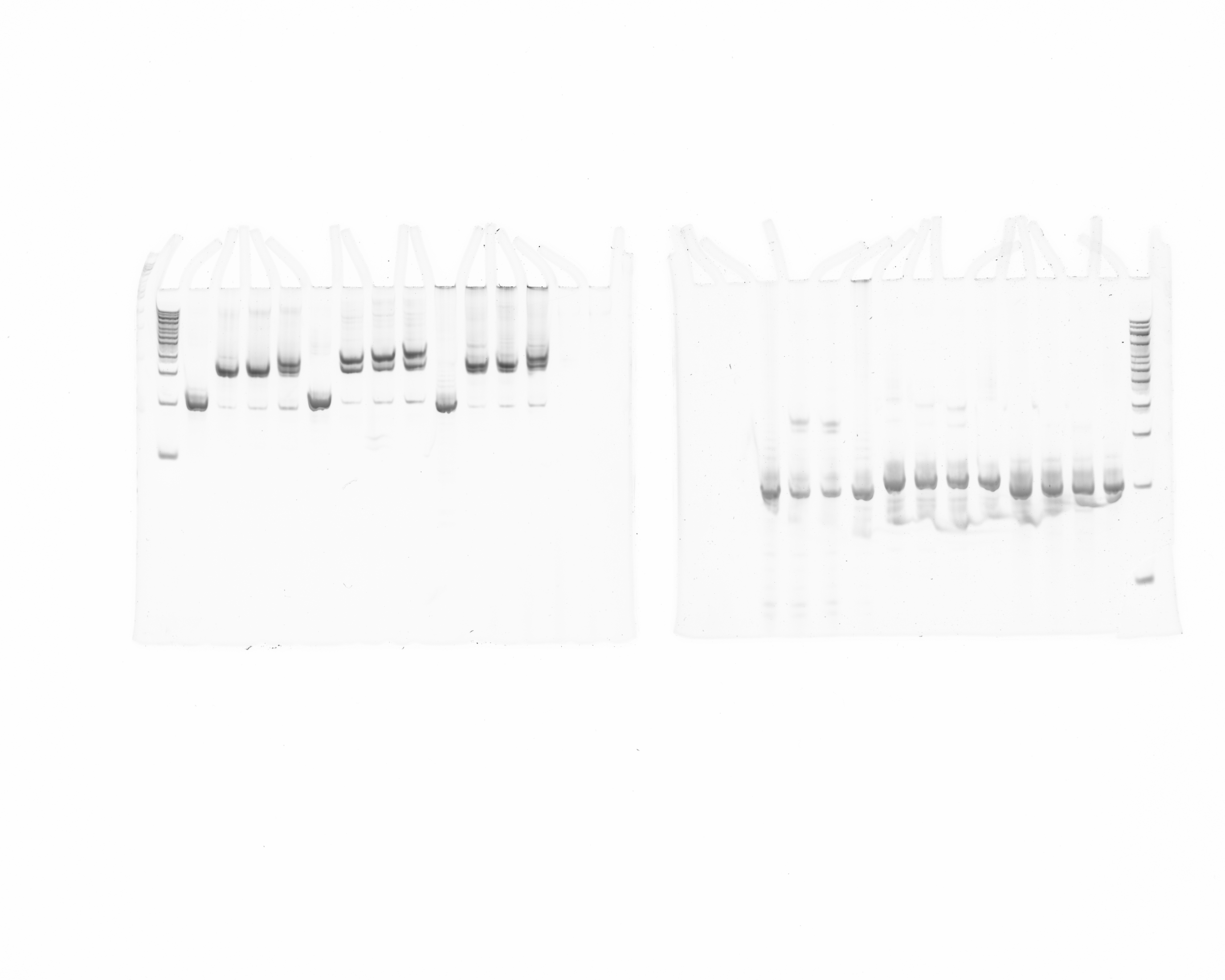

Supplement: Supplementary file 10 — Expanded View Figure Source Data [file 44319_2024_306_MOESM10_ESM.zip › EMBOR-2024-60481V2_SourceDataFor_Expandedview/EMBOR-2024-60481V2_SourceDataForFigEV4/EV4H/230609 ncps after peg.tif]

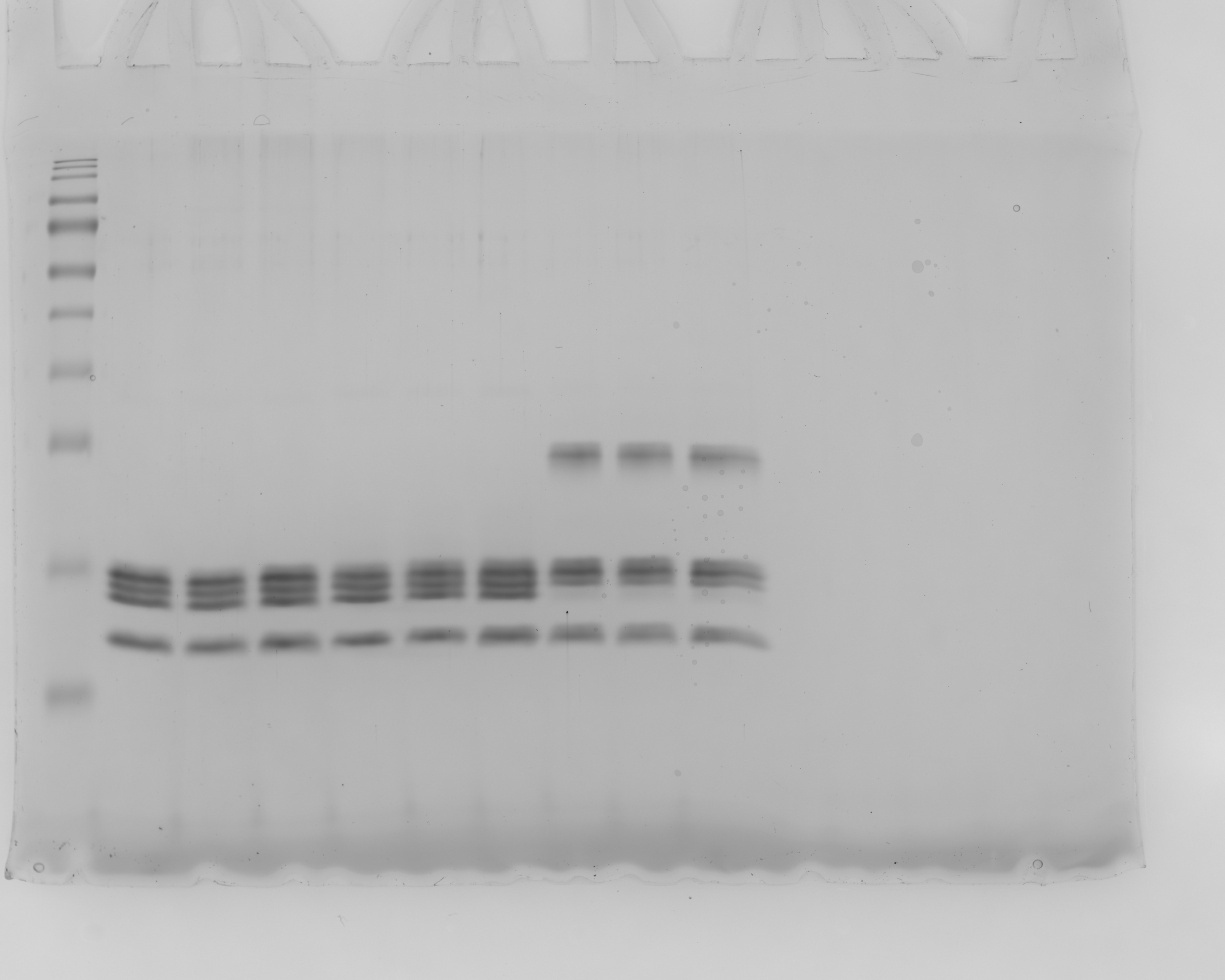

Supplement: Supplementary file 10 — Expanded View Figure Source Data [file 44319_2024_306_MOESM10_ESM.zip › EMBOR-2024-60481V2_SourceDataFor_Expandedview/EMBOR-2024-60481V2_SourceDataForFigEV4/EV4H/2023-06-10 15h30m12s Coomassie Blue 0.714s.tif]

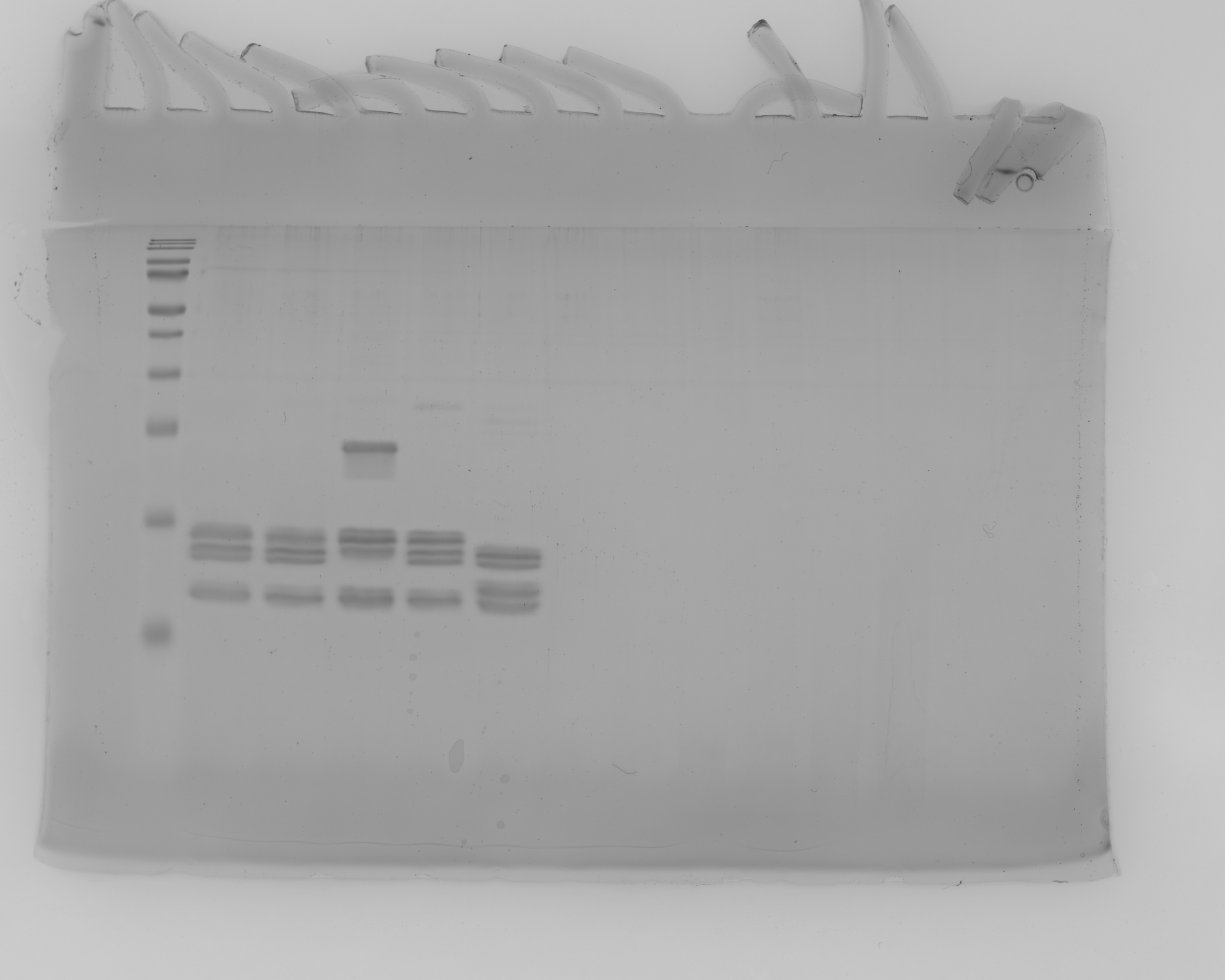

Supplement: Supplementary file 10 — Expanded View Figure Source Data [file 44319_2024_306_MOESM10_ESM.zip › EMBOR-2024-60481V2_SourceDataFor_Expandedview/EMBOR-2024-60481V2_SourceDataForFigEV4/EV4A/230310_sds_page_nuckeosomes_2.tif]

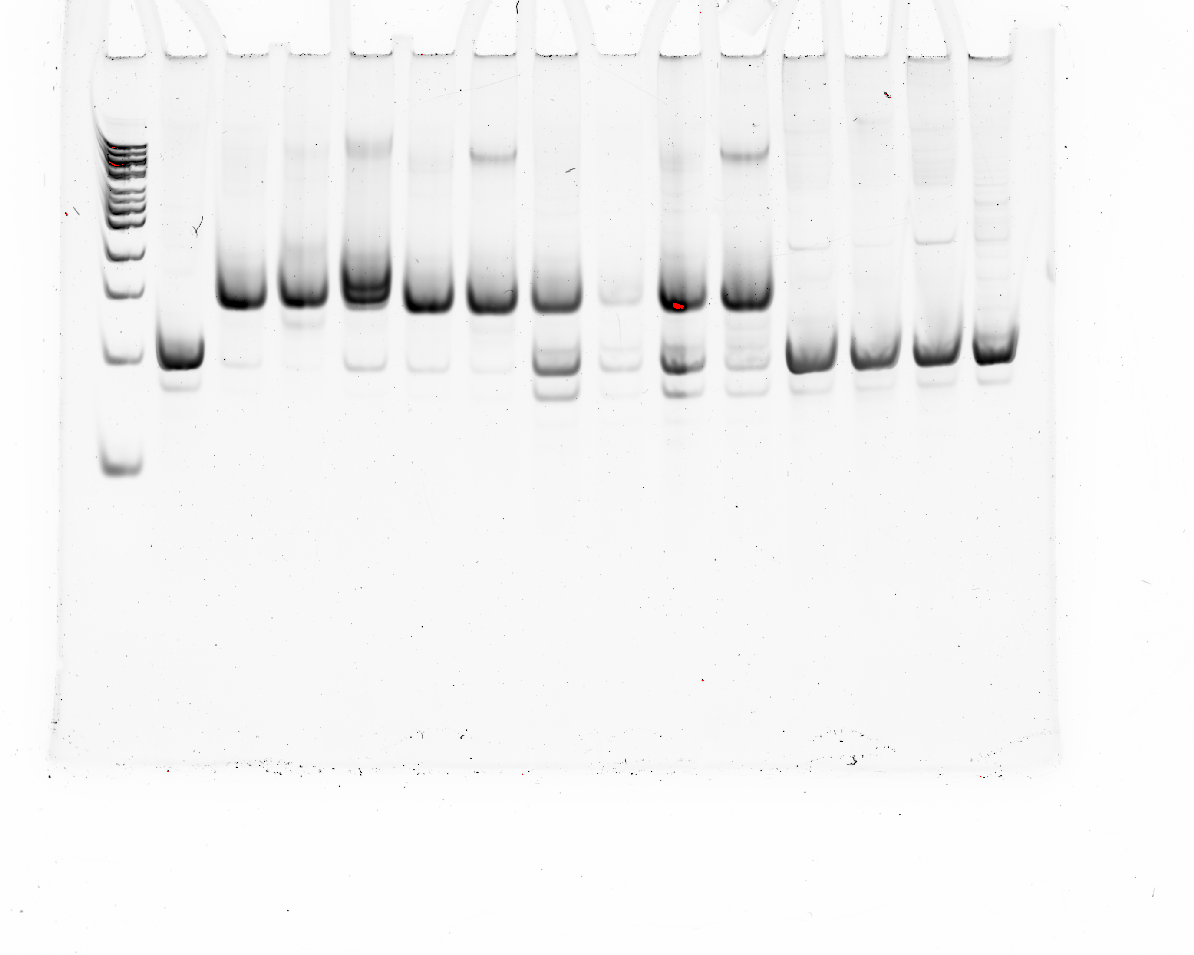

Supplement: Supplementary file 10 — Expanded View Figure Source Data [file 44319_2024_306_MOESM10_ESM.zip › EMBOR-2024-60481V2_SourceDataFor_Expandedview/EMBOR-2024-60481V2_SourceDataForFigEV4/EV4A/230308 hw ncps and dna(SYBR┬« Safe).tif]

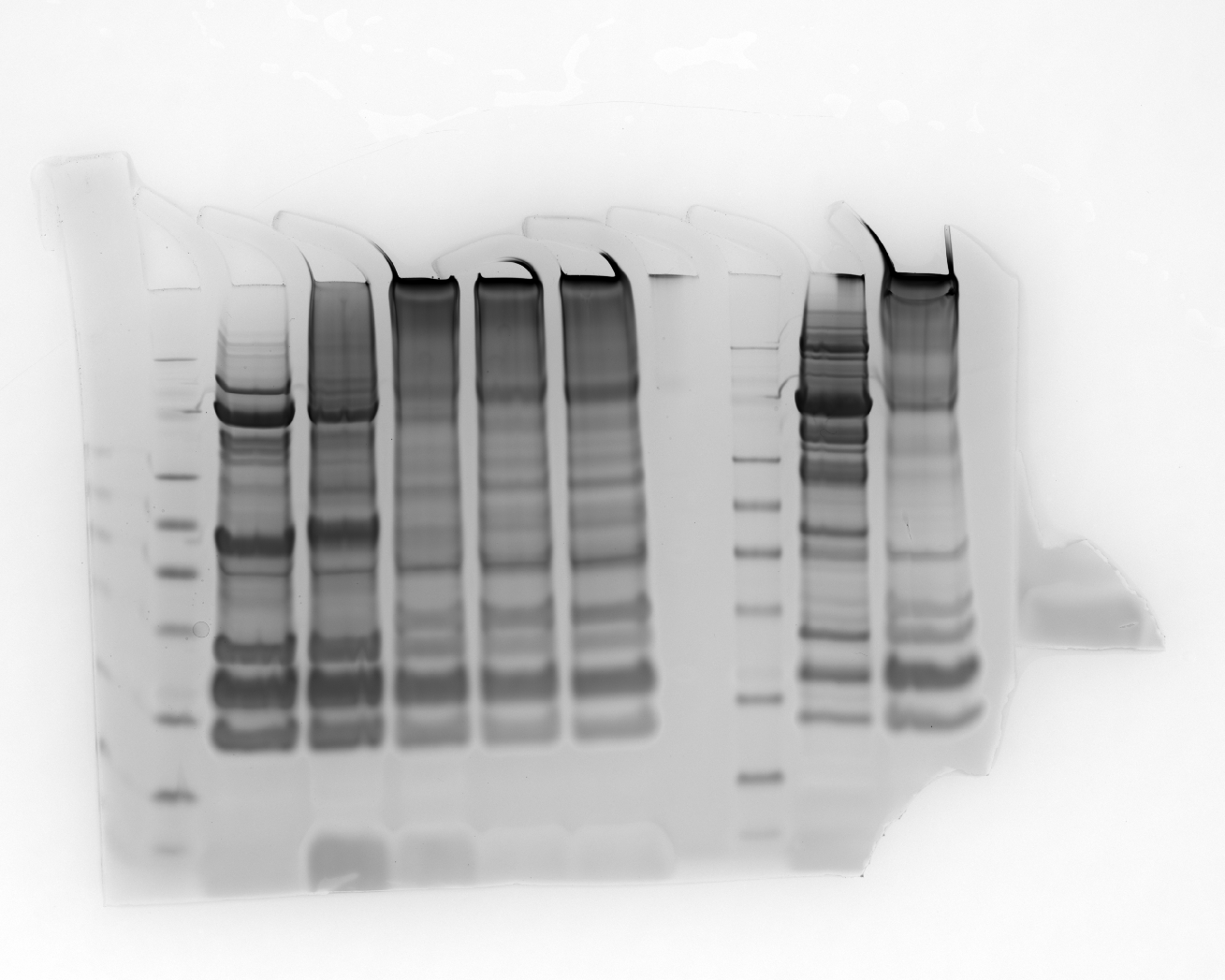

Supplement: Supplementary file 10 — Expanded View Figure Source Data [file 44319_2024_306_MOESM10_ESM.zip › EMBOR-2024-60481V2_SourceDataFor_Expandedview/EMBOR-2024-60481V2_SourceDataForFigEV1/EV1G-H/user 2024-08-06 02h17m10s(Coomassie Blue).tif]
