# Supplementary material for: The N-terminal region of DNMT3A engages the nucleosome surface to aid chromatin recruitment
Source: EMBO Rep. 2024 Nov 11;25(12):5743–79. doi: 10.1038/s44319-024-00306-3 (PMC11624362; doi:10.1038/s44319-024-00306-3)
Supplement: Supplementary file 11 — Expanded View Figures [file 44319_2024_306_MOESM11_ESM.pdf]

Expanded View Figures

Figure EV1. Preparation of full-length DNMT3A1-3L and nucleosomes for enzymology and Cryo-EM.

(**A**) Sequence of 195 bp DNA optimised for the DNMT3A1 catalytic domain, used for nucleosomes in cryo-EM and enzymology experiments. The sequence comprises strong positioning sequence Widom 601 followed by a 50 bp linker. The linker is engineered for optimum DNMT3 catalytic activity (Gao et al, 2020; Mallona et al, 2021) with flanking sites marked in green box and methylated CpG highlighted green. (**B**) Native gels and SDS-PAGE gels of 195 bp DNA, unmodified and H2AKc119ub nucleosomes used in Fig. 1C and for cryo-EM experiments. Nucleosomes (100 ng based on DNA concentration) were loaded on 5% 19:1 acrylamide native gels using native loading buffer (8% sucrose, 0.02 mg/mL BSA, 0.01% bromophenol blue) and run 100 V for 90 min, then stained with Diamond stain. Native gels show shift in mobility of DNA when wrapped into a nucleosome. H2AK119ub nucleosome appears as two bands on gel due to conformation flexibility of the ubiquitin altering mobility. For SDS-PAGE, 900 ng nucleosomes were loaded on 17% 37.5:1 acrylamide gels using SDS-PAGE loading buffer (56 mM Tris pH 6.8, 11% glycerol, 2.4%SDS, 0.016% bromophenol blue) and run 100 V for 10 min then 200 V for approximately 60 min, then stained with colloidal Coomassie blue stain. Denatured gels show equal loading of all four histones and reduced mobility of ubiquitylated H2A. (**C**) Schematic of the purification strategy of full-length DNMT3A1-DNMT3L-StrepII complex used. Full-length His-MBP-DNMT3A1 and His-GFP-DNMT3L-StrepII were co-expressed in *E. coli* BL21 RIL. Cells were lysed and purified with Nickel NTA affinity purification followed by StrepTrap. His-MBP and His-GFP tags were cleaved using TEV protease followed by a further purification using a heparin column. The salt was reduced to 150 mM NaCl and complex was flash frozen and stored at −80 °C. SDS-PAGE gel of purified full-length DNMT3A1-DNMT3L-StrepII complex. Asterisk indicates degradation product. (**D**) A sample of 40 µg of DNMT3A1-DNMT3L-StrepII (1.77 mg/mL) complex was run on analytical size exclusion chromatography to assess monodispersity. Two overlapping peaks (0.9 ml and 1.05 ml elution volume) corresponding to rough sizes for heterotetramer and heterodimeric species. An SDS-PAGE gels was run of all fractions across the peaks. (**E**) Surface rendering of the 5.1 Å DNMT3A1-3L: H2AK119ub Nucleosome at 0.02, 0.01 and 0.0065 threshold. (**F**) Segmented surface rendering of the 5.1 Å DNMT3A1-3L: H2AK119ub Nucleosome. Locally filtered segmented map with nucleosome density displayed at 0.0065 threshold and ubiquitin and UDR density displayed 0.05. Rigid body fitting of nucleosome model (PDB 7VVU (Qu et al, 2022)), guided segmentation to identify non-nucleosome features including ubiquitin/UDR density (purple), nucleosome contacting UDR density (cyan) and linker DNA adjacent DNMT3A1-DNMT3L density (cyan). (**G**) Cross-linking mass spectrometry of full-length DNMT3A1-DNMT3L-strepII to H2AKc119ub nucleosomes wrapped with 195 bp DNA. (**H**) Circular representations of combined detected cross-links over a range of EDC ratios (7.5–30:1 molar ratio) measured in two separate experiments. (left) Only cross-links with DNMT3A1 shown. (middle) Cross-links to DNMT3A1 mapped on a nucleosome model (PDB 1AOI (Luger et al, 1997)) with ubiquitin docked close to H2AK119 and DNA extended to 195 bp. More abundant cross-linked residues shown as spheres, less as stick representation. Model coloured, Yellow, H2A; Red, H2B; Blue, H3; Green, H4. (right) all inter- and intra-protein cross-links detected in the two experiments. Source data are available online for this figure.

© The Author(s)

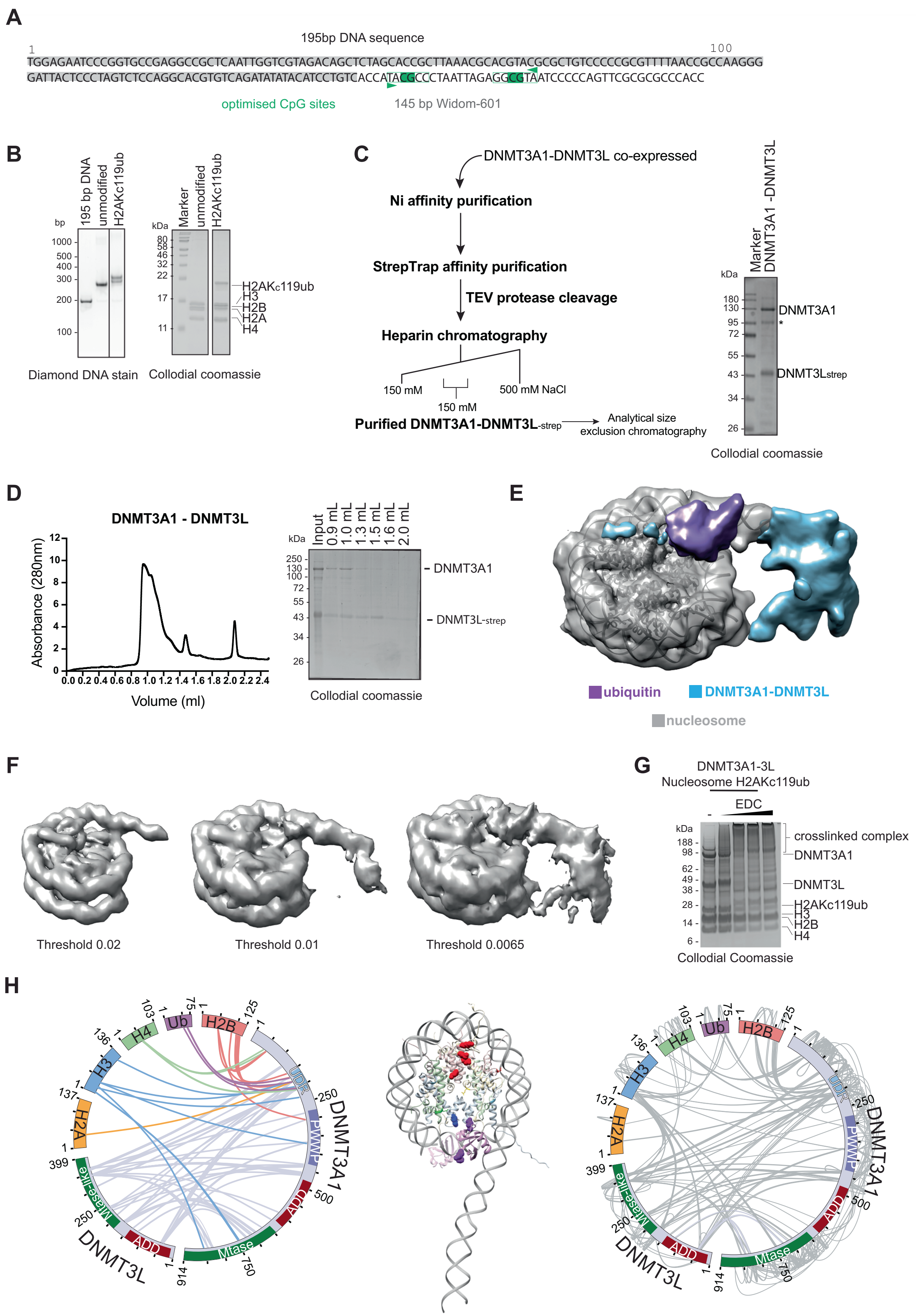
A
195bp DNA sequence
1
100
TGGAGAATCCCGGTGCCGAGGCCGCTCAATTGGTCGTAGACAGCTCTAGCACCGCTTAAACGCACGTACGCGCTGTCCCCCGCGTTTTAACCGCCAAGGG
GATTACTCCCTAGTCTCCAGGCACGTGTCAGATATATACATCCTGTCACCATACGCCCTAATTAGAGGCGTAATCCCCCAGTTCGCGCGCCCACC
optimised CpG sites
145 bp Widom-601
B
bp
195 bp DNA
unmodified
H2AKc119ub
1000
500
400
300
200
100
Diamond DNA stain
kDa
Marker
unmodified
H2AKc119ub
80
58
46
32
22
17
11
H2AKc119ub
H3
H2B
H2A
H4
Collodial coomassie
C
DNMT3A1-DNMT3L co-expressed
Ni affinity purification
StrepTrap affinity purification
TEV protease cleavage
Heparin chromatography
150 mM
500 mM NaCl
150 mM
Purified DNMT3A1-DNMT3L-strep
Analytical size exclusion chromatography
kDa
Marker
DNMT3A1 -DNMT3L
180
130
95
72
55
43
34
26
DNMT3A1
*
DNMT3Lstrep
Collodial coomassie
D
DNMT3A1 - DNMT3L
Absorbance (280nm)
Volume (ml)
kDa
Input
0.9 mL
1.0 mL
1.3 mL
1.5 mL
1.6 mL
2.0 mL
250
130
100
72
55
43
34
26
DNMT3A1
DNMT3L-strep
Collodial coomassie
E
ubiquitin
DNMT3A1-DNMT3L
nucleosome
F
Threshold 0.02
Threshold 0.01
Threshold 0.0065
G
DNMT3A1-3L
Nucleosome H2AKc119ub
EDC
kDa
188
98
62
49
38
28
14
6
crosslinked complex
DNMT3A1
DNMT3L
H2AKc119ub
H3
H2B
H4
Collodial Coomassie
H
H4
Ub
H2B
H3
H2A
UDR
PWWP
ADD
Mtase
DNMT3A1
Mtase-like
ADD
DNMT3L

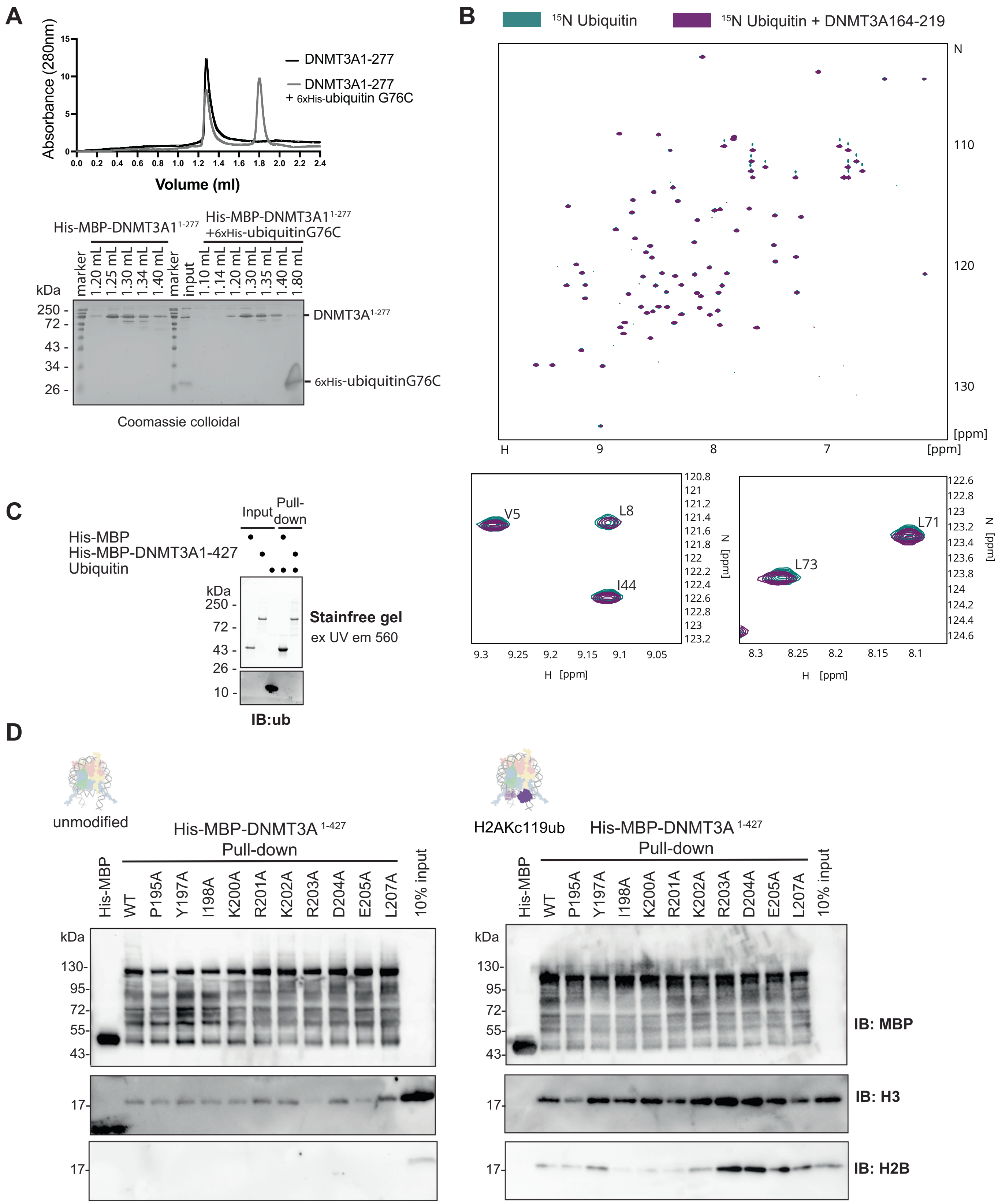
A
Absorbance (280nm)
Volume (ml)
DNMT3A1-277
DNMT3A1-277
+ 6xHis-ubiquitin G76C
His-MBP-DNMT3A1 1-277
His-MBP-DNMT3A1 1-277
+6xHis-ubiquitinG76C
marker
1.20 mL
1.25 mL
1.30 mL
1.34 mL
1.40 mL
input
1.10 mL
1.14 mL
1.35 mL
1.80 mL
kDa
DNMT3A 1-277
6xHis-ubiquitinG76C
Coomassie colloidal
B
15N Ubiquitin
15N Ubiquitin + DNMT3A164-219
V5
L8
I44
L71
L73
H [ppm]
N [ppm]
C
Input
Pull-down
His-MBP
His-MBP-DNMT3A1-427
Ubiquitin
Stainfree gel
ex UV em 560
IB:ub
D
unmodified
H2AKc119ub
His-MBP-DNMT3A 1-427
Pull-down
His-MBP
WT
P195A
Y197A
I198A
K200A
R201A
K202A
R203A
D204A
E205A
L207A
10% input
IB: MBP
IB: H3
IB: H2B

Figure EV2. DNMT3A1 UDR region alone does not interact with ubiquitin.

(**A**) Size exclusion chromatography of DNMT3A1$^{1\text{-}277}$ with and without 6xHis-ubiquitin. DNMT3A1$^{1\text{-}277}$ (20 µg) was loaded on a Superdex 200 Increase 3.2/300 (Cytiva) column with (grey) or without (black) first incubating with 5x molar excess of 6xHis-ubiquitin (20 µg). An SDS-PAGE gel was run of the fractions of each peak. (**B**) HSQC NMR of ^{15}N ubiquitin with and without DMT3A$^{164\text{-}219}$. Spectra were taken of ^{15}N ubiquitin (green), then DNMT3A1$^{164\text{-}219}$ was added (purple). (bottom) magnified view of assigned residues, highlighting crosspeaks for canonical hydrophobic patch residues (left) and C-terminal hydrophobic patch region (right). (**C**) Pull down assay measuring binding of 6xHis-ubiquitin to His-MBP-DNMTA1$^{1\text{-}427}$. His-MBP-DNMTA1$^{1\text{-}427}$ (50 µg) was immobilised on amylose beads (50 µL) and 6xHis-ubiquitin (50 µg) was added. Bound proteins were detected using stainfree UV detection of Mini-PROTEAN TGX Stain-Free Precast Gels. 6xHis-ubiquitin was detected using western blot followed by detection with antibodies against ubiquitin. (**D**) Pull-down assay using partially purified His-MBP, His-MBP-DNMT3A1$^{1\text{-}427}$ and His-MBP-DNMT3A1$^{1\text{-}427}$ alanine scanning mutants in the proposed ubiquitin interacting region of the UDR. Proteins were expressed and purified on Nickel affinity beads, eluted and bound to amylose beads prior to incubation with unmodified nucleosomes (left) and H2AKc119ub nucleosomes (right), prior to washing and detection by western blot. Source data are available online for this figure.

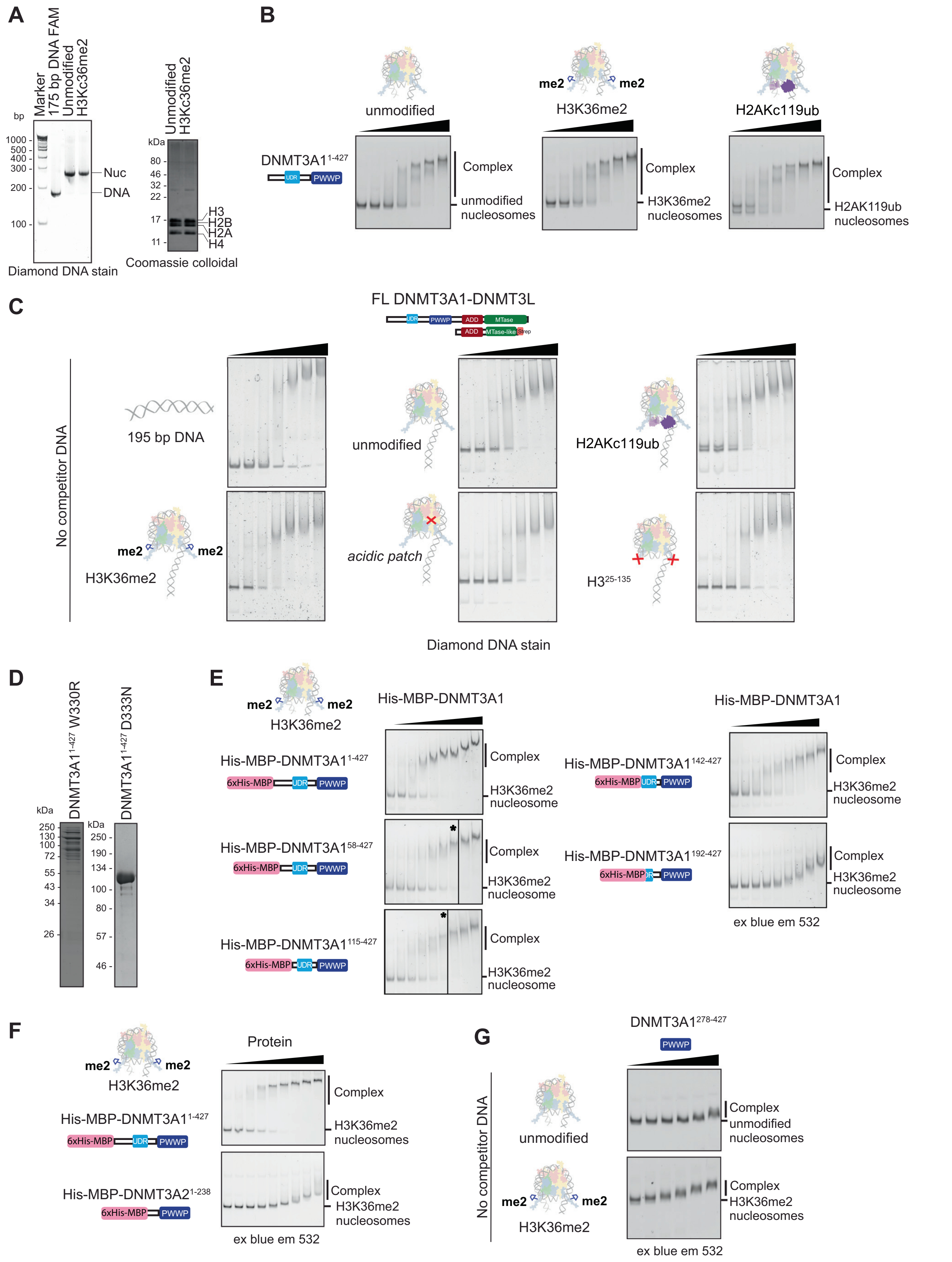
A
Marker
175 bp DNA FAM
Unmodified
H3Kc36me2
bp
1000
500
400
300
200
100
Nuc
DNA
Diamond DNA stain
Unmodified
H3Kc36me2
kDa
80
46
32
22
17
11
H3
H2B
H2A
H4
Coomassie colloidal
B
unmodified
me2
me2
H3K36me2
H2AKc119ub
DNMT3A1 1-427
UDR
PWWP
Complex
unmodified nucleosomes
Complex
H3K36me2 nucleosomes
Complex
H2AK119ub nucleosomes
C
FL DNMT3A1-DNMT3L
UDR
PWWP
ADD
MTase
ADD
MTase-like
Strep
No competitor DNA
195 bp DNA
unmodified
H2AKc119ub
me2
me2
H3K36me2
acidic patch
H3 25-135
Diamond DNA stain
D
DNMT3A1 1-427 W330R
DNMT3A1 1-427 D333N
kDa
250
130
100
72
55
43
34
26
kDa
250
190
134
100
80
57
46
E
me2
me2
H3K36me2
His-MBP-DNMT3A1
His-MBP-DNMT3A1 1-427
6xHis-MBP
UDR
PWWP
Complex
H3K36me2 nucleosome
His-MBP-DNMT3A1 58-427
6xHis-MBP
UDR
PWWP
Complex
H3K36me2 nucleosome
His-MBP-DNMT3A1 115-427
6xHis-MBP
UDR
PWWP
Complex
H3K36me2 nucleosome
His-MBP-DNMT3A1
His-MBP-DNMT3A1 142-427
6xHis-MBP
UDR
PWWP
Complex
H3K36me2 nucleosome
His-MBP-DNMT3A1 192-427
6xHis-MBP
PWWP
Complex
H3K36me2 nucleosome
ex blue em 532
F
me2
me2
H3K36me2
Protein
His-MBP-DNMT3A1 1-427
6xHis-MBP
UDR
PWWP
Complex
H3K36me2 nucleosomes
His-MBP-DNMT3A2 1-238
6xHis-MBP
PWWP
Complex
H3K36me2 nucleosomes
ex blue em 532
G
DNMT3A1 278-427
PWWP
No competitor DNA
unmodified
me2
me2
H3K36me2
Complex
unmodified nucleosomes
Complex
H3K36me2 nucleosomes
ex blue em 532

 © The Author(s)

Figure EV3. DNMT3A1-H3K36me2 nucleosome interaction is localised in N-terminal region and PWWP domains.

(**A**) Native gels and SDS-PAGE gels of H3Kc36me2 nucleosomes. (**B**) EMSAs showing binding of DNMT3A1 1-427 after cleaving His-MBP to nucleosomes wrapped with FAM labelled 175 bp DNA. DNMT3A1 1-427 (0–10 µM, 2x dilution series) were mixed with unmodified, H3K36me2 and H2AKc119ub nucleosomes wrapped with 5′ 6-FAM labelled 175 bp DNA (5.4 nM) and incubated for 1 h on ice. Complexes were resolved by native-PAGE and imaged using blue light excitation and 532 nm emission filters. Gels show concentrations 9.8–1250 nM. (**C**) EMSA comparing binding of full-length DNMT3A1-DNMT3L-StrepII to 195 bp-wrapped unmodified, H3K36me2, H2AKc119ub, acidic patch (H2A$^{E61A/E91A/E92A}$ and H2B^{E105A}) and H3$^{25-135}$ nucleosomes and free 195 bp DNA. Limiting amounts (8 nM) of nucleosomes or DNA were incubated with increasing concentrations (0–2666 nM, 2x dilution series) of full-length DNMT3A1-DNMT3L-StrepII. Complexes were resolved by native-PAGE, stained with diamond stain and imaged using blue light. Representative gels show concentrations 41–2666 nM of one of two independent experiments. EMSA of unmodified, H2AKc119ub and DNA alone also appears in Fig. 1B. (**D**) SDS-PAGE gel of purified DNMT3A proteins used in Fig. 4B. (**E**) EMSA comparing binding of DNMT3A1 constructs with different lengths of the N-terminal region to H3K36me2 nucleosomes wrapped with 5′ FAM labelled 175 bp Widom601 DNA. Limiting amounts (2.3 nM) of H3K36me2 nucleosomes were incubated with increasing concentrations (0–8000 nM) of His-MBP-DNMT3A1 constructs. Complexes were resolved by native-PAGE and imaged using blue light excitation and 532 nm emission filters. Representative gels show concentrations 25–8000 nM of one of three experiments. (**F**) EMSA comparing DNMT3A splice isoform fragments DNMT3A1$^{1-427}$ and DNMT3A2$^{1-238}$. Limiting amounts (2.3 nM) of H3K36me2 nucleosomes were incubated with increasing concentrations (0–8000 nM) of protein. Complexes were resolved by native-PAGE and imaged using blue light excitation and 532 nm emission filters. Gels show concentrations 32–8000 nM. (**G**) EMSAs showing binding of DNMT3A1 278-427 (PWWP domain alone) after cleaving His-MBP to nucleosomes wrapped with FAM labelled 175 bp DNA. DNMT3A1 278-427 (0–25 µM, 1.5x dilution series) were mixed with unmodified and H3K36me2 nucleosomes wrapped with 5′ 6-FAM labelled 175 bp DNA (5.4 nM) and incubated for 1 h on ice. Complexes were resolved by native-PAGE and imaged using blue light excitation and 532 nm emission filters. Gels show concentrations 650–5000 nM. Source data are available online for this figure.

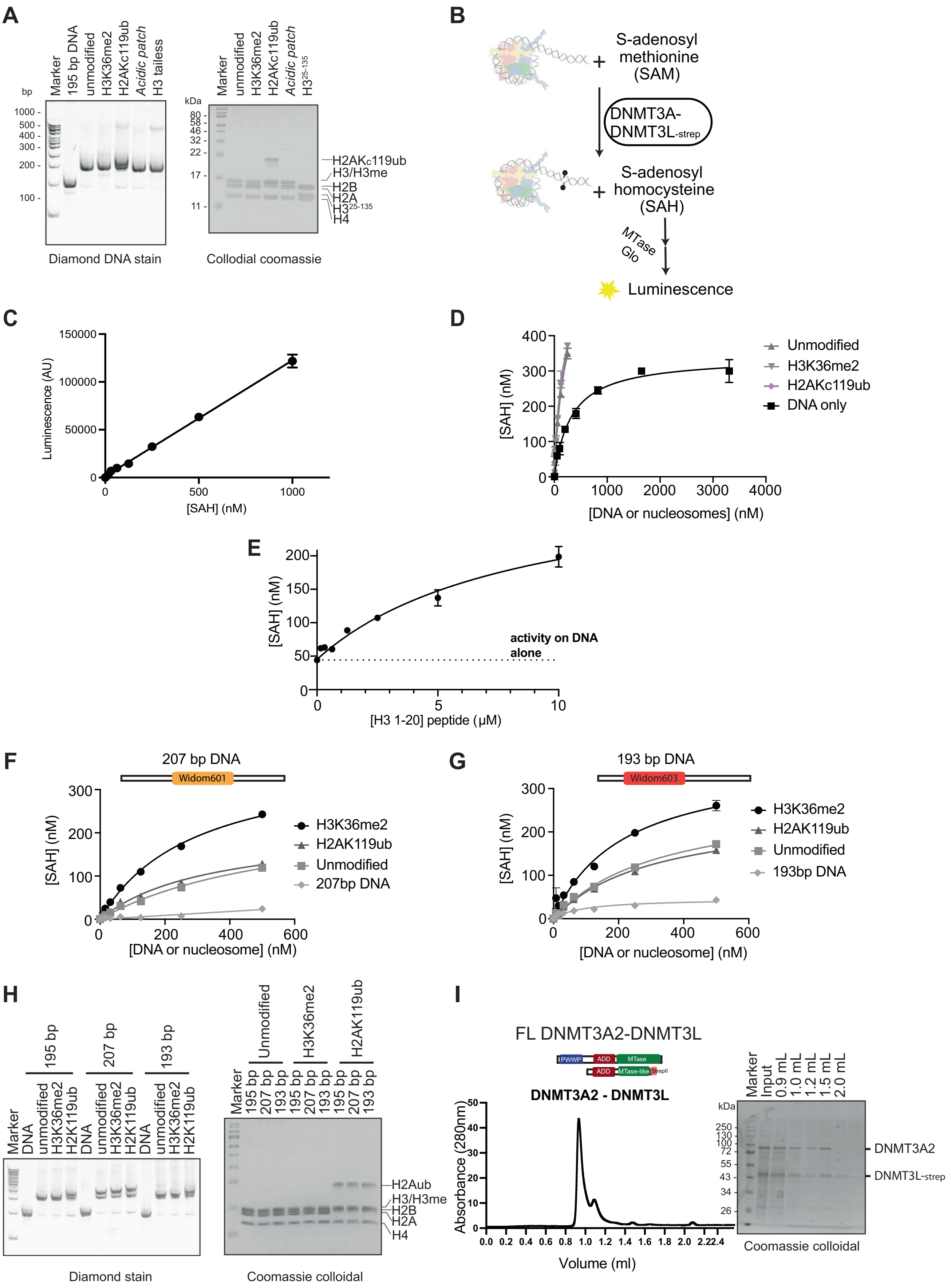
A
Marker
195 bp DNA
unmodified
H3K36me2
H2AKc119ub
Acidic patch
H3 tailess
bp
1000
500
400
300
200
100
Diamond DNA stain
Marker
unmodified
H3K36me2
H2AKc119ub
Acidic patch
H3 25-135
kDa
80
58
46
32
22
17
11
H2AKc119ub
H3/H3me
H2B
H2A
H3 25-135
H4
Collodial coomassie
B
S-adenosyl methionine (SAM)
DNMT3A-DNMT3L-strep
S-adenosyl homocysteine (SAH)
MTase Glo
Luminescence
C
Luminescence (AU)
[SAH] (nM)
D
[SAH] (nM)
[DNA or nucleosomes] (nM)
Unmodified
H3K36me2
H2AKc119ub
DNA only
E
[SAH] (nM)
activity on DNA alone
[H3 1-20] peptide (μM)
F
207 bp DNA
Widom601
[SAH] (nM)
[DNA or nucleosome] (nM)
H3K36me2
H2AK119ub
Unmodified
207bp DNA
G
193 bp DNA
Widom603
[SAH] (nM)
[DNA or nucleosome] (nM)
H3K36me2
H2AK119ub
Unmodified
193bp DNA
H
195 bp
207 bp
193 bp
Marker
DNA
unmodified
H3K36me2
H2K119ub
Diamond stain
Unmodified
H3K36me2
H2AK119ub
Marker
195 bp
207 bp
193 bp
H2Aub
H3/H3me
H2B
H2A
H4
Coomassie colloidal
I
FL DNMT3A2-DNMT3L
PWWP
ADD
MTase
MTase-like
StrepII
DNMT3A2 - DNMT3L
Absorbance (280nm)
Volume (ml)
Marker
Input
0.9 mL
1.0 mL
1.2 mL
1.5 mL
2.0 mL
kDa
250
130
100
72
55
43
34
26
DNMT3A2
DNMT3L-strep
Coomassie colloidal

Figure EV4. DNMT3A1 activity differs on different nucleosome substrates.

(**A**) Native gels and SDS-PAGE gels of modified and unmodified nucleosomes wrapped with 195 Widom601 DNA used in used for enzymology in Fig. 6 and EMSA experiments in Fig. EV3B. The unmodified, H2AK119ub and DNA SDS-PAGE and Native gels are duplicated from Fig. EV1B. (**B**) Schematic of Promega MTase-Glo™ Methyltransferase Assay. (**C**) Representative S-Adenosyl-L-homocysteine (SAH) standard curve used to convert luminescence units to SAH concentration. (**D**) Methyltransferase assay showing activity of full-length DNMT3A1-DNMT3L-StrepII on high concentrations (0–3300 nM) of 195 bp DNA. Activities on unmodified, H3K36me2 and H2AK119ub nucleosomes (0–250 nM) are shown as comparison. DNMT3A1-DNMT3L was incubated with increasing concentrations of DNA or nucleosomes for 1 h at 37 °C. Methyltransferase activity was detected using Promega MTase-Glo™ Methyltransferase Assay. Experiment done in duplicate. Michaelis-Menten curves were fit using GraphPad Prism 10. (**E**) Methyltransferase assay showing activity of full-length DNMT3A1-DNMT3L-StrepII on free 195 bp DNA in the presence of H3 (H1-20) peptide. The level of activity in the absence of H3 peptide is marked by a dotted line. Methyltransferase activity was detected using Promega MTase-Glo™ Methyltransferase Assay. Experiment done in duplicate. Michaelis-Menten curves were fit using GraphPad Prism 10. (**F**) Methyltransferase activity of full-length DNMT3A1-DNMT3L-StrepII on unmodified, H3K36me2, H2AKc119ub wrapped with 207 bp Widom601 DNA and free 207 bp DNA. DNMT3A1-DNMT3L was incubated with increasing concentrations of nucleosomes for 1 h at 37 °C. Methyltransferase activity was detected using Promega MTase-Glo™ Methyltransferase Assay. Experiment done in duplicate. Michaelis-Menten curves were fit using GraphPad Prism 10. (**G**) Methyltransferase activity of full-length DNMT3A1-DNMT3L-StrepII on unmodified, H3K36me2, H2AKc119ub nucleosomes wrapped with 193 bp Widom603 DNA and free 193 bp DNA. DNMT3A1-DNMT3L was incubated with increasing concentrations of nucleosomes for 1 h at 37 °C. Methyltransferase activity was detected using Promega MTase-Glo™ Methyltransferase Assay. Experiment done in duplicate. Michaelis-Menten curves were fit using GraphPad Prism 10. (**H**) Native gels and SDS-PAGE gels of modified and unmodified nucleosomes used in Fig. EV4F,G. (**I**) Analytical size exclusion chromatography of full-length DNMT3A2-DNMT3L-StrepII. 40 µg protein at 2 mg/ml was loaded on a Superdex 200 Increase 3.2/300 (Cytiva) column. An SDS-PAGE gels was run of all fractions across the peaks. Source data are available online for this figure.

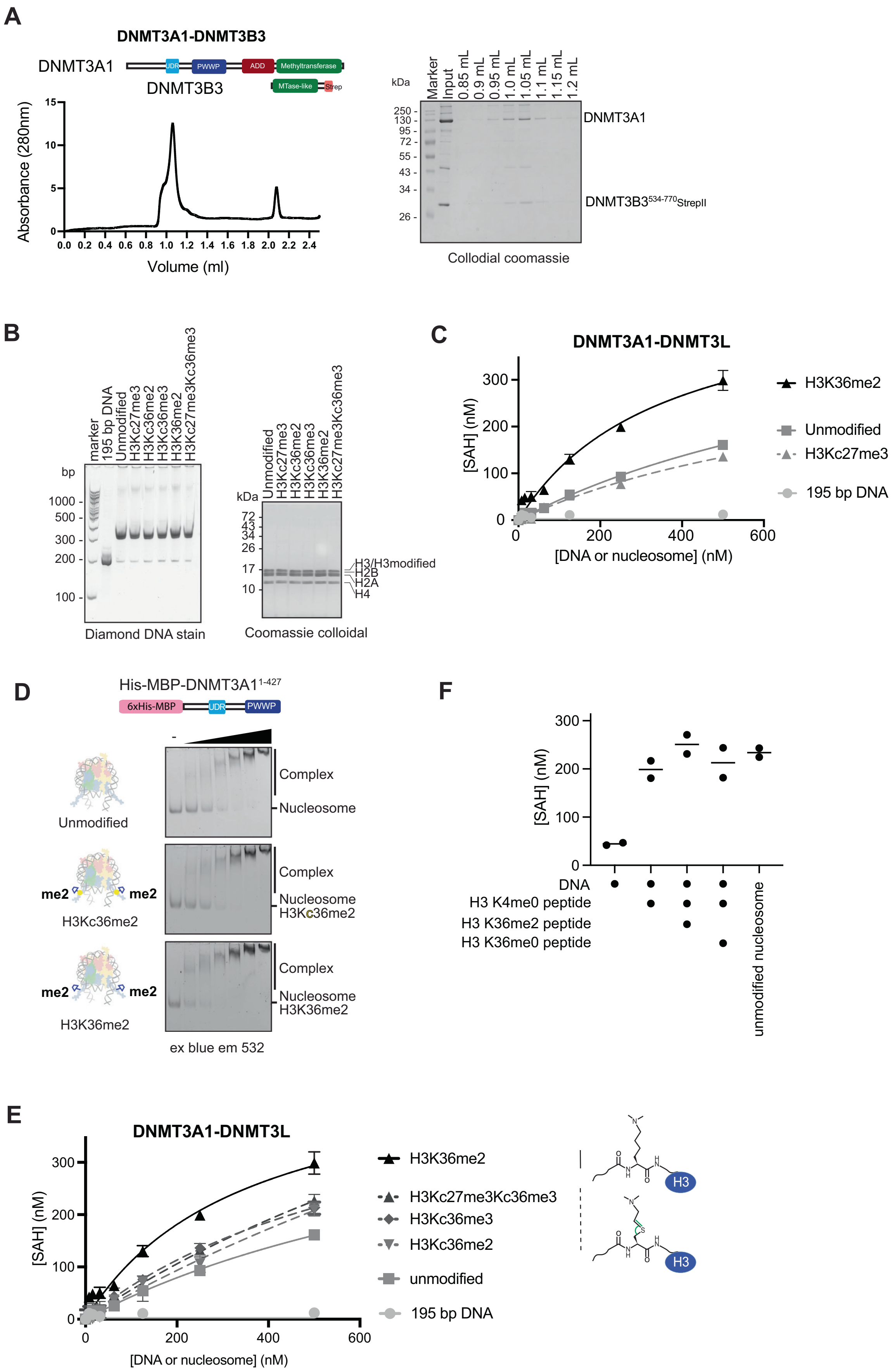
A
DNMT3A1-DNMT3B3
DNMT3A1
DNMT3B3
UDR
PWWP
ADD
Methyltransferase
MTase-like
Strep
Absorbance (280nm)
Volume (ml)
kDa
Marker
Input
0.85 mL
0.9 mL
0.95 mL
1.0 mL
1.05 mL
1.1 mL
1.15 mL
1.2 mL
DNMT3A1
DNMT3B3534-770StrepII
Collodial coomassie
B
marker
195 bp DNA
Unmodified
H3Kc27me3
H3Kc36me2
H3Kc36me3
H3K36me2
H3Kc27me3Kc36me3
bp
Diamond DNA stain
kDa
H3/H3modified
H2B
H2A
H4
Coomassie colloidal
C
DNMT3A1-DNMT3L
[SAH] (nM)
[DNA or nucleosome] (nM)
H3K36me2
Unmodified
H3Kc27me3
195 bp DNA
D
His-MBP-DNMT3A11-427
6xHis-MBP
UDR
PWWP
Complex
Nucleosome
Unmodified
me2
H3Kc36me2
Nucleosome H3Kc36me2
H3K36me2
Nucleosome H3K36me2
ex blue em 532
F
[SAH] (nM)
DNA
H3 K4me0 peptide
H3 K36me2 peptide
H3 K36me0 peptide
unmodified nucleosome
E
DNMT3A1-DNMT3L
[SAH] (nM)
[DNA or nucleosome] (nM)
H3K36me2
H3Kc27me3Kc36me3
H3Kc36me3
H3Kc36me2
unmodified
195 bp DNA
H3

 © The Author(s)

Figure EV5. DNMT3A activity is only weakly affected by combinatorial PWWP-UDR status readout.

(**A**) Analytical size exclusion chromatography of DNMT3A1-DNMT3B3$^{534\text{-}770}$-StrepII. 18 µg protein at 1.8 mg/ml was loaded on a Superdex 200 Increase 3.2/300 (Cytiva) column. An SDS-PAGE gels was run of all fractions across the peaks. (**B**) Native gels and SDS-PAGE gels of nucleosomes used for enzymology experiments in Fig. EV5C,E. (**C**) Methyltransferase activity of full-length DNMT3A1-DNMT3L-StrepII on unmodified, H3K36me2, H3Kc27me3 nucleosomes wrapped with 195 bp Widom601 DNA and free 195 bp DNA. DNMT3A1-DNMT3L was incubated with increasing concentrations of nucleosomes for 1 h at 37 °C. Methyltransferase activity was detected using Promega MTase-Glo™ Methyltransferase Assay. Experiment done in duplicate. Michaelis-Menten curves were fit using GraphPad Prism 10. (**D**) EMSA assay comparing DNMT3A1$^{1\text{-}427}$ interaction with unmodified, H3K36me2 and Methyl-lysine analogue H3Kc36me2 nucleosomes. Methyl-lysine analogues bind less well compared to true native methylated bond due too differences in the gamma-position on the modified sidechain. (**E**) Methyltransferase assay comparing activity of DNMT3A1-DNMT3L on nucleosomes with different H3 methylation states. Methyltransferase activity was detected using Promega MTase-Glo™ Methyltransferase Assay. Experiment done in duplicate. Michaelis-Menten curves were fit using GraphPad Prism 10. (left) Schematic of the H3 methyl lysine analogues and methylated lysine, green highlights carbon-sulphur bond differences in bond angle and bond length compared to carbon-carbon bond. (**F**) Methyltransferase assay showing activity of full-length DNMT3A1-DNMT3L-StrepII on free 195 bp DNA in the presence of differently methylated peptides (H3 1-20, H3$^{26\text{-}46}$K36me2, H3$^{21\text{-}44}$K36me0. Methyltransferase activity was detected using Promega MTase-Glo™ Methyltransferase Assay. Experiment done in duplicate. Michaelis-Menten curves were fit using GraphPad Prism 10. Source data are available online for this figure.
